# Impact of BMI on the survival outcomes of non-small cell lung cancer patients treated with immune checkpoint inhibitors: a meta-analysis

**DOI:** 10.1186/s12885-023-11512-y

**Published:** 2023-10-23

**Authors:** Tongtong Zhang, Shuluan Li, Jianhua Chang, Yan Qin, Chao li

**Affiliations:** 1 Department of Medical Oncology, National Cancer Center, National Clinical Research Center for Cancer/Cancer Hospital & Shenzhen Hospital, Chinese Academy of Medical Sciences and Peking Union Medical College, Guangdong, Shenzhen, 518116 China; 2 Department of Nutrition, Shenzhen Hospital, National Cancer Center, National Clinical Research Center for Cancer/Cancer Hospital, Chinese Academy of Medical Sciences and Peking Union Medical College, Guangdong, Shenzhen, 518116 China; 3 Department of Pharmacy, Shenzhen Hospital, National Cancer Center, National Clinical Research Center for Cancer/Cancer Hospital, Chinese Academy of Medical Sciences and Peking Union Medical College, Guangdong, Shenzhen, 518116 China

**Keywords:** BMI, ICIs, NSCLC

## Abstract

**Objectives:**

ICIs have become the standard treatment for advanced NSCLC patients. Currently, PD-L1 is the most widely useful biomarker to predict ICI efficacy, but the sensitivity and specificity are limited. Therefore, the useful predictive biomarkers of ICI efficacy is urgently needed. BMI is an internationally used measure of body health. Obesity may affect ICI efficacy by changing T cell functions. This meta-analysis aimed to clarify the relationship between BMI and survival outcomes of NSCLC patients treated with ICIs.

**Methods:**

A systematic review was conducted to identify studies that assessed the association between BMI and survival outcomes in patients treated with ICIs. OS was the primary endpoint, and PFS was the secondary endpoint. Random-effect models or fixed-effect models were utilized to combine study effects according to the Cochran Q and *I*^*2*^ tests.

**Results:**

Nine studies, including 4602 NSCLC patients treated with ICIs, that met the inclusion criteria were selected for this meta-analysis. There was no significant difference in PFS (HR 0.885; 95% CI 0.777–1.009, *p* = 0.068) or OS (HR 0.947; 95% CI 0.789–1.137, *p* = 0.560) between the low BMI group and the high BMI group. However, in the subgroup analysis, compared with normal-weight patients, overweight and obese patients achieved prolonged PFS (HR 0.862; 95% CI 0.760–0.978, *p* = 0.021) and OS (HR 0.818; 95% CI 0.741–0.902, *p*<0.0001).

**Conclusion:**

Overweight and obese NSCLC patients tend to achieve prolonged survival time with ICI regimens. Further prospective studies are needed to strengthen the association between ICI outcomes and BMI levels.

**Supplementary Information:**

The online version contains supplementary material available at 10.1186/s12885-023-11512-y.

## Introduction

Lung cancer is the leading cause of tumor-related death worldwide, and non-small cell lung cancer (NSCLC) is the most common type, accounting for approximately 80–85% of cases [1, 2]. Over the past two decades, chemotherapy has been the critical cornerstone for advanced NSCLC patients without driver oncogenes. These patients’ median overall survival (OS) was only 8–12 months [3]. The advent of immune checkpoint inhibitors (ICIs) has restructured the clinical treatment paradigm of advanced NSCLC and become the standard regimen. Several studies have shown that ICIs can significantly prolong the survival of NSCLC patients [4, 5]. Durable efficacy is an essential advantage of ICIs, but its suboptimal efficacy. Therefore, the identification of useful predictive biomarkers of ICIs is urgently needed. The most widely applied predictor is programmed death ligand-1 (PD-L1). Other predictors include tumor mutational load (TMB), microsatellite instability (MSI), and tumor-infiltrating immune cells in the tumor microenvironment. The sensitivity and specificity of these predictors are limited [6]. There are still no precise biomarkers that can accurately predict the efficacy of ICIs.

Body Mass Index (BMI) is an internationally used measure of body fatness and health. Obesity (BMI>30 kg/m^2^) is associated with many diseases such as diabetes, heart disease, and cancer [7–9]. A recent study showed that obesity may be related to immunotherapy efficacy [10]. Researchers first examined the functional differences in T cells between obese and nonobese mice. The function of T cells was weaker in obese mice than in nonobese mice, in which more PD-1 proteins were expressed. In this study, a similar phenomenon was also observed in macaques and human volunteers. Several clinical studies investigated the correlation between BMI and the efficacy of ICIs, but their findings were inconsistent. Therefore, the authors of this meta-analysis aimed to clarify the relationship between BMI and survival outcomes of NSCLC patients treated with ICIs.

## Methods

### Data sources and search strategy

This study was performed following the meta-analysis protocol for observational studies [11]. The protocol was registered with the PROSPERO International Prospective Register of Systematic Reviews (https://www.crd.york.ac.uk/prospero/) under the registration number CRD42022330046. After developing the clinical question, we manually searched medical databases, including PubMed, EMBASE, and the Cochrane Library, for studies published in English from inception until March 2022. The investigation was conducted using the PICOS format: P: NSCLC patients; I: ICI treatment; C: different BMIs; O: progression-free survival (PFS) or overall survival (OS); and S: observational studies. We used the following keywords: “non-small cell lung cancer” AND “body mass index” AND “immune checkpoint inhibitors”. We also searched the references of the included articles to identify any additional studies. We contacted the authors for the full reports of relevant unpublished studies.

Two reviewers (ZTT and LSL) independently carried out the literature search and research selection using titles and abstracts. If a potentially available article was found, the full text of the article was found and read to identify whether the study met the inclusion criteria. A team discussion was conducted to resolve discrepancies if there was any disagreement.

### Eligibility criteria

The inclusion criteria were as follows: (1) studies that involved humans; (2) studies including participants aged ≥ 18 years; (3) studies of NSCLC patients treated with ICIs (CTLA-4 or PD-1/PD-L1); and (4) studies reporting OS/PFS.

The exclusion criteria were as follows: (1) nonhuman studies (cell culture studies, animal models); (2) case reports, editorials, comments, letters, reviews, meta-analyses, or interventional studies; (3) duplicate studies; and (4) studies for which the data on clinical outcome statistical measures (hazard ratios, 95% CIs) were incomplete or for which the outcome measures could not be calculated with the available data.

### Outcome measures

The primary objective of this meta-analysis was to specify and quantify the association between BMI and the efficacy of ICIs in NSCLC patients. BMI was calculated using the formula of weight/ height^2^ (kilograms per square meter).

### Data extraction and study quality assessment

We collected the following data from eligible studies: author, publication year, country/region, age, sex, histology, smoking status, Eastern Cooperative Oncology Group performance status (ECOG PS), treatment regimen, median PFS and OS, and HRs for PFS and OS. We recorded these data in a standard data extraction spreadsheet for analysis. We only selected the most extensive and latest study to avoid duplicate patient populations. When a study reported both univariate and multivariate analyses, we selected the multivariate analysis data. We assessed the quality of observational studies by using the Newcastle–Ottawa quality assessment scale (NOS) to evaluate the following items: patient selection, the comparability of study groups, and outcome assessments. The scoring criteria were as follows: 7–9 points indicated high quality, 4–6 points indicated moderate quality, and ≤ 3 points indicated low quality. Two authors (ZTT and LSL) evaluated the bias risks independently.

### Statistical analysis

The extracted data were used in the meta-analysis using STATA 14.0 analysis software (StataCorp, College Station, TX, USA). The estimates of HR and 95% CI values were weighted and pooled. Cochran Q and *I*^*2*^ tests were used to evaluate heterogeneity between studies. When the Cochran Q *p value* was ≥ 0.10 and the *I*^*2*^ value was ≤ 50%, the fixed-effect model was used to combine the study effects. The random-effect model was used to combine the study effects when the Cochran Q p value was < 0.10 and the I2 value was > 50%. We conducted subgroup analyses for OS and PFS based on sex, histology, ECOG PS, smoking history, and PD-L1. A sensitivity analysis of the investigated outcomes was conducted sequentially, excluding each included study [12]. Funnel plot and Egger’s tests were conducted to assess publication bias of research products with more than ten studies [13]. All *p* values were two-sided, and *p* values < 0.05 were considered statistically significant.

## Results

### Literature search

**Fig. 1:**
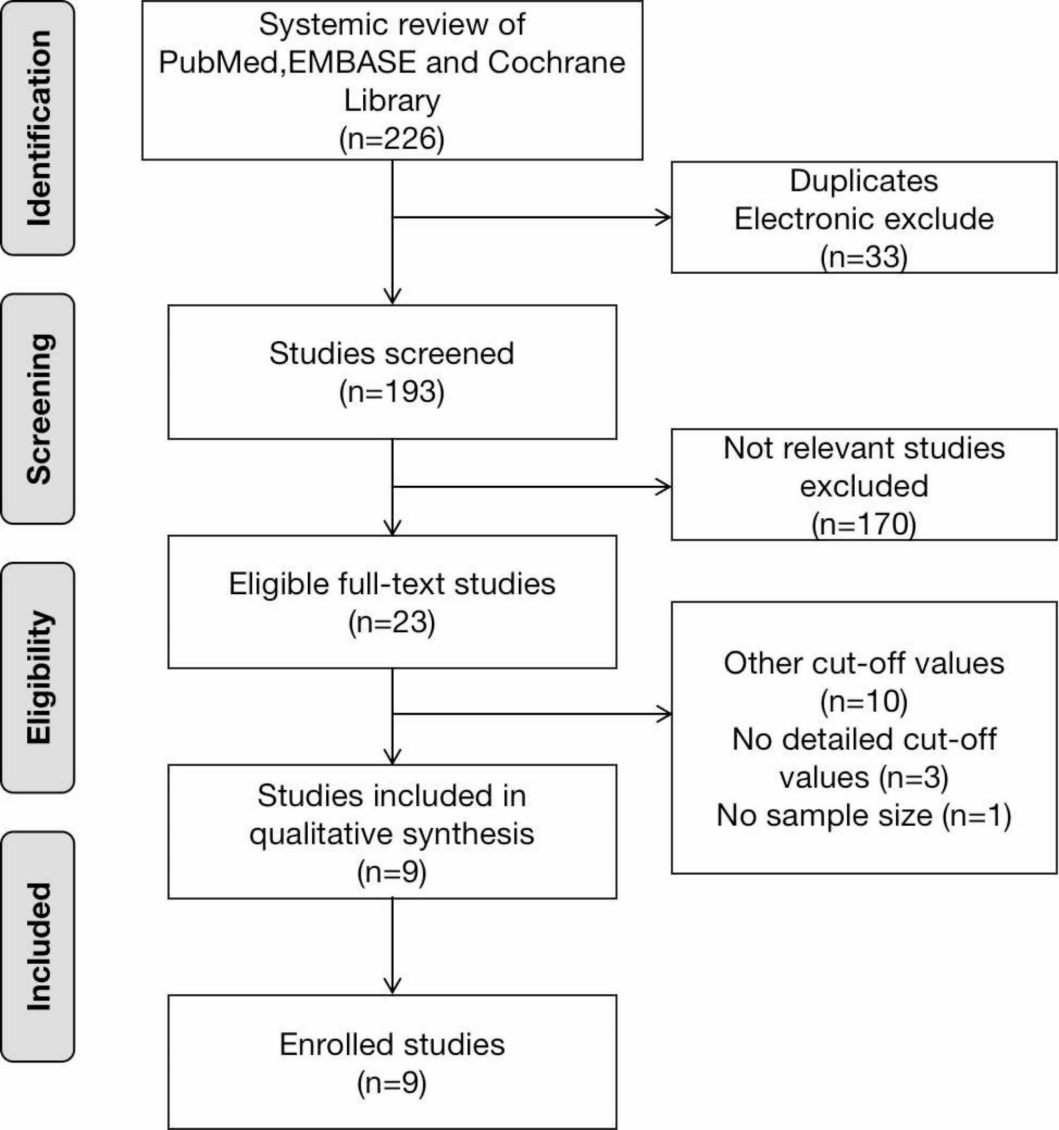
Study selection process

Figure 1 shows the study selection process. During the initial search, we discovered 226 articles. A total of 33 studies were excluded due to duplication or irrelevance. In total, 193 potentially available articles were selected. After detailed evaluations, 170 studies were excluded because they were irrelevant according to the inclusion criteria. Moreover, we excluded another 14 studies because of cut-off values or lack of sample size. Ultimately, nine studies, including 4602 NSCLC patients treated with ICIs, were selected for this meta-analysis [14–22].

### Study characteristics

Table 1 shows the general characteristics of the included studies. All studies were retrospective studies. These articles were published from 2019 to 2022. The countries/regions of the patients included in the meta-analysis included China, the USA, Japan, Canada, Australia, and other European countries. All nine studies enrolled advanced NSCLC patients with ICI treatment. The quality of the included studies was assessed using the NOS score. Four studies scored 8 points, two studies scored 7 points, and three scored 6 points. All studies are regarded as having moderate or high quality. The definition of underweight, normal, overweight and obesity are as follows: underweight is BMI < 18.5 kg/ m^2^, normal is 18.5 ≤ BMI ≤ 24.9 kg/m^2^, overweight is 25 ≤ BMI ≤ 29.9 kg/m^2^ and obese is BMI ≥ 30 kg/m^2^.

**Table 1:** Baseline characteristics and NOS score of the studies included in the meta-analysis

Study	Publication year	Country/ Region	Study design	Sample size	ICIs	BMI category	HR for PFS	HR for OS	Data derived from multivariable analyses	NOS
J. Zhou et al.	2020	China	Retrospective Single center	223	N.A.	<25 kg/m^2^ ≥ 25 kg/m^2^	0.653[0.455–0.938]	N.A.	PFS	7
S. Zhang et al.	2021	USA	Retrospective Single center	220	atezolizumab, nivolumab, pembrolizumab	<25 kg/m^2^ ≥ 25 kg/m^2^	1.32[0.962–1.818]	1.61[1.139–2.273]	PFS, OS	8
A. Tateishi et al.	2022	Japan	Retrospective Single center	324	pembrolizumab, nivolumab	<25 kg/m^2^ ≥ 25 kg/m^2^	1.00[0.41–2.50]	0.97[0.65–1.47]	PFS	8
C. Richard et al.	2019	Canada	Retrospective Multicenter	381	Anti-PD1	<25 kg/m^2^ ≥ 25 kg/m^2^	0.68[0.47–0.99]	N.A.	N.A.	6
R. Rhone et al.	2021	USA	Retrospective Multicenter	61	N.A.	Normal weight Over weight	Overweight vs. Normal 0.54[0.47–0.99]	N.A.	N.A.	6
A. Kulkarni et al.	2019	USA	Retrospective Single center	148	Anti-PD1, Anti-PD-L1	<25 kg/m^2^ ≥ 25 kg/m^2^	1.37[0.97–1.96]	1.56[1.11–2.22]	N.A.	6
G. Kichenadasse et al.	2020	Australia	Retrospective Multicenter	1434	atezolizumab	Normal weight Overweight Obese	Overweight vs. Normal 0.88[0.77–1.02] Obese vs. Normal 0.87[0.72–1.04]	Overweight vs. Normal 0.80[0.67–0.96] Obese vs. Normal 0.69[0.54–0.87]	PFS, OS	7
A. Cortellini et al.	2022	USA, Europe	Retrospective Multicenter	853	N.A.	Underweight Normal weight Overweight Obese	Overweight vs. Normal 0.83[0.68–1.01] Obese vs. Normal 1.04[0.81–1.33]	Overweight vs. Normal 0.79[0.62–1.01] Obese vs. Normal 0.99[0.74–1.32]	PFS, OS	8
A. Cortellini et al.	2020	USA, Europe	Retrospective Multicenter	958	pembrolizumab	Underweight Normal weight Overweight Obese	Overweight vs. Normal 1.04[0.85–1.26] Obese vs. Normal 0.61[0.45–0.82]	Overweight vs. Normal 0.97[0.77–1.22] Obese vs. Normal 0.70[0.49–0.99]	PFS, OS	8

Abbreviations: ICIs,Immune Checkpoint Cnhibitors; BMI,Body Mass Index; Anti-PD1,Anti-Programmed Peath 1 (PD1); Anti-PD-L1,Anti-Programmed Death-ligand 1; HR,Hazard Ratio; PFS, Progression-Free-Survival; OS, Overall-Survival; N.A.,Not applicable; NOS, Newcastle-Ottawa Scale

### Progression-free survival for all studies

**Fig. 2:**
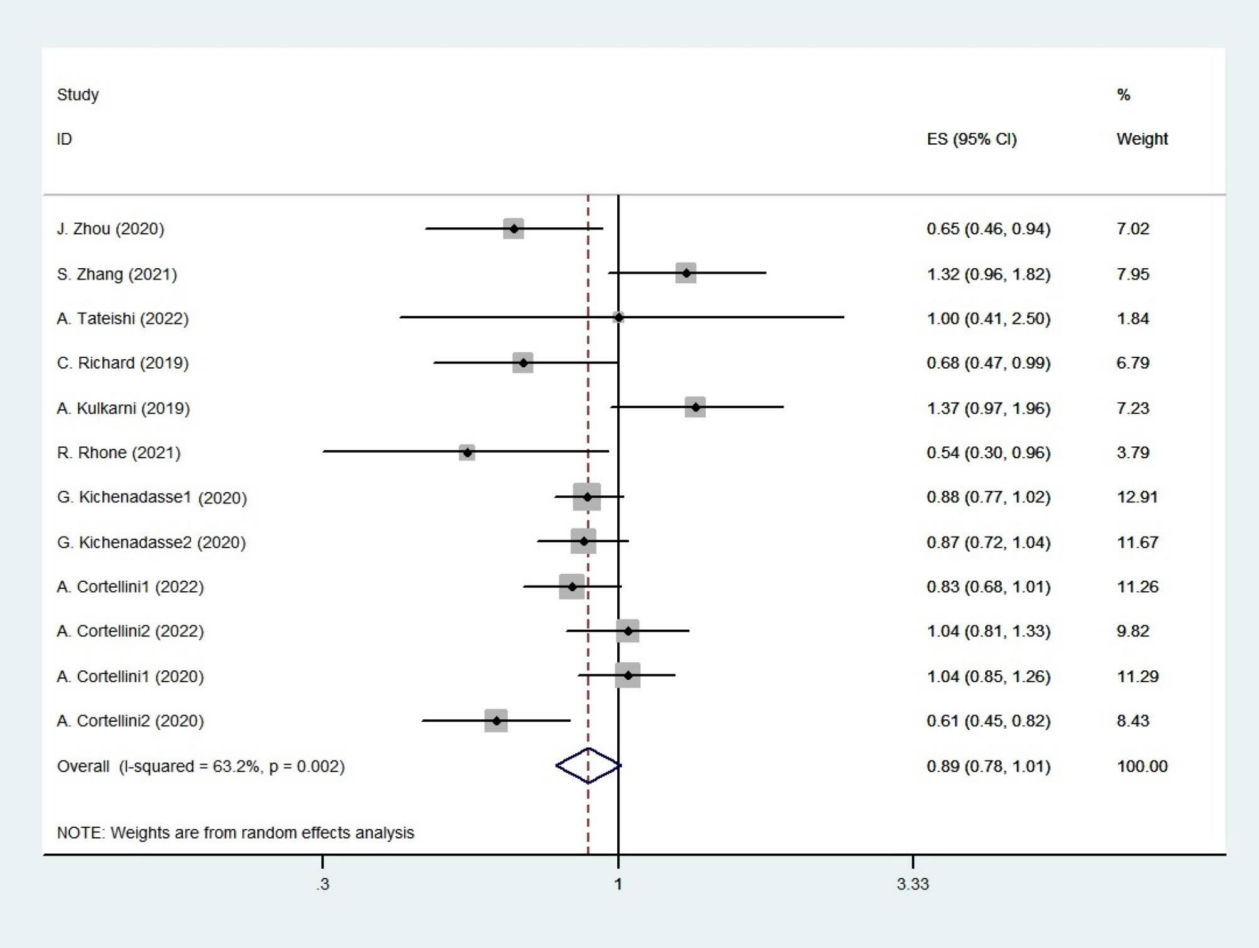
Forest plot of PFS for all studies. The diamonds and horizontal lines indicate the corresponding HRs and 95% CIs. The size of the gray area reflects the study-specific statistical weight. The vertical solid line shows the HR of 1, and the vertical red dashed line represents the combined effect estimate. The suffix “1” or “2” after the studies indicates two separate outcomes stratified by different BMI in the same study

**Fig. 3:**
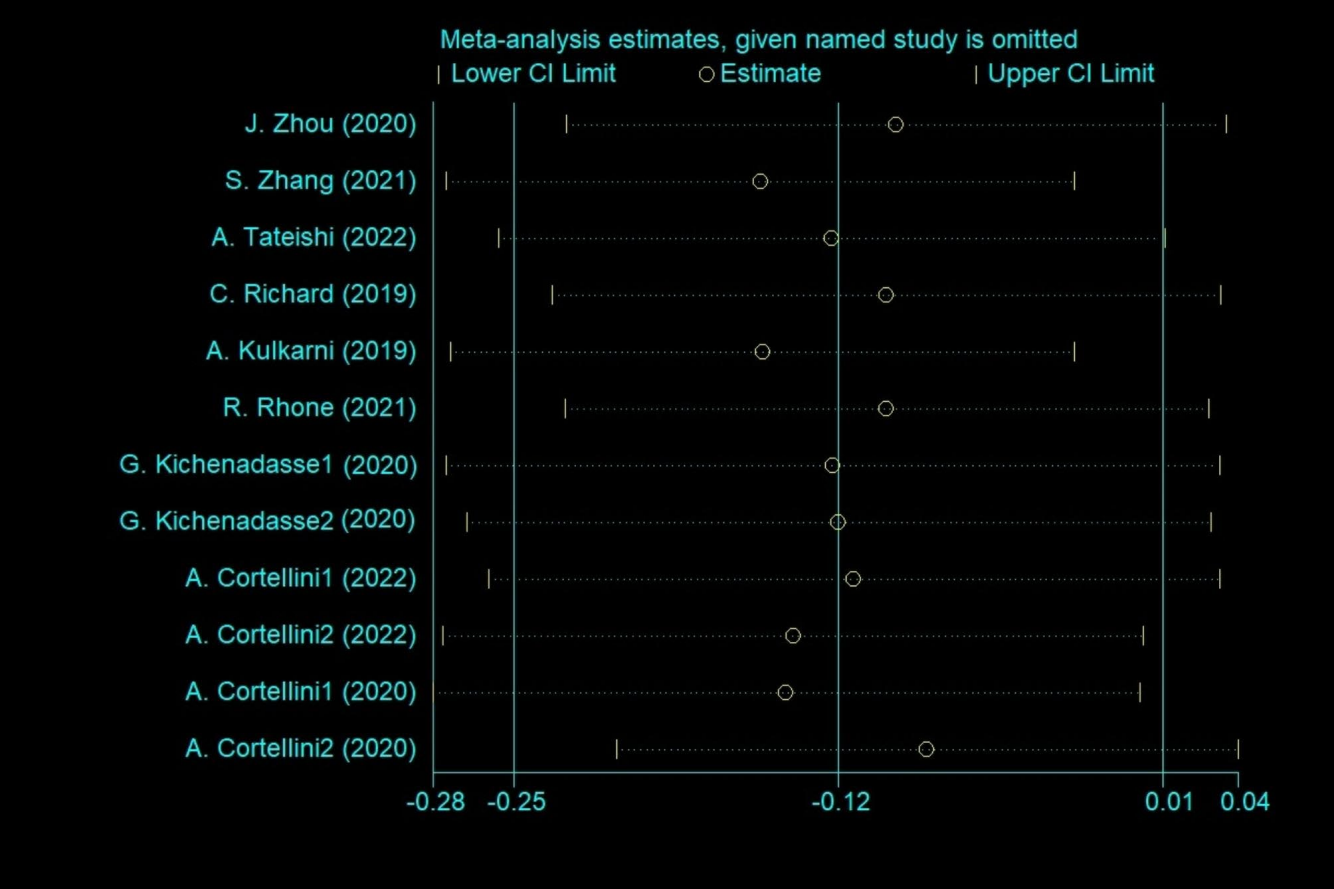
Sensitivity analyses of PFS for all studies

All nine studies reported the association between BMI and PFS [14–22]. The low BMI group included underweight patients (BMI < 18.5 kg/ m^2^) and normal patients (18.5 ≤ BMI ≤ 24.9 kg/m^2^). The high BMI group included overweight patients (25 ≤ BMI ≤ 29.9 kg/m^2^) and obese patients (BMI ≥ 30 kg/m^2^). The studies including results for overweight vs. normal-weight patients and obese vs. normal-weight patients were named Study 1 and Study 2, respectively. Pooled analysis showed no significant difference between the low BMI group and the high BMI group in PFS (HR 0.885; 95% CI 0.777–1.009, *p* = 0.068; Fig. 2) using a random-effect model (*I*^*2*^ = 63.2%; *p* = 0.002). The high BMI group tended to have long PFS. The sensitivity analyses revealed that excluding any specific study did not influence the results (Fig. 3).

### Overall survival for all studies

**Fig. 4:**
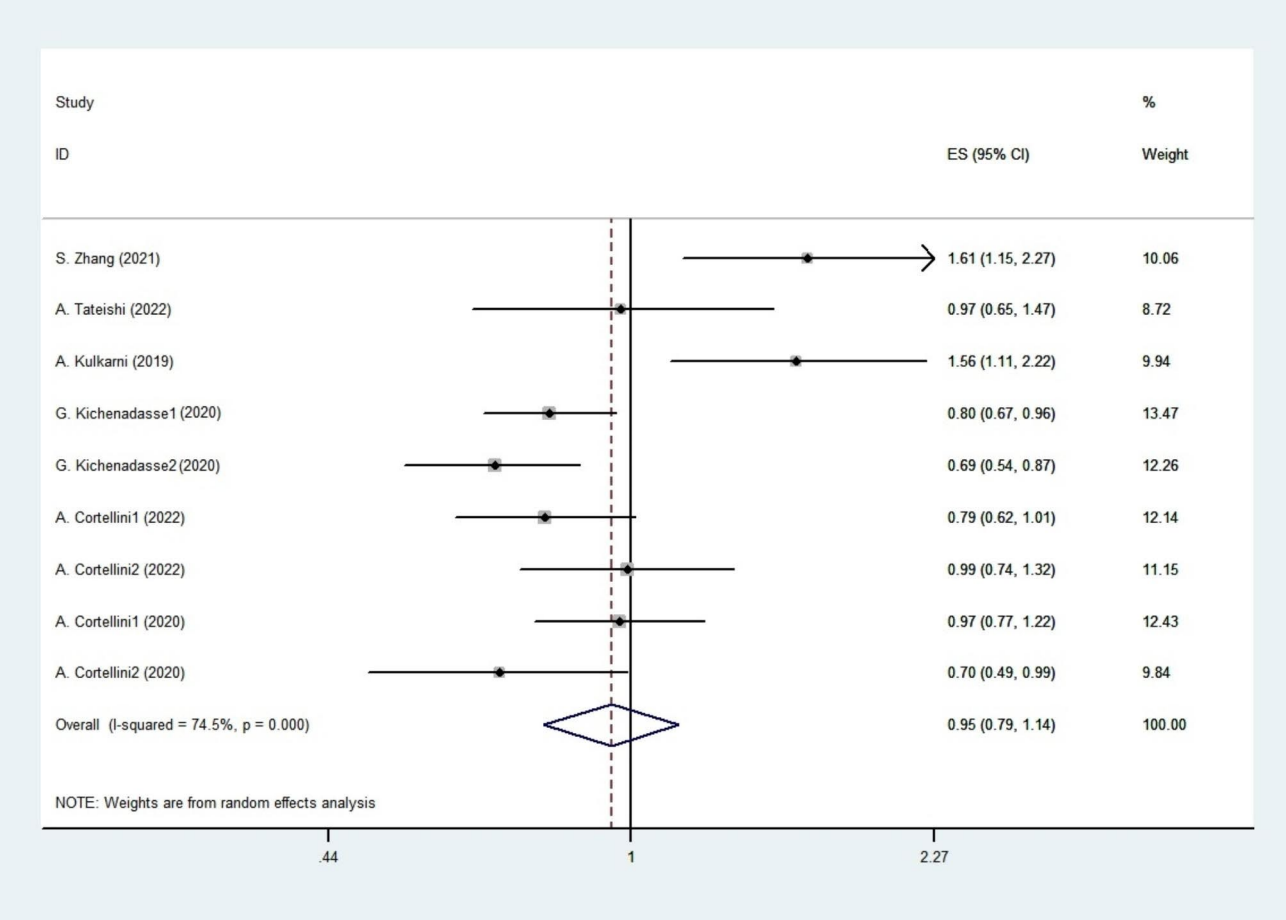
OS for all studies.The diamonds and horizontal lines indicate the corresponding HRs and 95% CIs. The size of the gray area reflects the study-specific statistical weight. The vertical solid line shows the HR of 1, and the vertical red dashed line represents the combined effect estimate. The suffix “1” or “2” after the studies indicates two separate outcomes stratified by different BMI in the same study.1 stand for overweight patient and 2 stand for obese patients

HRs for OS were reported in six studies [14–22]. The studies including results for overweight vs. normal-weight patients and obese vs. normal-weight patients were named Study 1 and Study 2, respectively. No significant difference between the low BMI group and the high BMI group was found in the analysis of OS (HR 0.947; 95% CI 0.789–1.137, *p* = 0.560; Fig. 4) by using the random-effect model (*I*^*2*^ = 74.5%; *p <* 0.001).

### Subgroup analysis

According to the various BMI cut-off values in each study, the nine studies were divided into two groups:

Group 1: Five studies chose 25 kg/m^2^ as the cut-off value. These studies did not provide more detailed BMI criteria.

Group 2: Four studies categorized BMI according to the World Health Organization (WHO) classification: underweight (BMI < 18.5 kg/ m^2^); normal (18.5 ≤ BMI ≤ 24.9 kg/m^2^); overweight (25 ≤ BMI ≤ 29.9 kg/m^2^); and obesity (BMI ≥ 30 kg/m^2^).

Part 1: PFS and OS analysis (BMI: <25 kg/m^2^ vs. ≥25 kg/m^2^).

**Fig. 5:**
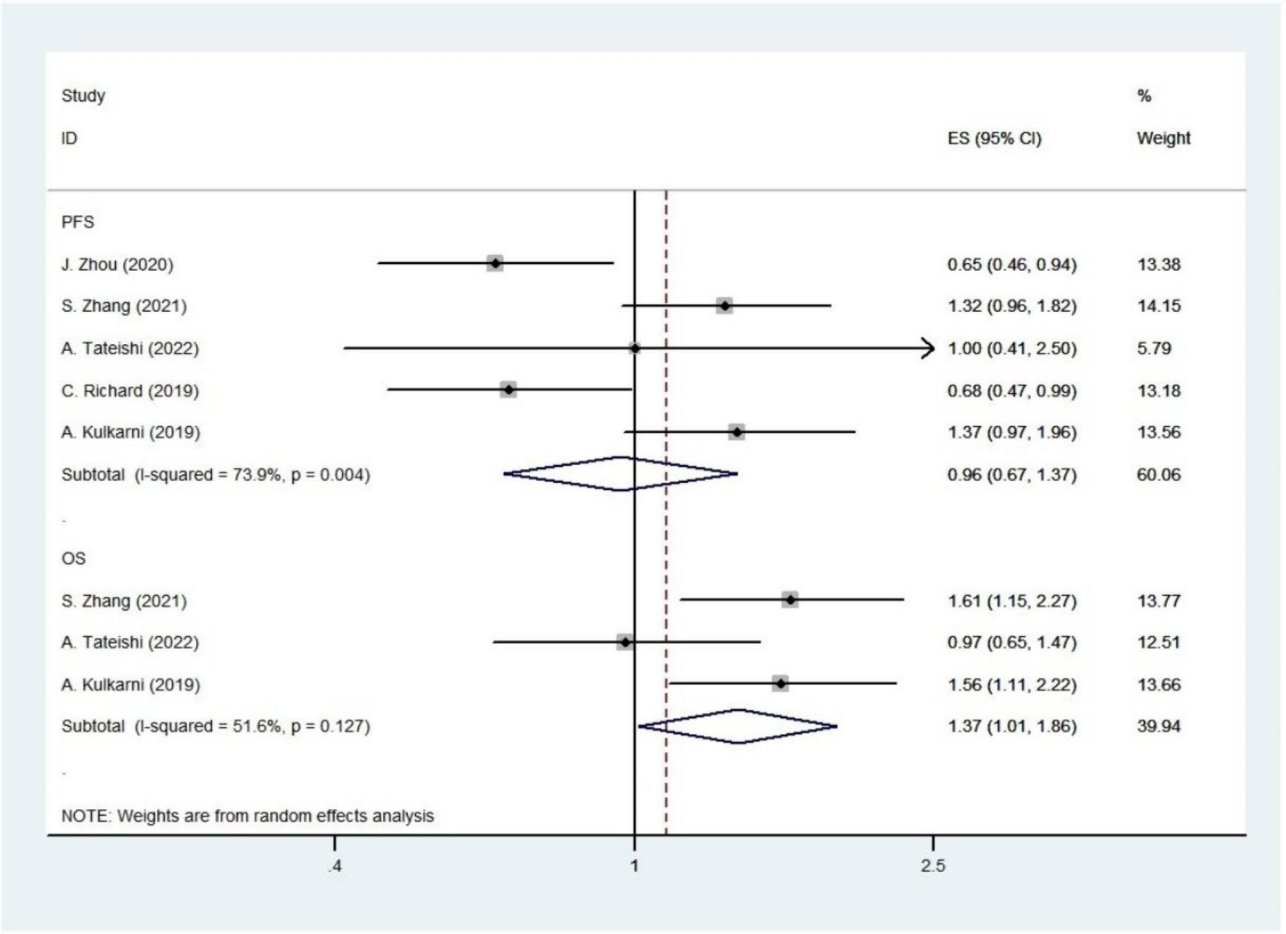
Forest plot of PFS and OS for part 1 studies.The diamonds and horizontal lines indicate the corresponding HRs and 95% CIs. The size of the gray area reflects the study-specific statistical weight. The vertical solid line shows the HR of 1, and the vertical red dashed line represents the combined effect estimate. The suffix “1” or “2” after the studies indicates two separate outcomes stratified by different BMI in the same study. 1 stand for overweight patient and 2 stand for obese patients

In PFS analysis [14–17, 19], no statistical difference was observed between the BMI < 25 kg/m^2^ group and the BMI ≥ 25 kg/m^2^ group (HR 0.958; 95% CI 0.671–1.366, *p* = 0.811; Fig. 5) by using the random-effect model (*I*^*2*^ = 73.9%; *p* = 0.004). For OS analysis [14–17, 19], the random-effect model yielded a pooled HR of 1.373 with a 95% CI of 1.015–1.856 (*p* = 0.040, Fig. 5). This result suggests a significantly prolonged OS in the BMI ≥ 25 kg/m^2^ group.

Part 2: PFS and OS analysis (overweight + obese vs. normal-weight patients).

The studies including results for overweight vs. normal-weight patients and obese vs. normal-weight patients were named Study 1 and Study 2, respectively.

**Fig. 6:**
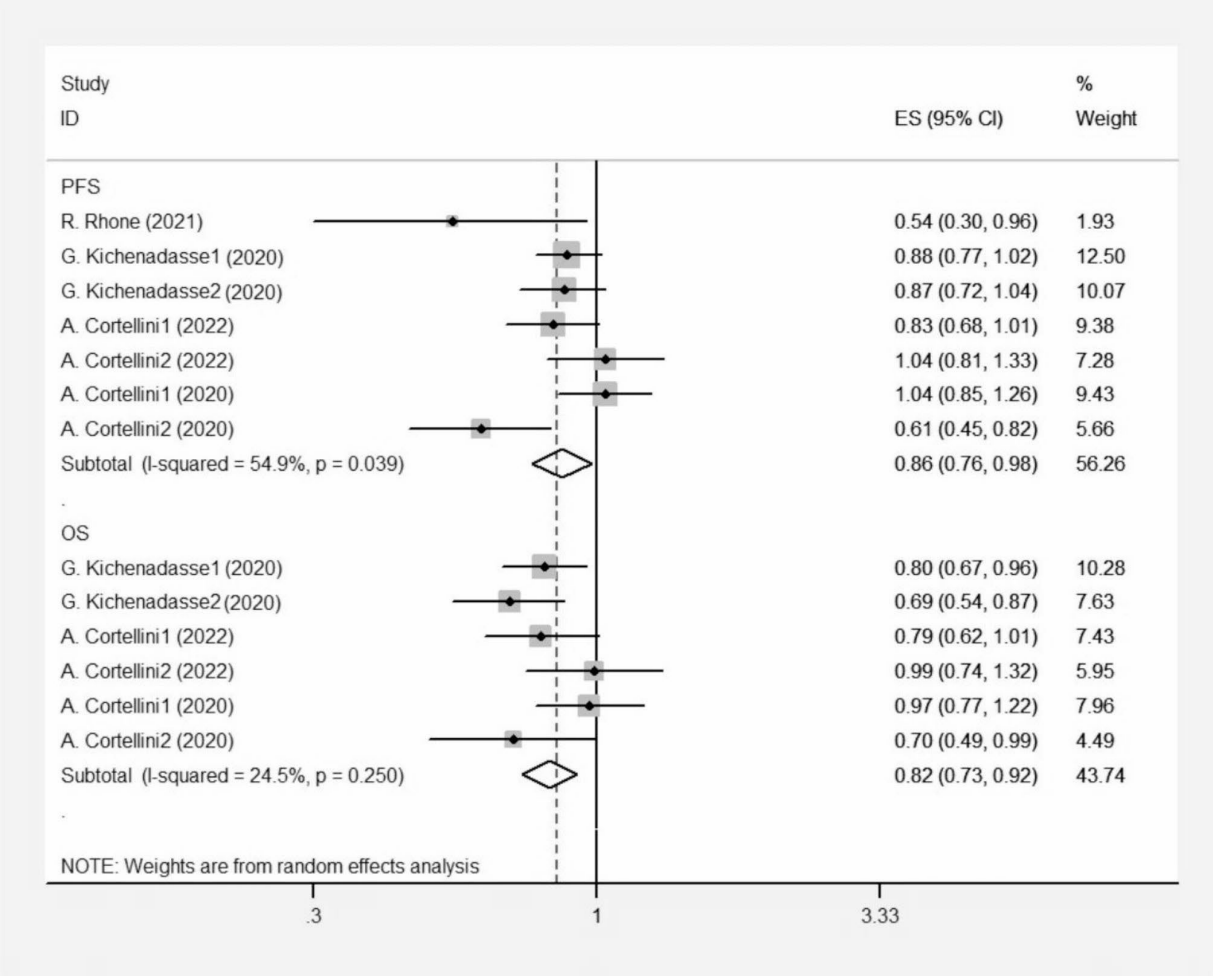
Forest plot of PFS and OS for part 2 studies.The diamonds and horizontal lines indicate the corresponding HRs and 95% CIs. The size of the gray area reflects the study-specific statistical weight. The vertical solid line shows the HR of 1, and the vertical red dashed line represents the combined effect estimate. The suffix “1” or “2” after the studies indicates two separate outcomes stratified by different BMI in the same study.1 stand for overweight patient and 2 stand for obese patients

In part 2, patients were divided into three or four groups: underweight, normal, overweight, and obese by WHO standards. All four studies reported the correlation between BMI and PFS [18, 20–22]. The summary HRs represented a prolonged PFS in the overweight or obese group compared with that in the normal-weight group (HR 0.862; 95% CI 0.760–0.978, *p* = 0.021; Fig. 6) using a random-effect model (*I*^*2*^ = 54.9%; *p* = 0.039). In part 2, the HRs for OS were given in all four studies [18, 20–22]. The fixed-effects model yielded a pooled HR of 0.818 with a 95% CI of 0.741–0.902, suggesting a significantly prolonged OS in overweight and obese patients (*p*<0.0001, Fig. 6) (*I*^*2*^ = 24.5%; *p* = 0.250).

**Fig. 7:**
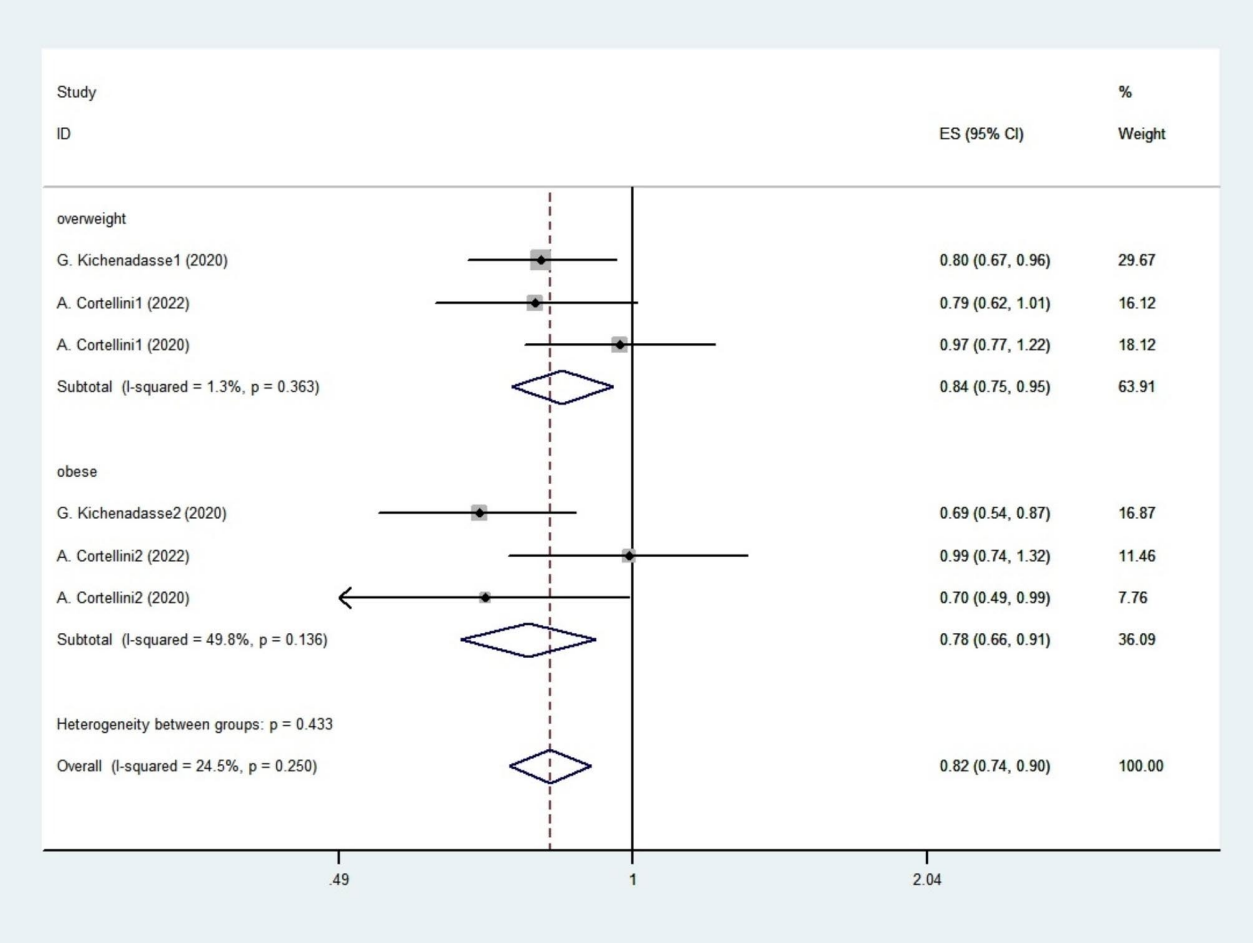
Forest plot of OS for overweight and obese patients for part 2 studies. The diamonds and horizontal lines indicate the corresponding HRs and 95% CIs. The size of the gray area reflects the study-specific statistical weight. The vertical solid line shows the HR of 1, and the vertical red dashed line represents the combined effect estimate. The suffix “1” or “2” after the studies indicates two separate outcomes stratified by different BMI in the same study.1 stand for overweight patient and 2 stand for obese patients Availability of Data and Materials

In further analysis, the OS of overweight and obese patients was significantly increased, with a HR = 0.842 (95% CI 0.745–0.952, *p* = 0.006) and a HR = 0.781 (95% CI 0.617–0.989, *p* = 0.040), respectively, by using a fixed-effect model (Fig. 7).

### Other factors for survival outcomes

We also evaluated other factors, including survival outcomes and sex, smoking history, ECOG PS, histology, and PD-L1 status (Table 2). The results revealed that smoking history, ECOG PS, and histology affected survival outcomes in NSCLC patients treated with ICIs.

**Table 2:** Other factors for survival outcomes

Factors	PFS				OS			
	HR (95%CI)	p value	Heterogeneity		HR (95%CI)	p value	Heterogeneity	
			I^2^	Q test p value			I^2^	Q test p value
Gender								
M vs. F	1.014 (0.929–1.107)	0.753	41.7%	0.127	1.041 (0.928–1.168)	0.489	0.0%	0.525
Smoking history								
Smoker vs. no	0.719 (0.645–0.802)	<0.001	49.4%	0.065	0.894 (0.771–1.037)	0.138	49.5%	0.095
ECOG								
<2 vs. ≥2	0.630 (0.482–0.822)	0.001	81.1%	<0.001	0.498 (0.372–0.667)	<0.001	81.0%	0.001
PD-L1								
1–49% vs. ≥50%	0.817 (0.599–1.114)	0.201	62.7%	0.045	2.021 (0.908–4.495)	0.085	80.9%	0.022
Histology								
Non-squa vs. squa	0.873 (0.777–0.980)	0.022	0.0%	0.412	0.822 (0.705–0.959)	0.013	17.4%	0.271

Abbreviations: PFS, Progression-Free-Survival; OS, Overall-Survival; M, male; F, Female; PD-L1, Programmed Death 1; squa, squamous; Non-squa, Non-squamous

## Discussion

Currently, PD-L1 expression status is the most commonly used biomarker to predict the efficacy of ICIs in NSCLC patients, but it has limited sensitivity and specificity [23–27]. Several studies have shown that BMI may affect the survival outcomes of patients treated with ICIs and is expected to be a potential prognostic predictor. Most studies have reported that obese patients benefited more from ICI regimens than normal-weight patients. Experimental studies have reported that obesity could induce low-grade systemic meta-inflammation and harm the immune response. Moreover, obesity also influences the immune response by causing T cell dysfunction and increasing the exhausted PD-1-positive T cell phenotype [10, 28]. ICIs maybe affect the hazard response of fat tissue. However, there is no conclusion regarding the relationship between BMI and the survival outcomes of NSCLC patients treated with ICIs and no information regarding how to choose the BMI cut-off value.

Our analysis isn’t the first meta-analysis about the relationship between BMI and ICIs, but the first meta-analysis focused on NSCLC patients to evaluate the association between BMI and ICI therapy outcomes based on observational studies. This comprehensive quantitative study included 4602 NSCLC patients from 9 studies with various characteristics. There were no differences in PFS or OS between the low BMI group and high BMI group in the primary analysis including all studies; however, in the subgroup analysis, compared with normal-weight NSCLC patients, overweight and obese NSCLC patients had prolonged PFS and OS. According to the low risk of publication bias of sensitivity analysis, we concluded that BMI level might be a prognostic factor of NSCLC patients treated with ICIs. Currently, the effect of BMI on survival is not clear. A study showed that high BMI was probably related to worse prognosis of breast cancer [29]. However, other studies have found that, compared to patients with normal BMI, patients who were underweight had a lower survival rate, while those who were obese had a higher survival rate [30, 31]. The potential mechanism of BMI in predicting drug efficacy is still unknown, however, it may be related to the tumor microenvironment, because the obese tumor microenvironment affects the drug treatment response by regulating adipose factors, angiogenesis, hypoxia, fibrosis, and immune factors. Therefore, although obesity is associated with T cell dysfunction and a worse prognosis of cancer, it also induces a better response to immunotherapy [32, 33].

However, BMI is a formula based on weight and height, it does not reflect body fat and muscle content. Sarcopenia is defined as loss of skeletal muscle mass and function [34], and regarded to be associated with increased protein degradation and decreased protein synthesis in cancer patients with involvement of Akt-mTORC1 pathway in the presence of disturbed metabolic homeostasis, malnutrition, or reduced activity [35, 36]. Several studies reported that sarcopenia is associated with treatment efficacy, quality of life and clinical outcomes in lung cancer patients [37–39]. Previous researches also showed that compared with normal patients, sarcopenia patients with ICIs had poor prognosis, revealing that sarcopenia may be a potential predictor for immunotherapy efficacy [40]. Due to the potential mechanism of sarcopenia in ICIs efficacy is still unclear, multiple studies have proposed that sarcopenia is associated with chronic inflammation which might play a central role in adverse affecting immunotherapy [41–43]. In addition, IL-15 is as a myokine expressed in skeletal muscle cells and regulates CD8 T-cell and promotes survival of T-cells [44, 45], which is important in maintaining body immune function. Previous study showed that IL-15 levels decrease in older people with sarcopenia, which suggested that sarcopenia may lead to immune function impaired [46].

Furthermore, the relationship between adipose tissue composition and survival outcomes in cancer patients is ambiguity. Mauland et al. found that high visceral fat percentage has been associated with poor outcomes in patients with endometrial cancer [47], other researches observed that high subcutaneous fat density has been associated with favorable outcomes among patients with prostate, colorectal, and renal cancer [48, 49]. Above all, the mechanism of adipose tissue composition affecting ICIs efficacy still need to be explored.

Recently, several studies revealed that obese patients might benefit from immunotherapy [30, 31]. Ziming Wang et al. [10] analyzed the effect of obesity on cancer. They proposed a possible explanation for the “paradox” phenomenon: obesity weakens the immune system. Obesity promotes tumor growth by upregulating PD-1, a “braking molecule” on immune cells, cutting the immune system and promoting tumor growth. However, overexpression of PD-1 stimulates an immune checkpoint inhibitor response. The researchers found that the obesity-induced upregulation of PD-1 expression is related to leptin [10]. Leptin, a hormone secreted by fat cells, was expressed more in overweight people/animals. In this study, leptin was demonstrated as a factor that stimulated PD-1 expression. They speculated that leptin triggered a T cell signaling pathway by upregulating PD-1 protein expression. In particular, obesity was likely a biomarker for predicting the response of patients treated with ICIs.

The result of the analysis of all nine studies was inconsistent with that of the subgroup analysis. Different criteria may be the reason for this inconsistency. Among all nine studies, five studies in Part 1 only used 25 kg/m^2^ as the cut-off value; four studies in Part 2 divided patients into three detailed groups: normal (18.5 ≤ BMI ≤ 24.9 kg/m^2^); overweight (25 ≤ BMI ≤ 29.9 kg/m^2^); and obesity (BMI ≥ 30 kg/m^2^). Therefore, the Part 1 studies enrolled patients with a BMI < 18.5 kg/ m2 in the low BMI group. However, the Part 2 studies did not include patients with a BMI < 18.5 kg/ m^2^. Most underweight patients have worse ECOG scores and are associated with shorter survival outcomes. This may explain the difference between the whole group and subgroup analysis results.

### Limitations

This study has several limitations. First, a high BMI level was not equivalent to a high body fat content. Our analysis only included BMI levels without further investigation of body muscle and fat content, which cannot fully reflect nutritional status. Second, diet status is also a critical factor in influencing immunotherapy. Another study showed that a high-fiber diet might improve ICI outcomes by affecting the gut microbiome in melanoma patients. Levels of free fatty acids, glucose, insulin, and other hormones also tend to be higher in patients with high BMI, which may be relevant factors affecting immunotherapy efficacy. Further research is needed to evaluate the role of the above factors in ICI treatment.

In conclusion, our meta-analysis showed a significant relationship between high BMI (overweight and obese) and increased OS of NSCLC patients treated with ICIs. Further prospective studies are needed to strengthen the association between ICI outcomes and BMI levels.

### Electronic supplementary material

Below is the link to the electronic supplementary material.

Supplementary Material 1

## Data Availability

All data generated or analysed during this study are included in this published article [and its supplementary information files]. Data of this article is listed in Table 1. This is a meta-analysis, all data sourced from publicly published articles.

## References

[1] Sung H, Ferlay J, Siegel RL (2021). Global Cancer statistics 2020: GLOBOCAN estimates of incidence and Mortality Worldwide for 36 cancers in 185 countries. CA Cancer J Clin.

[2] Herbst RS, Morgensztern D, Boshoff C (2018). The biology and management of non-small cell Lung cancer. Nature.

[3] Hirsch FR, Scagliotti GV, Mulshine JL (2017). Lung cancer: current therapies and new targeted treatments. Lancet.

[4] Julien Mazieres A, Rittmeyer S, Gadgeel (2021). Atezolizumab Versus Docetaxel in Pretreated patients with NSCLC: final results from the Randomized phase 2 POPLAR and phase 3 OAK clinical trials. J Thorac Oncol.

[5] Hossein Borghaei S, Gettinger, Everett E, Vokes (2021). Five-year outcomes from the Randomized, phase III trials CheckMate 017 and 057: Nivolumab Versus Docetaxel in previously treated non-small-cell Lung Cancer. J Clin Oncol.

[6] Giustini N, Bazhenova L (2021). Recognizing Prognostic and predictive biomarkers in the treatment of Non-small Cell Lung Cancer (NSCLC) with Immune Checkpoint inhibitors (ICIs). Lung Cancer (Auckl).

[7] Tao W, Lagergren J (2013). Clinical management of obese patients with cancer. Nat Rev Clin Oncol.

[8] Deng T, Lyon CJ, Bergin S (2016). Obesity, inflammation, and Cancer. Annu Rev Pathol.

[9] Hotamisligil GS (2006). Inflammation and metabolic disorders. Nature.

[10] Wang Z, Aguilar EG, Luna J et al. Paradoxical effects of obesity on T cell function during Tumor progression and PD-1 checkpoint blockade. 2019;25(1):141–51. 10.1038/s41591-018-0221-5. 10.1038/s41591-018-0221-5 PMC6324991 30420753

[11] Stroup DF, Berlin JA, Morton SC (2000). Meta- analysis of observational studies in epidemiology: a proposal for reporting. Meta-analysis of Observational studies in Epidemiology (MOOSE) Group. JAMA.

[12] Tobias A (1999). Assessing the influence of a single study in the meta-analysis estimate. Stata Tech Bull.

[13] Egger M, Davey Smith G, Schneider M (1997). Bias in meta-analysis detected by a simple, graphical test. BMJ.

[14] Zhou J, Zhou F, Chu X et al. Non-alcoholic fatty Liver Disease is associated with immune checkpoint inhibitor-based treatment response in patients with non-small cell Lung cancer with liver metastases. Transl Lung Cancer Res 2020 Vol. 9 Issue 2 Pages 316–24. 10.21037/tlcr.2020.04.15. 10.21037/tlcr.2020.04.15 PMC7225134 32420071

[15] Zhang S, Pease DF, Kulkarni AA et al. Real-World Outcomes and Clinical Predictors of Immune Checkpoint Inhibitor Monotherapy in Advanced Lung Cancer. Clin Med Insights Oncol. 2021;15:11795549211004489. 10.1177/11795549211004489. eCollection 2021. 10.1177/11795549211004489 PMC8237437 34248362

[16] Tateishi H, Horinouchi T, Yoshida (2022). Correlation between body mass index and efficacy of anti-PD-1 inhibitor in patients with non-small cell Lung cancer. Respir Investig.

[17] Richard A, Elkrief J, Malo, et al. Effect of body mass index and age on survival in patients with advanced Lung cancer treated with anti-PD-1 immune checkpoint inhibitors. J Clin Oncol. 2019;37. 10.1200/JCO.2019.37.15_suppl.e20676.

[18] Rhone R, Dumais K, Powery HW et al. Clinical predictive markers of response to immune checkpoint inhibitor therapy in advanced nonsmall cell lung cancer. Journal of Clinical Oncology. 2021; 39(15) SUPPL. 10.1200/JCO.2021.39.15_suppl.e21044.

[19] Kulkarni S, Zhang T, De For (2019). MA07.09 impact of body Mass Index on Clinical outcomes of Immune Checkpoint blockers in Advanced Non-small Cell Lung Cancer. J Thorac Oncol.

[20] Kichenadasse G, Miners JO, Mangoni AA (2020). Association between Body Mass Index and overall survival with Immune checkpoint inhibitor therapy for Advanced Non-small Cell Lung Cancer. JAMA Oncol.

[21] Cortellini B, Ricciuti VR, Vaz, et al. Prognostic effect of body mass index in patients with advanced NSCLC treated with chemoimmunotherapy combinations. J Immunother Cancer. 2022;10(2). 10.1136/jitc-2021-004374. 10.1136/jitc-2021-004374 PMC8852707 35173031

[22] Cortellini B, Ricciuti M, Tiseo (2020). Baseline BMI and BMI variation during first line pembrolizumab in NSCLC patients with a PD-L1 expression ≥ 50%: a multicenter study with external validation. J Immunother Cancer.

[23] Herbst RS, Baas P, Kim DW (2016). Pembrolizumab versus Docetaxel for previously treated, PD-L1-positive, advanced non-small-cell Lung cancer (KEYNOTE-010): a randomised controlled trial. Lancet.

[24] Borghaei H, Paz-Ares L, Horn L (2015). Nivolumab versus Docetaxel in advanced nonsquamous non-small-cell Lung cancer. N Eng J Med.

[25] Topalian SL, Taube JM, Anders RA (2016). Mechanism-driven biomarkers to guide immune checkpoint blockade in cancer therapy. Nat Rev Cancer.

[26] Tumeh PC, Harview CL, Yearley JH (2014). PD-1 blockade induces responses by inhibiting adaptive immune resistance. Nature.

[27] Herbst RS, Soria JC, Kowanetz M (2014). Predictive correlates of response to the anti-PD-L1 antibody MPDL3280A in cancer patients. Nature.

[28] Murphy KA, James BR, Sjaastad FV (2018). Cutting edge: elevated leptin during diet-induced obesity reduces the efficacy of Tumor immunotherapy. J Immunol.

[29] Liu YS, Wu PE, Chou WC (2021). Body mass index and type 2 Diabetes and Breast cancer survival: a mendelian randomization study. Am J Cancer Res.

[30] McQuade JL, Daniel CR, Hess KR (2018). Association of body-mass index and outcomes in patients with metastatic Melanoma treated with targeted therapy, immunotherapy, or chemotherapy: a retrospective, multicohort analysis. Lancet Oncol.

[31] Laurence Albiges 1, Hakimi AA 1, Xie W, et al. Body mass index and metastatic renal cell carcinoma: clinical and biological correlations. J Clin Oncol. 2016;34(30):3655–63. 10.1200/JCO.2016.66.7311. 10.1200/JCO.2016.66.7311 PMC5065111 27601543

[32] Bai M, Wang W, Gao X (2022). Efficacy of Immune checkpoint inhibitors in patients with EGFR Mutated NSCLC and potential risk factors Associated with prognosis: a single Institution experience. Front Immunol.

[33] Assumpção JAF, Pasquarelli-do-Nascimento G, Duarte MSV (2022). The ambiguous role of obesity in oncology by promoting cancer but boosting antitumor immunotherapy. J Biomed Sci.

[34] Fielding RA, Vellas B, Evans WJ (2011). Sarcopenia: an undiagnosed condition in older adults. Current consensus definition: prevalence, etiology, and consequences. International working group on Sarcopenia. J Am Med Dir Assoc.

[35] Geremia A, Sartori R, Baraldo M (2022). Activation of Akt-mTORC1 signalling reverts cancer-dependent muscle wasting. J Cachexia Sarcopenia Muscle.

[36] Meza-Valderrama D, Marco E, Dávalos-Yerovi V (2021). Sarcopenia, Malnutrition, and cachexia: adapting definitions and terminology of Nutritional Disorders in older people with cancer. Nutrients.

[37] Nipp RD, Fuchs G, El-Jawahri A (2018). Sarcopenia is associated with quality of life and depression in patients with advanced cancer. Oncologist.

[38] Chen X, Hou L, Shen Y (2021). The role of baseline Sarcopenia index in predicting chemotherapy-induced undesirable effects and mortality in older people with stage III or IV non-small cell Lung cancer. J Nutr Health Aging.

[39] Takahashi Y, Suzuki S, Hamada K (2021). Sarcopenia is poor risk for unfavorable short- and long-term outcomes in stage I non-small cell Lung cancer. Ann Transl Med.

[40] Li S, Wang T, Tong G (2021). Prognostic impact of Sarcopenia on clinical outcomes in malignancies treated with immune checkpoint inhibitors: a systematic review and meta-analysis. Front Oncol.

[41] Nishioka N, Uchino J, Hirai S (2019). Association of Sarcopenia with and efficacy of anti-PD-1/PD-L1 therapy in non-small-cell Lung cancer. J Clin Med.

[42] Wang Y, Chen P, Huang J (2021). Assessment of Sarcopenia as a predictor of poor overall survival for advanced non-small-cell Lung cancer patients receiving salvage anti-PD-1 immunotherapy. Ann Transl Med.

[43] Tenuta M, Gelibter A, Pandozzi C (2021). Impact of Sarcopenia and inflammation on patients with advanced nonsmall cell Lung cancer (NCSCL) treated with immune checkpoint inhibitors (ICIs): a prospective study. Cancers (Basel).

[44] Crane JD, MacNeil LG, Lally JS (2015). Exercise-stimulated interleukin-15 is controlled by AMPK and regulates skin metabolism and aging. Aging Cell.

[45] Conlon KC, Lugli E, Welles HC (2015). Redistribution, hyperproliferation, activation of natural killer cells and CD8 T cells, and cytokine production during first-in-human clinical trial of recombinant human interleukin-15 in patients with cancer. J Clin Oncol.

[46] Duggal NA, Pollock RD, Lazarus NR (2018). Major features of immunesenescence, including reduced thymic output, are ameliorated by high levels of physical activity in adulthood. Aging Cell.

[47] Mauland KK, Eng Ø, Ytre-Hauge S et al. High visceral fat percentage is associated with poor outcome in endometrial cancer. Oncotarget. 2017; 8(62):105184–105195. 10.18632/oncotarget PMID: 29285243. 10.18632/oncotarget.21917 PMC5739630 29285243

[48] Antoun S, Bayar A, Ileana E et al. High subcutaneous adipose tissue predicts the prognosis in metastatic castration-resistant prostate cancer patients in post chemotherapy setting. Eur J Cancer. 2015; 51(17):2570–2577. 10.1016/j.ejca.2015.07.042 PMID: 26278649. 10.1016/j.ejca.2015.07.042 26278649

[49] Ebadi M, Martin L, Ghosh S et al. Subcutaneous adiposity is an independent predictor of mortality in cancer patients. British Journal of Cancer. 2017; 117(1):148–155. 10.1038/bjc.2017.149 PMID: 28588319. 10.1038/bjc.2017.149 PMC5520211 28588319

