# Supplementary material for: Impact of BMI on the survival outcomes of non-small cell lung cancer patients treated with immune checkpoint inhibitors: a meta-analysis
Source: BMC Cancer. 2023 Oct 23;23:1023. doi: 10.1186/s12885-023-11512-y (PMC10594865; doi:10.1186/s12885-023-11512-y)
Supplement: Supplementary file 1 — Supplementary Material 1 [file 12885_2023_11512_MOESM1_ESM.docx]

**Appendix**

***Search terms for the systematic review in detail***

We launched the following query in Pubmed and replicated it in Embase and Cochorane database: ((((((((Index, Body Mass) OR (Quetelet Index)) OR (Index, Quetelet)) OR (Quetelet's Index)) OR (Quetelets Index)) OR (Body Mass Index)) OR (BMI)) AND (((((((((((((((((((((((((((((((((Checkpoint Inhibitors, Immune) OR (Immune Checkpoint Inhibitor)) OR (Checkpoint Inhibitor, Immune)) OR (Immune Checkpoint Blockers)) OR (Checkpoint Blockers, Immune)) OR (Immune Checkpoint Blockade)) OR (Checkpoint Blockade, Immune)) OR (Immune Checkpoint Inhibition)) OR (Checkpoint Inhibition, Immune)) OR (PD-L1 Inhibitors)) OR (PD L1 Inhibitors)) OR (PD-L1 Inhibitor)) OR (PD L1 Inhibitor)) OR (Programmed Death-Ligand 1 Inhibitors)) OR (Programmed Death Ligand 1 Inhibitors)) OR (PD-1-PD-L1 Blockade)) OR (Blockade, PD-1-PD-L1)) OR (PD 1 PD L1 Blockade)) OR (CTLA-4 Inhibitors)) OR (CTLA 4 Inhibitors)) OR (CTLA-4 Inhibitor)) OR (CTLA 4 Inhibitor)) OR (Cytotoxic T-Lymphocyte-Associated Protein 4 Inhibitors)) OR (Cytotoxic T Lymphocyte Associated Protein 4 Inhibitors)) OR (Cytotoxic T-Lymphocyte-Associated Protein 4 Inhibitor)) OR (Cytotoxic T Lymphocyte Associated Protein 4 Inhibitor)) OR (PD-1 Inhibitors)) OR (PD 1 Inhibitors)) OR (PD-1 Inhibitor)) OR (Inhibitor, PD-1)) OR (PD 1 Inhibitor)) OR (Programmed Cell Death Protein 1 Inhibitor)) OR (Programmed Cell Death Protein 1 Inhibitors))) AND (((((((((((((((((Pulmonary Neoplasms) OR (Neoplasms, Lung)) OR (Lung Neoplasm)) OR (Neoplasm, Lung)) OR (Neoplasms, Pulmonary)) OR (Neoplasm, Pulmonary)) OR (Pulmonary Neoplasm)) OR (Lung Cancer)) OR (Cancer, Lung)) OR (Cancers, Lung)) OR (Lung Cancers)) OR (Pulmonary Cancer)) OR (Cancer, Pulmonary)) OR (Cancers, Pulmonary)) OR (Pulmonary Cancers)) OR (Cancer of the Lung)) OR (Cancer of Lung))

**Supplementary 1. Search results of Cochorane database**

Record #1 of 32

Provider: John Wiley & Sons, Ltd

Content: text/plain; charset="UTF-8"

TY - JOUR

AN - CD008965

AU - Jefferson, T

AU - Jones, MA

AU - Doshi, P

AU - Del Mar, CB

AU - Hama, R

AU - Thompson, MJ

AU - Spencer, EA

AU - Onakpoya, IJ

AU - Mahtani, KR

AU - Nunan, D

AU - et al.

TI - Neuraminidase inhibitors for preventing and treating influenza in adults and children

JF - Cochrane Database of Systematic Reviews

PY - 2014

IS - 4

PB - John Wiley & Sons, Ltd

SN - 1465-1858

KW - Adult

KW - Antiviral Agents [adverse effects, *therapeutic use]

KW - Child

KW - Drug Evaluation

KW - Enzyme Inhibitors [adverse effects, *therapeutic use]

KW - Europe

KW - Health Status

KW - Humans

KW - Influenza, Human [*drug therapy, *prevention & control]

KW - Japan

KW - Legislation, Drug

KW - Neuraminidase [*antagonists & inhibitors]

KW - Oseltamivir [adverse effects, *therapeutic use]

KW - Pneumonia [prevention & control]

KW - Publication Bias

KW - Randomized Controlled Trials as Topic

KW - United Kingdom

KW - United States

KW - Zanamivir [adverse effects, *therapeutic use]

N1 - [Acute Respiratory Infections]

DO - 10.1002/14651858.CD008965.pub4

AB - Abstract - Background Neuraminidase inhibitors (NIs) are stockpiled and recommended by public health agencies for treating and preventing seasonal and pandemic influenza. They are used clinically worldwide. Objectives To describe the potential benefits and harms of NIs for influenza in all age groups by reviewing all clinical study reports of published and unpublished randomised, placebo‐controlled trials and regulatory comments. Search methods We searched trial registries, electronic databases (to 22 July 2013) and regulatory archives, and corresponded with manufacturers to identify all trials. We also requested clinical study reports. We focused on the primary data sources of manufacturers but we checked that there were no published randomised controlled trials (RCTs) from non‐manufacturer sources by running electronic searches in the following databases: the Cochrane Central Register of Controlled Trials (CENTRAL), MEDLINE, MEDLINE (Ovid), EMBASE, Embase.com, PubMed (not MEDLINE), the Database of Reviews of Effects, the NHS Economic Evaluation Database and the Health Economic Evaluations Database. Selection criteria Randomised, placebo‐controlled trials on adults and children with confirmed or suspected exposure to naturally occurring influenza. Data collection and analysis We extracted clinical study reports and assessed risk of bias using purpose‐built instruments. We analysed the effects of zanamivir and oseltamivir on time to first alleviation of symptoms, influenza outcomes, complications, hospitalisations and adverse events in the intention‐to‐treat (ITT) population. All trials were sponsored by the manufacturers. Main results We obtained 107 clinical study reports from the European Medicines Agency (EMA), GlaxoSmithKline and Roche. We accessed comments by the US Food and Drug Administration (FDA), EMA and Japanese regulator. We included 53 trials in Stage 1 (a judgement of appropriate study design) and 46 in Stage 2 (formal analysis), including 20 oseltamivir (9623 participants) and 26 zanamivir trials (14,628 participants). Inadequate reporting put most of the zanamivir studies and half of the oseltamivir studies at a high risk of selection bias. There were inadequate measures in place to protect 11 studies of oseltamivir from performance bias due to non‐identical presentation of placebo. Attrition bias was high across the oseltamivir studies and there was also evidence of selective reporting for both the zanamivir and oseltamivir studies. The placebo interventions in both sets of trials may have contained active substances. Time to first symptom alleviation . For the treatment of adults, oseltamivir reduced the time to first alleviation of symptoms by 16.8 hours (95% confidence interval (CI) 8.4 to 25.1 hours, P < 0.0001). This represents a reduction in the time to first alleviation of symptoms from 7 to 6.3 days. There was no effect in asthmatic children, but in otherwise healthy children there was (reduction by a mean difference of 29 hours, 95% CI 12 to 47 hours, P = 0.001). Zanamivir reduced the time to first alleviation of symptoms in adults by 0.60 days (95% CI 0.39 to 0.81 days, P < 0.00001), equating to a reduction in the mean duration of symptoms from 6.6 to 6.0 days. The effect in children was not significant. In subgroup analysis we found no evidence of a difference in treatment effect for zanamivir on time to first alleviation of symptoms in adults in the influenza‐infected and non‐influenza‐infected subgroups (P = 0.53). Hospitalisations . Treatment of adults with oseltamivir had no significant effect on hospitalisations: risk difference (RD) 0.15% (95% CI ‐0.78 to 0.91). There was also no significant effect in children or in prophylaxis. Zanamivir hospitalisation data were unreported. Serious influenza complications or those leading to study withdrawal . In adult treatment trials, oseltamivir did not significantly reduce those complications classified as serious or those which led to study withdrawal (RD 0.07%, 95% CI ‐0.78 to 0.44), nor in child treatment trials; neither did zanamivir in the treatment of adults or in prophylaxis. There were insufficient events to compare this outcome for oseltamivir in prophylaxis or zanamivir in the treatment of children. Pneumonia . Oseltamivir significantly reduced self reported, investigator‐mediated, unverified pneumonia (RD 1.00%, 95% CI 0.22 to 1.49); number needed to treat to benefit (NNTB) = 100 (95% CI 67 to 451) in the treated population. The effect was not significant in the five trials that used a more detailed diagnostic form for pneumonia. There were no definitions of pneumonia (or other complications) in any trial. No oseltamivir treatment studies reported effects on radiologically confirmed pneumonia. There was no significant effect on unverified pneumonia in children. There was no significant effect of zanamivir on either self reported or radiologically confirmed pneumonia. In prophylaxis, zanamivir significantly reduced the risk of self reported, investigator‐mediated, unverified pneumonia in adults (RD 0.32%, 95% CI 0.09 to 0.41); NNTB = 311 (95% CI 244 to 1086), but not oseltamivir. Bronchitis, sinusitis and otitis media . Zanamivir significantly reduced the risk of bronchitis in adult treatment trials (RD 1.80%, 95% CI 0.65 to 2.80); NNTB = 56 (36 to 155), but not oseltamivir. Neither NI significantly reduced the risk of otitis media and sinusitis in both adults and children. Harms of treatment . Oseltamivir in the treatment of adults increased the risk of nausea (RD 3.66%, 95% CI 0.90 to 7.39); number needed to treat to harm (NNTH) = 28 (95% CI 14 to 112) and vomiting (RD 4.56%, 95% CI 2.39 to 7.58); NNTH = 22 (14 to 42). The proportion of participants with four‐fold increases in antibody titre was significantly lower in the treated group compared to the control group (RR 0.92, 95% CI 0.86 to 0.97, I 2 statistic = 0%) (5% absolute difference between arms). Oseltamivir significantly decreased the risk of diarrhoea (RD 2.33%, 95% CI 0.14 to 3.81); NNTB = 43 (95% CI 27 to 709) and cardiac events (RD 0.68%, 95% CI 0.04 to 1.0); NNTB = 148 (101 to 2509) compared to placebo during the on‐treatment period. There was a dose‐response effect on psychiatric events in the two oseltamivir "pivotal" treatment trials, WV15670 and WV15671, at 150 mg (standard dose) and 300 mg daily (high dose) (P = 0.038). In the treatment of children, oseltamivir induced vomiting (RD 5.34%, 95% CI 1.75 to 10.29); NNTH = 19 (95% CI 10 to 57). There was a significantly lower proportion of children on oseltamivir with a four‐fold increase in antibodies (RR 0.90, 95% CI 0.80 to 1.00, I 2 = 0%). Prophylaxis . In prophylaxis trials, oseltamivir and zanamivir reduced the risk of symptomatic influenza in individuals (oseltamivir: RD 3.05% (95% CI 1.83 to 3.88); NNTB = 33 (26 to 55); zanamivir: RD 1.98% (95% CI 0.98 to 2.54); NNTB = 51 (40 to 103)) and in households (oseltamivir: RD 13.6% (95% CI 9.52 to 15.47); NNTB = 7 (6 to 11); zanamivir: RD 14.84% (95% CI 12.18 to 16.55); NNTB = 7 (7 to 9)). There was no significant effect on asymptomatic influenza (oseltamivir: RR 1.14 (95% CI 0.39 to 3.33); zanamivir: RR 0.97 (95% CI 0.76 to 1.24)). Non‐influenza, influenza‐like illness could not be assessed due to data not being fully reported. In oseltamivir prophylaxis studies, psychiatric adverse events were increased in the combined on‐ and off‐treatment periods (RD 1.06%, 95% CI 0.07 to 2.76); NNTH = 94 (95% CI 36 to 1538) in the study treatment population. Oseltamivir increased the risk of headaches whilst on treatment (RD 3.15%, 95% CI 0.88 to 5.78); NNTH = 32 (95% CI 18 to 115), renal events whilst on treatment (RD 0.67%, 95% CI ‐2.93 to 0.01); NNTH = 150 (NNTH 35 to NNTB > 1000) and nausea whilst on treatment (RD 4.15%, 95% CI 0.86 to 9.51); NNTH = 25 (95% CI 11 to 116). Authors' conclusions Oseltamivir and zanamivir have small, non‐specific effects on reducing the time to alleviation of influenza symptoms in adults, but not in asthmatic children. Using either drug as prophylaxis reduces the risk of developing symptomatic influenza. Treatment trials with oseltamivir or zanamivir do not settle the question of whether the complications of influenza (such as pneumonia) are reduced, because of a lack of diagnostic definitions. The use of oseltamivir increases the risk of adverse effects, such as nausea, vomiting, psychiatric effects and renal events in adults and vomiting in children. The lower bioavailability may explain the lower toxicity of zanamivir compared to oseltamivir. The balance between benefits and harms should be considered when making decisions about use of both NIs for either the prophylaxis or treatment of influenza. The influenza virus‐specific mechanism of action proposed by the producers does not fit the clinical evidence. Plain language summary Regulatory information on trials of oseltamivir (Tamiflu) and zanamivir (Relenza) for influenza in adults and children Oseltamivir and zanamivir have been stockpiled in many countries to treat and prevent seasonal and pandemic influenza, before an influenza vaccine matched to the circulating virus becomes available. Oseltamivir is classified by the World Health Organization as an essential medicine. How this review has been approached We have updated and combined our reviews on the antiviral drugs zanamivir and oseltamivir for influenza in adults and children on the basis of the manufacturers' reports to regulators (clinical study reports) and the regulators' comments. We have called these comments and reports 'regulatory information'. Clinical study reports are unpublished, extensive documents with great detail on the trials that formed the basis for market approval. They include the protocols, methods and results. Clinical study reports have until now been confidential, seen only by the manufacturers and regulators. Why we have taken this approach In previous versions of this review we identified unresolved discrepancies in the data presented in published trial reports and substantial publication bias. As a consequence, we elected not to use data from journal articles but included the documents generated during licensing processes. We have accessed such data from the UK, USA, European Medicines Agency (EMA), Japanese regulators and clinical study reports from the manufacturers (after a protracted media campaign). This has enabled us to verify information from the randomised, placebo‐controlled trials on adults and children with confirmed or suspected exposure to naturally occurring influenza. Based on our assessments of the regulatory documents (in excess of 160,000 pages), we came to the conclusion that there were substantial problems with the design, conduct, reporting and availability of information from many of the trials. What we have found We have used data from 46 trials (20 oseltamivir and 26 zanamivir studies) in this review. We identified problems in the design of many of the studies that we included, which affects our confidence in their results. We found that both drugs shorten the duration of symptoms of influenza‐like illness (unconfirmed influenza or 'the flu') by less than a day. Oseltamivir did not affect the number of hospitalisations, based on the data from all the people enrolled in treatment trials of oseltamivir. Zanamivir trials did not record this outcome. The effects on pneumonia and other complications of influenza, such as bronchitis, middle ear infection (otitis media) and sinusitis, were unreliably reported, as shown by the case report form in the trial documents. Some forms showed limitations in the diagnostic criteria for pneumonia. Regulatory comments noted problems with missing follow‐up diary cards from participants. In children with asthma there was no clear effect on the time to first alleviation of symptoms. Prophylaxis trials showed that oseltamivir and zanamivir reduced the risk of symptomatic influenza in individuals and households. There was no evidence of an effect on asymptomatic influenza or on non‐influenza, influenza‐like illness, but trial conduct problems prevent any definitive conclusion. Oseltamivir use was associated with nausea, vomiting, headaches, renal and psychiatric events; these last three were when it was used to prevent influenza (prophylaxis). Its effect on the heart is unclear: it may reduce cardiac symptoms, but may induce serious heart rhythm problems. In adult treatment trials of zanamivir there was no increased risk of reported adverse events. The evidence on the possible harms associated with the treatment of children with zanamivir was sparse. Agreement with other findings The lack of good evidence demonstrating an effect on complications agrees with the conservative conclusions on both drugs drawn by the US Food and Drug Administration (FDA). The FDA only allowed claims of effectiveness of both drugs for the prevention and treatment of symptoms of influenza and not for other effects (including the interruption of person‐to‐person spread of the influenza virus or prevention of pneumonia). The FDA described the overall performance of both drugs as 'modest'. Mechanism of action for beneficial effects These findings all suggest that the low immune response with low levels of pro‐inflammatory cytokines, which is induced by the action of oseltamivir carboxylate, may reduce the symptoms of influenza unrelated to an inhibition of influenza virus replication. The potential hypothermic or antipyretic effect of oseltamivir as a central nervous system depressant may also contribute to the apparent reduction of host symptoms. Statements made on the capacity of oseltamivir to interrupt viral transmission and reduce complications are not supported by any data we have been able to access. The mechanism of action proposed by the producers (influenza virus‐specific) does not fit the clinical evidence which suggests a multi‐system and central action.

UR - http://dx.doi.org/10.1002/14651858.CD008965.pub4

ER -

Record #2 of 32

Provider: John Wiley & Sons, Ltd

Content: text/plain; charset="UTF-8"

TY - JOUR

AN - CD006423

AU - Shyangdan, DS

AU - Royle, P

AU - Clar, C

AU - Sharma, P

AU - Waugh, N

AU - Snaith, A

TI - Glucagon‐like peptide analogues for type 2 diabetes mellitus

JF - Cochrane Database of Systematic Reviews

PY - 2011

IS - 10

PB - John Wiley & Sons, Ltd

SN - 1465-1858

KW - Diabetes Mellitus, Type 2 [blood, *drug therapy]

KW - Glucagon‐Like Peptide 1 [*analogs & derivatives]

KW - Glycated Hemoglobin A [metabolism]

KW - Humans

KW - Hypoglycemic Agents [*therapeutic use]

KW - Randomized Controlled Trials as Topic

N1 - [Metabolic and Endocrine Disorders]

DO - 10.1002/14651858.CD006423.pub2

AB - Abstract - Background Glucagon‐like peptide analogues are a new class of drugs used in the treatment of type 2 diabetes that mimic the endogenous hormone glucagon‐like peptide 1 (GLP‐1). GLP‐1 is an incretin, a gastrointestinal hormone that is released into the circulation in response to ingested nutrients. GLP‐1 regulates glucose levels by stimulating glucose‐dependent insulin secretion and biosynthesis, and by suppressing glucagon secretion, delayed gastric emptying and promoting satiety. Objectives To assess the effects of glucagon‐like peptide analogues in patients with type 2 diabetes mellitus. Search methods Studies were obtained from electronic searches of The Cochrane Library (last search issue 1, 2011), MEDLINE (last search March 2011), EMBASE (last search March 2011), Web of Science (last search March 2011) and databases of ongoing trials. Selection criteria Studies were included if they were randomised controlled trials of a minimum duration of eight weeks comparing a GLP‐1 analogue with placebo, insulin, an oral anti‐diabetic agent, or another GLP‐1 analogue in people with type 2 diabetes. Data collection and analysis Data extraction and quality assessment of studies were done by one reviewer and checked by a second. Data were analysed by type of GLP‐1 agonist and comparison treatment. Where appropriate, data were summarised in a meta‐analysis (mean differences and risk ratios summarised using a random‐effects model). Main results Seventeen randomised controlled trials including relevant analyses for 6899 participants were included in the analysis. Studies were mostly of short duration, usually 26 weeks. In comparison with placebo, all GLP‐1 agonists reduced glycosylated haemoglobin A1c (HbA1c) levels by about 1%. Exenatide 2 mg once weekly and liraglutide 1.8 mg reduced it by 0.20% and 0.24% respectively more than insulin glargine. Exenatide 2 mg once weekly reduced HbA1c more than exenatide 10 μg twice daily, sitagliptin and pioglitazone. Liraglutide 1.8 mg reduced HbA1c by 0.33% more than exenatide 10 μg twice daily. Liraglutide led to similar improvements in HbA1c compared to sulphonylureas but reduced it more than sitagliptin and rosiglitazone. Both exenatide and liraglutide led to greater weight loss than most active comparators, including in participants not experiencing nausea. Hypoglycaemia occurred more frequently in participants taking concomitant sulphonylurea. GLP‐1 agonists caused gastrointestinal adverse effects, mainly nausea. These adverse events were strongest at the beginning and then subsided. Beta‐cell function was improved with GLP‐1 agonists but the effect did not persist after cessation of treatment. None of the studies was long enough to assess long‐term positive or negative effects. Authors' conclusions GLP‐1 agonists are effective in improving glycaemic control. Plain language summary Glucagon‐like peptide analogues for type 2 diabetes Glucagon‐like peptide analogues or agonists are a new kind of drug in the treatment of type 2 diabetes that are given by injection under the skin. They regulate glucose levels by stimulating glucose‐dependent insulin secretion and biosynthesis, and by suppressing glucagon secretion, delaying gastric emptying and promoting satiety. Various glucagon‐like peptide‐1 agonists are in use or in the licensing process, including exenatide, liraglutide, albiglutide, taspoglutide, lixisenatide and LY2189265. Seventeen randomised controlled trials of mostly moderate to high quality randomised approximately 6899 people with type 2 diabetes mellitus. Studies were mostly of short duration, usually 26 weeks. The longest duration study was 30 weeks. Of the seventeen studies, one compared albiglutide with placebo, two compared exenatide 10 µg twice daily against exenatide 2 mg once weekly, one compared exenatide 2 mg once weekly against insulin glargine, one compared exenatide 2 mg once weekly against pioglitazone and sitagliptin, five compared liraglutide with placebo, two compared liraglutide with sulphonylurea, one each compared exenatide twice daily with liraglutide, liraglutide with sitagliptin, liraglutide with rosiglitazone and liraglutide with insulin glargine, two compared taspoglutide with placebo and one each compared lixisenatide with placebo and LY2189265 with placebo. In people already treated with oral anti‐diabetes drugs, addition of glucagon‐like peptide analogues improved blood sugar control in comparison to placebo, rosiglitazone, pioglitazone or sitagliptin, but not always in comparison to insulin (for exenatide) or glimepiride (a sulphonylurea). Glucagon‐like peptide analogous caused more weight loss than any of the comparison treatments. However, more nausea and other gastrointestinal effects such as diarrhoea or vomiting were seen, though these tended to wear off and were not seen in all participants. There was slightly more hypoglycaemia with glucagon‐like analogous than with placebo, but generally less than with other anti‐diabetic treatments. The incidence of hypoglycaemia occurred more frequently in participants taking concomitant sulphonylurea. The studies were not long enough to assess long‐term side effects. None of the studies investigated mortality or morbidity.

UR - http://dx.doi.org/10.1002/14651858.CD006423.pub2

ER -

Record #3 of 32

Provider: John Wiley & Sons, Ltd

Content: text/plain; charset="UTF-8"

TY - JOUR

AN - CD013879

AU - Pellicori, P

AU - Doolub, G

AU - Wong, CM

AU - Lee, KS

AU - Mangion, K

AU - Ahmad, M

AU - Berry, C

AU - Squire, I

AU - Lambiase, PD

AU - Lyon, A

AU - et al.

TI - COVID‐19 and its cardiovascular effects: a systematic review of prevalence studies

JF - Cochrane Database of Systematic Reviews

PY - 2021

IS - 3

PB - John Wiley & Sons, Ltd

SN - 1465-1858

KW - Arrhythmias, Cardiac [epidemiology]

KW - COVID-19 [*epidemiology, mortality]

KW - Cardiovascular Diseases [*epidemiology]

KW - Comorbidity

KW - Diabetes Mellitus [epidemiology]

KW - Heart Failure [epidemiology]

KW - Hospitalization [statistics & numerical data]

KW - Humans

KW - Hypertension [epidemiology]

KW - Incidence

KW - Myocardial Ischemia [epidemiology]

KW - Obesity [epidemiology]

KW - Prevalence

KW - Thrombosis [epidemiology]

N1 - [Heart]

DO - 10.1002/14651858.CD013879

AB - Abstract - Background A small minority of people with coronavirus disease 2019 (COVID‐19) develop a severe illness, characterised by inflammation, microvascular damage and coagulopathy, potentially leading to myocardial injury, venous thromboembolism (VTE) and arterial occlusive events. People with risk factors for or pre‐existing cardiovascular disease may be at greater risk. Objectives To assess the prevalence of pre‐existing cardiovascular comorbidities associated with suspected or confirmed cases of COVID‐19 in a variety of settings, including the community, care homes and hospitals. We also assessed the nature and rate of subsequent cardiovascular complications and clinical events in people with suspected or confirmed COVID‐19. Search methods We conducted an electronic search from December 2019 to 24 July 2020 in the following databases: the Cochrane Central Register of Controlled Trials (CENTRAL), MEDLINE, Embase, covid‐19.cochrane.org, ClinicalTrials.gov and EU Clinical Trial Register. Selection criteria We included prospective and retrospective cohort studies, controlled before‐and‐after, case‐control and cross‐sectional studies, and randomised controlled trials (RCTs). We analysed controlled trials as cohorts, disregarding treatment allocation. We only included peer‐reviewed studies with 100 or more participants, and excluded articles not written in English or only published in pre‐print servers. Data collection and analysis Two review authors independently screened the search results and extracted data. Given substantial variation in study designs, reported outcomes and outcome metrics, we undertook a narrative synthesis of data, without conducting a meta‐analysis. We critically appraised all included studies using the Joanna Briggs Institute (JBI) checklist for prevalence studies and the JBI checklist for case series. Main results We included 220 studies. Most of the studies originated from China (47.7%) or the USA (20.9%); 9.5% were from Italy. A large proportion of the studies were retrospective (89.5%), but three (1.4%) were RCTs and 20 (9.1%) were prospective. Using JBI’s critical appraisal checklist tool for prevalence studies, 75 studies attained a full score of 9, 57 studies a score of 8, 31 studies a score of 7, 5 studies a score of 6, three studies a score of 5 and one a score of 3; using JBI’s checklist tool for case series, 30 studies received a full score of 10, six studies a score of 9, 11 studies a score of 8, and one study a score of 5 We found that hypertension (189 studies, n = 174,414, weighted mean prevalence (WMP): 36.1%), diabetes (197 studies, n = 569,188, WMP: 22.1%) and ischaemic heart disease (94 studies, n = 100,765, WMP: 10.5%) are highly prevalent in people hospitalised with COVID‐19, and are associated with an increased risk of death. In those admitted to hospital, biomarkers of cardiac stress or injury are often abnormal, and the incidence of a wide range of cardiovascular complications is substantial, particularly arrhythmias (22 studies, n = 13,115, weighted mean incidence (WMI) 9.3%), heart failure (20 studies, n = 29,317, WMI: 6.8%) and thrombotic complications (VTE: 16 studies, n = 7700, WMI: 7.4%). Authors' conclusions This systematic literature review indicates that cardiometabolic comorbidities are common in people who are hospitalised with a COVID‐19 infection, and cardiovascular complications are frequent. We plan to update this review and to conduct a formal meta‐analysis of outcomes based on a more homogeneous selected subsample of high‐certainty studies. Plain language summary What type of heart and blood vessel problems complicate COVID‐19 infections, how common are they and what other medical conditions do these patients have? Background Many people infected by COVID‐19 have few or no symptoms. However, COVID‐19 can make the blood ‘sticky’, clogging up both small blood vessels (capillaries) and large ones, which may cause heart attacks, strokes or blood clots in the legs or lungs. These can be fatal. People who have diabetes, high blood pressure or pre‐existing heart problems are at greater risk of developing such complications if they get COVID‐19. Our research question We wanted to find out, in cases of confirmed or suspected COVID‐19: ‐ what are the most common pre‐existing heart and blood vessel (cardiovascular) problems (for example, diabetes, high blood pressure and obesity) ‐ what are the most common complications affecting the heart and blood vessels (for example, irregular heartbeat, blood clots, heart failure and stroke) in different setting (in the community, care homes or in hospital). What we did We searched for published studies that reported heart and blood vessel problems in people with possible or confirmed COVID‐19. Studies could be of any design and could take place anywhere, but they had to have been checked by other researchers (be peer‐reviewed), be written in English, and include at least 100 cases. The evidence is current until July 2020. What we found We found 220 studies that reported relevant information, but the quality of the information was often poor. Studies were mostly from China and the USA. Most studies only had information on the small minority of cases that were admitted to hospital with COVID‐19, often to the intensive care unit. We found that high blood pressure, diabetes and heart disease are very common in people hospitalised with COVID‐19 and are associated with an increased risk of death. More than one‐third of patients with COVID‐19 had a history of high blood pressure, 23.5% had a pre‐existing heart or blood vessel problem, 22.1% had diabetes, and 21.6% were obese (many people had more than one of these conditions). The most common cardiovascular complication in people with COVID‐19 was an irregular heartbeat (atrial fibrillation; 8.5%). Blood clots in the legs (6.1%) or lungs (4.3%), and heart failure (6.8%) were also common, but the reported rates may be underestimated because the studies did not always carry out appropriate investigations. Heart attacks (1.7%) and strokes (1.2%) were reported less often. Blood tests also often suggested heart damage or stress. Next steps The studies focused on people in hospital, with severe COVID‐19, so the results may not apply to people who had milder COVID‐19 who were not hospitalised. The studies were very different from each other and did not always report the results in the same way or use the most reliable methods. Accordingly, our confidence in the precision of the prevalence of pre‐existing disease and of cardiovascular complications is not high. We plan to update this review. However, in future, we will focus only on higher‐quality evidence to increase the strength of our findings.

UR - http://dx.doi.org/10.1002/14651858.CD013879

ER -

Record #4 of 32

Provider: John Wiley & Sons, Ltd

Content: text/plain; charset="UTF-8"

TY - JOUR

AN - CD006652

AU - Akl, EA

AU - Kahale, LA

AU - Hakoum, MB

AU - Matar, CF

AU - Sperati, F

AU - Barba, M

AU - Yosuico, VED

AU - Terrenato, I

AU - Synnot, A

AU - Schünemann, H

TI - Parenteral anticoagulation in ambulatory patients with cancer

JF - Cochrane Database of Systematic Reviews

PY - 2017

IS - 9

PB - John Wiley & Sons, Ltd

SN - 1465-1858

KW - Anticoagulants [*administration & dosage, adverse effects]

KW - Cause of Death

KW - Hemorrhage [chemically induced, epidemiology]

KW - Heparin [*administration & dosage, adverse effects]

KW - Heparin, Low-Molecular-Weight [administration & dosage]

KW - Humans

KW - Neoplasms [*mortality]

KW - Quality of Life

KW - Randomized Controlled Trials as Topic

KW - Survival Analysis

KW - Time Factors

KW - Venous Thromboembolism [epidemiology, *prevention & control]

KW - Warfarin [administration & dosage]

N1 - [Gynaecological, Neuro-oncology and Orphan Cancer]

DO - 10.1002/14651858.CD006652.pub5

AB - Abstract - Background Anticoagulation may improve survival in patients with cancer through a speculated anti‐tumour effect, in addition to the antithrombotic effect, although may increase the risk of bleeding. Objectives To evaluate the efficacy and safety of parenteral anticoagulants in ambulatory patients with cancer who, typically, are undergoing chemotherapy, hormonal therapy, immunotherapy or radiotherapy, but otherwise have no standard therapeutic or prophylactic indication for anticoagulation. Search methods A comprehensive search included (1) a major electronic search (February 2016) of the following databases: Cochrane Central Register of Controlled Trials (CENTRAL) (2016, Issue 1), MEDLINE (1946 to February 2016; accessed via OVID) and Embase (1980 to February 2016; accessed via OVID); (2) handsearching of conference proceedings; (3) checking of references of included studies; (4) use of the 'related citation' feature in PubMed and (5) a search for ongoing studies in trial registries. As part of the living systematic review approach, we are running searches continually and we will incorporate new evidence rapidly after it is identified. This update of the systematic review is based on the findings of a literature search conducted on 14 August 2017. Selection criteria Randomized controlled trials (RCTs) assessing the benefits and harms of parenteral anticoagulation in ambulatory patients with cancer. Typically, these patients are undergoing chemotherapy, hormonal therapy, immunotherapy or radiotherapy, but otherwise have no standard therapeutic or prophylactic indication for anticoagulation. Data collection and analysis Using a standardized form we extracted data in duplicate on study design, participants, interventions outcomes of interest, and risk of bias. Outcomes of interested included all‐cause mortality, symptomatic venous thromboembolism (VTE), symptomatic deep vein thrombosis (DVT), pulmonary embolism (PE), major bleeding, minor bleeding, and quality of life. We assessed the certainty of evidence for each outcome using the GRADE approach (GRADE handbook). Main results Of 6947 identified citations, 19 RCTs fulfilled the eligibility criteria. These trials enrolled 9650 participants. Trial registries' searches identified nine registered but unpublished trials, two of which were labeled as 'ongoing trials'. In all included RCTs, the intervention consisted of heparin (either unfractionated heparin or low molecular weight heparin). Overall, heparin appears to have no effect on mortality at 12 months (risk ratio (RR) 0.98; 95% confidence interval (CI) 0.93 to 1.03; risk difference (RD) 10 fewer per 1000; 95% CI 35 fewer to 15 more; moderate certainty of evidence) and mortality at 24 months (RR 0.99; 95% CI 0.96 to 1.01; RD 8 fewer per 1000; 95% CI 31 fewer to 8 more; moderate certainty of evidence). Heparin therapy reduces the risk of symptomatic VTE (RR 0.56; 95% CI 0.47 to 0.68; RD 30 fewer per 1000; 95% CI 36 fewer to 22 fewer; high certainty of evidence), while it increases in the risks of major bleeding (RR 1.30; 95% 0.94 to 1.79; RD 4 more per 1000; 95% CI 1 fewer to 11 more; moderate certainty of evidence) and minor bleeding (RR 1.70; 95% 1.13 to 2.55; RD 17 more per 1000; 95% CI 3 more to 37 more; high certainty of evidence). Results failed to confirm or to exclude a beneficial or detrimental effect of heparin on thrombocytopenia (RR 0.69; 95% CI 0.37 to 1.27; RD 33 fewer per 1000; 95% CI 66 fewer to 28 more; moderate certainty of evidence); quality of life (moderate certainty of evidence). Authors' conclusions Heparin appears to have no effect on mortality at 12 months and 24 months. It reduces symptomatic VTE and likely increases major and minor bleeding. Future research should further investigate the survival benefit of different types of anticoagulants in patients with different types and stages of cancer. The decision for a patient with cancer to start heparin therapy should balance the benefits and downsides, and should integrate the patient's values and preferences. Editorial note:This is a living systematic review. Living systematic reviews offer a new approach to review updating in which the review is continually updated, incorporating relevant new evidence, as it becomes available. Please refer to the Cochrane Database of Systematic Reviews for the current status of this review. Plain language summary Injectable blood thinners (anticoagulants) in patients with cancer Background  Research evidence suggests that blood thinners may improve the survival of patients with cancer, by preventing life‐threatening blood clots and might also have a direct anticancer effect. However, blood thinners can also increase the risk of bleeding, which can be serious and reduce survival. It is therefore important to understand the pros and cons of treatment to allow patients and their doctors to be aware of the balance of risks and benefits. Study characteristics  We searched the scientific literature for studies of anticoagulants in people with cancer. The evidence is current to 14 August 2017. We included 19 eligible trials. Key results  We selected 19 trials including 9650 participants with cancer. Most trials included participants with various types of cancer, especially small cell lung cancer, non‐small cell lung cancer, and pancreatic cancer. All studies were conducted in the outpatient setting. The results suggest that the effect of injectable blood thinners on survival is uncertain, but if anything of small size. Also the results suggest that injectable blood thinners reduce the risk of blood clots by about half and possibly increase the risk of major bleeding and minor bleeding by 4 more per 1000 and 17 more per 1000, respectively. The effect on quality of life is uncertain. Certainty of evidence  We judged the certainty of evidence to be high for symptomatic VTE and minor bleeding, and moderate for mortality, major bleeding and quality of life. Editorial note: This is a living systematic review. Living systematic reviews offer a new approach to review updating in which the review is continually updated, incorporating relevant new evidence, as it becomes available. Please refer to the Cochrane Database of Systematic Reviews for the current status of this review.

UR - http://dx.doi.org/10.1002/14651858.CD006652.pub5

ER -

Record #5 of 32

Provider: John Wiley & Sons, Ltd

Content: text/plain; charset="UTF-8"

TY - JOUR

AN - CD007478

AU - Hannon, CW

AU - McCourt, C

AU - Lima, HC

AU - Chen, S

AU - Bennett, C

TI - Interventions for cutaneous disease in systemic lupus erythematosus

JF - Cochrane Database of Systematic Reviews

PY - 2021

IS - 3

PB - John Wiley & Sons, Ltd

SN - 1465-1858

KW - Age of Onset

KW - Azathioprine [therapeutic use]

KW - Bias

KW - Biological Factors [therapeutic use]

KW - Chloroquine [adverse effects, therapeutic use]

KW - Cosmetic Techniques

KW - Cyclosporine [therapeutic use]

KW - Dermatologic Agents [adverse effects, *therapeutic use]

KW - Exanthema

KW - Female

KW - Humans

KW - Hydroxychloroquine [adverse effects, therapeutic use]

KW - Immunosuppressive Agents [*therapeutic use]

KW - Lupus Erythematosus, Cutaneous [classification, diagnosis, therapy]

KW - Lupus Erythematosus, Systemic [classification, complications, *therapy]

KW - Male

KW - Medicine, Chinese Traditional

KW - Methotrexate [adverse effects, therapeutic use]

KW - Placebos [therapeutic use]

KW - Quality of Life

KW - Randomized Controlled Trials as Topic

KW - Skin Diseases [etiology, *therapy]

KW - Symptom Flare Up

N1 - [Skin]

DO - 10.1002/14651858.CD007478.pub2

AB - Abstract - Background Lupus erythematosus is an autoimmune disease with significant morbidity and mortality. Cutaneous disease in systemic lupus erythematosus (SLE) is common. Many interventions are used to treat SLE with varying efficacy, risks, and benefits. Objectives To assess the effects of interventions for cutaneous disease in SLE. Search methods We searched the following databases up to June 2019: the Cochrane Skin Specialised Register, CENTRAL, MEDLINE, Embase, Wiley Interscience Online Library, and Biblioteca Virtual em Saude (Virtual Health Library). We updated our search in September 2020, but these results have not yet been fully incorporated. Selection criteria We included randomised controlled trials (RCTs) of interventions for cutaneous disease in SLE compared with placebo, another intervention, no treatment, or different doses of the same intervention. We did not evaluate trials of cutaneous lupus in people without a diagnosis of SLE. Data collection and analysis We used standard methodological procedures expected by Cochrane. Primary outcomes were complete and partial clinical response. Secondary outcomes included reduction (or change) in number of clinical flares; and severe and minor adverse events. We used GRADE to assess the quality of evidence. Main results Sixty‐one RCTs, involving 11,232 participants, reported 43 different interventions. Trials predominantly included women from outpatient clinics; the mean age range of participants was 20 to 40 years. Twenty‐five studies reported baseline severity, and 22 studies included participants with moderate to severe cutaneous lupus erythematosus (CLE); duration of CLE was not well reported. Studies were conducted mainly in multi‐centre settings. Most often treatment duration was 12 months. Risk of bias was highest for the domain of reporting bias, followed by performance/detection bias. We identified too few studies for meta‐analysis for most comparisons. We limited this abstract to main comparisons (all administered orally) and outcomes. We did not identify clinical trials of other commonly used treatments, such as topical corticosteroids, that reported complete or partial clinical response or numbers of clinical flares. Complete clinical response Studies comparing oral hydroxychloroquine against placebo did not report complete clinical response. Chloroquine may increase complete clinical response at 12 months' follow‐up compared with placebo (absence of skin lesions) (risk ratio (RR) 1.57, 95% confidence interval (CI) 0.95 to 2.61; 1 study, 24 participants; low‐quality evidence). There may be little to no difference between methotrexate and chloroquine in complete clinical response (skin rash resolution) at 6 months' follow‐up (RR 1.13, 95% CI 0.84 to 1.50; 1 study, 25 participants; low‐quality evidence). Methotrexate may be superior to placebo with regard to complete clinical response (absence of malar/discoid rash) at 6 months' follow‐up (RR 3.57, 95% CI 1.63 to 7.84; 1 study, 41 participants; low‐quality evidence). At 12 months' follow‐up, there may be little to no difference between azathioprine and ciclosporin in complete clinical response (malar rash resolution) (RR 0.83, 95% CI 0.46 to 1.52; 1 study, 89 participants; low‐quality evidence). Partial clinical response Partial clinical response was reported for only one key comparison: hydroxychloroquine may increase partial clinical response at 12 months compared to placebo, but the 95% CI indicates that hydroxychloroquine may make no difference or may decrease response (RR 7.00, 95% CI 0.41 to 120.16; 20 pregnant participants, 1 trial; low‐quality evidence). Clinical flares Clinical flares were reported for only two key comparisons: hydroxychloroquine is probably superior to placebo at 6 months' follow‐up for reducing clinical flares (RR 0.49, 95% CI 0.28 to 0.89; 1 study, 47 participants; moderate‐quality evidence). At 12 months' follow‐up, there may be no difference between methotrexate and placebo, but the 95% CI indicates there may be more or fewer flares with methotrexate (RR 0.77, 95% CI 0.32 to 1.83; 1 study, 86 participants; moderate‐quality evidence). Adverse events Data for adverse events were limited and were inconsistently reported, but hydroxychloroquine, chloroquine, and methotrexate have well‐documented adverse effects including gastrointestinal symptoms, liver problems, and retinopathy for hydroxychloroquine and chloroquine and teratogenicity during pregnancy for methotrexate. Authors' conclusions Evidence supports the commonly‐used treatment hydroxychloroquine, and there is also evidence supporting chloroquine and methotrexate for treating cutaneous disease in SLE. Evidence is limited due to the small number of studies reporting key outcomes. Evidence for most key outcomes was low or moderate quality, meaning findings should be interpreted with caution. Head‐to‐head intervention trials designed to detect differences in efficacy between treatments for specific CLE subtypes are needed. Thirteen further trials are awaiting classification and have not yet been incorporated in this review; they may alter the review conclusions. Plain language summary What are the benefits and risks of different treatments for skin disease in people with systemic lupus erythematosus (an autoimmune disease that affects the whole body) Why is this question important? Systemic lupus erythematosus (SLE; also known as ‘lupus’) is a disease in which the body's immune (defence) system mistakenly attacks healthy tissue in many parts of the body. It affects 7.5 million people worldwide. Around 70% of affected people develop skin problems such as rash on the nose or cheeks. Often, SLE also causes pain in joints and muscles and extreme tiredness. Symptoms can improve temporarily, or they can worsen suddenly (flares). In severe cases, SLE can cause life‐threatening damage to the heart, lungs, brain, or kidneys. There is no cure for SLE. However, there are treatments designed to improve symptoms. In particular, there are a range of options for treating skin problems. • Medicines that can be taken by mouth (orally), applied as creams, or given as injections. • Therapies to help people cope with their skin problems, such as talking therapies. • Other approaches, including herbal medicine, light therapy, or make‐up. To find out which treatments work best for people with SLE, and to compare adverse (unwanted) effects, we reviewed the evidence from research studies. How did we identify and evaluate the evidence? We searched the medical literature for studies that compared any treatment for skin disease in SLE against: • a placebo (dummy) treatment; • no treatment; • another treatment; or • a different dose of the same treatment. We compared the results and summarised the evidence from all the studies. Finally, we rated our confidence in the evidence based on factors such as study methods and sizes and the consistency of findings across studies. What did we find? We found 61 studies that included 11,232 people (mostly women) and investigated 43 different treatments. Most treatments lasted one year, and people were followed for up to 48 months. Here we report the main findings of our review on the effects of five different oral medicines: hydroxychloroquine, chloroquine, methotrexate, ciclosporin, and azathioprine. Disappearance of skin problems We do not know if hydroxychloroquine is better or worse than placebo at making skin problems disappear because no studies reported information about this. The evidence suggests that: • chloroquine may be better at making skin problems disappear after 12 months than placebo (1 study, 24 people); • when we compare methotrexate and choloroquine, there may be little to no difference in how often they make skin rashes disappear after six months (1 study, 25 people); • methotrexate may be better for making skin rashes disappear after six months than placebo (1 study, 41 people); and • there may be little to no difference in how often skin problems disappear after 12 months between ciclosporin and aziathropine (1 study, 25 people). Partial disappearance of skin problems (at least 50% improvement in the skin condition) It is unclear if hydroxycholoroquine is better or worse than placebo at making skin problems disappear at least partially after 12 months. This is because the evidence is too imprecise (1 study, 20 pregnant women). No other studies have examined how treatments affect the partial disappearance of skin problems. Flares The evidence suggests that after six months, fewer flares probably occur with hydroxychloroquine than with placebo (1 study, 47 people). It is unclear if flares are more, or less, likely to occur after 12 months with methotrexate compared to placebo (1 study, 86 people). No other studies have reported information on how treatments affect flares. Adverse events Evidence is often imprecise, and whether treatments lead to more or fewer adverse events than placebo or other treatments is not clear. We found limited data for adverse events, and reports were discrepant, but hydroxychloroquine, chloroquine, and methotrexate have well‐known adverse effects including stomach and liver problems. Hydroxychloroquine and chloroquine can cause eye problems, and methotrexate can cause serious harm to a developing baby if taken during pregnancy. Other outcomes We do not know how treatments affect other aspects of disease severity or quality of life. This is because studies did not report information on this. What does this mean? When compared against a placebo, studies in people with SLE show that: • fewer flares probably occur with hydroxychloroquine; and • methotrexate and chloroquine may be better at making skin problems disappear. Information about adverse effects is limited. How up‐to‐date is this review? The evidence in this Cochrane Review is current to June 2019.

UR - http://dx.doi.org/10.1002/14651858.CD007478.pub2

ER -

Record #6 of 32

Provider: John Wiley & Sons, Ltd

Content: text/plain; charset="UTF-8"

TY - JOUR

AN - CD012838

AU - Narayan, V

AU - Kahlmeyer, A

AU - Dahm, P

AU - Skoetz, N

AU - Risk, MC

AU - Bongiorno, C

AU - Patel, N

AU - Hwang, EC

AU - Jung, JH

AU - Gartlehner, G

AU - et al.

TI - Pembrolizumab monotherapy versus chemotherapy for treatment of advanced urothelial carcinoma with disease progression during or following platinum‐containing chemotherapy. A Cochrane Rapid Review

JF - Cochrane Database of Systematic Reviews

PY - 2018

IS - 7

PB - John Wiley & Sons, Ltd

SN - 1465-1858

KW - Antibodies, Monoclonal, Humanized [adverse effects, *therapeutic use]

KW - Antineoplastic Agents, Immunological [adverse effects, *therapeutic use]

KW - Antineoplastic Combined Chemotherapy Protocols [adverse effects, *therapeutic use]

KW - Carcinoma [*drug therapy, pathology]

KW - Disease Progression

KW - Docetaxel

KW - Humans

KW - Paclitaxel [administration & dosage]

KW - Quality of Life

KW - Taxoids [administration & dosage]

KW - Urinary Bladder Neoplasms [*drug therapy, pathology]

KW - Vinblastine [administration & dosage, analogs & derivatives]

N1 - [Urology]

DO - 10.1002/14651858.CD012838.pub2

AB - Abstract - Background The use of systemic immunotherapy targets is emerging as an important treatment option for metastatic urothelial carcinoma, particularly for patients who cannot tolerate or who fail cisplatin‐based chemotherapy. One such target is the inhibition of the checkpoint protein programmed cell death‐1 (PD‐1) receptor and its ligand (PD‐L1) by monoclonal antibodies. Objectives To assess the effects of pembrolizumab monotherapy versus chemotherapy for treatment of advanced urothelial carcinoma with disease progression during or following platinum‐containing chemotherapy. Search methods We performed a Cochrane Rapid Review, limiting our search to published studies in the English language. We searched databases of the medical literature, including the Cochrane Central Register of Controlled Trials and MEDLINE, as well as trial registries including ClinicalTrials.gov and the World Health Organization International Clinical Trials Registry Platform (WHO ICTRP). Our search extended from January 2000 to June 2018. Selection criteria We included randomised controlled trials except cross‐over trials and cluster randomised trials. We excluded all other study designs. Participants included had locally advanced or metastatic urothelial carcinoma of the bladder, with disease progression during or following platinum‐containing chemotherapy (synonymous with second‐/third‐/fourth‐line therapy). This review focused on pembrolizumab (synonyms: MK‐3475, lambrolizumab, Keytruda). Data collection and analysis Two review authors independently classified and abstracted data from the included study. The certainty of evidence was rated according to the Grading of Recommendations Assessment, Development and Evaluation (GRADE) approach. Main results We identified one randomised controlled trial that included 542 participants, which compared the use of pembrolizumab monotherapy versus chemotherapy for the treatment of advanced urothelial carcinoma with disease progression during or following platinum‐containing chemotherapy. Results were reported after a median follow‐up of 14.1 months (range 9.9 to 22.1 months). Primary outcomes Pembrolizumab probably reduces the risk of death from any cause (hazard ratio (HR) 0.73, 95% confidence interval (CI) 0.59 to 0.90; moderate certainty evidence). This corresponds to 115 fewer deaths (191 fewer to 38 fewer) per 1000 participants with pembrolizumab at 12 months. We downgraded the certainty of evidence one level for imprecision. Pembrolizumab may slightly improve quality of life (change from baseline to week 15 assessed with the Core Quality of Life Questionnaire; higher value reflects better quality of life; scale 0 to 100) with a mean difference (MD) of 9.05, 95% CI 4.61 to 13.50; low certainty evidence). We downgraded the certainty of evidence two levels for study limitations and imprecision. Secondary outcomes Pembrolizumab may have little or no effect on disease progression (HR 0.98, 95% CI 0.81 to 1.19; low certainty evidence). This corresponds to three fewer patients (42 fewer to 24 more) whose disease progressed per 1000 participants at 12 months. We downgraded the certainty of evidence two levels for study limitations and imprecision. Pembrolizumab probably improves treatment response (based on complete or partial radiologic response) with a risk ratio (RR) of 1.85, 95% CI 1.24 to 2.77; moderate certainty evidence). This corresponds to 97 more respondents (27 more to 202 more) per 1000 participants with pembrolizumab. We downgraded the certainty of evidence one level for imprecision. Pembrolizumab may have little or no effect on treatment‐related mortality (RR 0.96, 95% CI 0.24 to 3.79; low certainty evidence). This corresponds to one fewer (12 fewer to 44 more) treatment‐related deaths per 1000 participants with pembrolizumab. We downgraded the certainty of evidence two levels for study limitations and imprecision. Pembrolizumab may have little or no effect on discontinuations due to adverse events (RR 0.66, 95% CI 0.39 to 1.10). This corresponds to 54 fewer discontinuations per 1000 participants (95% CI 79 fewer to 7 more). We downgraded the certainty of evidence for study limitations and imprecision. Pembrolizumab may reduce serious adverse events (RR 0.83, 95 CI 0.72 to 0.97; low certainty evidence). This corresponds to 107 fewer serious averse events per 1000 participants (95% CI 19 fewer to 176 fewer). We downgraded two levels for study limitations and imprecision. Authors' conclusions The use of pembrolizumab in men with advanced urothelial carcinoma with disease progression during or following platinum‐containing chemotherapy probably improves overall survival when compared with chemotherapy alone. At 12 months follow‐up about 70% of those in the chemotherapy group had died, compared with 59% of those treated with pembrolizumab. We are very uncertain about the effects of pembolizumab on quality of life. Pembolizumab may also improve treatment response rates, and reduce the risk of serious adverse events, but may make little or no difference to discontinuations of treatment due to adverse events. These conclusions are based on a single trial that was sponsored by the producer of pembrolizumab. Plain language summary Pembrolizumab versus chemotherapy for treating advanced bladder cancer after recurrence/progression following platinum‐based chemotherapy Review question How does pembrolizumab (a newer medicine that works through the body's immune system) compare to chemotherapy in patients with cancer of the inner lining of the urinary system, called urothelial cancer, that has either come back or worsened after treatment? Background Medications that target the body's immune system have been used for a long time to treat urothelial cancer. When the cancer has spread to other organs outside the urinary tract, patients are often treated with chemotherapy using medicines called cisplatin or carboplatin (platinum‐containing chemotherapy). However, often the cancer comes back or becomes worse despite treatment. This review considers the evidence for pembrolizumab, which is a member of a new class of medications that work through the immune sytem, and compares it to chemotherapy. Study characteristics We considered only randomised controlled trials in this Cochrane Rapid Review, as they offer the most reliable results. This review is current to 20 June 2018. Key results We found only one randomised study for our question. Participants included in this trial had metastatic (cancer that has spread to other parts of the body) or advanced cancer that could not be removed by surgery, that had come back or worsened with other chemotherapy. We found that pembrolizumab probably improves overall survival a little (evidence of moderate certainty). It may improve quality of life slightly (low certainty evidence). Pembrolizumab may have little or not effect on the time for the cancer to worsen or advance (low certainty evidence). It probably improves treatment response as seen on X‐ray scans such as computer tomography (moderate certainty of evidence). Pembrolizumab may have little or no effect on deaths resulting from the treatment itself (low certainty evidence) but may result in fewer patients stopping treatment due to unwanted side effects (low certainty evidence). It may also cause less serious side effects. These conclusions are based on a single trial paid for by the company that makes pembrolizumab. Certainty of evidence The certainty of evidence ranged from moderate to very low.

UR - http://dx.doi.org/10.1002/14651858.CD012838.pub2

ER -

Record #7 of 32

Provider: John Wiley & Sons, Ltd

Content: text/plain; charset="UTF-8"

TY - JOUR

AN - CD012143

AU - Jakobsen, JC

AU - Nielsen, EE

AU - Feinberg, J

AU - Katakam, KK

AU - Fobian, K

AU - Hauser, G

AU - Poropat, G

AU - Djurisic, S

AU - Weiss, KH

AU - Bjelakovic, M

AU - et al.

TI - Direct‐acting antivirals for chronic hepatitis C

JF - Cochrane Database of Systematic Reviews

PY - 2017

IS - 9

PB - John Wiley & Sons, Ltd

SN - 1465-1858

KW - Antiviral Agents [adverse effects, *therapeutic use]

KW - Cause of Death

KW - Hepacivirus [drug effects]

KW - Hepatitis C, Chronic [complications, *drug therapy, mortality]

KW - Humans

KW - Nucleic Acid Synthesis Inhibitors [adverse effects, therapeutic use]

KW - Placebos [therapeutic use]

KW - Protease Inhibitors [adverse effects, therapeutic use]

KW - Randomized Controlled Trials as Topic

KW - Safety-Based Drug Withdrawals

KW - Simeprevir [adverse effects, therapeutic use]

N1 - [Hepato-Biliary]

DO - 10.1002/14651858.CD012143.pub3

AB - Abstract - Background Millions of people worldwide suffer from hepatitis C, which can lead to severe liver disease, liver cancer, and death. Direct‐acting antivirals (DAAs), e.g. sofosbuvir, are relatively new and expensive interventions for chronic hepatitis C, and preliminary results suggest that DAAs may eradicate hepatitis C virus (HCV) from the blood (sustained virological response). Sustained virological response (SVR) is used by investigators and regulatory agencies as a surrogate outcome for morbidity and mortality, based solely on observational evidence. However, there have been no randomised trials that have validated that usage. Objectives To assess the benefits and harms of DAAs in people with chronic HCV. Search methods We searched for all published and unpublished trials in The Cochrane Hepato‐Biliary Group Controlled Trials Register, CENTRAL, MEDLINE, Embase, Science Citation Index Expanded, LILACS, and BIOSIS; the Chinese Biomedical Literature Database (CBM), China Network Knowledge Information (CNKI), the Chinese Science Journal Database (VIP), Google Scholar, The Turning Research into Practice (TRIP) Database, ClinicalTrials.gov , European Medicines Agency (EMA) ( www.ema.europa.eu/ema/ ), WHO International Clinical Trials Registry Platform ( www.who.int/ictrp ), the Food and Drug Administration (FDA) ( www.fda.gov ), and pharmaceutical company sources for ongoing or unpublished trials. Searches were last run in October 2016. Selection criteria Randomised clinical trials comparing DAAs versus no intervention or placebo, alone or with co‐interventions, in adults with chronic HCV. We included trials irrespective of publication type, publication status, and language. Data collection and analysis We used standard methodological procedures expected by Cochrane. Our primary outcomes were hepatitis C‐related morbidity, serious adverse events, and health‐related quality of life. Our secondary outcomes were all‐cause mortality, ascites, variceal bleeding, hepato‐renal syndrome, hepatic encephalopathy, hepatocellular carcinoma, non‐serious adverse events (each reported separately), and SVR. We systematically assessed risks of bias, performed Trial Sequential Analysis, and followed an eight‐step procedure to assess thresholds for statistical and clinical significance. We evaluated the overall quality of the evidence, using GRADE. Main results We included a total of 138 trials randomising a total of 25,232 participants. The trials were generally short‐term trials and designed primarily to assess the effect of treatment on SVR. The trials evaluated 51 different DAAs. Of these, 128 trials employed matching placebo in the control group. All included trials were at high risk of bias. Eighty‐four trials involved DAAs on the market or under development (13,466 participants). Fifty‐seven trials administered DAAs that were discontinued or withdrawn from the market. Study populations were treatment‐naive in 95 trials, had been exposed to treatment in 17 trials, and comprised both treatment‐naive and treatment‐experienced individuals in 24 trials. The HCV genotypes were genotype 1 (119 trials), genotype 2 (eight trials), genotype 3 (six trials), genotype 4 (nine trials), and genotype 6 (one trial). We identified two ongoing trials. We could not reliably determine the effect of DAAs on the market or under development on our primary outcome of hepatitis C‐related morbidity or all‐cause mortality. There were no data on hepatitis C‐related morbidity and only limited data on mortality from 11 trials (DAA 15/2377 (0.63%) versus control 1/617 (0.16%); OR 3.72, 95% CI 0.53 to 26.18, very low‐quality evidence). We did not perform Trial Sequential Analysis on this outcome. There is very low quality evidence that DAAs on the market or under development do not influence serious adverse events (DAA 5.2% versus control 5.6%; OR 0.93, 95% CI 0.75 to 1.15 , 15,817 participants, 43 trials). The Trial Sequential Analysis showed that there was sufficient information to rule out that DAAs reduce the relative risk of a serious adverse event by 20% when compared with placebo. The only DAA that showed a lower risk of serious adverse events when meta‐analysed separately was simeprevir (OR 0.62, 95% CI 0.45 to 0.86). However, Trial Sequential Analysis showed that there was not enough information to confirm or reject a relative risk reduction of 20%, and when one trial with an extreme result was excluded, the meta‐analysis result showed no evidence of a difference. DAAs on the market or under development may reduce the risk of no SVR from 54.1% in untreated people to 23.8% in people treated with DAA (RR 0.44, 95% CI 0.37 to 0.52, 6886 participants, 32 trials, low quality evidence). Trial Sequential Analysis confirmed this meta‐analysis result. Only 1/84 trials on the market or under development assessed the effects of DAAs on health‐related quality of life (SF‐36 mental score and SF‐36 physical score). There was insufficient evidence from trials on withdrawn or discontinued DAAs to determine their effect on hepatitis C‐related morbidity and all‐cause mortality (OR 0.64, 95% CI 0.23 to 1.79; 5 trials, very low‐quality evidence). However, these DAAs seemed to increase the risk of serious adverse events (OR 1.45, 95% CI 1.22 to 1.73; 29 trials, very low‐quality evidence). Trial Sequential Analysis confirmed this meta‐analysis result. None of the 138 trials provided useful data to assess the effects of DAAs on the remaining secondary outcomes (ascites, variceal bleeding, hepato‐renal syndrome, hepatic encephalopathy, and hepatocellular carcinoma). Authors' conclusions The evidence for our main outcomes of interest come from short‐term trials, and we are unable to determine the effect of long‐term treatment with DAAs. The rates of hepatitis C morbidity and mortality observed in the trials are relatively low and we are uncertain as to how DAAs affect this outcome. Overall, there is very low quality evidence that DAAs on the market or under development do not influence serious adverse events. There is insufficient evidence to judge if DAAs have beneficial or harmful effects on other clinical outcomes for chronic HCV. Simeprevir may have beneficial effects on risk of serious adverse event. In all remaining analyses, we could neither confirm nor reject that DAAs had any clinical effects. DAAs may reduce the number of people with detectable virus in their blood, but we do not have sufficient evidence from randomised trials that enables us to understand how SVR affects long‐term clinical outcomes. SVR is still an outcome that needs proper validation in randomised clinical trials. Plain language summary Direct‐acting antivirals for chronic hepatitis C Background   Millions of people worldwide suffer from hepatitis C, which can lead to severe liver disease, liver cancer, and death. Numerous previous interferon‐based interventions have been used for hepatitis C, but none of these interventions have proven effective on patient‐centred outcomes and their use was associated with serious side‐effects. DAAs are relatively new but expensive interventions for hepatitis C, and preliminary results have shown that DAAs seem to eradicate hepatitis C virus from the blood (sustained virological response) much more frequently. In addition, these agents do appear to create much less serious adverse‐effects. In this Cochrane Review, we assessed the evidence on the clinical effects of DAAs for hepatitis C. Study characteristics   We included 138 randomised clinical trials. All included trials were at high risk of bias. The 138 trials used 51 different DAAs. Of these, 84 trials assessed DAAs on the market or under development; 57 trials were on DAAs withdrawn from development or the market. Trials were conducted from 2004 to 2016. The trials were from all over the world including 34 different countries. We included 17 trials where all the participants had previously been treated for hepatitis C (treatment‐experienced) before being included in the trial. There were 95 trials that included only participants who had not been previously treated for hepatitis C (treatment‐naive). The intervention periods ranged from one day to 48 weeks with an average of 14 weeks. The combined intervention period and follow‐up period ranged from one day to 120 weeks with an average of 34 weeks. Key results   We could not reliably determine the effect of DAAs on hepatitis C‐related morbidity or death from any cause. There were no data on hepatitis C‐related morbidity and very few deaths occurred over the course of the trials (15 deaths/2377 direct‐acting antiviral participants (0.63%) versus 1 death/617 control participants (0.16%), very low quality evidence). Based on very low quality evidence, 5.2% people treated with DAAs had one or more serious adverse events versus 5.6% participants who were untreated during the observation period. When analysed separately, simeprevir was the only direct‐acting antiviral that showed evidence of a beneficial effect when assessing risk of a serious adverse event. Our analyses, however, showed that the validity of this result is questionable and that 'play of chance' might be the cause for the difference. There was not enough information to determine if there was any effect of DAAs on other clinically relevant outcomes. Our results confirm that DAAs seem to reduce the number of people who have the hepatitis C virus in their blood from 54.1% in untreated people to 23.8% in those who were treated. Because the loss of detectable hepatitis C virus in the blood stream is only a blood test, the studies could not tell what this result means in the long term. Quality of the evidence   Due to several limitations (e.g. lack of blinding, lack of relevant data, missing data, no published protocol) we assessed the quality of the evidence in this review as very low or low quality. First, all trials and outcome results were at high risk of bias, which means that our results presumably overestimate the beneficial effects of DAAs and underestimate any potential harmful effects. Second, there were limited data on most of our clinical outcomes, that is, there were only relevant clinical data for meta‐analyses on all‐cause mortality and serious adverse events, and for these, data were sparse. There are no long‐term trials that have assessed whether or not DAA treatment improves morbidity or mortality.

UR - http://dx.doi.org/10.1002/14651858.CD012143.pub3

ER -

Record #8 of 32

Provider: John Wiley & Sons, Ltd

Content: text/plain; charset="UTF-8"

TY - JOUR

AN - CD012806

AU - Dinnes, J

AU - Ferrante di Ruffano, L

AU - Takwoingi, Y

AU - Cheung, ST

AU - Nathan, P

AU - Matin, RN

AU - Chuchu, N

AU - Chan, SA

AU - Durack, A

AU - Bayliss, SE

AU - et al.

TI - Ultrasound, CT, MRI, or PET‐CT for staging and re‐staging of adults with cutaneous melanoma

JF - Cochrane Database of Systematic Reviews

PY - 2019

IS - 7

PB - John Wiley & Sons, Ltd

SN - 1465-1858

KW - *Neoplasm Metastasis

KW - *Neoplasm Staging

KW - Adult

KW - Diagnosis, Computer‐Assisted [methods]

KW - Humans

KW - Magnetic Resonance Imaging

KW - Melanoma [*diagnostic imaging]

KW - Neoplasm Recurrence, Local [diagnostic imaging]

KW - Positron Emission Tomography Computed Tomography

KW - Randomized Controlled Trials as Topic

KW - Sensitivity and Specificity

KW - Skin Neoplasms [*diagnostic imaging]

KW - Tomography, X‐Ray Computed

KW - Ultrasonography

N1 - [Skin]

DO - 10.1002/14651858.CD012806.pub2

AB - Abstract - Background Melanoma is one of the most aggressive forms of skin cancer, with the potential to metastasise to other parts of the body via the lymphatic system and the bloodstream. Melanoma accounts for a small percentage of skin cancer cases but is responsible for the majority of skin cancer deaths. Various imaging tests can be used with the aim of detecting metastatic spread of disease following a primary diagnosis of melanoma (primary staging) or on clinical suspicion of disease recurrence (re‐staging). Accurate staging is crucial to ensuring that patients are directed to the most appropriate and effective treatment at different points on the clinical pathway. Establishing the comparative accuracy of ultrasound, computed tomography (CT), magnetic resonance imaging (MRI), and positron emission tomography (PET)‐CT imaging for detection of nodal or distant metastases, or both, is critical to understanding if, how, and where on the pathway these tests might be used. Objectives Primary objectives We estimated accuracy separately according to the point in the clinical pathway at which imaging tests were used. Our objectives were: • to determine the diagnostic accuracy of ultrasound or PET‐CT for detection of nodal metastases before sentinel lymph node biopsy in adults with confirmed cutaneous invasive melanoma; and • to determine the diagnostic accuracy of ultrasound, CT, MRI, or PET‐CT for whole body imaging in adults with cutaneous invasive melanoma: ○ for detection of any metastasis in adults with a primary diagnosis of melanoma (i.e. primary staging at presentation); and ○ for detection of any metastasis in adults undergoing staging of recurrence of melanoma (i.e. re‐staging prompted by findings on routine follow‐up). We undertook separate analyses according to whether accuracy data were reported per patient or per lesion. Secondary objectives We sought to determine the diagnostic accuracy of ultrasound, CT, MRI, or PET‐CT for whole body imaging (detection of any metastasis) in mixed or not clearly described populations of adults with cutaneous invasive melanoma. For study participants undergoing primary staging or re‐staging (for possible recurrence), and for mixed or unclear populations, our objectives were: • to determine the diagnostic accuracy of ultrasound, CT, MRI, or PET‐CT for detection of nodal metastases; • to determine the diagnostic accuracy of ultrasound, CT, MRI, or PET‐CT for detection of distant metastases; and • to determine the diagnostic accuracy of ultrasound, CT, MRI, or PET‐CT for detection of distant metastases according to metastatic site. Search methods We undertook a comprehensive search of the following databases from inception up to August 2016: Cochrane Central Register of Controlled Trials; MEDLINE; Embase; CINAHL; CPCI; Zetoc; Science Citation Index; US National Institutes of Health Ongoing Trials Register; NIHR Clinical Research Network Portfolio Database; and the World Health Organization International Clinical Trials Registry Platform. We studied reference lists as well as published systematic review articles. Selection criteria We included studies of any design that evaluated ultrasound (with or without the use of fine needle aspiration cytology (FNAC)), CT, MRI, or PET‐CT for staging of cutaneous melanoma in adults, compared with a reference standard of histological confirmation or imaging with clinical follow‐up of at least three months' duration. We excluded studies reporting multiple applications of the same test in more than 10% of study participants. Data collection and analysis Two review authors independently extracted all data using a standardised data extraction and quality assessment form (based on the Quality Assessment of Diagnostic Accuracy Studies 2 (QUADAS‐2)). We estimated accuracy using the bivariate hierarchical method to produce summary sensitivities and specificities with 95% confidence and prediction regions. We undertook analysis of studies allowing direct and indirect comparison between tests. We examined heterogeneity between studies by visually inspecting the forest plots of sensitivity and specificity and summary receiver operating characteristic (ROC) plots. Numbers of identified studies were insufficient to allow formal investigation of potential sources of heterogeneity. Main results We included a total of 39 publications reporting on 5204 study participants; 34 studies reporting data per patient included 4980 study participants with 1265 cases of metastatic disease, and seven studies reporting data per lesion included 417 study participants with 1846 potentially metastatic lesions, 1061 of which were confirmed metastases. The risk of bias was low or unclear for all domains apart from participant flow. Concerns regarding applicability of the evidence were high or unclear for almost all domains. Participant selection from mixed or not clearly defined populations and poorly described application and interpretation of index tests were particularly problematic. The accuracy of imaging for detection of regional nodal metastases before sentinel lymph node biopsy (SLNB) was evaluated in 18 studies. In 11 studies (2614 participants; 542 cases), the summary sensitivity of ultrasound alone was 35.4% (95% confidence interval (CI) 17.0% to 59.4%) and specificity was 93.9% (95% CI 86.1% to 97.5%). Combining pre‐SLNB ultrasound with FNAC revealed summary sensitivity of 18.0% (95% CI 3.58% to 56.5%) and specificity of 99.8% (95% CI 99.1% to 99.9%) (1164 participants; 259 cases). Four studies demonstrated lower sensitivity (10.2%, 95% CI 4.31% to 22.3%) and specificity (96.5%,95% CI 87.1% to 99.1%) for PET‐CT before SLNB (170 participants, 49 cases). When these data are translated to a hypothetical cohort of 1000 people eligible for SLNB, 237 of whom have nodal metastases (median prevalence), the combination of ultrasound with FNAC potentially allows 43 people with nodal metastases to be triaged directly to adjuvant therapy rather than having SLNB first, at a cost of two people with false positive results (who are incorrectly managed). Those with a false negative ultrasound will be identified on subsequent SLNB. Limited test accuracy data were available for whole body imaging via PET‐CT for primary staging or re‐staging for disease recurrence, and none evaluated MRI. Twenty‐four studies evaluated whole body imaging. Six of these studies explored primary staging following a confirmed diagnosis of melanoma (492 participants), three evaluated re‐staging of disease following some clinical indication of recurrence (589 participants), and 15 included mixed or not clearly described population groups comprising participants at a number of different points on the clinical pathway and at varying stages of disease (1265 participants). Results for whole body imaging could not be translated to a hypothetical cohort of people due to paucity of data. Most of the studies (6/9) of primary disease or re‐staging of disease considered PET‐CT, two in comparison to CT alone, and three studies examined the use of ultrasound. No eligible evaluations of MRI in these groups were identified. All studies used histological reference standards combined with follow‐up, and two included FNAC for some participants. Observed accuracy for detection of any metastases for PET‐CT was higher for re‐staging of disease (summary sensitivity from two studies: 92.6%, 95% CI 85.3% to 96.4%; specificity: 89.7%, 95% CI 78.8% to 95.3%; 153 participants; 95 cases) compared to primary staging (sensitivities from individual studies ranged from 30% to 47% and specificities from 73% to 88%), and was more sensitive than CT alone in both population groups, but participant numbers were very small. No conclusions can be drawn regarding routine imaging of the brain via MRI or CT. Authors' conclusions Review authors found a disappointing lack of evidence on the accuracy of imaging in people with a diagnosis of melanoma at different points on the clinical pathway. Studies were small and often reported data according to the number of lesions rather than the number of study participants. Imaging with ultrasound combined with FNAC before SLNB may identify around one‐fifth of those with nodal disease, but confidence intervals are wide and further work is needed to establish cost‐effectiveness. Much of the evidence for whole body imaging for primary staging or re‐staging of disease is focused on PET‐CT, and comparative data with CT or MRI are lacking. Future studies should go beyond diagnostic accuracy and consider the effects of different imaging tests on disease management. The increasing availability of adjuvant therapies for people with melanoma at high risk of disease spread at presentation will have a considerable impact on imaging services, yet evidence for the relative diagnostic accuracy of available tests is limited. Plain language summary How good are ultrasound, CT, MRI, and PET‐CT for identifying spread of disease in the body among people with melanoma? What is the aim of the review? We wanted to find out which imaging tests are better for identifying spread of disease among people with a first diagnosis of melanoma (primary staging) and among people with possible recurrence of melanoma (re‐staging). We looked at the evidence for ultrasound, CT, MRI, and PET‐CT and included 39 studies to answer these questions. Why are imaging tests for melanoma important? Melanoma is one of the most aggressive forms of skin cancer, with potential for metastases (cancer cells) to spread to the lymph nodes and other organs of the body. To make sure that people with melanoma receive the most appropriate and effective treatment, it is important to identify whether the disease has spread and to which parts of the body it has spread. This is called 'staging of disease'. Staging is done to find out if a melanoma has spread to regional lymph nodes or to lymph nodes close to the original melanoma, and to determine if the melanoma has spread to lymph nodes in other parts of the body or to organs of the body such as the liver or the brain (distant metastases). Imaging tests are tools that can be used to help find out how much the disease has spread. Several new treatments are now available for reducing the risk of spread of melanoma and for treating melanoma when it has spread. What was studied in the review? The review includes four imaging tests that create images of the body in different ways. Ultrasound uses high‐frequency sound waves to create images, CT scans use ionising radiation in the form of X‐rays (a very low dose of radiation), and MRI uses large magnets and non‐ionising radiation in the form of radio waves (which are not harmful) to generate images of the body. PET‐CT requires injection of a weakly radioactive substance (FDG). The PET part of the scan identifies areas of the body that take up a lot of FDG (indicating possibly cancerous cells), and the CT part of the scan helps to improve image quality and to more accurately pinpoint areas using more FDG. Ultrasound can also be performed along with a fairly simple procedure called 'fine needle aspiration cytology' (FNAC), by which a very fine needle is used to take a small sample of cells from a lymph node that looks suspicious on ultrasound. A microscope is then used to identify whether or not the cells are malignant. Imaging can be used at different time points after diagnosis of melanoma. Healthcare providers can use imaging to look at the regional lymph nodes closest to the melanoma before a type of surgery called sentinel lymph node biopsy is performed. Sentinel lymph node biopsy takes out the lymph nodes that are most likely to have metastases inside them so they can be tested in a laboratory. Imaging can also be used after sentinel lymph node biopsy or in people with higher‐risk melanoma to look for any spread of disease. Imaging can be used in people who were treated for melanoma at an earlier point and who might be having a recurrence of their disease. What are the main results of the review? Ultrasound of regional lymph nodes before sentinel lymph node biopsy We found 11 relevant studies including 2614 people. Three of these studies compared ultrasound on its own to ultrasound combined with FNAC. Results suggest that the combined procedure correctly identifies around one‐fifth of people with metastases in the lymph nodes with very few false positive results (people with incorrect diagnosis of metastasis). These results can be illustrated by imagining a group of 1000 people with melanoma who are going to have sentinel lymph node biopsy, of whom 237 (24%) have metastases in the lymph nodes. The combination of ultrasound with FNAC potentially allows 43 people with lymph node metastases to be identified and avoid a sentinel lymph node biopsy, at a cost of two people with false positive results who might go on to have the wrong treatment. Those with metastases in the lymph nodes that are missed on ultrasound (false negatives) will be identified on subsequent SLNB. Whole body imaging (detection of any metastases) We found 24 studies, but only nine were clear about the point in the time course of disease that imaging was carried out. Six studies including 492 people looked at imaging for primary staging following a confirmed diagnosis of melanoma, and three studies in 589 people evaluated re‐staging of disease in people with possible recurrence of disease. Most of the studies (6/9) considered PET‐CT, two in comparison to CT alone, and three studies examined the use of ultrasound. We did not find any suitable studies of MRI in these groups. Overall results suggest that PET‐CT is better for correctly identifying people with metastatic spread of disease who might be having a recurrence of disease (re‐staging) than people who have a new diagnosis of melanoma (primary staging). PET‐CT also seems to be better than CT for identifying spread of disease in both groups of people, but studies were very small and results might not be reliable. How reliable are the results of the studies included in this review? In most of our studies, a reliable diagnosis of spread of disease (or reference standard) was made by performing biopsy and by following up with people over time using clinical assessment and imaging. There was often a lack of detail on how patients were followed up and which tests were used. Lots of studies did not include people at clearly defined time points in the disease process, making it difficult to assess the relevance of their results. Reporting of application and interpretation of tests was poor. To whom do the results of this review apply? Thirty‐three studies were done in Europe (85%), and the rest in North America (n = 4), Asia (n = 1), or Oceania (n = 1). The average age of people in the studies was between 50 and 67 years, and around half were men. Studies mostly included people with melanoma on any part of the body, but two included only people with melanoma on the head or neck. Studies often included people at different stages of disease, and we were not able to look at the accuracy of tests for people at any particular disease stage. Studies were small, and their results might not match what happens in real life. What are the implications of this review? Reviewers found some evidence to support the use of imaging with ultrasound combined with FNAC before sentinel lymph node biopsy, but further work is needed to establish cost‐effectiveness. Limited evidence is available for whole body imaging for primary staging or re‐staging of disease. Available evidence is focused on PET‐CT; there are few comparisons with CT and no comparisons with MRI. Future research needs to look at more than test accuracy and must consider the effects of different imaging tests on treatment decisions for patients. How up‐to‐date is this review? The reviewers searched for and included studies published up to August 2016.* *In these studies, biopsy and clinical or imaging follow‐up were the reference standards (methods of establishing the final diagnosis).

UR - http://dx.doi.org/10.1002/14651858.CD012806.pub2

ER -

Record #9 of 32

Provider: John Wiley & Sons, Ltd

Content: text/plain; charset="UTF-8"

TY - JOUR

AN - CD012657

AU - Singh, JA

AU - Hossain, A

AU - Mudano, AS

AU - Tanjong Ghogomu, E

AU - Suarez‐Almazor, ME

AU - Buchbinder, R

AU - Maxwell, LJ

AU - Tugwell, P

AU - Wells, GA

TI - Biologics or tofacitinib for people with rheumatoid arthritis naive to methotrexate: a systematic review and network meta‐analysis

JF - Cochrane Database of Systematic Reviews

PY - 2017

IS - 5

PB - John Wiley & Sons, Ltd

SN - 1465-1858

KW - Abatacept [therapeutic use]

KW - Adalimumab [therapeutic use]

KW - Adult

KW - Antibodies, Monoclonal [therapeutic use]

KW - Antirheumatic Agents [*therapeutic use]

KW - Arthritis, Rheumatoid [*drug therapy]

KW - Bayes Theorem

KW - Biological Products [*therapeutic use]

KW - Etanercept [therapeutic use]

KW - Humans

KW - Infliximab [therapeutic use]

KW - Methotrexate [*therapeutic use]

KW - Methylprednisolone [therapeutic use]

KW - Network Meta‐Analysis

KW - Piperidines [*therapeutic use]

KW - Pyrimidines [*therapeutic use]

KW - Pyrroles [*therapeutic use]

KW - Randomized Controlled Trials as Topic

KW - Rituximab [therapeutic use]

N1 - [Musculoskeletal]

DO - 10.1002/14651858.CD012657

AB - Abstract - Background Biologic disease‐modifying anti‐rheumatic drugs (biologics) are highly effective in treating rheumatoid arthritis (RA), however there are few head‐to‐head biologic comparison studies. We performed a systematic review, a standard meta‐analysis and a network meta‐analysis (NMA) to update the 2009 Cochrane Overview. This review is focused on the adults with RA who are naive to methotrexate (MTX) that is, receiving their first disease‐modifying agent. Objectives To compare the benefits and harms of biologics (abatacept, adalimumab, anakinra, certolizumab pegol, etanercept, golimumab, infliximab, rituximab, tocilizumab) and small molecule tofacitinib versus comparator (methotrexate (MTX)/other DMARDs) in people with RA who are naive to methotrexate. Methods In June 2015 we searched for randomized controlled trials (RCTs) in CENTRAL, MEDLINE and Embase; and trials registers. We used standard Cochrane methods. We calculated odds ratios (OR) and mean differences (MD) along with 95% confidence intervals (CI) for traditional meta‐analyses and 95% credible intervals (CrI) using a Bayesian mixed treatment comparisons approach for network meta‐analysis (NMA). We converted OR to risk ratios (RR) for ease of interpretation. We also present results in absolute measures as risk difference (RD) and number needed to treat for an additional beneficial or harmful outcome (NNTB/H). Main results Nineteen RCTs with 6485 participants met inclusion criteria (including five studies from the original 2009 review), and data were available for four TNF biologics (adalimumab (six studies; 1851 participants), etanercept (three studies; 678 participants), golimumab (one study; 637 participants) and infliximab (seven studies; 1363 participants)) and two non‐TNF biologics (abatacept (one study; 509 participants) and rituximab (one study; 748 participants)). Less than 50% of the studies were judged to be at low risk of bias for allocation sequence generation, allocation concealment and blinding, 21% were at low risk for selective reporting, 53% had low risk of bias for attrition and 89% had low risk of bias for major baseline imbalance. Three trials used biologic monotherapy, that is, without MTX. There were no trials with placebo‐only comparators and no trials of tofacitinib. Trial duration ranged from 6 to 24 months. Half of the trials contained participants with early RA (less than two years' duration) and the other half included participants with established RA (2 to 10 years). Biologic + MTX versus active comparator (MTX ( 17 trials (6344 participants) /MTX + methylprednisolone 2 trials (141 participants) ) In traditional meta‐analyses, there was moderate‐quality evidence downgraded for inconsistency that biologics with MTX were associated with statistically significant and clinically meaningful benefit versus comparator as demonstrated by ACR50 (American College of Rheumatology scale) and RA remission rates. For ACR50, biologics with MTX showed a risk ratio (RR) of 1.40 (95% CI 1.30 to 1.49), absolute difference of 16% (95% CI 13% to 20%) and NNTB = 7 (95% CI 6 to 8). For RA remission rates, biologics with MTX showed a RR of 1.62 (95% CI 1.33 to 1.98), absolute difference of 15% (95% CI 11% to 19%) and NNTB = 5 (95% CI 6 to 7). Biologics with MTX were also associated with a statistically significant, but not clinically meaningful, benefit in physical function (moderate‐quality evidence downgraded for inconsistency), with an improvement of HAQ scores of ‐0.10 (95% CI ‐0.16 to ‐0.04 on a 0 to 3 scale), absolute difference ‐3.3% (95% CI ‐5.3% to ‐1.3%) and NNTB = 4 (95% CI 2 to 15). We did not observe evidence of differences between biologics with MTX compared to MTX for radiographic progression (low‐quality evidence, downgraded for imprecision and inconsistency) or serious adverse events (moderate‐quality evidence, downgraded for imprecision). Based on low‐quality evidence, results were inconclusive for withdrawals due to adverse events (RR of 1.32, but 95% confidence interval included possibility of important harm, 0.89 to 1.97). Results for cancer were also inconclusive (Peto OR 0.71, 95% CI 0.38 to 1.33) and downgraded to low‐quality evidence for serious imprecision. Biologic without MTX versus active comparator (MTX 3 trials (866 participants) There was no evidence of statistically significant or clinically important differences for ACR50, HAQ, remission, (moderate‐quality evidence for these benefits, downgraded for imprecision), withdrawals due to adverse events,and serious adverse events (low‐quality evidence for these harms, downgraded for serious imprecision). All studies were for TNF biologic monotherapy and none for non‐TNF biologic monotherapy. Radiographic progression was not measured. Authors' conclusions In MTX‐naive RA participants, there was moderate‐quality evidence that, compared with MTX alone, biologics with MTX was associated with absolute and relative clinically meaningful benefits in three of the efficacy outcomes (ACR50, HAQ scores, and RA remission rates). A benefit regarding less radiographic progression with biologics with MTX was not evident (low‐quality evidence). We found moderate‐ to low‐quality evidence that biologic therapy with MTX was not associated with any higher risk of serious adverse events compared with MTX, but results were inconclusive for withdrawals due to adverse events and cancer to 24 months. TNF biologic monotherapy did not differ statistically significantly or clinically meaningfully from MTX for any of the outcomes (moderate‐quality evidence), and no data were available for non‐TNF biologic monotherapy. We conclude that biologic with MTX use in MTX‐naive populations is beneficial and that there is little/inconclusive evidence of harms. More data are needed for tofacitinib, radiographic progression and harms in this patient population to fully assess comparative efficacy and safety. Plain language summary Biologics for rheumatoid arthritis (RA) in people not previously treated with methotrexate (MTX) Review question We studied the benefits and harms of biologics or tofacitinib on people with rheumatoid arthritis (RA) who have not previously been treated with methotrexate (MTX), in trials done until June 2015. Data was available for four TNF biologics (adalimumab, etanercept, golimumab, infliximab) and two non‐TNF biologics (abatacept, rituximab). What is RA and what are biologics/tofacitinib? In RA, the immune system, which normally fights infection, attacks the joint lining making it inflamed. Without treatment, the inflammation can lead to joint damage and disability. Biologics or tofacitinib are medications that can reduce joint inflammation/damage and improve symptoms. The review shows that in people with RA: ‐ Biologics (abatacept, adalimumab, etanercept, golimumab, infliximab, rituximab) in combination with MTX probably improve signs and symptoms of RA (tender or swollen joints), the chances of RA remission (disappearance of symptoms) and probably slightly improve functional ability. We downgraded our confidence in the results because of concerns about the inconsistency of some results. ‐ Biologics in combination with MTX may make little or no difference in the risk of serious adverse events or withdrawals due to adverse events. We downgraded our confidence in the results because of concerns about the inconsistency of some results and the lack of data. ‐ We often do not have precise information about side effects and complications. Because of the lack of data, we are uncertain of the effect of biologics on the risk of cancer. ‐TNF biologics (adalimumab, etanercept, golimumab) alone (not in combination with MTX) probably make little or no difference in signs and symptoms of RA or chances of RA remission (no data for non‐TNF biologics alone). Best estimate of what happens to people with RA when taking biologics: ACR50 (American College of Rheumatology 50: number of tender or swollen joints, pain and disability) : Biologic + MTX versus MTX: 56 people out of 100 who were on a biologic (in combination with MTX) experienced improvement in RA compared to 40 people out of 100 who were on MTX (16% improvement). Biologic monotherapy (TNF biologics) versus MTX: 35 people out of 100 who were on a biologic experienced improvement in RA compared to 37 people out of 100 who were on MTX (2% reduction) Remission (DAS <1.6 or DAS28 < 2.6) Biologic + MTX versus MTX: 37 people out of 100 who took a biologic (in combination with MTX) had their RA symptoms disappear compared to 22 people out of 100 who were on MTX (15% improvement). Biologic monotherapy (TNF biologics) versus MTX: 22 people out of 100 who took a biologic had their RA symptoms disappear compared to 20 people out of 100 who were on MTX (2% improvement). Progression of disease damage as measured on X‐rays (scale 0 to 448) Biologic + MTX versus MTX: people who took a biologic (in combination with MTX) showed radiographic progression of 0.45 points compared to those on MTX who showed progression of 3 points (0.5% reduction). There were no studies for biologic monotherapy. Drug withdrawal due to adverse events Biologic + MTX versus MTX: 7 people out of 100 who took a biologic (in combination with MTX) withdrew from the study due to adverse events compared to 5 out of 100 participants who took MTX (2% more). Biologic monotherapy (TNF biologics) versus MTX: 6 people out of 100 who took a biologic withdrew from the study due to adverse events compared to 6 out of 100 participants who took MTX (0% difference). Serious adverse events Biologic + MTX versus MTX: 11 participants out of 100 who took a biologic (in combination with MTX) reported serious adverse events compared to 10 participants out of 100 on MTX (1% more serious adverse events). Biologic monotherapy (TNF biologics) versus MTX: 3 participants out of 100 who took a biologic reported serious adverse events compared to 7 participants out of 100 on MTX (4% fewer serious adverse events). Cancer The same number of people (1 out of 100) reported cancer for biologic (both alone and in combination with MTX) and the comparator MTX. However, there were few events of cancer so caution in this interpretation is needed.

UR - http://dx.doi.org/10.1002/14651858.CD012657

ER -

Record #10 of 32

Provider: John Wiley & Sons, Ltd

Content: text/plain; charset="UTF-8"

TY - JOUR

AN - CD012591

AU - Singh, JA

AU - Hossain, A

AU - Tanjong Ghogomu, E

AU - Mudano, AS

AU - Maxwell, LJ

AU - Buchbinder, R

AU - Lopez‐Olivo, MA

AU - Suarez‐Almazor, ME

AU - Tugwell, P

AU - Wells, GA

TI - Biologics or tofacitinib for people with rheumatoid arthritis unsuccessfully treated with biologics: a systematic review and network meta‐analysis

JF - Cochrane Database of Systematic Reviews

PY - 2017

IS - 3

PB - John Wiley & Sons, Ltd

SN - 1465-1858

KW - Antirheumatic Agents [adverse effects, *therapeutic use]

KW - Arthritis, Rheumatoid [diagnostic imaging, *therapy]

KW - Bayes Theorem

KW - Biological Products [adverse effects, *therapeutic use]

KW - Disease Progression

KW - Humans

KW - Methotrexate [therapeutic use]

KW - Neoplasms [etiology]

KW - Network Meta‐Analysis

KW - Piperidines [adverse effects, *therapeutic use]

KW - Protein Kinase Inhibitors [adverse effects, *therapeutic use]

KW - Pyrimidines [adverse effects, *therapeutic use]

KW - Pyrroles [adverse effects, *therapeutic use]

KW - Treatment Failure

N1 - [Musculoskeletal]

DO - 10.1002/14651858.CD012591

AB - Abstract - Background Biologic disease‐modifying anti‐rheumatic drugs (DMARDs: referred to as biologics) are effective in treating rheumatoid arthritis (RA), however there are few head‐to‐head comparison studies. Our systematic review, standard meta‐analysis and network meta‐analysis (NMA) updates the 2009 Cochrane overview, 'Biologics for rheumatoid arthritis (RA)' and adds new data. This review is focused on biologic or tofacitinib therapy in people with RA who had previously been treated unsuccessfully with biologics. Objectives To compare the benefits and harms of biologics (abatacept, adalimumab, anakinra, certolizumab pegol, etanercept, golimumab, infliximab, rituximab, tocilizumab) and small molecule tofacitinib versus comparator (placebo or methotrexate (MTX)/other DMARDs) in people with RA, previously unsuccessfully treated with biologics. Methods On 22 June 2015 we searched for randomized controlled trials (RCTs) in CENTRAL, MEDLINE, and Embase; and trials registries (WHO trials register, Clinicaltrials.gov). We carried out article selection, data extraction, and risk of bias and GRADE assessments in duplicate. We calculated direct estimates with 95% confidence intervals (CI) using standard meta‐analysis. We used a Bayesian mixed treatment comparison (MTC) approach for NMA estimates with 95% credible intervals (CrI). We converted odds ratios (OR) to risk ratios (RR) for ease of understanding. We have also presented results in absolute measures as risk difference (RD) and number needed to treat for an additional beneficial outcome (NNTB). Outcomes measured included four benefits (ACR50, function measured by Health Assessment Questionnaire (HAQ) score, remission defined as DAS < 1.6 or DAS28 < 2.6, slowing of radiographic progression) and three harms (withdrawals due to adverse events, serious adverse events, and cancer). Main results This update includes nine new RCTs for a total of 12 RCTs that included 3364 participants. The comparator was placebo only in three RCTs (548 participants), MTX or other traditional DMARD in six RCTs (2468 participants), and another biologic in three RCTs (348 participants). Data were available for four tumor necrosis factor (TNF)‐biologics: (certolizumab pegol (1 study; 37 participants), etanercept (3 studies; 348 participants), golimumab (1 study; 461 participants), infliximab (1 study; 27 participants)), three non‐TNF biologics (abatacept (3 studies; 632 participants), rituximab (2 studies; 1019 participants), and tocilizumab (2 studies; 589 participants)); there was only one study for tofacitinib (399 participants). The majority of the trials (10/12) lasted less than 12 months. We judged 33% of the studies at low risk of bias for allocation sequence generation, allocation concealment and blinding, 25% had low risk of bias for attrition, 92% were at unclear risk for selective reporting; and 92% had low risk of bias for major baseline imbalance. We downgraded the quality of the evidence for most outcomes to moderate or low due to study limitations, heterogeneity, or rarity of direct comparator trials. Biologic monotherapy versus placebo Compared to placebo, biologics were associated with clinically meaningful and statistically significant improvement in RA as demonstrated by higher ACR50 and RA remission rates. RR was 4.10 for ACR50 (95% CI 1.97 to 8.55; moderate‐quality evidence); absolute benefit RD 14% (95% CI 6% to 21%); and NNTB = 8 (95% CI 4 to 23). RR for RA remission was 13.51 (95% CI 1.85 to 98.45, one study available; moderate‐quality evidence); absolute benefit RD 9% (95% CI 5% to 13%); and NNTB = 11 (95% CI 3 to 136). Results for withdrawals due to adverse events and serious adverse events did not show any statistically significant or clinically meaningful differences. There were no studies available for analysis for function measured by HAQ, radiographic progression, or cancer outcomes. There were not enough data for any of the outcomes to look at subgroups. Biologic + MTX versus active comparator (MTX/other traditional DMARDs) Compared to MTX/other traditional DMARDs, biologic + MTX was associated with a clinically meaningful and statistically significant improvement in ACR50, function measured by HAQ, and RA remission rates in direct comparisons. RR for ACR50 was 4.07 (95% CI 2.76 to 5.99; high‐quality evidence); absolute benefit RD 16% (10% to 21%); NNTB = 7 (95% CI 5 to 11). HAQ scores showed an improvement with a mean difference (MD) of 0.29 (95% CI 0.21 to 0.36; high‐quality evidence); absolute benefit RD 9.7% improvement (95% CI 7% to 12%); and NNTB = 5 (95% CI 4 to 7). Remission rates showed an improved RR of 20.73 (95% CI 4.13 to 104.16; moderate‐quality evidence); absolute benefit RD 10% (95% CI 8% to 13%); and NNTB = 17 (95% CI 4 to 96), among the biologic + MTX group compared to MTX/other DMARDs. There were no studies for radiographic progression. Results were not clinically meaningful or statistically significantly different for withdrawals due to adverse events or serious adverse events, and were inconclusive for cancer. Tofacitinib monotherapy versus placebo There were no published data. Tofacitinib + MTX versus active comparator (MTX) In one study, compared to MTX, tofacitinib + MTX was associated with a clinically meaningful and statistically significant improvement in ACR50 (RR 3.24; 95% CI 1.78 to 5.89; absolute benefit RD 19% (95% CI 12% to 26%); NNTB = 6 (95% CI 3 to 14); moderate‐quality evidence), and function measured by HAQ, MD 0.27 improvement (95% CI 0.14 to 0.39); absolute benefit RD 9% (95% CI 4.7% to 13%), NNTB = 5 (95% CI 4 to 10); high‐quality evidence). RA remission rates were not statistically significantly different but the observed difference may be clinically meaningful (RR 15.44 (95% CI 0.93 to 256.1; high‐quality evidence); absolute benefit RD 6% (95% CI 3% to 9%); NNTB could not be calculated. There were no studies for radiographic progression. There were no statistically significant or clinically meaningful differences for withdrawals due to adverse events and serious adverse events, and results were inconclusive for cancer. Authors' conclusions Biologic (with or without MTX) or tofacitinib (with MTX) use was associated with clinically meaningful and statistically significant benefits (ACR50, HAQ, remission) compared to placebo or an active comparator (MTX/other traditional DMARDs) among people with RA previously unsuccessfully treated with biologics. No studies examined radiographic progression. Results were not clinically meaningful or statistically significant for withdrawals due to adverse events and serious adverse events, and were inconclusive for cancer. Plain language summary Biologics or tofacitinib for rheumatoid arthritis Review question We studied the effects of biologics on people with rheumtoid arthritis (RA), whose previous treatment with biologic therapy was unsuccessful, either due to lack of benefits or occurrence of side effects, or both. There were a total of 12 studies (up to June 2015) with data available for four of the tumor necrosis factor (TNF)‐biologics (certolizumab pegol, etanercept, golimumab, infliximab) and three of the non‐TNF biologics (abatacept, rituximab, and tocilizumab); only one study provided data for tofacitinib. What is RA and what are biologics/tofacitinib? In RA, your immune system, which normally fights infection, attacks the joint lining making it inflamed. If the inflammation is untreated, joint damage and disability may result. Biologics and tofacitinib are medications that can reduce joint inflammation, improve symptoms and prevent some of the joint damage. The review shows that in people with RA: ‐ Biologics alone or in combination with methotrexate (MTX), a disease‐modifying anti‐rheumatic drug (DMARD), improve signs (tender or swollen joints) and symptoms of RA, function, and probably improve the chances of RA remission (disappearance of symptoms), based on high‐ and moderate‐quality evidence (downgraded for imprecision). ‐ Tofacitinib in combination with MTX, probably improves signs and symptoms of RA (tender or swollen joints) and function, based on high‐ and moderate‐quality evidence (downgraded for imprecision). ‐ We often do not have precise information about side‐effects and complications. This is particularly true for rare but serious side‐effects. Because of the lack of data and low‐quality evidence, we are uncertain of the effect of biologics and tofacitinib on the risk of cancer, serious adverse events, and withdrawals due to adverse events. Best estimate of what happens to people with RA when taking biologics or tofacitinib: ACR50 (number of tender or swollen joints, pain, and disability) Biologic monotherapy versus placebo : 18 out of 100 people on a biologic monotherapy experienced improvement in their symptoms versus 4 out of 100 on placebo (14% absolute improvement). Biologic + MTX versus MTX/other traditional DMARDs : 21 people out of 100 on biologic + MTX experienced improvement in RA symptoms compared to 5 people out of 100 who were on MTX/DMARD (16% absolute improvement). Tofacitinib + MTX versus MTX : 28 people out of 100 on tofacitinib + MTX experienced improvement in RA symptoms compared to 9 people out of 100 who were on MTX/DMARD (19% absolute improvement), based on one study. Remission (DAS < 1.6 or DAS28 < 2.6) Biologic monotherapy versus placebo : 102 people out of 1000 who were on biologic had their RA symptoms disappear compared to 8 people out of 1000 on placebo (9% absolute improvement). Biologic + MTX versus MTX/other traditional DMARDs: 104 people out of 1000 who were on biologic + MTX had their RA symptoms disappear compared to 3 people out of 1000 who were on MTX/DMARD (10% absolute improvement). Tofacitinib + MTX versus MTX : 56 people out of 1000 who were on tofacitinib + MTX had their RA symptoms disappear compared to 0 people out of 1000 who were on MTX/DMARD (6% absolute improvement), based on one study. Progression of radiographic destruction No studies were available for analysis. Drug withdrawal due to adverse events Biologic monotherapy versus placebo : 32 people out of 1000 on biologic reported withdrawal due to adverse events versus 42 out of 1000 on placebo (1% fewer withdrawals). Biologic + MTX versus MTX/other traditional DMARDs : 38 people out of 1000 on biologic + MTX reported withdrawal due to adverse events compared to 8 out of 1000 people on MTX/DMARD (5% more withdrawals). Tofacitinib + MTX versus MTX : there was no difference in withdrawals due to adverse events between people on tofacitinib + MTX and people on MTX/DMARD, both with 5 participants out of 100, based on one study. Serious a dverse events There was a 1% to 3% difference for fewer serious adverse events in all comparisons compared with people on MTX/DMARD. Cancer Biologic + MTX versus MTX/other traditional DMARDs : there was a less than 1% difference for the risk of cancer between biologic + MTX and MTX/DMARD; 5 out of 1000 of those on biologic + MTX and 0 on MTX/DMARD developed cancer, although there were very few studies available.

UR - http://dx.doi.org/10.1002/14651858.CD012591

ER -

Record #11 of 32

Provider: John Wiley & Sons, Ltd

Content: text/plain; charset="UTF-8"

TY - JOUR

AN - CD004720

AU - Ilic, D

AU - Neuberger, MM

AU - Djulbegovic, M

AU - Dahm, P

TI - Screening for prostate cancer

JF - Cochrane Database of Systematic Reviews

PY - 2013

IS - 1

PB - John Wiley & Sons, Ltd

SN - 1465-1858

KW - Aged

KW - Aged, 80 and over

KW - Biopsy, Fine-Needle [adverse effects]

KW - Digital Rectal Examination [*methods]

KW - Endoscopic Ultrasound-Guided Fine Needle Aspiration [methods]

KW - Humans

KW - Male

KW - Mass Screening [*methods, statistics & numerical data]

KW - Middle Aged

KW - Prostate [pathology]

KW - Prostate-Specific Antigen [*blood]

KW - Prostatic Neoplasms [*diagnosis, *mortality]

KW - Randomized Controlled Trials as Topic

N1 - [Urology]

DO - 10.1002/14651858.CD004720.pub3

AB - Abstract - Background Any form of screening aims to reduce disease‐specific and overall mortality, and to improve a person's future quality of life. Screening for prostate cancer has generated considerable debate within the medical and broader community, as demonstrated by the varying recommendations made by medical organizations and governed by national policies. To better inform individual patient decision‐making and health policy decisions, we need to consider the entire body of data from randomised controlled trials (RCTs) on prostate cancer screening summarised in a systematic review. In 2006, our Cochrane review identified insufficient evidence to either support or refute the use of routine mass, selective, or opportunistic screening for prostate cancer. An update of the review in 2010 included three additional trials. Meta‐analysis of the five studies included in the 2010 review concluded that screening did not significantly reduce prostate cancer‐specific mortality. In the past two years, several updates to studies included in the 2010 review have been published thereby providing the rationale for this update of the 2010 systematic review. Objectives To determine whether screening for prostate cancer reduces prostate cancer‐specific mortality or all‐cause mortality and to assess its impact on quality of life and adverse events. Search methods An updated search of electronic databases (PROSTATE register, the Cochrane Central Register of Controlled Trials (CENTRAL), MEDLINE, EMBASE, CANCERLIT, and the NHS EED) was performed, in addition to handsearching of specific journals and bibliographies, in an effort to identify both published and unpublished trials. Selection criteria All RCTs of screening versus no screening for prostate cancer were eligible for inclusion in this review. Data collection and analysis The original search (2006) identified 99 potentially relevant articles that were selected for full‐text review. From these citations, two RCTs were identified as meeting the inclusion criteria. The search for the 2010 version of the review identified a further 106 potentially relevant articles, from which three new RCTs were included in the review. A total of 31 articles were retrieved for full‐text examination based on the updated search in 2012. Updated data on three studies were included in this review. Data from the trials were independently extracted by two authors. Main results Five RCTs with a total of 341,342 participants were included in this review. All involved prostate‐specific antigen (PSA) testing, with or without digital rectal examination (DRE), though the interval and threshold for further evaluation varied across trials. The age of participants ranged from 45 to 80 years and duration of follow‐up from 7 to 20 years. Our meta‐analysis of the five included studies indicated no statistically significant difference in prostate cancer‐specific mortality between men randomised to the screening and control groups (risk ratio (RR) 1.00, 95% confidence interval (CI) 0.86 to 1.17). The methodological quality of three of the studies was assessed as posing a high risk of bias. The European Randomized Study of Screening for Prostate Cancer (ERSPC) and the US Prostate, Lung, Colorectal and Ovarian (PLCO) Cancer Screening Trial were assessed as posing a low risk of bias, but provided contradicting results. The ERSPC study reported a significant reduction in prostate cancer‐specific mortality (RR 0.84, 95% CI 0.73 to 0.95), whilst the PLCO study concluded no significant benefit (RR 1.15, 95% CI 0.86 to 1.54). The ERSPC was the only study of the five included in this review that reported a significant reduction in prostate cancer‐specific mortality, in a pre‐specified subgroup of men aged 55 to 69 years of age. Sensitivity analysis for overall risk of bias indicated no significant difference in prostate cancer‐specific mortality when referring to the meta analysis of only the ERSPC and PLCO trial data (RR 0.96, 95% CI 0.70 to 1.30). Subgroup analyses indicated that prostate cancer‐specific mortality was not affected by the age at which participants were screened. Meta‐analysis of four studies investigating all‐cause mortality did not determine any significant differences between men randomised to screening or control (RR 1.00, 95% CI 0.96 to 1.03). A diagnosis of prostate cancer was significantly greater in men randomised to screening compared to those randomised to control (RR 1.30, 95% CI 1.02 to 1.65). Localised prostate cancer was more commonly diagnosed in men randomised to screening (RR 1.79, 95% CI 1.19 to 2.70), whilst the proportion of men diagnosed with advanced prostate cancer was significantly lower in the screening group compared to the men serving as controls (RR 0.80, 95% CI 0.73 to 0.87). Screening resulted in a range of harms that can be considered minor to major in severity and duration. Common minor harms from screening include bleeding, bruising and short‐term anxiety. Common major harms include overdiagnosis and overtreatment, including infection, blood loss requiring transfusion, pneumonia, erectile dysfunction, and incontinence. Harms of screening included false‐positive results for the PSA test and overdiagnosis (up to 50% in the ERSPC study). Adverse events associated with transrectal ultrasound (TRUS)‐guided biopsies included infection, bleeding and pain. No deaths were attributed to any biopsy procedure. None of the studies provided detailed assessment of the effect of screening on quality of life or provided a comprehensive assessment of resource utilization associated with screening (although preliminary analyses were reported). Authors' conclusions Prostate cancer screening did not significantly decrease prostate cancer‐specific mortality in a combined meta‐analysis of five RCTs. Only one study (ERSPC) reported a 21% significant reduction of prostate cancer‐specific mortality in a pre‐specified subgroup of men aged 55 to 69 years. Pooled data currently demonstrates no significant reduction in prostate cancer‐specific and overall mortality. Harms associated with PSA‐based screening and subsequent diagnostic evaluations are frequent, and moderate in severity. Overdiagnosis and overtreatment are common and are associated with treatment‐related harms. Men should be informed of this and the demonstrated adverse effects when they are deciding whether or not to undertake screening for prostate cancer. Any reduction in prostate cancer‐specific mortality may take up to 10 years to accrue; therefore, men who have a life expectancy less than 10 to 15 years should be informed that screening for prostate cancer is unlikely to be beneficial. No studies examined the independent role of screening by DRE. Plain language summary Screening for prostate cancer Prostate cancer is one of the most prevalent forms of cancer in men worldwide. Screening for prostate cancer implies that diagnostic tests be performed in the absence of any symptoms or indications of disease. These tests include the digital rectal examination (DRE), the prostate‐specific antigen (PSA) blood test and transrectal ultrasound (TRUS) guided biopsy. Screening aims to identify cancers at an early and treatable stage, therefore increasing the chances of successful treatment while also improving a patient's future quality of life. This review identified five relevant studies, comprised of 341,342 participants in total. Two of the studies were assessed to be of low risk of bias, whilst the remaining three had more substantive methodological weaknesses. Meta‐analysis of all five included studies demonstrated no statistically significant reduction in prostate cancer‐specific mortality (risk ratio (RR) 1.00, 95% confidence interval (CI) 0.86 to 1.17). Meta‐analysis of the two low risk of bias studies indicated no significant reduction in prostate cancer‐specific mortality (RR 0.96, 95% CI 0.70 to 1.30). Only one study included in this review (ERSPC) reported a significant 21% relative reduction (95% CI 31% to 8%) in prostate cancer‐specific mortality in a pre‐specified subgroup of men. These results were primarily driven by two countries within the ERSPC study that had very high prostate cancer mortality rates and unusually large reduction estimates. Among men aged 55 to 69 years in the ERSPC study, the study authors reported that 1055 men would need to be screened to prevent one additional death from prostate cancer during a median follow‐up duration of 11 years. Harms included overdiagnosis and harms associated with overtreatment, including false‐positive results for the PSA test, infection, bleeding, and pain associated with subsequent biopsy.

UR - http://dx.doi.org/10.1002/14651858.CD004720.pub3

ER -

Record #12 of 32

Provider: John Wiley & Sons, Ltd

Content: text/plain; charset="UTF-8"

TY - JOUR

AN - CD000011

AU - Nieuwlaat, R

AU - Wilczynski, N

AU - Navarro, T

AU - Hobson, N

AU - Jeffery, R

AU - Keepanasseril, A

AU - Agoritsas, T

AU - Mistry, N

AU - Iorio, A

AU - Jack, S

AU - et al.

TI - Interventions for enhancing medication adherence

JF - Cochrane Database of Systematic Reviews

PY - 2014

IS - 11

PB - John Wiley & Sons, Ltd

SN - 1465-1858

KW - *Drug Therapy

KW - *Medication Adherence

KW - Humans

KW - Patient Education as Topic

KW - Publication Bias

KW - Randomized Controlled Trials as Topic

KW - Self Administration

N1 - [Consumers and Communication]

DO - 10.1002/14651858.CD000011.pub4

AB - Abstract - Background People who are prescribed self administered medications typically take only about half their prescribed doses. Efforts to assist patients with adherence to medications might improve the benefits of prescribed medications. Objectives The primary objective of this review is to assess the effects of interventions intended to enhance patient adherence to prescribed medications for medical conditions, on both medication adherence and clinical outcomes. Search methods We updated searches of The Cochrane Library , including CENTRAL (via http://onlinelibrary.wiley.com/cochranelibrary/search/), MEDLINE, EMBASE, PsycINFO (all via Ovid), CINAHL (via EBSCO), and Sociological Abstracts (via ProQuest) on 11 January 2013 with no language restriction. We also reviewed bibliographies in articles on patient adherence, and contacted authors of relevant original and review articles. Selection criteria We included unconfounded RCTs of interventions to improve adherence with prescribed medications, measuring both medication adherence and clinical outcome, with at least 80% follow‐up of each group studied and, for long‐term treatments, at least six months follow‐up for studies with positive findings at earlier time points. Data collection and analysis Two review authors independently extracted all data and a third author resolved disagreements. The studies differed widely according to medical condition, patient population, intervention, measures of adherence, and clinical outcomes. Pooling results according to one of these characteristics still leaves highly heterogeneous groups, and we could not justify meta‐analysis. Instead, we conducted a qualitative analysis with a focus on the RCTs with the lowest risk of bias for study design and the primary clinical outcome. Main results The present update included 109 new RCTs published since the previous update in January 2007, bringing the total number of RCTs to 182; we found five RCTs from the previous update to be ineligible and excluded them. Studies were heterogeneous for patients, medical problems, treatment regimens, adherence interventions, and adherence and clinical outcome measurements, and most had high risk of bias. The main changes in comparison with the previous update include that we now: 1) report a lack of convincing evidence also specifically among the studies with the lowest risk of bias; 2) do not try to classify studies according to intervention type any more, due to the large heterogeneity; 3) make our database available for collaboration on sub‐analyses, in acknowledgement of the need to make collective advancement in this difficult field of research. Of all 182 RCTs, 17 had the lowest risk of bias for study design features and their primary clinical outcome, 11 from the present update and six from the previous update. The RCTs at lowest risk of bias generally involved complex interventions with multiple components, trying to overcome barriers to adherence by means of tailored ongoing support from allied health professionals such as pharmacists, who often delivered intense education, counseling (including motivational interviewing or cognitive behavioral therapy by professionals) or daily treatment support (or both), and sometimes additional support from family or peers. Only five of these RCTs reported improvements in both adherence and clinical outcomes, and no common intervention characteristics were apparent. Even the most effective interventions did not lead to large improvements in adherence or clinical outcomes. Authors' conclusions Across the body of evidence, effects were inconsistent from study to study, and only a minority of lowest risk of bias RCTs improved both adherence and clinical outcomes. Current methods of improving medication adherence for chronic health problems are mostly complex and not very effective, so that the full benefits of treatment cannot be realized. The research in this field needs advances, including improved design of feasible long‐term interventions, objective adherence measures, and sufficient study power to detect improvements in patient‐important clinical outcomes. By making our comprehensive database available for sharing we hope to contribute to achieving these advances. Plain language summary Ways to help people follow prescribed medicines Background Patients who are prescribed medicines take only about half of their doses and many stop treatment entirely. Assisting patients to adhere better to medicines could improve their health, and many studies have tested ways to achieve this. Question We updated our review from 2007 to answer the question: What are the findings of high‐quality studies that tested ways to assist patients with adhering to their medicines? Search strategy We retrieved studies published until 11 January 2013. To find relevant studies we searched six online databases and references in other reviews, and we contacted authors of relevant studies and reviews. Selection criteria We selected studies reporting a randomized controlled trial (RCT) comparing a group receiving an intervention to improve medicine adherence with a group not receiving the intervention. We included trials if they measured both medicine adherence and a clinical outcome (e.g. blood pressure), with at least 80% of patients studied until the end. Main results The studies differed widely regarding included patients, treatments, adherence intervention types, medicine adherence measurement, and clinical outcomes. Therefore, we could not combine the results in statistical analysis to reach general conclusions, as it would be misleading to suggest that they are comparable. Instead, we provide the key features and findings of each study in tables, and we describe intervention effects in studies of the highest quality. The present update included 109 new studies, bringing the total number to 182. In the 17 studies of the highest quality, interventions were generally complex with several different ways to try to improve medicine adherence. These frequently included enhanced support from family, peers, or allied health professionals such as pharmacists, who often delivered education, counseling, or daily treatment support. Only five of these RCTs improved both medicine adherence and clinical outcomes, and no common characteristics for their success could be identified. Overall, even the most effective interventions did not lead to large improvements. Authors' conclusions Characteristics and effects of interventions to improve medicine adherence varied among studies. It is uncertain how medicine adherence can consistently be improved so that the full health benefits of medicines can be realized. We need more advanced methods for researching ways to improve medicine adherence, including better interventions, better ways of measuring adherence, and studies that include sufficient patients to draw conclusions on clinically important effects.

UR - http://dx.doi.org/10.1002/14651858.CD000011.pub4

ER -

Record #13 of 32

Provider: John Wiley & Sons, Ltd

Content: text/plain; charset="UTF-8"

TY - JOUR

AN - CD013020

AU - Jakob, T

AU - Tesfamariam, YM

AU - Macherey, S

AU - Kuhr, K

AU - Adams, A

AU - Monsef, I

AU - Heidenreich, A

AU - Skoetz, N

TI - Bisphosphonates or RANK‐ligand‐inhibitors for men with prostate cancer and bone metastases: a network meta‐analysis

JF - Cochrane Database of Systematic Reviews

PY - 2020

IS - 12

PB - John Wiley & Sons, Ltd

SN - 1465-1858

KW - Adult

KW - Alendronate [adverse effects, therapeutic use]

KW - Antineoplastic Agents, Hormonal [therapeutic use]

KW - Bisphosphonate-Associated Osteonecrosis of the Jaw [etiology]

KW - Bone Density Conservation Agents [adverse effects, *therapeutic use]

KW - Bone Neoplasms [*drug therapy, *secondary]

KW - Clodronic Acid [adverse effects, therapeutic use]

KW - Denosumab [adverse effects, *therapeutic use]

KW - Diphosphonates [adverse effects, *therapeutic use]

KW - Etidronic Acid [adverse effects, therapeutic use]

KW - Humans

KW - Male

KW - Network Meta-Analysis

KW - Pamidronate [adverse effects, therapeutic use]

KW - Prostatic Neoplasms [drug therapy, *pathology]

KW - Prostatic Neoplasms, Castration-Resistant [pathology]

KW - Quality of Life

KW - RANK Ligand [*antagonists & inhibitors]

KW - Randomized Controlled Trials as Topic

KW - Risedronic Acid [adverse effects, therapeutic use]

KW - Zoledronic Acid [adverse effects, therapeutic use]

N1 - [Urology]

DO - 10.1002/14651858.CD013020.pub2

AB - Abstract - Background Different bone‐modifying agents like bisphosphonates and receptor activator of nuclear factor‐kappa B ligand (RANKL)‐inhibitors are used as supportive treatment in men with prostate cancer and bone metastases to prevent skeletal‐related events (SREs). SREs such as pathologic fractures, spinal cord compression, surgery and radiotherapy to the bone, and hypercalcemia lead to morbidity, a poor performance status, and impaired quality of life. Efficacy and acceptability of the bone‐targeted therapy is therefore of high relevance. Until now recommendations in guidelines on which bone‐modifying agents should be used are rare and inconsistent. Objectives To assess the effects of bisphosphonates and RANKL‐inhibitors as supportive treatment for prostate cancer patients with bone metastases and to generate a clinically meaningful treatment ranking according to their safety and efficacy using network meta‐analysis. Search methods We identified studies by electronically searching the bibliographic databases Cochrane Controlled Register of Trials (CENTRAL), MEDLINE, and Embase until 23 March 2020. We searched the Cochrane Library and various trial registries and screened abstracts of conference proceedings and reference lists of identified trials. Selection criteria We included randomized controlled trials comparing different bisphosphonates and RANKL‐inihibitors with each other or against no further treatment or placebo for men with prostate cancer and bone metastases. We included men with castration‐restrictive and castration‐sensitive prostate cancer and conducted subgroup analyses according to this criteria. Data collection and analysis Two review authors independently extracted data and assessed the quality of trials. We defined proportion of participants with pain response and the adverse events renal impairment and osteonecrosis of the jaw (ONJ) as the primary outcomes. Secondary outcomes were SREs in total and each separately (see above), mortality, quality of life, and further adverse events such as grade 3 to 4 adverse events, hypocalcemia, fatigue, diarrhea, and nausea. We conducted network meta‐analysis and generated treatment rankings for all outcomes, except quality of life due to insufficient reporting on this outcome. We compiled ranking plots to compare single outcomes of efficacy against outcomes of acceptability of the bone‐modifying agents. We assessed the certainty of the evidence for the main outcomes using the GRADE approach. Main results Twenty‐five trials fulfilled our inclusion criteria. Twenty‐one trials could be considered in the quantitative analysis, of which six bisphosphonates (zoledronic acid, risedronate, pamidronate, alendronate, etidronate, or clodronate) were compared with each other, the RANKL‐inhibitor denosumab, or no treatment/placebo. By conducting network meta‐analysis we were able to compare all of these reported agents directly and/or indirectly within the network for each outcome. In the abstract only the comparisons of zoledronic acid and denosumab against the main comparator (no treatment/placebo) are described for outcomes that were predefined as most relevant and that also appear in the 'Summary of findings' table. Other results, as well as results of subgroup analyses regarding castration status of participants, are displayed in the Results section of the full text. Treatment with zoledronic acid probably neither reduces nor increases the proportion of participants with pain response when compared to no treatment/placebo (risk ratio (RR) 1.46, 95% confidence interval (CI) 0.93 to 2.32; per 1000 participants 121 more (19 less to 349 more); moderate‐certainty evidence; network based on 4 trials including 1013 participants). For this outcome none of the trials reported results for the comparison with denosumab. The adverse event renal impairment probably occurs more often when treated with zoledronic acid compared to no treatment/placebo (RR 1.63, 95% CI 1.08 to 2.45; per 1000 participants 78 more (10 more to 180 more); moderate‐certainty evidence; network based on 6 trials including 1769 participants). Results for denosumab could not be included for this outcome, since zero events cannot be considered in the network meta‐analysis, therefore it does not appear in the ranking. Treatment with denosumab results in increased occurrence of the adverse event ONJ (RR 3.45, 95% CI 1.06 to 11.24; per 1000 participants 30 more (1 more to 125 more); high‐certainty evidence; 4 trials, 3006 participants) compared to no treatment/placebo. When comparing zoledronic acid to no treatment/placebo, the confidence intervals include the possibility of benefit or harm, therefore treatment with zoledronic acid probably neither reduces nor increases ONJ (RR 1.88, 95% CI 0.73 to 4.87; per 1000 participants 11 more (3 less to 47 more); moderate‐certainty evidence; network based on 4 trials including 3006 participants). Compared to no treatment/placebo, treatment with zoledronic acid (RR 0.84, 95% CI 0.72 to 0.97) and denosumab (RR 0.72, 95% CI 0.54 to 0.96) may result in a reduction of the total number of SREs (per 1000 participants 75 fewer (131 fewer to 14 fewer) and 131 fewer (215 fewer to 19 fewer); both low‐certainty evidence; 12 trials, 5240 participants). Treatment with zoledronic acid and denosumab likely neither reduces nor increases mortality when compared to no treatment/placebo (zoledronic acid RR 0.90, 95% CI 0.80 to 1.01; per 1000 participants 48 fewer (97 fewer to 5 more); denosumab RR 0.93, 95% CI 0.77 to 1.11; per 1000 participants 34 fewer (111 fewer to 54 more); both moderate‐certainty evidence; 13 trials, 5494 participants). Due to insufficient reporting, no network meta‐analysis was possible for the outcome quality of life. One study with 1904 participants comparing zoledronic acid and denosumab showed that more zoledronic acid‐treated participants than denosumab‐treated participants experienced a greater than or equal to five‐point decrease in Functional Assessment of Cancer Therapy‐General total scores over a range of 18 months (average relative difference = 6.8%, range −9.4% to 14.6%) or worsening of cancer‐related quality of life. Authors' conclusions When considering bone‐modifying agents as supportive treatment, one has to balance between efficacy and acceptability. Results suggest that Zoledronic acid likely increases both the proportion of participants with pain response, and the proportion of participants experiencing adverse events However, more trials with head‐to‐head comparisons including all potential agents are needed to draw the whole picture and proof the results of this analysis. Plain language summary Bone‐modifying agents for men with prostate cancer and bone metastases Review question In this systematic review we aimed to compare different agents to prevent skeletal complications in men with prostate cancer and bone metastases and to provide a ranking of these treatment options. We looked at different outcomes like reduction in pain, prevention of different skeletal‐related events, occurrence of adverse events, and quality of life. We wanted to find out which bone‐modifying agent is most effective while causing the fewest adverse events when given as supportive treatment to men with prostate cancer and bone metastases. Background The prostate is a gland in the male reproductive system. Prostate cancer can spread to other parts of the body (called metastases) including the bones. Bone metastases in men with prostate cancer may lead to skeletal complications like fractures or pain. Different bone‐modifying agents are used as supportive treatment to prevent skeletal complications through formation of new bone mass. Until now no clear recommendations could be given about which agents are the most effective while also causing the fewest adverse events. We used statistical methods to compare all agents with each other based on the available information. Study characteristics We conducted thorough searches in various databases until 23 March 2020. We included 25 studies comparing different bone‐modifying agents with each other or against no further treatment or placebo treatment (dummy treatment) in men with prostate cancer and bone metastases. Key results Twenty‐one of the 25 included studies reported data for our predefined patient‐relevant outcomes. A total of seven different agents were included, six bisphosphonates (zoledronic acid, risedronate, pamidronate, alendronate, etidronate, and clodronate) and one other agent, denosumab. Analysis was only possible for each outcome of interest separately. Considering skeletal‐related events, zoledronic acid and denosumab appeared to be the most effective, but also seemed to cause the most and worst adverse events (like renal impairment for treatment with zoledronic acid and osteonecrosis of the jaw for denosumab). Most of the included studies did not report data on quality of life or reported it very poorly, so that we could not analyse this outcome combining the information from different studies. The results were therefore described with words. Certainty of the evidence We rated the certainty of the evidence as high to low for the different agents and outcomes. A limitation of this review is that an overall ranking considering all outcomes at the same time is not possible. In order to make an informed decision about which treatment option should be used, one therefore must look at all the outcomes of interest and balance the pros and cons of each option.

UR - http://dx.doi.org/10.1002/14651858.CD013020.pub2

ER -

Record #14 of 32

Provider: John Wiley & Sons, Ltd

Content: text/plain; charset="UTF-8"

TY - JOUR

AN - CD008558

AU - Madsen, KS

AU - Chi, Y

AU - Metzendorf, MI

AU - Richter, B

AU - Hemmingsen, B

TI - Metformin for prevention or delay of type 2 diabetes mellitus and its associated complications in persons at increased risk for the development of type 2 diabetes mellitus

JF - Cochrane Database of Systematic Reviews

PY - 2019

IS - 12

PB - John Wiley & Sons, Ltd

SN - 1465-1858

KW - Diabetes Mellitus, Type 2 [*prevention & control]

KW - Glucose Intolerance

KW - Glycated Hemoglobin A

KW - Humans

KW - Hypoglycemic Agents [*therapeutic use]

KW - Metformin [*therapeutic use]

KW - Prediabetic State

KW - Quality of Life

KW - Randomized Controlled Trials as Topic

N1 - [Metabolic and Endocrine Disorders]

DO - 10.1002/14651858.CD008558.pub2

AB - Abstract - Background The projected rise in the incidence of type 2 diabetes mellitus (T2DM) could develop into a substantial health problem worldwide. Whether metformin can prevent or delay T2DM and its complications in people with increased risk of developing T2DM is unknown. Objectives To assess the effects of metformin for the prevention or delay of T2DM and its associated complications in persons at increased risk for the T2DM. Search methods We searched the Cochrane Central Register of Controlled Trials, MEDLINE, Scopus, ClinicalTrials.gov, the World Health Organization (WHO) International Clinical Trials Registry Platform and the reference lists of systematic reviews, articles and health technology assessment reports. We asked investigators of the included trials for information about additional trials. The date of the last search of all databases was March 2019. Selection criteria We included randomised controlled trials (RCTs) with a duration of one year or more comparing metformin with any pharmacological glucose‐lowering intervention, behaviour‐changing intervention, placebo or standard care in people with impaired glucose tolerance, impaired fasting glucose, moderately elevated glycosylated haemoglobin A1c (HbA1c) or combinations of these. Data collection and analysis Two review authors read all abstracts and full‐text articles and records, assessed risk of bias and extracted outcome data independently. We used a random‐effects model to perform meta‐analysis and calculated risk ratios (RRs) for dichotomous outcomes and mean differences (MDs) for continuous outcomes, using 95% confidence intervals (CIs) for effect estimates. We assessed the certainty of the evidence using GRADE. Main results We included 20 RCTs randomising 6774 participants. One trial contributed 48% of all participants. The duration of intervention in the trials varied from one to five years. We judged none of the trials to be at low risk of bias in all 'Risk of bias' domains. Our main outcome measures were all‐cause mortality, incidence of T2DM, serious adverse events (SAEs), cardiovascular mortality, non‐fatal myocardial infarction or stroke, health‐related quality of life and socioeconomic effects.The following comparisons mostly reported only a fraction of our main outcome set.  Fifteen RCTs compared metformin with diet and exercise with or without placebo: all‐cause mortality was 7/1353 versus 7/1480 (RR 1.11, 95% CI 0.41 to 3.01; P = 0.83; 2833 participants, 5 trials; very low‐quality evidence); incidence of T2DM was 324/1751 versus 529/1881 participants (RR 0.50, 95% CI 0.38 to 0.65; P < 0.001; 3632 participants, 12 trials; moderate‐quality evidence); the reporting of SAEs was insufficient and diverse and meta‐analysis could not be performed (reported numbers were 4/118 versus 2/191; 309 participants; 4 trials; very low‐quality evidence); cardiovascular mortality was 1/1073 versus 4/1082 (2416 participants; 2 trials; very low‐quality evidence). One trial reported no clear difference in health‐related quality of life after 3.2 years of follow‐up (very low‐quality evidence). Two trials estimated the direct medical costs (DMC) per participant for metformin varying from $220 to $1177 versus $61 to $184 in the comparator group (2416 participants; 2 trials; low‐quality evidence). Eight RCTs compared metformin with intensive diet and exercise: all‐cause mortality was 7/1278 versus 4/1272 (RR 1.61, 95% CI 0.50 to 5.23; P = 0.43; 2550 participants, 4 trials; very low‐quality evidence); incidence of T2DM was 304/1455 versus 251/1505 (RR 0.80, 95% CI 0.47 to 1.37; P = 0.42; 2960 participants, 7 trials; moderate‐quality evidence); the reporting of SAEs was sparse and meta‐analysis could not be performed (one trial reported 1/44 in the metformin group versus 0/36 in the intensive exercise and diet group with SAEs). One trial reported that 1/1073 participants in the metformin group compared with 2/1079 participants in the comparator group died from cardiovascular causes. One trial reported that no participant died due to cardiovascular causes (very low‐quality evidence). Two trials estimated the DMC per participant for metformin varying from $220 to $1177 versus $225 to $3628 in the comparator group (2400 participants; 2 trials; very low‐quality evidence). Three RCTs compared metformin with acarbose: all‐cause mortality was 1/44 versus 0/45 (89 participants; 1 trial; very low‐quality evidence); incidence of T2DM was 12/147 versus 7/148 (RR 1.72, 95% CI 0.72 to 4.14; P = 0.22; 295 participants; 3 trials; low‐quality evidence); SAEs were 1/51 versus 2/50 (101 participants; 1 trial; very low‐quality evidence). Three RCTs compared metformin with thiazolidinediones: incidence of T2DM was 9/161 versus 9/159 (RR 0.99, 95% CI 0.41 to 2.40; P = 0.98; 320 participants; 3 trials; low‐quality evidence). SAEs were 3/45 versus 0/41 (86 participants; 1 trial; very low‐quality evidence). Three RCTs compared metformin plus intensive diet and exercise with identical intensive diet and exercise: all‐cause mortality was 1/121 versus 1/120 participants (450 participants; 2 trials; very low‐quality evidence); incidence of T2DM was 48/166 versus 53/166 (RR 0.55, 95% CI 0.10 to 2.92; P = 0.49; 332 participants; 2 trials; very low‐quality evidence). One trial estimated the DMC of metformin plus intensive diet and exercise to be $270 per participant compared with $225 in the comparator group (94 participants; 1 trial; very‐low quality evidence). One trial in 45 participants compared metformin with a sulphonylurea. The trial reported no patient‐important outcomes. For all comparisons there were no data on non‐fatal myocardial infarction, non‐fatal stroke or microvascular complications. We identified 11 ongoing trials which potentially could provide data of interest for this review. These trials will add a total of 17,853 participants in future updates of this review. Authors' conclusions Metformin compared with placebo or diet and exercise reduced or delayed the risk of T2DM in people at increased risk for the development of T2DM (moderate‐quality evidence). However, metformin compared to intensive diet and exercise did not reduce or delay the risk of T2DM (moderate‐quality evidence). Likewise, the combination of metformin and intensive diet and exercise compared to intensive diet and exercise only neither showed an advantage or disadvantage regarding the development of T2DM (very low‐quality evidence). Data on patient‐important outcomes such as mortality, macrovascular and microvascular diabetic complications and health‐related quality of life were sparse or missing. Plain language summary Metformin for prevention/delay of type 2 diabetes mellitus (T2DM) and associated complications in persons at increased risk for development of T2DM Review question Is the antidiabetic drug metformin able to prevent or delay the development of type 2 diabetes and its associated complications in people with moderately elevated blood sugar levels? Background People with moderately elevated blood sugar levels (often referred to as 'prediabetes') are said to have an increased risk for developing diabetes. Metformin is a blood sugar‐lowering medicine which has been used for a long time to treat people with type 2 diabetes. Type 2 diabetes, also known as adult‐onset diabetes, is the most common type of diabetes and prevents the body from using insulin properly (insulin resistance). Type 2 diabetes can have bad effects on health in the long term (diabetic complications), such as severe eye or kidney disease or 'diabetic feet', eventually resulting in foot ulcers. We investigated whether metformin can also be used to prevent or delay type 2 diabetes in people at increased risk. We examined the effects of metformin on patient‐important outcomes, such as complications of diabetes, death from any cause, health‐related quality of life and side effects of the drug. Study characteristics To be included, people had to have blood sugar levels higher than normal, but below the levels that are used to diagnose diabetes. We found 20 randomised controlled trials (clinical studies where people are randomly put into one of two or more treatment groups) with a total of 6774 participants. The comparator group consisted of diet and exercise, intensive diet and exercise or another blood sugar‐lowering drug. One study dominated the evidence (48% of the total number of all participants). Twelve studies were performed in China. We only included studies with a treatment duration of one year or more. The treatment duration in the included studies varied from one to five years. This evidence is up to date as of March 2019. Key results Fifteen studies compared metformin against diet and exercise. Eight studies compared metformin against intensive diet and exercise and three studies compared metformin plus intensive diet and exercise against intensive diet and exercise only. When compared to standard diet and exercise metformin slightly reduces or delays development of diabetes. However, when compared to intensive diet and exercise, metformin does not provide an additional benefit in reducing or delaying development of diabetes. Seven studies compared metformin with another glucose‐lowering drug: three studies compared metformin with acarbose. Three studies compared metformin with a thiazolidinedione (such as pioglitazone). There was neither an advantage or disadvantage when comparing metformin with these drugs with respect to the development of diabetes. One study compared metformin with a sulphonylurea (glimepiride). The trial did not report patient‐important outcomes. In general, the reporting of serious side effects was sparse. Few participants died and we did not detect a clear difference between the intervention and comparator groups. We also did not detect an advantage or disadvantage of metformin in relation to health‐related quality of life. Our included studies did not report on non‐fatal heart attacks, strokes or complications of diabetes such as kidney or eye disease. Few studies estimated the direct medical costs. When compared to diet and exercise, metformin was more expensive. When compared to intensive diet and exercise, metformin was less expensive. We identified 11 ongoing studies which potentially could provide data for this review. These studies will add a total of 17,853 participants in future updates of our review. Future studies should investigate more patient‐important outcomes such as complications of diabetes and especially the side effects of the drugs. We do not know whether 'prediabetes' is just a condition defined by laboratory measurements, or whether it is in fact a real risk factor for diabetes. It is also unknown whether treatment of this condition translates into better patient‐important outcomes. Certainty of the evidence All included studies had problems in the way they were conduced or reported.

UR - http://dx.doi.org/10.1002/14651858.CD008558.pub2

ER -

Record #15 of 32

Provider: John Wiley & Sons, Ltd

Content: text/plain; charset="UTF-8"

TY - JOUR

AN - CD007613

AU - Ngo, K

AU - Kotecha, D

AU - Walters, JAE

AU - Manzano, L

AU - Palazzuoli, A

AU - van Veldhuisen, DJ

AU - Flather, M

TI - Erythropoiesis‐stimulating agents for anaemia in chronic heart failure patients

JF - Cochrane Database of Systematic Reviews

PY - 2010

IS - 1

PB - John Wiley & Sons, Ltd

SN - 1465-1858

KW - Anemia [*drug therapy, etiology]

KW - Heart Failure [*complications]

KW - Hematinics [*therapeutic use]

KW - Humans

KW - Randomized Controlled Trials as Topic

N1 - [Heart]

DO - 10.1002/14651858.CD007613.pub2

AB - Abstract - Background Chronic heart failure (CHF) is a leading cause of morbidity and mortality worldwide. Anaemia is a common (12‐55%) co‐morbid condition and is associated with worsening symptoms and increased mortality. Anaemia is treatable and can be targeted in the treatment of patients with CHF. Erythropoiesis‐stimulating agents (ESA), supplemented by iron therapy, are used to treat anaemia in chronic kidney disease and cancer, however safety concerns have been raised in these patients. The clinical benefit and safety of these agents in CHF remains unclear. Objectives To assess the benefits and risks of ESA for CHF patients with anaemia. Search methods We searched the Cochrane Central Register of Controlled Trials ( The Cochrane Library 2008, Issue 3), MEDLINE (1950 to October 2008), EMBASE (1980 to October 2008) and reference lists of articles. No language restrictions were applied. Selection criteria Randomised controlled trials of any ESA, with or without iron therapy, in CHF patients were eligible for inclusion. Data collection and analysis Three reviewers independently assessed study quality and extracted data. Original authors were contacted for additional information. The outcomes of interest were: exercise tolerance, haemoglobin level, New York Heart Association (NYHA) functional class, quality of life, left‐ventricular ejection fraction, B‐type natriuretic peptide, CHF‐related hospitalisations, all‐cause mortality and adverse effects. Risk ratios (RR) were calculated for dichotomous data and weighted mean difference (WMD) for continuous data. Main results Eleven studies (794 participants) were included. Overall quality of studies was moderate with nine studies being placebo‐controlled but only five double‐blinded. Compared to control, ESA treatment significantly improved exercise duration by 96.8 seconds (95% CI 5.2 to 188.4, p=0.04) and 6‐minute walk distance by 69.3 metres (95% CI 17.0 to 121.7, p=0.009). Benefit was also noted in terms of peak VO2 (+2.29 mL/kg/min, p=0.007), NYHA class (‐0.73, p<0.001), ejection fraction (+5.8%, p<0.001), B‐type natriuretic peptide (‐226.99 pg/mL, p<0.001) and quality‐of‐life indicators, with a mean increase in haemoglobin of 1.98 g/dL (p<0.0001). There was also a significantly lower rate of heart failure related hospitalisations (RR 0.62, 95% CI 0.44 to 0.87) and lower all‐cause mortality (RR 0.61, 95% CI 0.37 to 0.99). No increase in adverse events with ESA therapy was observed, however studies were of small sample sizes and limited duration. Authors' conclusions Meta‐analysis of small RCTs suggests that ESA treatment in patients with symptomatic CHF and mild anaemia (haemoglobin more than 10g/dL) can improve anaemia and exercise tolerance, reduce symptoms and have benefits on clinical outcomes. Confirmation requires well‐designed studies with careful attention to dose, haemoglobin treatment target and associated iron therapy. Plain language summary Erythropoiesis‐stimulating agents for people with chronic heart failure and anaemia Chronic heart failure is a disorder in which the heart is unable to pump blood and deliver oxygen adequately throughout the body. Patients with heart failure may also suffer from anaemia, a condition of reduced red blood cells and diminished ability of the blood to carry oxygen. These patients appear to have worse symptoms and poorer survival and may benefit from additional therapy for their anaemia. Erythropoiesis‐stimulating agents (ESAs) with iron supplements have been used since the 1980s to treat anaemia in chronic kidney disease and cancer patients. ESAs have the same action as erythropoietin, a hormone that is naturally produced by the kidneys to increase red blood cell production. This review shows that ESAs improves anaemia, exercise tolerance, quality of life and reduces symptoms in heart failure patients with a mild anaemia. ESAs may also reduce hospital admission and improve survival. There was no increase in major side effects in those receiving ESA therapy compared to control over the 2‐12 month study period (maximum 12 months) although the effects of treatment over a longer period are not known. More research is needed to clarify the full effects and safety of ESAs as a treatment for anaemia in these patients.

UR - http://dx.doi.org/10.1002/14651858.CD007613.pub2

ER -

Record #16 of 32

Provider: John Wiley & Sons, Ltd

Content: text/plain; charset="UTF-8"

TY - JOUR

AN - CD015045

AU - Mikolajewska, A

AU - Fischer, A-L

AU - Piechotta, V

AU - Mueller, A

AU - Metzendorf, M-I

AU - Becker, M

AU - Dorando, E

AU - Pacheco, RL

AU - Martimbianco, ALC

AU - Riera, R

AU - et al.

TI - Colchicine for the treatment of COVID‐19

JF - Cochrane Database of Systematic Reviews

PY - 2021

IS - 10

PB - John Wiley & Sons, Ltd

SN - 1465-1858

KW - *COVID-19

KW - *Colchicine [adverse effects]

KW - Cause of Death

KW - Humans

KW - Male

KW - Middle Aged

KW - Quality of Life

KW - SARS-CoV-2

N1 - [Haematology]

DO - 10.1002/14651858.CD015045

AB - Abstract - Background The development of severe coronavirus disease 2019 (COVID‐19) and poor clinical outcomes are associated with hyperinflammation and a complex dysregulation of the immune response. Colchicine is an anti‐inflammatory medicine and is thought to improve disease outcomes in COVID‐19 through a wide range of anti‐inflammatory mechanisms. Patients and healthcare systems need more and better treatment options for COVID‐19 and a thorough understanding of the current body of evidence. Objectives To assess the effectiveness and safety of Colchicine as a treatment option for COVID‐19 in comparison to an active comparator, placebo, or standard care alone in any setting, and to maintain the currency of the evidence, using a living systematic review approach. Search methods We searched the Cochrane COVID‐19 Study Register (comprising CENTRAL, MEDLINE (PubMed), Embase, ClinicalTrials.gov, WHO International Clinical Trials Registry Platform, and medRxiv), Web of Science (Science Citation Index Expanded and Emerging Sources Citation Index), and WHO COVID‐19 Global literature on coronavirus disease to identify completed and ongoing studies without language restrictions to 21 May 2021. Selection criteria We included randomised controlled trials evaluating colchicine for the treatment of people with COVID‐19, irrespective of disease severity, age, sex, or ethnicity. We excluded studies investigating the prophylactic effects of colchicine for people without severe acute respiratory syndrome coronavirus 2 (SARS‐CoV‐2) infection but at high risk of SARS‐CoV‐2 exposure. Data collection and analysis We followed standard Cochrane methodology. We used the Cochrane risk of bias tool (ROB 2) to assess bias in included studies and GRADE to rate the certainty of evidence for the following prioritised outcome categories considering people with moderate or severe COVID‐19: all‐cause mortality, worsening and improvement of clinical status, quality of life, adverse events, and serious adverse events and for people with asymptomatic infection or mild disease: all‐cause mortality, admission to hospital or death, symptom resolution, duration to symptom resolution, quality of life, adverse events, serious adverse events. Main results We included three RCTs with 11,525 hospitalised participants (8002 male) and one RCT with 4488 (2067 male) non‐hospitalised participants. Mean age of people treated in hospital was about 64 years, and was 55 years in the study with non‐hospitalised participants. Further, we identified 17 ongoing studies and 11 studies completed or terminated, but without published results. Colchicine plus standard care versus standard care (plus/minus placebo) Treatment of hospitalised people with moderate to severe COVID‐19 All‐cause mortality : colchicine plus standard care probably results in little to no difference in all‐cause mortality up to 28 days compared to standard care alone (risk ratio (RR) 1.00, 95% confidence interval (CI) 0.93 to 1.08; 2 RCTs, 11,445 participants; moderate‐certainty evidence). Worsening of clinical status : colchicine plus standard care probably results in little to no difference in worsening of clinical status assessed as new need for invasive mechanical ventilation or death compared to standard care alone (RR 1.02, 95% CI 0.96 to 1.09; 2 RCTs, 10,916 participants; moderate‐certainty evidence). Improvement of clinical status : colchicine plus standard care probably results in little to no difference in improvement of clinical status, assessed as number of participants discharged alive up to day 28 without clinical deterioration or death compared to standard care alone (RR 0.99, 95% CI 0.96 to 1.01; 1 RCT, 11,340 participants; moderate‐certainty evidence). Quality of lif e , including fatigue and neurological status : we identified no studies reporting this outcome. Adverse events : the evidence is very uncertain about the effect of colchicine on adverse events compared to placebo (RR 1.00, 95% CI 0.56 to 1.78; 1 RCT, 72 participants; very low‐certainty evidence). Serious adverse events : the evidence is very uncertain about the effect of colchicine plus standard care on serious adverse events compared to standard care alone (0 events observed in 1 RCT of 105 participants; very low‐certainty evidence). Treatment of non‐hospitalised people with asymptomatic SARS‐CoV‐2 infection or mild COVID‐19 All‐cause mortality : the evidence is uncertain about the effect of colchicine on all‐cause mortality at 28 days (Peto odds ratio (OR) 0.57, 95% CI 0.20 to 1.62; 1 RCT, 4488 participants; low‐certainty evidence). Admission to hospital or death within 28 days : colchicine probably slightly reduces the need for hospitalisation or death within 28 days compared to placebo (RR 0.80, 95% CI 0.62 to 1.03; 1 RCT, 4488 participants; moderate‐certainty evidence). Symptom resolution : we identified no studies reporting this outcome. Quality of lif e , including fatigue and neurological status : we identified no studies reporting this outcome. Adverse events : the evidence is uncertain about the effect of colchicine on adverse events compared to placebo . Results are from one RCT reporting treatment‐related events only in 4412 participants (low‐certainty evidence). Serious adverse events : colchicine probably slightly reduces serious adverse events (RR 0.78, 95% CI 0.61 to 1.00; 1 RCT, 4412 participants; moderate‐certainty evidence). Colchicine versus another active treatment (e.g. corticosteroids, anti‐viral drugs, monoclonal antibodies) No studies evaluated this comparison. Different formulations, doses, or schedules of colchicine No studies assessed this. Authors' conclusions Based on the current evidence, in people hospitalised with moderate to severe COVID‐19 the use of colchicine probably has little to no influence on mortality or clinical progression in comparison to placebo or standard care alone. We do not know whether colchicine increases the risk of (serious) adverse events. We are uncertain about the evidence of the effect of colchicine on all‐cause mortality for people with asymptomatic infection or mild disease. However, colchicine probably results in a slight reduction of hospital admissions or deaths within 28 days, and the rate of serious adverse events compared with placebo. None of the studies reported data on quality of life or compared the benefits and harms of colchicine versus other drugs, or different dosages of colchicine. We identified 17 ongoing and 11 completed but not published RCTs, which we expect to incorporate in future versions of this review as their results become available. Editorial note: due to the living approach of this work, we monitor newly published results of RCTs on colchicine on a weekly basis and will update the review when the evidence or our certainty in the evidence changes. Plain language summary Is colchicine an effective treatment for people with COVID‐19? Key messages ○ In hospitalised people with moderate to severe COVID‐19, colchicine probably has little to no benefit; we are uncertain about its side effects. ○In non‐hospitalised people with no symptoms or mild COVID‐19, we are uncertain whether colchicine prevents deaths or side effects, however it probably reduces the need for hospitalisation or death and serious side effects slightly. ○ Future studies should assess quality of life in people with no symptoms or mild COVID‐19 and non‐serious side effects and compare colchicine to other medicines for COVID‐19, such as corticosteroids. What is colchicine? Colchicine is a medicine used to reduce swelling and inflammation and may consequently relieve pain. It is often used to treat gout, a condition where people’s joints become swollen and painful. On the other hand, colchicine can be harmful to people with some health conditions, such as kidney or liver problems, or if you take too much of it. How might colchicine treat COVID‐19? Since colchicine is an anti‐inflammatory drug; researchers are interested in whether it might help with reducing inflammation caused by COVID‐19. What did we want to find out? We wanted to know whether colchicine is an effective treatment for people with COVID‐19 compared to placebo (a treatment that looks and tastes the same as colchicine but with no active ingredient) or usual care alone. We looked at people with moderate or severe disease being treated in hospital or with mild disease being treated in the community. We were particularly interested in the effects of colchicine on: ○ number of deaths; ○ whether people’s condition worsened or improved; ○ quality of life; ○ serious and non‐serious side effects What did we do? We searched for studies that compared colchicine together with usual care to usual care (plus/minus placebo). Studies could take place anywhere in the world and include people with mild or no symptoms, moderate or severe COVID‐19, of any age, sex, or ethnicity. We compared and summarised the results of the studies and rated our certainty in the evidence, based on factors such as study methods and sizes. What did we find? We identified four eligible randomised trials. Three included 11,525 hospitalised people and one included 4488 non‐hospitalised people. For hospitalised people, the average age was 64 years, and for non‐hospitalised people, the average age was 55 years. Two studies compared colchicine and usual care with usual care alone and 2 studies compared colchicine with usual care and placebo. None of the studies reported quality of life. We also found 17 ongoing studies and 11 completed but unpublished studies. Main results Hospitalised people with moderate to severe COVID‐19 (3 studies, 11,525 people) ○Colchicine probably does not reduce deaths in the 28 days after treatment (2 studies, 11,445 people). ○Colchicine probably does not prevent the worsening of patients’ condition (2 studies, 10,916 people) and probably does not improve it (1 study, 11,340 people). ○We are very uncertain about the effect of colchicine on side effects and serious side effects (2 studies, 177 people). Non‐hospitalised people with no symptoms or mild COVID‐19 (1 study, 4488 people) ○We are uncertain whether colchicine prevents deaths up to 28 days after treatment. ○Colchicine probably slightly reduces the risk of hospitalisation or death. ○We are uncertain about the effect of colchicine on side effects, but it probably slightly reduces serious side effects. What are the limitations of the evidence? Our certainty in the evidence is limited. Two studies did not use a placebo, so everybody knew who was treated with colchicine, which could influence the results. There were too few events for non‐hospitalised people, such as admissions to hospital and deaths, to be certain about the evidence. Studies used different ways to assess and report unwanted effects, so we could not combine studies into a single result to make a judgement. How up to date is this evidence? The evidence is up to date to 21 May 2021. Editorial note: this is a living systematic review. We search for new evidence every week and update the review when we identify relevant new evidence. Refer to the Cochrane Database of Systematic Reviews for the current status of this review.

UR - http://dx.doi.org/10.1002/14651858.CD015045

ER -

Record #17 of 32

Provider: John Wiley & Sons, Ltd

Content: text/plain; charset="UTF-8"

TY - JOUR

AN - CD012069

AU - Storebø, OJ

AU - Pedersen, N

AU - Ramstad, E

AU - Kielsholm, ML

AU - Nielsen, SS

AU - Krogh, HB

AU - Moreira‐Maia, CR

AU - Magnusson, FL

AU - Holmskov, M

AU - Gerner, T

AU - et al.

TI - Methylphenidate for attention deficit hyperactivity disorder (ADHD) in children and adolescents – assessment of adverse events in non‐randomised studies

JF - Cochrane Database of Systematic Reviews

PY - 2018

IS - 5

PB - John Wiley & Sons, Ltd

SN - 1465-1858

KW - Adolescent

KW - Attention Deficit Disorder with Hyperactivity [*drug therapy]

KW - Central Nervous System Stimulants [*adverse effects, therapeutic use]

KW - Child

KW - Child, Preschool

KW - Humans

KW - Methylphenidate [*adverse effects, therapeutic use]

KW - Non‐Randomized Controlled Trials as Topic

KW - Patient Dropouts [statistics & numerical data]

KW - Young Adult

N1 - [Developmental, Psychosocial and Learning Problems]

DO - 10.1002/14651858.CD012069.pub2

AB - Abstract - Background Attention deficit hyperactivity disorder (ADHD) is a common neurodevelopmental disorder in childhood. The psychostimulant methylphenidate is the most frequently used medication to treat it. Several studies have investigated the benefits of methylphenidate, showing possible favourable effects on ADHD symptoms, but the true magnitude of the effect is unknown. Concerning adverse events associated with the treatment, our systematic review of randomised clinical trials (RCTs) demonstrated no increase in serious adverse events, but a high proportion of participants suffered a range of non‐serious adverse events. Objectives To assess the adverse events associated with methylphenidate treatment for children and adolescents with ADHD in non‐randomised studies. Search methods In January 2016, we searched CENTRAL, MEDLINE, Embase, PsycINFO, CINAHL, 12 other databases and two trials registers. We also checked reference lists and contacted authors and pharmaceutical companies to identify additional studies. Selection criteria We included non‐randomised study designs. These comprised comparative and non‐comparative cohort studies, patient‐control studies, patient reports/series and cross‐sectional studies of methylphenidate administered at any dosage or formulation. We also included methylphenidate groups from RCTs assessing methylphenidate versus other interventions for ADHD as well as data from follow‐up periods in RCTs. Participants had to have an ADHD diagnosis (from the 3rd to the 5th edition of the Diagnostic and Statistical Manual of Mental Disorders or the 9th or 10th edition of the International Classification of Diseases , with or without comorbid diagnoses. We required that at least 75% of participants had a normal intellectual capacity (intelligence quotient of more than 70 points) and were aged below 20 years. We excluded studies that used another ADHD drug as a co‐intervention. Data collection and analysis Fourteen review authors selected studies independently. Two review authors assessed risk of bias independently using the ROBINS‐I tool for assessing r isk o f b ias i n n on‐randomised s tudies of i nterventions. All review authors extracted data. We defined serious adverse events according to the International Committee of Harmonization as any lethal, life‐threatening or life‐changing event. We considered all other adverse events to be non‐serious adverse events and conducted meta‐analyses of data from comparative studies. We calculated meta‐analytic estimates of prevalence from non‐comparative cohorts studies and synthesised data from patient reports/series qualitatively. We investigated heterogeneity by conducting subgroup analyses, and we also conducted sensitivity analyses. Main results We included a total of 260 studies: 7 comparative cohort studies, 6 of which compared 968 patients who were exposed to methylphenidate to 166 controls, and 1 which assessed 1224 patients that were exposed or not exposed to methylphenidate during different time periods; 4 patient‐control studies (53,192 exposed to methylphenidate and 19,906 controls); 177 non‐comparative cohort studies (2,207,751 participants); 2 cross‐sectional studies (96 participants) and 70 patient reports/series (206 participants). Participants' ages ranged from 3 years to 20 years. Risk of bias in the included comparative studies ranged from moderate to critical, with most studies showing critical risk of bias. We evaluated all non‐comparative studies at critical risk of bias. The GRADE quality rating of the evidence was very low. Primary outcomes In the comparative studies, methylphenidate increased the risk ratio (RR) of serious adverse events (RR 1.36, 95% confidence interval (CI) 1.17 to 1.57; 2 studies, 72,005 participants); any psychotic disorder (RR 1.36, 95% CI 1.17 to 1.57; 1 study, 71,771 participants); and arrhythmia (RR 1.61, 95% CI 1.48 to 1.74; 1 study, 1224 participants) compared to no intervention. In the non‐comparative cohort studies, the proportion of participants on methylphenidate experiencing any serious adverse event was 1.20% (95% CI 0.70% to 2.00%; 50 studies, 162,422 participants). Withdrawal from methylphenidate due to any serious adverse events occurred in 1.20% (95% CI 0.60% to 2.30%; 7 studies, 1173 participants) and adverse events of unknown severity led to withdrawal in 7.30% of participants (95% CI 5.30% to 10.0%; 22 studies, 3708 participants). Secondary outcomes In the comparative studies, methylphenidate, compared to no intervention, increased the RR of insomnia and sleep problems (RR 2.58, 95% CI 1.24 to 5.34; 3 studies, 425 participants) and decreased appetite (RR 15.06, 95% CI 2.12 to 106.83; 1 study, 335 participants). With non‐comparative cohort studies, the proportion of participants on methylphenidate with any non‐serious adverse events was 51.2% (95% CI 41.2% to 61.1%; 49 studies, 13,978 participants). These included difficulty falling asleep, 17.9% (95% CI 14.7% to 21.6%; 82 studies, 11,507 participants); headache, 14.4% (95% CI 11.3% to 18.3%; 90 studies, 13,469 participants); abdominal pain, 10.7% (95% CI 8.60% to 13.3%; 79 studies, 11,750 participants); and decreased appetite, 31.1% (95% CI 26.5% to 36.2%; 84 studies, 11,594 participants). Withdrawal of methylphenidate due to non‐serious adverse events occurred in 6.20% (95% CI 4.80% to 7.90%; 37 studies, 7142 participants), and 16.2% were withdrawn for unknown reasons (95% CI 13.0% to 19.9%; 57 studies, 8340 participants). Authors' conclusions Our findings suggest that methylphenidate may be associated with a number of serious adverse events as well as a large number of non‐serious adverse events in children and adolescents, which often lead to withdrawal of methylphenidate. Our certainty in the evidence is very low, and accordingly, it is not possible to accurately estimate the actual risk of adverse events. It might be higher than reported here. Given the possible association between methylphenidate and the adverse events identified, it may be important to identify people who are most susceptible to adverse events. To do this we must undertake large‐scale, high‐quality RCTs, along with studies aimed at identifying responders and non‐responders. Plain language summary Methylphenidate for attention deficit hyperactivity disorder (ADHD) in children and adolescents ‐ assessment of harmful effects Review question Is methylphenidate administration associated with harmful effects in children and adolescents with attention deficit hyperactivity disorder (ADHD)? Background ADHD is one of the most common neurodevelopmental disorders in childhood and is associated with impaired functioning and negative outcomes for development. Individuals diagnosed with ADHD are often hyperactive and impulsive. Methylphenidate, a psychostimulant, is the drug most often prescribed for children and adolescents with ADHD. Study characteristics We searched for available research up to January 2016 and found 260 studies with different designs. We included a number of non‐randomised designs (where investigators did not assign participants to a certain treatment): – 7 comparative cohort studies (a group of people followed over time; six studies compared 968 patients who were taking methylphenidate to 166 controls who were not taking methylphenidate; and 1 study included 1224 patients that were taking or not taking methylphenidate during different time periods);  – 4 patient‐control studies (comparing two groups of people: 53,192 were taking methylphenidate, and 19,906 were not);  – 177 non‐comparative cohort studies (2,207,751 participants) with no control group (i.e. who were not taking methylphenidate);  – 2 cross‐sectional studies (96 participants were taking methylphenidate at a single time point); and  – 70 patient reports/series (206 participants were taking methylphenidate). We also included methylphenidate groups from randomised clinical trials (RCTs; experiments in which participants are randomly put into independent groups that compare different treatments). All RCTs assessed methylphenidate versus other interventions for ADHD and follow‐up periods from RCTs. We only used the data from the intervention arm with methylphenidate. In all the included non‐comparative cohort studies, 2,207,751 participants were taking methylphenidate. Participants' ages ranged from 3 years to 20 years. Key results The findings suggest that methylphenidate administration might lead to serious adverse (harmful) events, including death, cardiac problems, and psychotic disorders. About 1 in 100 patients treated with methylphenidate seemed to suffer a serious adverse event. Withdrawal from methylphenidate due to serious adverse events occurred in about 1.2 out of 100 patients treated with methylphenidate. Withdrawal from methylphenidate due to any adverse events occurred in about 7.3 out of 100 patients treated with methylphenidate. We also noted a large proportion of non‐serious adverse events. More than half the patients exposed to methylphenidate seemed to suffer one or more adverse events. Withdrawal from methylphenidate due to non‐serious adverse events occurred in about 6.2 out of 100 patients exposed to methylphenidate. Withdrawal of methylphenidate for unknown reasons was 16.2 out of 100 patients exposed to methylphenidate. Quality of the evidence The quality of the evidence and hence the certainty or reliability of the evidence for the comparative studies is very low. The reliability of the evidence for the non‐comparative studies is low due to weaknesses in study design. Accordingly, it is not possible to accurately estimate the risks of adverse events in children and adolescents prescribed methylphenidate. Conclusions Methyphenidiate might be associated with a number of serious adverse events. Methylphenidate produces a large number of other non‐serious harmful effects in children and adolescents with ADHD. We suggest that clinicians and parents are alert to the importance of monitoring adverse events in a systematic, meticulous manner. If methylphenidate is to continue to have a place in ADHD treatment in the future, we need to identify subgroups of patients in whom the benefits of methylphenidate outweigh the harms. Just as we need to be able to identify who is likely to benefit from treatment, we also need to be able to identify those who are most at risk of experiencing adverse events. In order to do this, we need to undertake large‐scale, high‐quality RCTs along with other studies aimed at identifying those who respond and those who do not respond to treatment.

UR - http://dx.doi.org/10.1002/14651858.CD012069.pub2

ER -

Record #18 of 32

Provider: John Wiley & Sons, Ltd

Content: text/plain; charset="UTF-8"

TY - JOUR

AN - CD013516

AU - Ipsen, EØ

AU - Madsen, KS

AU - Chi, Y

AU - Pedersen-Bjergaard, U

AU - Richter, B

AU - Metzendorf, M-I

AU - Hemmingsen, B

TI - Pioglitazone for prevention or delay of type 2 diabetes mellitus and its associated complications in people at risk for the development of type 2 diabetes mellitus

JF - Cochrane Database of Systematic Reviews

PY - 2020

IS - 11

PB - John Wiley & Sons, Ltd

SN - 1465-1858

KW - Acarbose [therapeutic use]

KW - Bias

KW - Carbamates [therapeutic use]

KW - Cardiovascular Diseases [mortality]

KW - Confidence Intervals

KW - Diabetes Mellitus, Type 2 [complications, *prevention & control]

KW - Humans

KW - Hypoglycemic Agents [*therapeutic use]

KW - Metformin [therapeutic use]

KW - Pioglitazone [*therapeutic use]

KW - Piperidines [therapeutic use]

KW - Placebos [therapeutic use]

KW - Randomized Controlled Trials as Topic

KW - Risk

N1 - [Metabolic and Endocrine Disorders]

DO - 10.1002/14651858.CD013516.pub2

AB - Abstract - Background The term prediabetes is used to describe a population with an elevated risk of developing type 2 diabetes mellitus (T2DM). With projections of an increase in the incidence of T2DM, prevention or delay of the disease and its complications is paramount. It is currently unknown whether pioglitazone is beneficial in the treatment of people with increased risk of developing T2DM. Objectives To assess the effects of pioglitazone for prevention or delay of T2DM and its associated complications in people at risk of developing T2DM. Search methods We searched CENTRAL, MEDLINE, Chinese databases, ICTRP Search Portal and ClinicalTrials.gov. We did not apply any language restrictions. Further, we investigated the reference lists of all included studies and reviews. We tried to contact all study authors. The date of the last search of databases was November 2019 (March 2020 for Chinese databases). Selection criteria We included randomised controlled trials (RCTs) with a minimum duration of 24 weeks, and participants diagnosed with intermediate hyperglycaemia with no concomitant diseases, comparing pioglitazone as monotherapy or part of dual therapy with other glucose‐lowering drugs, behaviour‐changing interventions, placebo or no intervention. Data collection and analysis Two review authors independently screened abstracts, read full‐text articles and records, assessed risk of bias and extracted data. We performed meta‐analyses with a random‐effects model and calculated risk ratios (RRs) for dichotomous outcomes and mean differences (MDs) for continuous outcomes, with 95% confidence intervals (CIs) for effect estimates. We evaluated the certainty of the evidence with the GRADE. Main results We included 27 studies with a total of 4186 randomised participants. The size of individual studies ranged between 43 and 605 participants and the duration varied between 6 and 36 months. We judged none of the included studies as having low risk of bias across all 'Risk of bias' domains. Most studies identified people at increased risk of T2DM by impaired fasting glucose or impaired glucose tolerance (IGT), or both. Our main outcome measures were all‐cause mortality, incidence of T2DM, serious adverse events (SAEs), cardiovascular mortality, nonfatal myocardial infarction or stroke (NMI/S), health‐related quality of life (QoL) and socioeconomic effects. The following comparisons mostly reported only a fraction of our main outcome set. Three studies compared pioglitazone with metformin. They did not report all‐cause and cardiovascular mortality, NMI/S, QoL or socioeconomic effects. Incidence of T2DM was 9/168 participants in the pioglitazone groups versus 9/163 participants in the metformin groups (RR 0.98, 95% CI 0.40 to 2.38; P = 0.96; 3 studies, 331 participants; low‐certainty evidence). No SAEs were reported in two studies (201 participants; low‐certainty evidence). One study compared pioglitazone with acarbose. Incidence of T2DM was 1/50 participants in the pioglitazone group versus 2/46 participants in the acarbose group (very low‐certainty evidence). No participant experienced a SAE (very low‐certainty evidence).One study compared pioglitazone with repaglinide. Incidence of T2DM was 2/48 participants in the pioglitazone group versus 1/48 participants in the repaglinide group (low‐certainty evidence). No participant experienced a SAE (low‐certainty evidence). One study compared pioglitazone with a personalised diet and exercise consultation. All‐cause and cardiovascular mortality, NMI/S, QoL or socioeconomic effects were not reported. Incidence of T2DM was 2/48 participants in the pioglitazone group versus 5/48 participants in the diet and exercise consultation group (low‐certainty evidence). No participant experienced a SAE (low‐certainty evidence). Six studies compared pioglitazone with placebo. No study reported on QoL or socioeconomic effects. All‐cause mortality was 5/577 participants the in the pioglitazone groups versus 2/579 participants in the placebo groups (Peto odds ratio 2.38, 95% CI 0.54 to 10.50; P = 0.25; 4 studies, 1156 participants; very low‐certainty evidence). Incidence of T2DM was 80/700 participants in the pioglitazone groups versus 131/695 participants in the placebo groups (RR 0.40, 95% CI 0.17 to 0.95; P = 0.04; 6 studies, 1395 participants; low‐certainty evidence). There were 3/93 participants with SAEs in the pioglitazone groups versus 1/94 participants in the placebo groups (RR 3.00, 95% CI 0.32 to 28.22; P = 0.34; 2 studies, 187 participants; very low‐certainty evidence). However, the largest study for this comparison did not distinguish between serious and non‐serious adverse events. This study reported that 121/303 (39.9%) participants in the pioglitazone group versus 151/299 (50.5%) participants in the placebo group experienced an adverse event (P = 0.03). One study observed cardiovascular mortality in 2/181 participants in the pioglitazone group versus 0/186 participants in the placebo group (RR 5.14, 95% CI 0.25 to 106.28; P = 0.29; very low‐certainty evidence). One study observed NMI in 2/303 participants in the pioglitazone group versus 1/299 participants in the placebo group (RR 1.97: 95% CI 0.18 to 21.65; P = 0.58; very low‐certainty evidence). Twenty‐one studies compared pioglitazone with no intervention. No study reported on cardiovascular mortality, NMI/S, QoL or socioeconomic effects. All‐cause mortality was 11/441 participants in the pioglitazone groups versus 12/425 participants in the no‐intervention groups (RR 0.85, 95% CI 0.38 to 1.91; P = 0.70; 3 studies, 866 participants; very low‐certainty evidence). Incidence of T2DM was 60/1034 participants in the pioglitazone groups versus 197/1019 participants in the no‐intervention groups (RR 0.31, 95% CI 0.23 to 0.40; P < 0.001; 16 studies, 2053 participants; moderate‐certainty evidence). Studies reported SAEs in 16/610 participants in the pioglitazone groups versus 21/601 participants in the no‐intervention groups (RR 0.71, 95% CI 0.38 to 1.32; P = 0.28; 7 studies, 1211 participants; low‐certainty evidence). We identified two ongoing studies, comparing pioglitazone with placebo and with other glucose‐lowering drugs. These studies, with 2694 participants. may contribute evidence to future updates of this review. Authors' conclusions Pioglitazone reduced or delayed the development of T2DM in people at increased risk of T2DM compared with placebo (low‐certainty evidence) and compared with no intervention (moderate‐certainty evidence). It is unclear whether the effect of pioglitazone is sustained once discontinued. Pioglitazone compared with metformin neither showed advantage nor disadvantage regarding the development of T2DM in people at increased risk (low‐certainty evidence). The data and reporting of all‐cause mortality, SAEs, micro‐ and macrovascular complications were generally sparse. None of the included studies reported on QoL or socioeconomic effects. Plain language summary Does pioglitazone prevent or delay type 2 diabetes and its complications in people at risk of developing type 2 diabetes mellitus? What is type 2 diabetes? Type 2 diabetes, also known as adult‐onset diabetes, is the most common type of diabetes. It prevents the body from using insulin properly ‐ insulin is a hormone that helps the body to regulate blood sugar levels. People with type 2 diabetes may suffer long‐term effects (diabetic complications), such as eye or kidney disease, or develop foot ulcers. People with moderately elevated blood sugar levels (often referred to as 'prediabetes') are said to have an increased risk of developing diabetes. Pioglitazone is a blood sugar‐lowering medicine, which is used to treat people with type 2 diabetes. What did we want to find out? We wanted to know whether pioglitazone can also be used to prevent or delay type 2 diabetes in people at increased risk of developing the condition. We examined the effects of pioglitazone on important outcomes for patients, such as complications of diabetes, death from any cause, health‐related quality of life and unwanted effects of the treatment. What did we do? We searched for studies that investigated pioglitazone used to prevent or delay the onset of type 2 diabetes. Participants had to have elevated blood sugar levels, but lower than diagnostic levels for diabetes, and they needed to be free from other diseases. Studies had to apply the intervention (pioglitazone) for at least 24 weeks. What we found We found 27 randomised controlled trials (clinical studies where people are randomly put into one of two or more treatment groups) with a total of 4186 participants. The studies compared pioglitazone with other antidiabetic drugs, diet and exercise, placebo (a 'sham' treatment), or no intervention. Twenty‐three out of 27 studies were conducted in China. The studies lasted between 24 weeks and three years. This evidence is up to date as of November 2019. Key results Five studies compared pioglitazone with other antidiabetic drugs (metformin, acarbose or repaglinide) and one study compared pioglitazone with diet and exercise. There were no clear beneficial or harmful effects on the risk of developing diabetes comparing the drugs. Six studies compared pioglitazone with placebo. There was a reduction or delay in the development of type 2 diabetes: 188 out of 1000 people treated with placebo developed type 2 diabetes compared with 75 per 1000 people treated with pioglitazone (possible spread: 32 per 1000 to 179 per 1000). Twenty‐three studies compared pioglitazone with no intervention. There was a reduction or delay in the development of type 2 diabetes: 193 out of 1000 people with no intervention developed type 2 diabetes compared with 60 per 1000 treated with pioglitazone (possible spread: 44 per 1000 to 77 per 1000). Only a few studies reported death from any cause, serious unwanted effects, non‐fatal heart‐attacks or strokes. We were not able to detect any clear benefits or harms of pioglitazone for these outcomes. None of the included studies reported health‐related quality of life or socioeconomic effects (such as costs of the intervention, absence from work, medication consumption). We found two ongoing studies that we could potentially include in this review. These studies may contribute data from around 2694 participants to future updates of our review. Future research should focus on whether the effect of pioglitazone is sustained after people stop taking it. Furthermore, research should focus on patient‐important outcomes such as unwanted effects and complications of diabetes. Quality of the evidence All studies had problems in their methods or the way they reported results. Moreover, many outcomes were reported by no or just a few studies. We are therefore uncertain whether pioglitazone prevents or delays type 2 diabetes in people at risk of developing the condition.

UR - http://dx.doi.org/10.1002/14651858.CD013516.pub2

ER -

Record #19 of 32

Provider: John Wiley & Sons, Ltd

Content: text/plain; charset="UTF-8"

TY - JOUR

AN - CD008294

AU - Robertson, NU

AU - Schoonees, A

AU - Brand, A

AU - Visser, J

TI - Pine bark (Pinus spp.) extract for treating chronic disorders

JF - Cochrane Database of Systematic Reviews

PY - 2020

IS - 9

PB - John Wiley & Sons, Ltd

SN - 1465-1858

KW - Adolescent

KW - Adult

KW - Antioxidants [*therapeutic use]

KW - Asthma [drug therapy]

KW - Attention Deficit Disorder with Hyperactivity [drug therapy]

KW - Bias

KW - Bone Diseases, Metabolic [drug therapy]

KW - Brain Injuries, Traumatic [drug therapy]

KW - Cardiovascular Diseases [drug therapy]

KW - Child

KW - Chronic Disease [*drug therapy]

KW - Diabetes Mellitus, Type 1 [drug therapy]

KW - Diabetes Mellitus, Type 2 [drug therapy]

KW - Erectile Dysfunction [drug therapy]

KW - Female

KW - Flavonoids [*therapeutic use]

KW - Humans

KW - Hypertension [drug therapy]

KW - Male

KW - Middle Aged

KW - Osteoarthritis [drug therapy]

KW - Pinus

KW - Plant Bark [*chemistry]

KW - Plant Extracts [*therapeutic use]

KW - Randomized Controlled Trials as Topic

KW - Sexual Dysfunctions, Psychological [drug therapy]

KW - Venous Insufficiency [drug therapy]

N1 - [Cystic Fibrosis and Genetic Disorders]

DO - 10.1002/14651858.CD008294.pub5

AB - Abstract - Background Pine bark ( Pinus spp.) extract is rich in bioflavonoids, predominantly proanthocyanidins, which are antioxidants. Commercially‐available extract supplements are marketed for preventing or treating various chronic conditions associated with oxidative stress. This is an update of a previously published review. Objectives To assess the efficacy and safety of pine bark extract supplements for treating chronic disorders. Search methods We searched three databases and three trial registries; latest search: 30 September 2019. We contacted the manufacturers of pine bark extracts to identify additional studies and hand‐searched bibliographies of included studies. Selection criteria Randomised controlled trials (RCTs) evaluating pine bark extract supplements in adults or children with any chronic disorder. Data collection and analysis Two authors independently assessed trial eligibility, extracted data and assessed risk of bias. Where possible, we pooled data in meta‐analyses. We used GRADE to evaluate the certainty of evidence. Primary outcomes were participant‐ and investigator‐reported clinical outcomes directly related to each disorder and all‐cause mortality. We also assessed adverse events and biomarkers of oxidative stress. Main results This review included 27 RCTs (22 parallel and five cross‐over designs; 1641 participants) evaluating pine bark extract supplements across 10 chronic disorders: asthma (two studies; 86 participants); attention deficit hyperactivity disorder (ADHD) (one study; 61 participants), cardiovascular disease (CVD) and risk factors (seven studies; 338 participants), chronic venous insufficiency (CVI) (two studies; 60 participants), diabetes mellitus (DM) (six studies; 339 participants), erectile dysfunction (three studies; 277 participants), female sexual dysfunction (one study; 83 participants), osteoarthritis (three studies; 293 participants), osteopenia (one study; 44 participants) and traumatic brain injury (one study; 60 participants). Two studies exclusively recruited children; the remainder recruited adults. Trials lasted between four weeks and six months. Placebo was the control in 24 studies. Overall risk of bias was low for four, high for one and unclear for 22 studies. In adults with asthma, we do not know whether pine bark extract increases change in forced expiratory volume in one second (FEV 1 ) % predicted/forced vital capacity (FVC) (mean difference (MD) 7.70, 95% confidence interval (CI) 3.19 to 12.21; one study; 44 participants; very low‐certainty evidence), increases change in FEV 1 % predicted (MD 7.00, 95% CI 0.10 to 13.90; one study; 44 participants; very low‐certainty evidence), improves asthma symptoms (risk ratio (RR) 1.85, 95% CI 1.32 to 2.58; one study; 60 participants; very low‐certainty evidence) or increases the number of people able to stop using albuterol inhalers (RR 6.00, 95% CI 1.97 to 18.25; one study; 60 participants; very low‐certainty evidence). In children with ADHD, we do not know whether pine bark extract decreases inattention and hyperactivity assessed by parent‐ and teacher‐rating scales (narrative synthesis; one study; 57 participants; very low‐certainty evidence) or increases the change in visual‐motoric coordination and concentration (MD 3.37, 95% CI 2.41 to 4.33; one study; 57 participants; very low‐certainty evidence). In participants with CVD, we do not know whether pine bark extract decreases diastolic blood pressure (MD ‐3.00 mm Hg, 95% CI ‐4.51 to ‐1.49; one study; 61 participants; very low‐certainty evidence); increases HDL cholesterol (MD 0.05 mmol/L, 95% CI ‐0.01 to 0.11; one study; 61 participants; very low‐certainty evidence) or decreases LDL cholesterol (MD ‐0.03 mmol/L, 95% CI ‐0.05 to 0.00; one study; 61 participants; very low‐certainty evidence). In participants with CVI, we do not know whether pine bark extract decreases pain scores (MD ‐0.59, 95% CI ‐1.02 to ‐0.16; one study; 40 participants; very low‐certainty evidence), increases the disappearance of pain (RR 25.0, 95% CI 1.58 to 395.48; one study; 40 participants; very low‐certainty evidence) or increases physician‐judged treatment efficacy (RR 4.75, 95% CI 1.97 to 11.48; 1 study; 40 participants; very low‐certainty evidence). In type 2 DM, we do not know whether pine bark extract leads to a greater reduction in fasting blood glucose (MD 1.0 mmol/L, 95% CI 0.91 to 1.09; one study; 48 participants;very low‐certainty evidence) or decreases HbA1c (MD ‐0.90 %, 95% CI ‐1.78 to ‐0.02; 1 study; 48 participants; very low‐certainty evidence). In a mixed group of participants with type 1 and type 2 DM we do not know whether pine bark extract decreases HbA1c (MD ‐0.20 %, 95% CI ‐1.83 to 1.43; one study; 67 participants; very low‐certainty evidence). In men with erectile dysfunction, we do not know whether pine bark extract supplements increase International Index of Erectile Function‐5 scores (not pooled; two studies; 147 participants; very low‐certainty evidence). In women with sexual dysfunction, we do not know whether pine bark extract increases satisfaction as measured by the Female Sexual Function Index (MD 5.10, 95% CI 3.49 to 6.71; one study; 75 participants; very low‐certainty evidence) or leads to a greater reduction of pain scores (MD 4.30, 95% CI 2.69 to 5.91; one study; 75 participants; very low‐certainty evidence). In adults with osteoarthritis of the knee, we do not know whether pine bark extract decreases composite Western Ontario and McMaster Universities Osteoarthritis Index scores (MD ‐730.00, 95% CI ‐1011.95 to ‐448.05; one study; 37 participants; very low‐certainty evidence) or the use of non‐steroidal anti‐inflammatory medication (MD ‐18.30, 95% CI ‐25.14 to ‐11.46; one study; 35 participants; very low‐certainty evidence). We do not know whether pine bark extract increases bone alkaline phosphatase in post‐menopausal women with osteopenia (MD 1.16 ug/L, 95% CI ‐2.37 to 4.69; one study; 40 participants; very low‐certainty evidence). In individuals with traumatic brain injury, we do not know whether pine bark extract decreases cognitive failure scores (MD ‐2.24, 95% CI ‐11.17 to 6.69; one study; 56 participants; very low‐certainty evidence) or post‐concussion symptoms (MD ‐0.76, 95% CI ‐5.39 to 3.87; one study; 56 participants; very low‐certainty evidence). For most comparisons, studies did not report outcomes of hospital admissions or serious adverse events. Authors' conclusions Small sample sizes, limited numbers of RCTs per condition, variation in outcome measures, and poor reporting of the included RCTs mean no definitive conclusions regarding the efficacy or safety of pine bark extract supplements are possible. Plain language summary Using pine bark supplements to help treat a variety of chronic diseases Review question Can pine bark antioxidant supplements help to treat chronic diseases? Background The main ingredients in pine bark extract supplements are proanthocyanidins, which are antioxidants. These supplements are marketed to prevent or treat a wide range of chronic diseases. This is an update of a previously published review, where we assessed how well these supplements work for treating chronic diseases, as well as their safety. Search date The evidence is current to 30 September 2019. Study characteristics We included 27 studies (1641 participants) across 10 chronic diseases. These included: asthma (two studies; 86 participants), attention deficit hyperactivity disorder (ADHD) (one study; 61 participants), heart disease and risk factors (seven studies; 332 participants), chronic venous insufficiency (leg veins not working effectively; two studies; 60 participants), diabetes (six studies; 336 participants), erectile dysfunction (three studies; 227 participants), female sexual dysfunction (one study; 75 participants), osteoarthritis (cartilage damage in joints; three studies; 293 participants), osteopenia (beginning of bone mass loss; one study; 44 participants) and traumatic brain injury (one study; 56 participants). Two of the studies were conducted exclusively in children; the others were in adults. Studies compared pine bark supplements with placebo (i.e. a dummy supplement) or non‐antioxidant interventions and participants were randomly selected for one treatment or the other. The duration of treatment ranged from four weeks to six months. Key results For most outcomes across the different chronic diseases we only included one study with a small number of participants. In adults with asthma, we do not know whether pine bark extract increases lung function, improves asthma symptoms or increases the number of people able to stop using albuterol inhalers (a specific type of asthma pump). In children with ADHD, we do not know whether pine bark supplements decrease inattention and hyperactivity (assessed by parents and teachers) or whether it increases co‐ordination and concentration. In people with heart disease we do not know if pine bark supplements decrease blood pressure and LDL cholesterol (the bad type) or whether it increases HDL cholesterol (the good type). In adults with chronic venous insufficiency we do not know whether pine bark supplements decrease pain, increase the number of people free of pain, or how well doctors think the treatment works. In people with type 2 diabetes, we do not know whether pine bark supplements improve blood sugar levels or HbA1c levels (the long‐term marker for measuring blood sugar control); we also do not know if pine bark supplements decrease HbA1c in people with type 1 or type 2 diabetes. In men with erectile dysfunction, we do not know if pine bark supplements increase erectile function. Similarly, in women with sexual dysfunction, we do not know if pine bark supplements increase sexual satisfaction or reduce pain. In adults with osteoarthritis, it is not clear if pine bark supplements improve pain, swelling or stiffness in the knee, or whether it decreases the use of anti‐inflammatory drugs. We also do not know if pine bark supplements increase bone formation in postmenopausal women with weakened bones. Lastly, in people with a traumatic brain injury, it is not clear if pine bark supplements improve memory and post‐concussion symptoms . For most comparisons, studies did not report outcomes of hospital admissions or serious adverse events. With the available studies, we cannot clearly say how well (or not) pine bark supplements work and whether they are safe. Quality of the evidence The overall risk of bias was low for four studies, high for one and unclear for 22 studies. By this we mean the extent to which the methods used in a study enable it to determine the truth. The certainty of evidence was very low for all outcomes across all chronic diseases in the included studies.

UR - http://dx.doi.org/10.1002/14651858.CD008294.pub5

ER -

Record #20 of 32

Provider: John Wiley & Sons, Ltd

Content: text/plain; charset="UTF-8"

TY - JOUR

AN - CD012556

AU - Goldkuhle, M

AU - Dimaki, M

AU - Gartlehner, G

AU - Monsef, I

AU - Dahm, P

AU - Glossmann, JP

AU - Engert, A

AU - von Tresckow, B

AU - Skoetz, N

TI - Nivolumab for adults with Hodgkin's lymphoma (a rapid review using the software RobotReviewer)

JF - Cochrane Database of Systematic Reviews

PY - 2018

IS - 7

PB - John Wiley & Sons, Ltd

SN - 1465-1858

KW - Adult

KW - Antibodies, Monoclonal [*therapeutic use]

KW - Antineoplastic Agents [*therapeutic use]

KW - Hodgkin Disease [*drug therapy, pathology]

KW - Humans

KW - Nivolumab

KW - Software

N1 - [Haematology]

DO - 10.1002/14651858.CD012556.pub2

AB - Abstract - Background Hodgkin's lymphoma (HL) is a cancer of the lymphatic system, and involves the lymph nodes, spleen and other organs such as the liver, lung, bone or bone marrow, depending on the tumour stage. With cure rates of up to 90%, HL is one of the most curable cancers worldwide. Approximately 10% of people with HL will be refractory to initial treatment or will relapse; this is more common in people with advanced stage or bulky disease. Standard of care for these people is high‐dose chemotherapy and autologous stem cell transplantation (ASCT), but only 55% of participants treated with high‐dose chemotherapy and ASCT are free from treatment failure at three years, with an overall survival (OS) of about 80% at three years. Checkpoint inhibitors that target the interaction of the programmed death (PD)‐1 immune checkpoint receptor, and its ligands PD‐L1 and PD‐L2, have shown remarkable activity in a wide range of malignancies. Nivolumab is an anti‐(PD)‐1 monoclonal antibody and currently approved by the US Food and Drug Administration (FDA) for the treatment of melanoma, non‐small cell lung cancer, renal cell carcinoma and, since 2016, for classical Hodgkin's lymphoma (cHL) after treatment with ASCT and brentuximab vedotin. Objectives To assess the benefits and harms of nivolumab in adults with HL (irrespective of stage of disease). Search methods We searched CENTRAL, MEDLINE, Embase, International Pharmaceutical Abstracts, conference proceedings and six study registries from January 2000 to May 2018 for prospectively planned trials evaluating nivolumab. Selection criteria We included prospectively planned trials evaluating nivolumab in adults with HL. We excluded trials in which less than 80% of participants had HL, unless the trial authors provided the subgroup data for these participants in the publication or after we contacted the trial authors. Data collection and analysis Two review authors independently extracted data and assessed potential risk of bias. We used the software RobotReviewer to extract data and compared results with our findings. As we did not identify any randomised controlled trials (RCTs) or non‐RCTs, we did not meta‐analyse data. Main results Our search found 782 potentially relevant references. From these, we included three trials without a control group, with 283 participants. In addition, we identified 14 ongoing trials evaluating nivolumab, of which two are randomised. Risk of bias of the three included studies was moderate to high. All of the participants were in relapsed stage, most of them were heavily pretreated and had received at least two previous treatments, most of them had also undergone ASCT. As we did not identify any RCTs, we could not use the software RobotReviewer to assess risk of bias. The software identified correctly that one study was not an RCT and did not extract any trial data, but extracted characteristics of the other two studies (although also not RCTs) in a sufficient way. Two studies with 260 participants evaluated OS. After six months, OS was 100% in one study and median OS (the timepoint when only 50% of participants were alive) was not reached in the other trial after a median follow‐up of 18 months (interquartile range (IQR) 15 to 22 months) (very low certainty evidence, due to observational trial design, heterogenous patient population in terms of pretreatments and various follow‐up times (downgrading by 1 point)). In one study, one out of three cohorts reported quality of life. It was unclear whether there was an effect on quality of life as only a subset of participants filled out the follow‐up questionnaire (very low certainty evidence). Three trials (283 participants) evaluated progression‐free survival (PFS) (very low certainty evidence). Six‐month PFS ranged between 60% and 86%, and median PFS ranged between 12 and 18 months. All three trials (283 participants) reported complete response rates, ranging from 12% to 29%, depending on inclusion criteria and participants' previous treatments (very low certainty evidence). One trial (243 participants) reported drug‐related grade 3 or 4 adverse events (AEs) only after a median follow‐up of 18 months (IQR 15 to 22 months); these were fatigue (23%), diarrhoea (15%), infusion reactions (14%) and rash (12%). The other two trials (40 participants) reported 23% to 52% grade 3 or 4 AEs after six months' follow‐up (very low certainty evidence). Only one trial (243 participants) reported drug‐related serious AEs; 2% of participants developed infusion reactions and 1% pneumonitis (very low certainty evidence). None of the studies reported treatment‐related mortality. Authors' conclusions To date, data on OS, quality of life, PFS, response rate, or short‐ and long‐term AEs are available from small uncontrolled trials only. The three trials included heavily pretreated participants, which had previously undergone regimens of BV or ASCT. For these participants, median OS was not reached after follow‐up times of at least 16 months (more than 50% of participants with a limited life expectancy were alive at this timepoint). Only one cohort out of three only reported quality of life, with limited follow‐up data so that meaningful conclusions were not possible. Serious adverse events occurred rarely. Currently, data are too sparse to make a clear statement on nivolumab for people with relapsed or refractory HL except for heavily pretreated people, which had previously undergone regimens of BV or ASCT. When interpreting these results, it is important to consider that proper RCTs should confirm these findings. As there are 14 ongoing trials evaluating nivolumab, of which two are RCTs, it is possible that an update of this review will be published in the near future and that this update will show different results to those reported here. Plain language summary Nivolumab for adults with Hodgkin's lymphoma Background Hodgkin's lymphoma (HL) is a cancer of the lymphatic system. As part of the immune system, the lymphatic system comprises a network of lymphatic vessels, which transport lymph throughout the body. Lymph is a fluid which contains white blood cells, that tackle infection. HL occurs in children and adults, but it is more common in the third decade of life. It is one of the most curable forms of cancer and up to 90% of people will be cured; however, approximately 10% of people with HL will relapse (the cancer will return). Treatment options are chemotherapy, radiotherapy, or both, or newly developed agents, called checkpoint inhibitors that target the cancer cell directly. Nivolumab is one checkpoint inhibitor and currently approved by the US Food and Drug Administration for the treatment of various cancers and relapsed HL after treatment with stem cell transplantation and brentuximab vedotin, which is a medicine used to treat cancer. In stem cell transplantation patients receive blood building cells, so called stem cells, which replace their own when they have been destroyed along the disease or previous therapy regimens. Review question This systematic review evaluated the benefits and harms of nivolumab for adults with Hodgkin's lymphoma. Study characteristics We searched important medical databases for clinical trials assessing the benefits and harms of nivolumab in adults with HL. Two review authors independently screened, summarised and analysed the results. In addition, we tested the computer software RobotReviewer to extract data. Our search led to the inclusion of three studies involving 283 participants and 14 ongoing trials. The evidence provided is current to May 2018. Key results Two studies with 260 participants evaluated survival. After six months, all participants were alive in one trial (17 participants). One trial reported quality of life for a subgroup of participants using a questionnaire but not all follow‐up data were available. Although it seemed that the participants answering the questionnaire might have had a benefit, it was unclear whether this applied to all the participants. The studies also reported tumour control and tumour response, but with different results, depending on the treatment and how many previous treatments participants had received before nivolumab was given. As nivolumab is given until the disease progresses (gets worse) or until unacceptable side effects occur, people receive the drug for a long time. Therefore, reporting of side effects is related to the time the person received the medicine, with potentially more side effects with longer usage. The most commonly reported side effects were fatigue (tiredness), diarrhoea (loose stools), infusion reactions (during or shortly after giving the medicine by a vein) and rash. Only one study reported medicine‐related serious side effects. They occurred rarely (infusion reactions and lung disease). Deaths related to the medicine were not reported. Reliability of the evidence Due to the study design and varied type of participants with different numbers of previous treatments and various treatment options, the reliability of the evidence was low to very low. Conclusion This systematic review evaluated the benefits and harms of nivolumab in adults with HL. Data on survival, quality of life, tumour response and side effects were available from small trials only. The three trials included only people different previous treatment options, very often also with a previous stem cell transplantation. In one trial, all participants were alive after six months. Quality of life data were not reported for all the included participants; moreover, data after a long period of treatment were not available for all evaluated participants, therefore meaningful conclusions were not possible. Serious side effects occurred rarely. Currently, data are too sparse to make a clear statement on nivolumab for people with relapsed or refractory HL except for those who had received several treatments before. As there are currently 14 ongoing trials evaluating nivolumab, of which two are well designed, it is possible that an update of this review will be published in the near future and that this update will show different results to those reported here.

UR - http://dx.doi.org/10.1002/14651858.CD012556.pub2

ER -

Record #21 of 32

Provider: John Wiley & Sons, Ltd

Content: text/plain; charset="UTF-8"

TY - JOUR

AN - CD010429

AU - Bhardwaj, A

AU - Swe, KMM

AU - Sinha, NK

AU - Osunkwo, I

TI - Treatment for osteoporosis in people with ß‐thalassaemia

JF - Cochrane Database of Systematic Reviews

PY - 2016

IS - 3

PB - John Wiley & Sons, Ltd

SN - 1465-1858

KW - Adolescent

KW - Adult

KW - Alendronate [therapeutic use]

KW - Bone Density Conservation Agents [*therapeutic use]

KW - Bone Density [drug effects]

KW - Child

KW - Clodronic Acid [therapeutic use]

KW - Diphosphonates [*therapeutic use]

KW - Female

KW - Femur Neck [drug effects]

KW - Humans

KW - Male

KW - Middle Aged

KW - Osteoporosis [*drug therapy]

KW - Randomized Controlled Trials as Topic

KW - Zinc Sulfate [*therapeutic use]

KW - beta‐Thalassemia [*complications]

N1 - [Cystic Fibrosis and Genetic Disorders]

DO - 10.1002/14651858.CD010429.pub2

AB - Abstract - Background Osteoporosis is a systemic skeletal disease characterized by low bone mass and micro‐architectural deterioration of bone tissue with a consequent increase in bone fragility and susceptibility to fracture. Osteoporosis represents an important cause of morbidity in people with beta‐thalassaemia and its pathogenesis is multifactorial. Factors include bone marrow expansion due to ineffective erythropoiesis, resulting in reduced trabecular bone tissue with cortical thinning; endocrine dysfunction secondary to excessive iron loading, leading to increased bone turnover; and lastly, a predisposition to physical inactivity due to disease complications with a subsequent reduction in optimal bone mineralization. A number of therapeutic strategies have been applied to treat osteoporosis in people with beta‐thalassaemia, which include bisphosphonates, with or without, hormone replacement therapy. There are various forms of bisphosphonates, such as clodronate, pamidronate, alendronate and zoledronic acid. Other treatments include calcitonin, calcium, zinc supplementation, hydroxyurea and hormone replacement therapy for preventing hypogonadism. Objectives To review the evidence on the efficacy and safety of treatment for osteoporosis in people with beta‐thalassaemia. Search methods We searched the Cochrane Cystic Fibrosis and Genetic Disorders Group’s Haemoglobinopathies Trials Register comprising references identified from comprehensive electronic database searches and handsearches of relevant journals and abstract books of conference proceedings. Date of most recent search: 04 February 2016. Selection criteria Randomised, placebo‐controlled trials in people with thalassaemia with a bone mineral density z score of less than ‐2 standard deviations for: children less than 15 years old; adult males (15 to 50 years old); and all pre‐menopausal females above 15 years and a bone mineral density t score of less than ‐2.5 standard deviations for post‐menopausal females and males above 50 years old. Data collection and analysis Two review authors assessed the eligibility and risk of bias of the included trials, extracted and analysed data and completed the review. We summarised results using risk ratios or rate ratios for dichotomous data and mean differences for continuous data. We combined trial results where appropriate. Main results Four trials (with 211 participants) were included; three trials investigated the effect of bisphosphonate therapies and one trial investigated the effect of zinc supplementation. Only one trial was judged to be of good quality (low risk of bias); the remaining trials had a high or unclear risk of bias in at least one key domain. One trial (data not available for analysis) assessing the effect of neridronate (118 participants) reported significant increases in favour of the bisphosphonate group for bone mineral density at the lumbar spine and hip at both six and 12 months. For the femoral neck, a significant difference was noted at 12 months only. A further trial (25 participants) assessed the effect of alendronate and clodronate and found that after two years, bone mineral density increased significantly in the alendronate and clodronate groups as compared to placebo at the lumbar spine, mean difference 0.14 g/cm 2 (95% confidence interval 0.05 to 0.22) and at the femoral neck, mean difference 0.40 g/cm 2 (95% confidence interval 0.22 to 0.57). One 12‐month trial (26 participants) assessed the effects of different doses of pamidronate (30 mg versus 60 mg) and found a significant difference in bone mineral density in favour of the 60 mg dose at the lumbar spine and forearm, mean difference 0.43 g/cm 2 (95% CI 0.10 to 0.76), mean difference 0.87 g/cm 2 (95% CI 0.23 to 1.51), respectively, but not at the femoral neck. In a zinc sulphate supplementation trial (42 participants), bone mineral density increased significantly compared to placebo at the lumbar spine after 12 months (37 participants), mean difference 0.15 g/cm 2 (95% confidence interval 0.10 to 0.20) and after 18 months (32 participants), mean difference 0.34 g/cm 2 (95% confidence interval 0.28 to 0.40). The same was true for bone mineral density at the hip after 12 months, mean difference 0.15 g/cm 2 (95% confidence interval 0.11 to 0.19) and after 18 months, mean difference 0.26 g/cm 2 (95% confidence interval 0.21 to 0.31). Fractures were not observed in one trial and not reported in three trials. There were no major adverse effects reported in two of the bisphosphonate trials; in the neridronate trial there was a reduction noted in the use of analgesic drugs and in the reported back pain score in favour of bisphosphonate treatment. Adverse effects were not reported in the trial of different doses of pamidronate or the zinc supplementation trial. Authors' conclusions There is evidence to indicate an increase in bone mineral density at the femoral neck, lumbar spine and forearm after administration of bisphosphonates and at the lumbar spine and hip after zinc sulphate supplementation . The authors recommend that further long‐term randomised control trials on different bisphosphonates and zinc supplementation therapies in people with beta‐thalassaemia and osteoporosis are undertaken. Plain language summary Treatment of osteoporosis in people with beta thalassaemia Review question We reviewed the evidence on the effects and safety of different treatments of osteoporosis in people with beta‐thalassaemia. Background Osteoporosis affects bone density over time and leads to an increased risk of fractures. It is an important cause of illness in people with beta‐thalassaemia. We wanted to find the most effective available treatment for treating osteoporosis and improving quality of life. Search date The evidence is current to: 04 February 2016. Trial characteristics The review included four trials with 211 people with beta‐thalassaemia aged between 10 years and 58 years of age . Trials compared bisphosphonates (alendronate, clodronate and neridronate and pamidronate) and zinc sulphate to either control groups or, as in one trial a different dose of treatment, with people selected for one treatment or the other randomly. Three trials were carried out for two years, with one currently only reporting 12‐month data and one trial was carried out for 12 months. Key results Three trials assessed bisphosphonate therapy, in one trial (118 volunteers) there was an increase (in favour of the treatment group) in bone mineral density at the lumbar spine and hip at six and 12 months and at the femoral neck only after 12 months. In a second trial (25 volunteers), bone mineral density at the lumbar spine increased after two years of treatment with alendronate and clodronate. In the 12‐month trial comparing different doses of pamidronate (26 volunteers), the 60 mg group reported a larger increase in bone mineral density at the lumbar spine and forearm than the 30 mg group, but this was not the case at the femoral neck. In a zinc sulphate supplements trial (42 volunteers), bone mineral density increased at the lumbar spine and hip after 12 and 18 months. It was stated that there were no fractures in one trial and this outcome was not reported in three trials. There were no major adverse effects reported in either of the trials comparing bisphosphonates to control; although in the neridronate trial there were fewer painkillers used and a lower score for back pain reported in the bisphosphonate group. Adverse effects were not reported in the bisphosphonate trial comparing different doses or in the zinc sulphate supplements trial. Therefore, evidence indicates an increase in bone mineral density at the femoral neck, lumbar spine and forearm after giving bisphosphonates. This is also true for the lumbar spine and hip after zinc sulphate supplementation . The authors recommend that further long‐term randomised controlled trials looking at different treatments with bisphosphonates and zinc supplementation are carried out on people with beta‐thalassaemia who have osteoporosis. Quality of the evidence The quality of the included trials was mixed. Specifically, although all the trials stated that people received different treatments at random, two of them did not describe exactly how they did this. Also, none of the four trials described how they stopped people knowing which group they were going to be put into.

UR - http://dx.doi.org/10.1002/14651858.CD010429.pub2

ER -

Record #22 of 32

Provider: John Wiley & Sons, Ltd

Content: text/plain; charset="UTF-8"

TY - JOUR

AN - CD012763

AU - Hristovska, AM

AU - Duch, P

AU - Allingstrup, M

AU - Afshari, A

TI - Efficacy and safety of sugammadex versus neostigmine in reversing neuromuscular blockade in adults

JF - Cochrane Database of Systematic Reviews

PY - 2017

IS - 8

PB - John Wiley & Sons, Ltd

SN - 1465-1858

KW - *Neuromuscular Blockade

KW - Adult

KW - Androstanols [antagonists & inhibitors]

KW - Atracurium [analogs & derivatives, antagonists & inhibitors]

KW - Cholinesterase Inhibitors [administration & dosage, adverse effects, *pharmacology]

KW - Humans

KW - Neostigmine [administration & dosage, adverse effects, *pharmacology]

KW - Neuromuscular Nondepolarizing Agents [*antagonists & inhibitors]

KW - Randomized Controlled Trials as Topic

KW - Rocuronium

KW - Sugammadex

KW - Time Factors

KW - Vecuronium Bromide [antagonists & inhibitors]

KW - gamma‐Cyclodextrins [administration & dosage, adverse effects, *pharmacology]

N1 - [Anaesthesia]

DO - 10.1002/14651858.CD012763

AB - Abstract - Background Acetylcholinesterase inhibitors, such as neostigmine, have traditionally been used for reversal of non‐depolarizing neuromuscular blocking agents. However, these drugs have significant limitations, such as indirect mechanisms of reversal, limited and unpredictable efficacy, and undesirable autonomic responses. Sugammadex is a selective relaxant‐binding agent specifically developed for rapid reversal of non‐depolarizing neuromuscular blockade induced by rocuronium. Its potential clinical benefits include fast and predictable reversal of any degree of block, increased patient safety, reduced incidence of residual block on recovery, and more efficient use of healthcare resources. Objectives The main objective of this review was to compare the efficacy and safety of sugammadex versus neostigmine in reversing neuromuscular blockade caused by non‐depolarizing neuromuscular agents in adults. Search methods We searched the following databases on 2 May 2016: Cochrane Central Register of Controlled Trials (CENTRAL); MEDLINE (WebSPIRS Ovid SP), Embase (WebSPIRS Ovid SP), and the clinical trials registries www.controlled‐trials.com , clinicaltrials.gov , and www.centerwatch.com . We re‐ran the search on 10 May 2017. Selection criteria We included randomized controlled trials (RCTs) irrespective of publication status, date of publication, blinding status, outcomes published, or language. We included adults, classified as American Society of Anesthesiologists (ASA) I to IV, who received non‐depolarizing neuromuscular blocking agents for an elective in‐patient or day‐case surgical procedure. We included all trials comparing sugammadex versus neostigmine that reported recovery times or adverse events. We included any dose of sugammadex and neostigmine and any time point of study drug administration. Data collection and analysis Two review authors independently screened titles and abstracts to identify trials for eligibility, examined articles for eligibility, abstracted data, assessed the articles, and excluded obviously irrelevant reports. We resolved disagreements by discussion between review authors and further disagreements through consultation with the last review author. We assessed risk of bias in 10 methodological domains using the Cochrane risk of bias tool and examined risk of random error through trial sequential analysis. We used the principles of the GRADE approach to prepare an overall assessment of the quality of evidence. For our primary outcomes (recovery times to train‐of‐four ratio (TOFR) > 0.9), we presented data as mean differences (MDs) with 95 % confidence intervals (CIs), and for our secondary outcomes (risk of adverse events and risk of serious adverse events), we calculated risk ratios (RRs) with CIs. Main results We included 41 studies (4206 participants) in this updated review, 38 of which were new studies. Twelve trials were eligible for meta‐analysis of primary outcomes (n = 949), 28 trials were eligible for meta‐analysis of secondary outcomes (n = 2298), and 10 trials (n = 1647) were ineligible for meta‐analysis. We compared sugammadex 2 mg/kg and neostigmine 0.05 mg/kg for reversal of rocuronium‐induced moderate neuromuscular blockade (NMB). Sugammadex 2 mg/kg was 10.22 minutes (6.6 times) faster then neostigmine 0.05 mg/kg (1.96 vs 12.87 minutes) in reversing NMB from the second twitch (T2) to TOFR > 0.9 (MD 10.22 minutes, 95% CI 8.48 to 11.96; I 2 = 84%; 10 studies, n = 835; GRADE: moderate quality). We compared sugammadex 4 mg/kg and neostigmine 0.07 mg/kg for reversal of rocuronium‐induced deep NMB. Sugammadex 4 mg/kg was 45.78 minutes (16.8 times) faster then neostigmine 0.07 mg/kg (2.9 vs 48.8 minutes) in reversing NMB from post‐tetanic count (PTC) 1 to 5 to TOFR > 0.9 (MD 45.78 minutes, 95% CI 39.41 to 52.15; I 2 = 0%; two studies, n = 114; GRADE: low quality). For our secondary outcomes, we compared sugammadex, any dose, and neostigmine, any dose, looking at risk of adverse and serious adverse events. We found significantly fewer composite adverse events in the sugammadex group compared with the neostigmine group (RR 0.60, 95% CI 0.49 to 0.74; I 2 = 40%; 28 studies, n = 2298; GRADE: moderate quality). Risk of adverse events was 28% in the neostigmine group and 16% in the sugammadex group, resulting in a number needed to treat for an additional beneficial outcome (NNTB) of 8. When looking at specific adverse events, we noted significantly less risk of bradycardia (RR 0.16, 95% CI 0.07 to 0.34; I 2 = 0%; 11 studies, n = 1218; NNTB 14; GRADE: moderate quality), postoperative nausea and vomiting (PONV) (RR 0.52, 95% CI 0.28 to 0.97; I 2 = 0%; six studies, n = 389; NNTB 16; GRADE: low quality) and overall signs of postoperative residual paralysis (RR 0.40, 95% CI 0.28 to 0.57; I 2 = 0%; 15 studies, n = 1474; NNTB 13; GRADE: moderate quality) in the sugammadex group when compared with the neostigmine group. Finally, we found no significant differences between sugammadex and neostigmine regarding risk of serious adverse events (RR 0.54, 95% CI 0.13 to 2.25; I 2 = 0%; 10 studies, n = 959; GRADE: low quality). Application of trial sequential analysis (TSA) indicates superiority of sugammadex for outcomes such as recovery time from T2 to TOFR > 0.9, adverse events, and overall signs of postoperative residual paralysis. Authors' conclusions Review results suggest that in comparison with neostigmine, sugammadex can more rapidly reverse rocuronium‐induced neuromuscular block regardless of the depth of the block. Sugammadex 2 mg/kg is 10.22 minutes (˜ 6.6 times) faster in reversing moderate neuromuscular blockade (T2) than neostigmine 0.05 mg/kg (GRADE: moderate quality), and sugammadex 4 mg/kg is 45.78 minutes (˜ 16.8 times) faster in reversing deep neuromuscular blockade (PTC 1 to 5) than neostigmine 0.07 mg/kg (GRADE: low quality). With an NNTB of 8 to avoid an adverse event, sugammadex appears to have a better safety profile than neostigmine. Patients receiving sugammadex had 40% fewer adverse events compared with those given neostigmine. Specifically, risks of bradycardia (RR 0.16, NNTB 14; GRADE: moderate quality), PONV (RR 0.52, NNTB 16; GRADE: low quality), and overall signs of postoperative residual paralysis (RR 0.40, NNTB 13; GRADE: moderate quality) were reduced. Both sugammadex and neostigmine were associated with serious adverse events in less than 1% of patients, and data showed no differences in risk of serious adverse events between groups (RR 0.54; GRADE: low quality). Plain language summary Benefits and harms of sugammadex versus neostigmine in reversing induced paralysis Background Different levels of induced paralysis are sometimes necessary when patients are put to sleep or are prepared for operations. When the operation is finished, paralysis should be reversed in a fast, reliable, and safe way. Neostigmine is a medication that is traditionally used to reverse induced paralysis. However, its use can be associated with incomplete or slow reversal as well as changes in lung function, heart function, and vomiting and nausea. Sugammadex is a relatively new medication specifically designed to reverse rocuronium‐induced paralysis in a faster, more reliable, and safer way when compared with neostigmine. Objective This review systematically sets out to compare the benefits and harms of sugammadex and neostigmine. The evidence is current up to May 2017. Study characteristics We identified 41 randomized controlled trials comparing sugammadex with neostigmine that provided suitable data on efficacy and safety. All of these trials included adults undergoing surgery and involved a total of 4206 participants. Key results Data indicate that sugammadex was 10.22 minutes (6.6 times) faster than neostigmine (1.96 vs 12.87 minutes) in reversing moderate induced paralysis. Sugammadex was 45.78 minutes (16.8 times) faster than neostigmine (2.9 vs 48.8 minutes) in reversing deep induced paralysis. Participants receiving sugammadex appeared to have a 40% reduced risk of experiencing harmful events than those given neostigmine. Statistically, eight persons can be treated with sugammadex as opposed to neostigmine to avoid one person experiencing a single random harmful event. The occurrence of serious harmful events was nearly non‐existent and data show no differences between compared groups. Conclusion Sugammadex is more efficient and safer than neostigmine for reversing moderate and deep induced paralysis. Quality of evidence We consider our overall findings on benefits and harms to provide evidence of moderate quality in favour of sugammadex.

UR - http://dx.doi.org/10.1002/14651858.CD012763

ER -

Record #23 of 32

Provider: John Wiley & Sons, Ltd

Content: text/plain; charset="UTF-8"

TY - JOUR

AN - CD013092

AU - da Silva Lopes, K

AU - Yamaji, N

AU - Rahman, MO

AU - Suto, M

AU - Takemoto, Y

AU - Garcia-Casal, MN

AU - Ota, E

TI - Nutrition‐specific interventions for preventing and controlling anaemia throughout the life cycle: an overview of systematic reviews

JF - Cochrane Database of Systematic Reviews

PY - 2021

IS - 9

PB - John Wiley & Sons, Ltd

SN - 1465-1858

KW - *Anemia [epidemiology, prevention & control]

KW - *Anemia, Iron-Deficiency [epidemiology, prevention & control]

KW - Adolescent

KW - Adult

KW - Aged

KW - Animals

KW - Child

KW - Dietary Supplements

KW - Female

KW - Food, Fortified

KW - Humans

KW - Iron

KW - Life Cycle Stages

KW - Male

KW - Micronutrients

KW - Middle Aged

KW - Pregnancy

KW - Systematic Reviews as Topic

KW - Young Adult

N1 - [Developmental, Psychosocial and Learning Problems]

DO - 10.1002/14651858.CD013092.pub2

AB - Abstract - Background Anaemia is a prevalent health problem worldwide. Some types are preventable or controllable with iron supplementation (pills or drops), fortification (sprinkles or powders containing iron added to food) or improvements to dietary diversity and quality (e.g. education or counselling). Objectives To summarise the evidence from systematic reviews regarding the benefits or harms of nutrition‐specific interventions for preventing and controlling anaemia in anaemic or non‐anaemic, apparently healthy populations throughout the life cycle. Methods In August 2020, we searched MEDLINE, Embase and 10 other databases for systematic reviews of randomised controlled trials (RCTs) in anaemic or non‐anaemic, apparently healthy populations. We followed Cochrane methodology, extracting GRADE ratings where provided. The primary outcomes were haemoglobin (Hb) concentration, anaemia, and iron deficiency anaemia (IDA); secondary outcomes were iron deficiency (ID), severe anaemia and adverse effects (e.g. diarrhoea, vomiting). Main results We included 75 systematic reviews, 33 of which provided GRADE assessments; these varied between high and very low. Infants (6 to 23 months; 13 reviews) Iron supplementation increased Hb levels and reduced the risk of anaemia and IDA in two reviews. Iron fortification of milk or cereals, multiple‐micronutrient powder (MMNP), home fortification of complementary foods, and supplementary feeding increased Hb levels and reduced the risk of anaemia in six reviews. In one review, lipid‐based nutrient supplementation (LNS) reduced the risk of anaemia. In another, caterpillar cereal increased Hb levels and reduced IDA prevalence. Food‐based strategies (red meat and fortified cow's milk, beef) showed no evidence of a difference (1 review). Preschool and school‐aged children (2 to 10 years; 8 reviews) Daily or intermittent iron supplementation increased Hb levels and reduced the risk of anaemia and ID in two reviews. One review found no evidence of difference in Hb levels, but an increased risk of anaemia and ID for the intermittent regime. All suggested that zinc plus iron supplementation versus zinc alone, multiple‐micronutrient (MMN)‐fortified beverage versus control, and point‐of‐use fortification of food with iron‐containing micronutrient powder (MNP) versus placebo or no intervention may increase Hb levels and reduce the risk of anaemia and ID. Fortified dairy products and cereal food showed no evidence of a difference on the incidence of anaemia (1 review). Adolescent children (11 to 18 years; 4 reviews) Compared with no supplementation or placebo, five types of iron supplementation may increase Hb levels and reduce the risk of anaemia (3 reviews). One review on prevention found no evidence of a difference in anaemia incidence on iron supplementation with or without folic acid, but Hb levels increased. Another suggested that nutritional supplementation and counselling reduced IDA. One review comparing MMN fortification with no fortification observed no evidence of a difference in Hb levels. Non‐pregnant women of reproductive age (19 to 49 years; 5 reviews) Two reviews suggested that iron therapy (oral, intravenous (IV), intramuscular (IM)) increased Hb levels; one showed that iron folic acid supplementation reduced anaemia incidence; and another that daily iron supplementation with or without folic acid or vitamin C increased Hb levels and reduced the risk of anaemia and ID. No review reported interventions related to fortification or dietary diversity and quality. Pregnant women of reproductive age (15 to 49 years; 23 reviews) One review apiece suggested that: daily iron supplementation with or without folic acid increased Hb levels in the third trimester or at delivery and in the postpartum period, and reduced the risk of anaemia, IDA and ID in the third trimester or at delivery; intermittent iron supplementation had no effect on Hb levels and IDA, but increased the risk of anaemia at or near term and ID, and reduced the risk of side effects; vitamin A supplementation alone versus placebo, no intervention or other micronutrient might increase maternal Hb levels and reduce the risk of maternal anaemia; MMN with iron and folic acid versus placebo reduced the risk of anaemia; supplementation with oral bovine lactoferrin versus oral ferrous iron preparations increased Hb levels and reduced gastrointestinal side effects; MNP for point‐of‐use fortification of food versus iron and folic acid supplementation might decrease Hb levels at 32 weeks' gestation and increase the risk of anaemia; and LNS versus iron or folic acid and MMN increased the risk of anaemia. Mixed population (all ages; 22 reviews) Iron supplementation versus placebo or control increased Hb levels in healthy children, adults, and elderly people (4 reviews). Hb levels appeared to increase and risk of anaemia and ID decrease in two reviews investigating MMN fortification versus placebo or no treatment, iron fortified flour versus control, double fortified salt versus iodine only fortified salt, and rice fortification with iron alone or in combination with other micronutrients versus unfortified rice or no intervention. Each review suggested that fortified versus non‐fortified condiments or noodles, fortified (sodium iron ethylenediaminetetraacetate; NaFeEDTA) versus non‐fortified soy sauce, and double‐fortified salt versus control salt may increase Hb concentration and reduce the risk of anaemia. One review indicated that Hb levels increased for children who were anaemic or had IDA and received iron supplementation, and decreased for those who received dietary interventions. Another assessed the effects of foods prepared in iron pots, and found higher Hb levels in children with low‐risk malaria status in two trials, but no difference when comparing food prepared in non‐cast iron pots in a high‐risk malaria endemicity mixed population. There was no evidence of a difference for adverse effects. Anaemia and malaria prevalence were rarely reported. No review focused on women aged 50 to 65 years plus or men (19 to 65 years plus). Authors' conclusions Compared to no treatment, daily iron supplementation may increase Hb levels and reduce the risk of anaemia and IDA in infants, preschool and school‐aged children and pregnant and non‐pregnant women. Iron fortification of foods in infants and use of iron pots with children may have prophylactic benefits for malaria endemicity low‐risk populations. In any age group, only a limited number of reviews assessed interventions to improve dietary diversity and quality. Future trials should assess the effects of these types of interventions, and consider the requirements of different populations. Plain language summary Interventions throughout life for the prevention or treatment of anaemia What is the issue? Anaemia (low iron levels in the blood) is a health problem worldwide, caused by nutritional (e.g. nutrient deficiencies) or non‐nutritional (e.g. diseases or genetic disorders) factors. Its health consequences include fatigue, loss of productivity and adverse pregnancy and child outcomes. Why is this important? Iron deficiency (ID) is a common cause of nutritional anaemia, resulting from a lack of iron in the diet or reduced absorption of iron in the body (e.g. components in coffee, tea or cocoa inhibit iron absorption, while beverages and foods high in vitamin C, such as fruits and vegetables, enhance iron absorption). Some types of anaemia are preventable or controllable with iron supplementation (via capsules or drops), fortification (food enriched with sprinkles or powders containing iron) or improvements to diet diversity and quality (e.g. education or counselling). What evidence did we find? Infants (6 to 23 months) Two reviews suggested that iron supplementation increased haemoglobin (Hb) levels, and reduced the risk of anaemia and iron deficiency anaemia (IDA) compared with placebo, no intervention or other interventions. Six reviews suggested that iron fortification of milk or cereals, multiple‐micronutrient powder (MMNP), home fortification of complementary foods and supplementary feeding increased Hb levels and reduced the risk of anaemia. In one review apiece, lipid‐based nutrient supplementation (LNS) reduced the risk of anaemia, while caterpillar cereal increased Hb levels and reduced IDA prevalence. Preschool and school‐aged children (2 to 10 years) Two reviews suggested that daily or intermittent (e.g. 1 to 3 times per week) iron supplementation increased Hb levels and reduced the risk of anaemia and ID. For daily versus intermittent iron supplementation, one review found no difference in Hb levels, but an increased risk of anaemia and ID for the intermittent regime. One review apiece found higher Hb levels and reduced risk of anaemia and ID for zinc plus iron supplementation versus zinc alone, multiple‐micronutrient (MMN)‐fortified beverages, and point‐of‐use fortification of food with iron‐containing micronutrient powder (MNP). Adolescent children (11 to 18 years) Three reviews for prevention or treatment suggested that intermittent iron supplementation alone or in combination with other micronutrients, iron supplementation with or without folic acid supplementation, or other micronutrient supplementation increased Hb levels and reduced the risk of anaemia. One review suggested that nutritional supplementation and counselling reduced IDA. In one review for prevention, iron supplementation with or without folic acid appeared to increase Hb levels but have no effect on the incidence of anaemia. Non‐pregnant women of reproductive age (19 to 49 years) Two reviews suggested that iron therapy (oral, intravenous, intramuscular) increased Hb levels. One review found that intravenous iron increased Hb levels compared with oral iron, and another that daily iron supplementation with or without folic acid or vitamin C increased Hb levels and reduced the risk of anaemia and ID. Pregnant women of reproductive age (15 to 49 years) In one review, daily iron supplementation with or without folic acid increased Hb levels in the third trimester or at delivery, and in the postpartum period, and reduced the risk of anaemia, IDA and ID in the third trimester or at delivery. Six reviews suggested that intravenous iron versus oral iron or intramuscular iron increased Hb levels. In one review, vitamin A supplementation alone versus placebo, no intervention or other micronutrient increased Hb levels and reduced the risk of anaemia for the mother. One review found that supplementation with oral bovine lactoferrin versus oral ferrous iron preparations increased Hb levels and reduced gastrointestinal side effects. In one review, compared to iron or folic acid and MMNs, LNS increased the risk of anaemia. Mixed population (all ages) Iron supplementation versus placebo or control increased Hb levels in healthy children, adults, and elderly people in four reviews. In two reviews, MMN fortification versus placebo or no treatment increased Hb levels in children, as did iron supplementation, but Hb levels decreased for those receiving dietary interventions. Intravenous iron resulted in higher Hb levels than oral iron in one review. In another, vitamin B 12 or folic acid supplementation did not increase Hb levels. Each review suggested that iron fortification of food, iron‐fortified soy sauce, double‐fortified salt with iron and iodine, and fortified condiments or noodles increased Hb levels and reduced the risk of anaemia. In one review, foods prepared in iron pots showed the potential to increase Hb levels in children. No review focused on older adult women (50 to 65 years plus) or men (19 to 65 years plus), and anaemia and malaria prevalence were rarely reported. What does this mean? Compared to no treatment, daily iron supplementation may increase Hb levels and reduce the risk of anaemia and IDA in infants, preschool and school‐aged children and pregnant and non‐pregnant women. Iron fortification of foods in infants and use of iron pots with children may have benefits for low‐risk populations. Many trials reported the effects of supplementations, but very few reviews focused on fortification or improving diet diversity and quality. Future trials should focus on different types of interventions to increase food variety and dietary quality.

UR - http://dx.doi.org/10.1002/14651858.CD013092.pub2

ER -

Record #24 of 32

Provider: John Wiley & Sons, Ltd

Content: text/plain; charset="UTF-8"

TY - JOUR

AN - CD012179

AU - Nisenblat, V

AU - Bossuyt, PMM

AU - Shaikh, R

AU - Farquhar, C

AU - Jordan, V

AU - Scheffers, CS

AU - Mol, BWJ

AU - Johnson, N

AU - Hull, ML

TI - Blood biomarkers for the non‐invasive diagnosis of endometriosis

JF - Cochrane Database of Systematic Reviews

PY - 2016

IS - 5

PB - John Wiley & Sons, Ltd

SN - 1465-1858

KW - Adult

KW - Autoantibodies [blood]

KW - Biomarkers [*blood]

KW - CA-125 Antigen [blood]

KW - CA-19-9 Antigen [blood]

KW - Endometriosis [*diagnosis]

KW - Endometrium [immunology]

KW - Female

KW - Humans

KW - Interleukin-6 [blood]

KW - Ovarian Diseases [*diagnosis]

KW - Pelvis

KW - Peritoneal Diseases [*diagnosis]

KW - Randomized Controlled Trials as Topic

N1 - [Gynaecology and Fertility]

DO - 10.1002/14651858.CD012179

AB - Abstract - Background About 10% of reproductive‐aged women suffer from endometriosis, a costly chronic disease causing pelvic pain and subfertility. Laparoscopy is the gold standard diagnostic test for endometriosis, but is expensive and carries surgical risks. Currently, there are no non‐invasive or minimally invasive tests available in clinical practice to accurately diagnose endometriosis. Although other reviews have assessed the ability of blood tests to diagnose endometriosis, this is the first review to use Cochrane methods, providing an update on the rapidly expanding literature in this field. Objectives To evaluate blood biomarkers as replacement tests for diagnostic surgery and as triage tests to inform decisions on surgery for endometriosis. Specific objectives include: 1. To provide summary estimates of the diagnostic accuracy of blood biomarkers for the diagnosis of peritoneal, ovarian and deep infiltrating pelvic endometriosis, compared to surgical diagnosis as a reference standard. 2. To assess the diagnostic utility of biomarkers that could differentiate ovarian endometrioma from other ovarian masses. Search methods We did not restrict the searches to particular study designs, language or publication dates. We searched CENTRAL to July 2015, MEDLINE and EMBASE to May 2015, as well as these databases to 20 April 2015: CINAHL, PsycINFO, Web of Science, LILACS, OAIster, TRIP, ClinicalTrials.gov, DARE and PubMed. Selection criteria We considered published, peer‐reviewed, randomised controlled or cross‐sectional studies of any size, including prospectively collected samples from any population of reproductive‐aged women suspected of having one or more of the following target conditions: ovarian, peritoneal or deep infiltrating endometriosis (DIE). We included studies comparing the diagnostic test accuracy of one or more blood biomarkers with the findings of surgical visualisation of endometriotic lesions. Data collection and analysis Two authors independently collected and performed a quality assessment of data from each study. For each diagnostic test, we classified the data as positive or negative for the surgical detection of endometriosis, and we calculated sensitivity and specificity estimates. We used the bivariate model to obtain pooled estimates of sensitivity and specificity whenever sufficient datasets were available. The predetermined criteria for a clinically useful blood test to replace diagnostic surgery were a sensitivity of 0.94 and a specificity of 0.79 to detect endometriosis. We set the criteria for triage tests at a sensitivity of ≥ 0.95 and a specificity of ≥ 0.50, which 'rules out' the diagnosis with high accuracy if there is a negative test result (SnOUT test), or a sensitivity of ≥ 0.50 and a specificity of ≥ 0.95, which 'rules in' the diagnosis with high accuracy if there is a positive result (SpIN test). Main results We included 141 studies that involved 15,141 participants and evaluated 122 blood biomarkers. All the studies were of poor methodological quality. Studies evaluated the blood biomarkers either in a specific phase of the menstrual cycle or irrespective of the cycle phase, and they tested for them in serum, plasma or whole blood. Included women were a selected population with a high frequency of endometriosis (10% to 85%), in which surgery was indicated for endometriosis, infertility work‐up or ovarian mass. Seventy studies evaluated the diagnostic performance of 47 blood biomarkers for endometriosis (44 single‐marker tests and 30 combined tests of two to six blood biomarkers). These were angiogenesis/growth factors, apoptosis markers, cell adhesion molecules, high‐throughput markers, hormonal markers, immune system/inflammatory markers, oxidative stress markers, microRNAs, tumour markers and other proteins. Most of these biomarkers were assessed in small individual studies, often using different cut‐off thresholds, and we could only perform meta‐analyses on the data sets for anti‐endometrial antibodies, interleukin‐6 (IL‐6), cancer antigen‐19.9 (CA‐19.9) and CA‐125. Diagnostic estimates varied significantly between studies for each of these biomarkers, and CA‐125 was the only marker with sufficient data to reliably assess sources of heterogeneity. The mean sensitivities and specificities of anti‐endometrial antibodies (4 studies, 759 women) were 0.81 (95% confidence interval (CI) 0.76 to 0.87) and 0.75 (95% CI 0.46 to 1.00). For IL‐6, with a cut‐off value of > 1.90 to 2.00 pg/ml (3 studies, 309 women), sensitivity was 0.63 (95% CI 0.52 to 0.75) and specificity was 0.69 (95% CI 0.57 to 0.82). For CA‐19.9, with a cut‐off value of > 37.0 IU/ml (3 studies, 330 women), sensitivity was 0.36 (95% CI 0.26 to 0.45) and specificity was 0.87 (95% CI 0.75 to 0.99). Studies assessed CA‐125 at different thresholds, demonstrating the following mean sensitivities and specificities: for cut‐off > 10.0 to 14.7 U/ml: 0.70 (95% CI 0.63 to 0.77) and 0.64 (95% CI 0.47 to 0.82); for cut‐off > 16.0 to 17.6 U/ml: 0.56 (95% CI 0.24, 0.88) and 0.91 (95% CI 0.75, 1.00); for cut‐off > 20.0 U/ml: 0.67 (95% CI 0.50 to 0.85) and 0.69 (95% CI 0.58 to 0.80); for cut‐off > 25.0 to 26.0 U/ml: 0.73 (95% CI 0.67 to 0.79) and 0.70 (95% CI 0.63 to 0.77); for cut‐off > 30.0 to 33.0 U/ml: 0.62 (95% CI 0.45 to 0.79) and 0.76 (95% CI 0.53 to 1.00); and for cut‐off > 35.0 to 36.0 U/ml: 0.40 (95% CI 0.32 to 0.49) and 0.91 (95% CI 0.88 to 0.94). We could not statistically evaluate other biomarkers meaningfully, including biomarkers that were assessed for their ability to differentiate endometrioma from other benign ovarian cysts. Eighty‐two studies evaluated 97 biomarkers that did not differentiate women with endometriosis from disease‐free controls. Of these, 22 biomarkers demonstrated conflicting results, with some studies showing differential expression and others no evidence of a difference between the endometriosis and control groups. Authors' conclusions Of the biomarkers that were subjected to meta‐analysis, none consistently met the criteria for a replacement or triage diagnostic test. A subset of blood biomarkers could prove useful either for detecting pelvic endometriosis or for differentiating ovarian endometrioma from other benign ovarian masses, but there was insufficient evidence to draw meaningful conclusions. Overall, none of the biomarkers displayed enough accuracy to be used clinically outside a research setting. We also identified blood biomarkers that demonstrated no diagnostic value in endometriosis and recommend focusing research resources on evaluating other more clinically useful biomarkers. Plain language summary Blood biomarkers for the non‐invasive diagnosis of endometriosis Review Question How accurate are blood tests in detecting endometriosis? Can any blood test be accurate enough to replace or reduce the need for surgery in the diagnosis of endometriosis? Background Women with endometriosis have endometrial tissue (the tissue that lines the womb and is shed during menstruation) growing outside the womb within the pelvic cavity. This tissue responds to reproductive hormones, causing painful periods, chronic lower abdominal pain and difficulty conceiving. Currently, the only reliable way of diagnosing endometriosis is to perform keyhole surgery and visualise the endometrial deposits inside the abdomen. Because surgery is risky and expensive, we evaluated whether the results of blood tests (blood biomarkers) can help to detect endometriosis non‐invasively. An accurate blood test could lead to the diagnosis of endometriosis without the need for surgery, or it could reduce the need for diagnostic surgery to a group of women who were most likely to have endometriosis. Separate Cochrane reviews from this series evaluate other non‐invasive ways of diagnosing endometriosis using urine, imaging, endometrial and combination tests. Study characteristics The evidence included in this review is current to July 2015. We included 141 studies involving 15,141 participants. All studies evaluated reproductive‐aged women who were undertaking diagnostic surgery because they were suspected of having one or more of the following target conditions: ovarian, peritoneal or deep infiltrating endometriosis (DIE). Cancer antigen‐125 (CA‐125) was the most common blood biomarker studied. Seventy studies evaluated 47 blood biomarkers that were expressed differently in women with and without endometriosis, and 82 studies identified 97 biomarkers that did not distinguish between the two groups. Twenty‐two biomarkers were in both categories. Key results Only four of the assessed biomarkers (anti‐endometrial Abs (anti‐endometrial autoantibodies), interleukin‐6 (IL‐6), CA‐19.9 and CA‐125) were evaluated by enough studies to provide a meaningful assessment of test accuracy. None of these tests was accurate enough to replace diagnostic surgery. Several studies identified biomarkers that might be of value in diagnosing endometriosis, but there are too few reports to be sure of their diagnostic benefit. Overall, there is not enough evidence to recommend testing for any blood biomarker in clinical practice to diagnose endometriosis. Quality of the evidence Generally, the reports were of low methodological quality, and most blood tests were only assessed by a single or a small number of studies. When the same biomarker was studied, there were significant differences in how studies were conducted, the group of women studied and the cut‐offs used to determine a positive result. Future research More high quality research trials are necessary to accurately assess the diagnostic potential of certain blood biomarkers, whose diagnostic value for endometriosis was suggested by a limited number of studies.

UR - http://dx.doi.org/10.1002/14651858.CD012179

ER -

Record #25 of 32

Provider: John Wiley & Sons, Ltd

Content: text/plain; charset="UTF-8"

TY - JOUR

AN - CD012165

AU - Gupta, D

AU - Hull, ML

AU - Fraser, I

AU - Miller, L

AU - Bossuyt, PMM

AU - Johnson, N

AU - Nisenblat, V

TI - Endometrial biomarkers for the non‐invasive diagnosis of endometriosis

JF - Cochrane Database of Systematic Reviews

PY - 2016

IS - 4

PB - John Wiley & Sons, Ltd

SN - 1465-1858

KW - Biomarkers [*analysis]

KW - Endometriosis [*diagnosis]

KW - Endometrium [*chemistry]

KW - Female

KW - Humans

KW - Menstrual Cycle

KW - Menstruation [metabolism]

N1 - [Gynaecology and Fertility]

DO - 10.1002/14651858.CD012165

AB - Abstract - Background About 10% of reproductive‐aged women suffer from endometriosis, which is a costly, chronic disease that causes pelvic pain and subfertility. Laparoscopy is the gold standard diagnostic test for endometriosis, but it is expensive and carries surgical risks. Currently, there are no non‐invasive tests available in clinical practice that accurately diagnose endometriosis. This is the first diagnostic test accuracy review of endometrial biomarkers for endometriosis that utilises Cochrane methodologies, providing an update on the rapidly expanding literature in this field. Objectives To determine the diagnostic accuracy of the endometrial biomarkers for pelvic endometriosis, using a surgical diagnosis as the reference standard. We evaluated the tests as replacement tests for diagnostic surgery and as triage tests to inform decisions to undertake surgery for endometriosis. Search methods We did not restrict the searches to particular study designs, language or publication dates. To identify trials, we searched the following databases: CENTRAL (2015, July), MEDLINE (inception to May 2015), EMBASE (inception to May 2015), CINAHL (inception to April 2015), PsycINFO (inception to April 2015), Web of Science (inception to April 2015), LILACS (inception to April 2015), OAIster (inception to April 2015), TRIP (inception to April 2015) and ClinicalTrials.gov (inception to April 2015). We searched DARE and PubMed databases up to April 2015 to identify reviews and guidelines as sources of references to potentially relevant studies. We also performed searches for papers recently published and not yet indexed in the major databases. The search strategies incorporated words in the title, abstract, text words across the record and the medical subject headings (MeSH). Selection criteria We considered published peer‐reviewed, randomised controlled or cross‐sectional studies of any size that included prospectively collected samples from any population of reproductive‐aged women suspected of having one or more of the following target conditions: ovarian, peritoneal or deep infiltrating endometriosis (DIE). Data collection and analysis Two authors independently extracted data from each study and performed a quality assessment. For each endometrial diagnostic test, we classified the data as positive or negative for the surgical detection of endometriosis and calculated the estimates of sensitivity and specificity. We considered two or more tests evaluated in the same cohort as separate data sets. We used the bivariate model to obtain pooled estimates of sensitivity and specificity whenever sufficient data were available. The predetermined criteria for a clinically useful test to replace diagnostic surgery was one with a sensitivity of 94% and a specificity of 79%. The criteria for triage tests were set at sensitivity at or above 95% and specificity at or above 50%, which in case of negative results rules out the diagnosis (SnOUT test) or sensitivity at or above 50% with specificity at or above 95%, which in case of positive result rules in the diagnosis (SpIN test). Main results We included 54 studies involving 2729 participants, most of which were of poor methodological quality. The studies evaluated endometrial biomarkers either in specific phases of the menstrual cycle or outside of it, and the studies tested the biomarkers either in menstrual fluid, in whole endometrial tissue or in separate endometrial components. Twenty‐seven studies evaluated the diagnostic performance of 22 endometrial biomarkers for endometriosis. These were angiogenesis and growth factors (PROK‐1), cell‐adhesion molecules (integrins α3β1, α4β1, β1 and α6), DNA‐repair molecules (hTERT), endometrial and mitochondrial proteome, hormonal markers (CYP19, 17βHSD2, ER‐α, ER‐β), inflammatory markers (IL‐1R2), myogenic markers (caldesmon, CALD‐1), neural markers (PGP 9.5, VIP, CGRP, SP, NPY, NF) and tumour markers (CA‐125). Most of these biomarkers were assessed in single studies, whilst only data for PGP 9.5 and CYP19 were available for meta‐analysis. These two biomarkers demonstrated significant diversity for the diagnostic estimates between the studies; however, the data were too limited to reliably determine the sources of heterogeneity. The mean sensitivities and specificities of PGP 9.5 (7 studies, 361 women) were 0.96 (95% confidence interval (CI) 0.91 to 1.00) and 0.86 (95% CI 0.70 to 1.00), after excluding one outlier study, and for CYP19 (8 studies, 444 women), they were were 0.77 (95% CI 0.70 to 0.85) and 0.74 (95% CI 0.65 to 84), respectively. We could not statistically evaluate other biomarkers in a meaningful way. An additional 31 studies evaluated 77 biomarkers that showed no evidence of differences in expression levels between the groups of women with and without endometriosis. Authors' conclusions We could not statistically evaluate most of the biomarkers assessed in this review in a meaningful way. In view of the low quality of most of the included studies, the findings of this review should be interpreted with caution. Although PGP 9.5 met the criteria for a replacement test, it demonstrated considerable inter study heterogeneity in diagnostic estimates, the source of which could not be determined. Several endometrial biomarkers, such as endometrial proteome, 17βHSD2, IL‐1R2, caldesmon and other neural markers (VIP, CGRP, SP, NPY and combination of VIP, PGP 9.5 and SP) showed promising evidence of diagnostic accuracy, but there was insufficient or poor quality evidence for any clinical recommendations. Laparoscopy remains the gold standard for the diagnosis of endometriosis, and using any non‐invasive tests should only be undertaken in a research setting. We have also identified a number of biomarkers that demonstrated no diagnostic value for endometriosis. We recommend that researchers direct future studies towards biomarkers with high diagnostic potential in good quality diagnostic studies. Plain language summary Endometrial biomarkers for the non‐invasive diagnosis of endometriosis Review question Can physicians use biomarkers (distinctive molecules, genes or other characteristics that appear in certain conditions) to reduce the need to surgically diagnose endometriosis? Background The endometrium refers to the tissue that lines the womb and is shed during menstruation. Women with endometriosis have endometrial tissue growing outside the womb, within the pelvic cavity. This tissue responds to reproductive hormones causing painful periods, chronic lower abdominal pain and difficulty conceiving. Currently the only reliable way of diagnosing endometriosis is to perform keyhole surgery and visualise the endometriotic deposits inside the abdomen. Because surgery is risky and expensive, various tests within the endometrium that can be obtained during an in‐office womb sampling procedure have been assessed for their ability to detect endometriosis non‐invasively or with minimal invasion. An accurate test could lead to the diagnosis of endometriosis without the need for surgery, or it could reduce the need for diagnostic surgery so only women who were most likely to have endometriosis would require it. Review teams have also evaluated other non‐invasive ways of diagnosing endometriosis using blood, urine and imaging tests as well as a combination of several testing methods in separate Cochrane reviews within this series. Study characteristics The evidence in this review is current to April 2015. We included 54 studies involving 2729 participants. All studies evaluated reproductive‐aged women who were undertaking diagnostic surgery to investigate symptoms of endometriosis or for other indications. Twenty‐six studies evaluated the role of 22 different biomarkers in diagnosing endometriosis, and 31 studies identified 77 additional biomarkers that had no value in differentiating between women with and without the disease. Key results and quality of evidence Only two of the assessed biomarkers, a neural fibre marker PGP 9.5 and hormonal marker CYP19, were assessed in sufficient number of studies to obtain meaningful results. PGP 9.5 identified endometriosis with enough accuracy to replace surgical diagnosis. Several additional biomarkers (endometrial proteome, 17βHSD2, IL‐1R2, caldesmon and other neural markers) show promise in detecting endometriosis, but there are too few studies to be sure of their diagnostic value. The studies differed in how they were conducted, which groups of women were studied and how the surgery was undertaken. The reports were of low methodological quality, which is why readers cannot consider these results to be reliable unless confirmed in large, high quality studies. Overall, there is not enough evidence to recommend any endometrial test for use in clinical practice for the diagnosis of endometriosis. Future research Further high quality research is necessary to accurately evaluate the diagnostic potential of the endometrial biomarkers for the diagnosis of endometriosis.

UR - http://dx.doi.org/10.1002/14651858.CD012165

ER -

Record #26 of 32

Provider: John Wiley & Sons, Ltd

Content: text/plain; charset="UTF-8"

TY - JOUR

AN - CD009191

AU - Sasongko, TH

AU - Nagalla, S

TI - Angiotensin‐converting enzyme (ACE) inhibitors for proteinuria and microalbuminuria in people with sickle cell disease

JF - Cochrane Database of Systematic Reviews

PY - 2021

IS - 12

PB - John Wiley & Sons, Ltd

SN - 1465-1858

KW - *Albuminuria [drug therapy, etiology]

KW - *Anemia, Sickle Cell [complications, drug therapy]

KW - *Angiotensin-Converting Enzyme Inhibitors [therapeutic use]

KW - *Proteinuria [drug therapy, etiology]

KW - Angiotensins

KW - Humans

KW - Randomized Controlled Trials as Topic

N1 - [Cystic Fibrosis and Genetic Disorders]

DO - 10.1002/14651858.CD009191.pub4

AB - Abstract - Background Sickle cell disease is a group of disorders characterized by deformation of erythrocytes. Renal damage is a frequent complication in sickle cell disease as a result of long‐standing anemia and disturbed circulation through the renal medullary capillaries. Due to the improvement in life expectancy of people with sickle cell disease, there has been a corresponding significant increase in the incidence of renal complications. Microalbuminuria and proteinuria are noted to be a strong predictor of subsequent renal failure. There is extensive experience and evidence with angiotensin‐converting enzyme (ACE) inhibitors over many years in a variety of clinical situations for patients who do not have sickle cell disease, but their effect in people with this disease is unknown. It is common practice to administer ACE inhibitors for sickle nephropathy due to their renoprotective properties; however, little is known about their effectiveness and safety in this setting. This is an update of a Cochrane Review first published in 2013 and 2015. Objectives To determine the effectiveness of ACE inhibitor administration in people with sickle cell disease for decreasing intraglomerular pressure, microalbuminuria and proteinuria and to to assess the safety of ACE inhibitors as pertains to their adverse effects. Search methods The authors searched the Cochrane Cystic Fibrosis and Genetic Disorders Group's Hameoglobinopathies Trials Register comprising references identified from comprehensive electronic database searches and handsearches of relevant journals and abstract books of conference proceedings. Date of the most recent search: 18 October 2021. We also searched clinical trial registries. Date of the most recent search: 22 August 2021. Selection criteria Randomized or quasi‐randomized controlled trials of ACE inhibitors designed to reduce microalbuminuria and proteinuria in people with sickle cell disease compared to either placebo or standard treatment regimen. Data collection and analysis Three authors independently applied the inclusion criteria in order to select studies for inclusion in the review. Two authors assessed the risk of bias of studies and extracted data and the third author verified these assessments. Main results Seven studies were identified through the searches. Six studies were excluded. The included study randomized 22 participants (7 males and 15 females) having proteinuria or microalbuminuria with sickle cell disease and treated the participants for six months (median length of follow up of three months) with captopril or placebo. Overall, the certainty of the evidence provided in this review was very low, since most risk of bias domains were judged to have either an unclear or a high risk of bias. Because of this, we are uncertain whether captopril makes any difference, in total urinary albumin excretion (at six months) as compared to the placebo group, although it yielded a mean difference of ‐49.00 (95% confidence interval (CI) ‐124.10 to 26.10) or in the absolute change score, although it yielded a mean difference of ‐63.00 (95% CI ‐93.78 to ‐32.22). At six months albumin excretion in the captopril group was noted to decrease from baseline by a mean (standard deviation) of 45 (23) mg/day and the placebo group was noted to increase by 18 (45) mg/day. Serum creatinine and potassium levels were reported constant throughout the study (very low‐certainty evidence). The potential for inducing hypotension should be highlighted; the study reported a decrease of 8 mmHg in systolic pressure and 5 mmHg in diastolic and mean blood pressure (very low‐certainty evidence). Authors' conclusions Overall, we judged the certainty of the evidence to be very low. The included study selectively reported its results, was not powered to detect a group difference, should it exist, and otherwise did not offer enough information to allow us to judge the bias inherent in the study. Indirectness (in relation to the limited age and type of population included) and imprecision (wide confidence intervals around the effect estimate) were observed. More long‐term studies involving multiple centers and larger cohorts using a randomized‐controlled design are warranted, especially among the pediatric age group. Detailed reporting of each outcome measure is necessary to allow a clear cut interpretation in a systematic review. One of the difficulties encountered in this review was the lack of detailed data reported in the included study. Overall, we judged the certainty of this evidence to be very low. Plain language summary Drugs that aim to prevent the loss of protein or albumin through urine in people with sickle cell disease Review question We reviewed the evidence on the effect of drugs that aim to prevent people with sickle cell disease losing protein or albumin (a protein made in the liver) in their urine. Background Sickle cell disease is a group of inherited conditions that often lead to kidney damage. High protein or albumin levels in urine is a strong predictor of future kidney failure. Angiotensin‐converting enzyme (ACE) inhibitors are often given to reduce the level of protein or albumin in urine and to protect the kidneys from damage. However, we do not know very much about how effective and safe these are in people with sickle cell disease. Search date The evidence is current to: 22 August 2021. Study characteristics We only included and analysed one study (with 22 adults with sickle cell disease) in the review. The participants had high levels of protein (proteinuria) or albumin (microalbuminuria) in their urine and were randomly selected to be treated for six months with either captopril (an angiotensin‐converting enzyme inhibitor) or placebo (dummy drug with no active medication). Key results The results from this small and very low‐quality study were not convincing. We downgraded our judgements on the certainty of the evidence because of at least an unclear or a high risk of bias in almost all areas we assessed, as well as imprecision (results showed a wide range of possible effects) and indirectness (the study did not include any children and only a small number of adults with normal blood pressure and microalbuminuria). This study did not show that ACE inhibitors could reduce the level of protein or albumin in the urine. The levels of creatinine (a chemical compound left over from energy‐producing processes in muscles; a high level can indicate kidney failure) and potassium in the blood were reported as constant throughout the study. No serious adverse events were noted, although the potential for causing low blood pressure should be highlighted. More long‐term studies involving multiple centers and larger numbers of participants are needed. Certainty of the evidence Overall certainty of the evidence was very low, since we thought there was either a high or unclear risk of bias from all aspects that may contribute to biasing the results (e.g. randomisation, the way treatment allocations were concealed, whether all participants recruited were analysed, whether all planned outcomes were reported, as well as blinding of participants, research personnel and outcome assessment). We also noted that the study did not include children, which limits the evidence for this treatment in children. Our statistical calculations on the effect found a high degree of imprecision. There may be selective reporting on sodium levels, but other electrolyte levels were reported. Nevertheless, the amount of data with detailed descriptions has allowed only limited analysis in this review.

UR - http://dx.doi.org/10.1002/14651858.CD009191.pub4

ER -

Record #27 of 32

Provider: John Wiley & Sons, Ltd

Content: text/plain; charset="UTF-8"

TY - JOUR

AN - CD012204

AU - Hemmingsen, B

AU - Sonne, DP

AU - Metzendorf, MI

AU - Richter, B

TI - Dipeptidyl‐peptidase (DPP)‐4 inhibitors and glucagon‐like peptide (GLP)‐1 analogues for prevention or delay of type 2 diabetes mellitus and its associated complications in people at increased risk for the development of type 2 diabetes mellitus

JF - Cochrane Database of Systematic Reviews

PY - 2017

IS - 5

PB - John Wiley & Sons, Ltd

SN - 1465-1858

KW - Adamantane [analogs & derivatives, therapeutic use]

KW - Blood Glucose [metabolism]

KW - Diabetes Mellitus, Type 2 [complications, *prevention & control]

KW - Dipeptidyl‐Peptidase IV Inhibitors [*therapeutic use]

KW - Exenatide

KW - Fasting

KW - Glucagon‐Like Peptide 1 [*analogs & derivatives]

KW - Glucose Intolerance

KW - Glycated Hemoglobin A [metabolism]

KW - Humans

KW - Hypoglycemic Agents [therapeutic use]

KW - Incretins [*therapeutic use]

KW - Liraglutide [therapeutic use]

KW - Metformin [therapeutic use]

KW - Nitriles [therapeutic use]

KW - Peptides [therapeutic use]

KW - Pyrrolidines [therapeutic use]

KW - Randomized Controlled Trials as Topic

KW - Risk Factors

KW - Venoms [therapeutic use]

KW - Vildagliptin

N1 - [Metabolic and Endocrine Disorders]

DO - 10.1002/14651858.CD012204.pub2

AB - Abstract - Background The projected rise in the incidence of type 2 diabetes mellitus (T2DM) could develop into a substantial health problem worldwide. Whether dipeptidyl‐peptidase (DPP)‐4 inhibitors or glucagon‐like peptide (GLP)‐1 analogues are able to prevent or delay T2DM and its associated complications in people at risk for the development of T2DM is unknown. Objectives To assess the effects of DPP‐4 inhibitors and GLP‐1 analogues on the prevention or delay of T2DM and its associated complications in people with impaired glucose tolerance, impaired fasting blood glucose, moderately elevated glycosylated haemoglobin A1c (HbA1c) or any combination of these. Search methods We searched the Cochrane Central Register of Controlled Trials; MEDLINE; PubMed; Embase; ClinicalTrials.gov; the World Health Organization (WHO) International Clinical Trials Registry Platform; and the reference lists of systematic reviews, articles and health technology assessment reports. We asked investigators of the included trials for information about additional trials. The date of the last search of all databases was January 2017. Selection criteria We included randomised controlled trials (RCTs) with a duration of 12 weeks or more comparing DPP‐4 inhibitors and GLP‐1 analogues with any pharmacological glucose‐lowering intervention, behaviour‐changing intervention, placebo or no intervention in people with impaired fasting glucose, impaired glucose tolerance, moderately elevated HbA1c or combinations of these. Data collection and analysis Two review authors read all abstracts and full‐text articles and records, assessed quality and extracted outcome data independently. One review author extracted data which were checked by a second review author. We resolved discrepancies by consensus or the involvement of a third review author. For meta‐analyses, we planned to use a random‐effects model with investigation of risk ratios (RRs) for dichotomous outcomes and mean differences (MDs) for continuous outcomes, using 95% confidence intervals (CIs) for effect estimates. We assessed the overall quality of the evidence using the GRADE instrument. Main results We included seven completed RCTs; about 98 participants were randomised to a DPP‐4 inhibitor as monotherapy and 1620 participants were randomised to a GLP‐1 analogue as monotherapy. Two trials investigated a DPP‐4 inhibitor and five trials investigated a GLP‐1 analogue. A total of 924 participants with data on allocation to control groups were randomised to a comparator group; 889 participants were randomised to placebo and 33 participants to metformin monotherapy. One RCT of liraglutide contributed 85% of all participants. The duration of the intervention varied from 12 weeks to 160 weeks. We judged none of the included trials at low risk of bias for all 'Risk of bias' domains and did not perform meta‐analyses because there were not enough trials. One trial comparing the DPP‐4 inhibitor vildagliptin with placebo reported no deaths (very low‐quality evidence). The incidence of T2DM by means of WHO diagnostic criteria in this trial was 3/90 participants randomised to vildagliptin versus 1/89 participants randomised to placebo (very low‐quality evidence). Also, 1/90 participants on vildagliptin versus 2/89 participants on placebo experienced a serious adverse event (very low‐quality evidence). One out of 90 participants experienced congestive heart failure in the vildagliptin group versus none in the placebo group (very low‐quality evidence). There were no data on non‐fatal myocardial infarction, stroke, health‐related quality of life or socioeconomic effects reported. All‐cause and cardiovascular mortality following treatment with GLP‐1 analogues were rarely reported; one trial of exenatide reported that no participant died. Another trial of liraglutide 3.0 mg showed that 2/1501 in the liraglutide group versus 2/747 in the placebo group died after 160 weeks of treatment (very low‐quality evidence). The incidence of T2DM following treatment with liraglutide 3.0 mg compared to placebo after 160 weeks was 26/1472 (1.8%) participants randomised to liraglutide versus 46/738 (6.2%) participants randomised to placebo (very low‐quality evidence). The trial established the risk for (diagnosis of) T2DM as HbA1c 5.7% to 6.4% (6.5% or greater), fasting plasma glucose 5.6 mmol/L or greater to 6.9 mmol/L or less (7.0 mmol/L or greater) or two‐hour post‐load plasma glucose 7.8 mmol/L or greater to 11.0 mmol/L (11.1 mmol/L). Altogether, 70/1472 (66%) participants regressed from intermediate hyperglycaemia to normoglycaemia compared with 268/738 (36%) participants in the placebo group. The incidence of T2DM after the 12‐week off‐treatment extension period (i.e. after 172 weeks) showed that five additional participants were diagnosed T2DM in the liraglutide group, compared with one participant in the placebo group. After 12‐week treatment cessation, 740/1472 (50%) participants in the liraglutide group compared with 263/738 (36%) participants in the placebo group had normoglycaemia. One trial used exenatide and 2/17 participants randomised to exenatide versus 1/16 participants randomised to placebo developed T2DM (very low‐quality evidence). This trial did not provide a definition of T2DM. One trial reported serious adverse events in 230/1524 (15.1%) participants in the liraglutide 3.0 mg arm versus 96/755 (12.7%) participants in the placebo arm (very low quality evidence). There were no serious adverse events in the trial using exenatide. Non‐fatal myocardial infarction was reported in 1/1524 participants in the liraglutide arm and in 0/55 participants in the placebo arm at 172 weeks (very low‐quality evidence). One trial reported congestive heart failure in 1/1524 participants in the liraglutide arm and in 1/755 participants in the placebo arm (very low‐quality evidence). Participants receiving liraglutide compared with placebo had a small mean improvement in the physical component of the 36‐item Short Form scale showing a difference of 0.87 points (95% CI 0.17 to 1.58; P = 0.02; 1 trial; 1791 participants; very low‐quality evidence). No trial evaluating GLP‐1‐analogues reported data on stroke, microvascular complications or socioeconomic effects. Authors' conclusions There is no firm evidence that DPP‐4 inhibitors or GLP‐1 analogues compared mainly with placebo substantially influence the risk of T2DM and especially its associated complications in people at increased risk for the development of T2DM. Most trials did not investigate patient‐important outcomes. Plain language summary Dipeptidyl‐peptidase (DPP)‐4 inhibitors and glucagon‐like peptide (GLP)‐1 analogues for prevention or delay of type 2 diabetes mellitus Review question Are the glucose‐lowering medicines, DPP‐4 inhibitors (e.g. linagliptin or vildagliptin) and GLP‐1 analogues (e.g. exenatide or liraglutide) able to prevent or delay the development of type 2 diabetes and its associated complications in people at risk for the development of type 2 diabetes? Background DPP‐4 inhibitors and GLP‐1 analogues are widely used to treat people with type 2 diabetes. People with moderately elevated blood glucose are said to be at an increased risk for developing type 2 diabetes (often referred to as 'prediabetes'). It is currently not known whether DPP‐4 inhibitors or GLP‐1 analogues should be prescribed for people with raised blood glucose levels who do not have type 2 diabetes. We wanted to find out whether these medicines could prevent or delay type 2 diabetes in people at increased risk. We also wanted to know the effects on patient‐important outcomes such as complications of diabetes (e.g. kidney and eye disease, heart attacks, strokes), death from any cause, health‐related quality of life (a measure of a person's satisfaction with their life and health) and side effects of the medicines. Study characteristics Participants had to have blood glucose levels higher than considered normal, but below the glucose levels that are used to diagnose type 2 diabetes mellitus. We found seven randomised controlled trials (clinical studies where people are randomly put into one of two or more treatment groups) with 2702 participants. The duration of the treatments varied from 12 weeks to 160 weeks. One study investigating liraglutide dominated the evidence (2285/2702 participants). The participants in this study were overweight or obese. This evidence is up to date as of January 2017. Key results DPP‐4 inhibitors did not reduce the risk of developing type 2 diabetes compared with placebo (a dummy medicine). In the big study investigating the GLP‐1‐analogue liraglutide, given in a dose used for obese people (3.0 mg), the development of type 2 diabetes was delayed: 26/1472 (1.8%) participants randomised to liraglutide compared with 46/738 (6.2%) participants randomised to placebo developed type 2 diabetes after 160 weeks. On the other side, 970/1472 (66%) participants randomised to liraglutide compared with 268/738 (36%) participants randomised to placebo switched back to normal glucose levels. This study was extended for another 12 weeks without treatment and five additional participants developed diabetes in the liraglutide group, compared with one participant in the placebo group. After the 12 weeks without treatment, 740/1472 (50%) participants in the liraglutide group compared with 263/738 (36%) participants in the placebo group had glucose levels considered as normal. This means that to keep chances high to prevent type 2 diabetes in people at risk one probably needs to continuously take this drug. Of note, serious adverse events (e.g. defined as hospitalisation or a hazard putting the participant at risk, such as an interaction with another medicine) happened more often following liraglutide treatment (230/1524 (15%) participants in the liraglutide group and 96/755 (13%) participants in the placebo group) and it is unclear whether taking this drug is safe in the long term. We detected neither an advantage nor a disadvantage of DPP‐4 inhibitors or GLP‐1 analogues in relation to non‐fatal heart attacks, non‐fatal strokes or heart failure. Our included studies did not report on other complications of diabetes such as kidney or eye disease. The effects on health‐related quality of life were inconclusive. In the included studies, very few participants died and there was no apparent relation to treatment. Future studies should investigate more patient‐important outcomes like complications of diabetes and especially the side effects of the medications, because we do not know for sure whether 'prediabetes' is just a condition arbitrarily defined by a laboratory measurement, is in fact a real risk factor for type 2 diabetes mellitus and whether treatment of this condition translates into better patient‐important outcomes. Quality of the evidence All included trials had deficiencies in the way they were conducted or how key items were reported. For the individual comparisons, the number of participants was small, resulting in a high risk of random errors (play of chance).

UR - http://dx.doi.org/10.1002/14651858.CD012204.pub2

ER -

Record #28 of 32

Provider: John Wiley & Sons, Ltd

Content: text/plain; charset="UTF-8"

TY - JOUR

AN - CD008650

AU - Jurgens, TM

AU - Whelan, AM

AU - Killian, L

AU - Doucette, S

AU - Kirk, S

AU - Foy, E

TI - Green tea for weight loss and weight maintenance in overweight or obese adults

JF - Cochrane Database of Systematic Reviews

PY - 2012

IS - 12

PB - John Wiley & Sons, Ltd

SN - 1465-1858

KW - *Tea

KW - *Weight Loss

KW - Adult

KW - Body Weight

KW - Camellia sinensis [chemistry]

KW - Humans

KW - Japan

KW - Obesity [*drug therapy]

KW - Overweight [*drug therapy]

KW - Phytotherapy [*methods]

KW - Randomized Controlled Trials as Topic

N1 - [Metabolic and Endocrine Disorders]

DO - 10.1002/14651858.CD008650.pub2

AB - Abstract - Background Preparations of green tea are used as aids in weight loss and weight maintenance. Catechins and caffeine, both contained in green tea, are each believed to have a role in increasing energy metabolism, which may lead to weight loss. A number of randomised controlled trials (RCTs) evaluating the role of green tea in weight loss have been published; however, the efficacy of green tea preparations in weight loss remains unclear. Objectives To assess the efficacy and safety of green tea preparations for weight loss and weight maintenance in overweight or obese adults. Search methods We searched the following databases from inception to specified date as well as reference lists of relevant articles: The Cochrane Library (Issue 12, 2011), MEDLINE (December 2011), EMBASE (December 2011), CINAHL (January 2012), AMED (January 2012), Biological Abstracts (January 2012), IBIDS (August 2010), Obesity+ (January 2012), IPA (January 2012) and Web of Science (December 2011). Current Controlled Trials with links to other databases of ongoing trials was also searched. Selection criteria RCTs of at least 12 weeks' duration comparing green tea preparations to a control in overweight or obese adults. Data collection and analysis Three authors independently extracted data, assessed studies for risk of bias and quality, with differences resolved by consensus. Heterogeneity of included studies was assessed visually using forest plots and quantified using the I 2 statistic. We synthesised data using meta‐analysis and descriptive analysis as appropriate; subgroup and sensitivity analyses were conducted. Adverse effects reported in studies were recorded. Main results Due to the level of heterogeneity among studies, studies were divided into two groups; those conducted in Japan and those conducted outside Japan. Study length ranged between 12 and 13 weeks. Meta‐analysis of six studies conducted outside Japan showed a mean difference (MD) in weight loss of ‐0.04 kg (95% CI ‐0.5 to 0.4; P = 0.88; I 2 = 18%; 532 participants). The eight studies conducted in Japan were not similar enough to allow pooling of results and MD in weight loss ranged from ‐0.2 kg to ‐3.5 kg (1030 participants) in favour of green tea preparations. Meta‐analysis of studies measuring change in body mass index (BMI) conducted outside Japan showed a MD in BMI of ‐0.2 kg/m 2 (95% CI ‐0.5 to 0.1; P = 0.21; I 2 = 38%; 222 participants). Differences among the eight studies conducted in Japan did not allow pooling of results and showed a reduction in BMI ranging from no effect to ‐1.3 kg/m 2 (1030 participants), in favour of green tea preparations over control. Meta‐analysis of five studies conducted outside Japan and measuring waist circumference reported a MD of ‐0.2 cm (95% CI ‐1.4 to 0.9; P = 0.70; I 2 = 58%; 404 participants). Differences among the eight studies conducted in Japan did not allow pooling of results and showed effects on waist circumference ranging from a gain of 1 cm to a loss of 3.3 cm (1030 participants). Meta‐analysis for three weight loss studies, conducted outside Japan, with waist‐to‐hip ratio data (144 participants) yielded no significant change (MD 0; 95% CI ‐0.02 to 0.01). Analysis of two studies conducted to determine if green tea could help to maintain weight after a period of weight loss (184 participants) showed a change in weight loss of 0.6 to ‐1.6 kg, a change in BMI from 0.2 to ‐0.5 kg/m 2 and a change in waist circumference from 0.3 to ‐1.7 cm. In the eight studies that recorded adverse events, four reported adverse events that were mild to moderate, with the exception of two (green tea preparations group) that required hospitalisation (reported as not associated with the intervention). Nine studies reported on compliance/adherence, one study assessed attitude towards eating as part of the health‐related quality of life outcome. No studies reported on patient satisfaction, morbidity or cost. Authors' conclusions Green tea preparations appear to induce a small, statistically non‐significant weight loss in overweight or obese adults. Because the amount of weight loss is small, it is not likely to be clinically important. Green tea had no significant effect on the maintenance of weight loss. Of those studies recording information on adverse events, only two identified an adverse event requiring hospitalisation. The remaining adverse events were judged to be mild to moderate. Plain language summary Green tea for weight loss and weight maintenance in overweight or obese adults Green tea has a long history of many uses, one of which is helping overweight people to lose weight and to maintain weight loss. Believed to be able to increase a person's energy output, green tea weight loss preparations are extracts of green tea that contain a higher concentration of ingredients (catechins and caffeine) than the typical green tea beverage prepared from a tea bag and boiling water. This review looked at 15 weight loss studies and three studies measuring weight maintenance where some form of a green tea preparation was given to one group and results compared to a group receiving a control. Neither group knew whether they were receiving the green tea preparation or the control. A total of 1945 participants completed the studies, ranging in length from 12 to 13 weeks. In summary, the loss in weight in adults who had taken a green tea preparation was statistically not significant, was very small and is not likely to be clinically important. Similar results were found in studies that used other ways to measure loss in weight (body mass index, waist circumference). Studies examining the effect of green tea preparations on weight maintenance did not show any benefit compared to the use of a control preparation. Most adverse effects, such as nausea, constipation, abdominal discomfort and increased blood pressure, were judged to be mild to moderate and to be unrelated to the green tea or control intervention. No deaths were reported, although adverse events required hospitalisation. One study attempted to look at health‐related quality of life by asking participants about their attitudes towards eating. Nine studies tracked participants' compliance with green tea preparations. Studies did not include any information about the effects of green tea preparations on morbidity, costs or patient satisfaction.

UR - http://dx.doi.org/10.1002/14651858.CD008650.pub2

ER -

Record #29 of 32

Provider: John Wiley & Sons, Ltd

Content: text/plain; charset="UTF-8"

TY - JOUR

AN - CD012368

AU - Madsen, KS

AU - Kähler, P

AU - Kähler, LKA

AU - Madsbad, S

AU - Gnesin, F

AU - Metzendorf, MI

AU - Richter, B

AU - Hemmingsen, B

TI - Metformin and second‐ or third‐generation sulphonylurea combination therapy for adults with type 2 diabetes mellitus

JF - Cochrane Database of Systematic Reviews

PY - 2019

IS - 4

PB - John Wiley & Sons, Ltd

SN - 1465-1858

KW - Diabetes Mellitus, Type 2 [*drug therapy]

KW - Drug Therapy, Combination

KW - Humans

KW - Hypoglycemia [chemically induced]

KW - Hypoglycemic Agents [*therapeutic use]

KW - Metformin [*therapeutic use]

KW - Sulfonylurea Compounds [*therapeutic use]

KW - Treatment Outcome

N1 - [Metabolic and Endocrine Disorders]

DO - 10.1002/14651858.CD012368.pub2

AB - Abstract - Background The number of people with type 2 diabetes mellitus (T2DM) is increasing worldwide. The combination of metformin and sulphonylurea (M+S) is a widely used treatment. Whether M+S shows better or worse effects in comparison with other antidiabetic medications for people with T2DM is still controversial. Objectives To assess the effects of metformin and sulphonylurea (second‐ or third‐generation) combination therapy for adults with type 2 diabetes mellitus. Search methods We updated the search of a recent systematic review from the Agency for Healthcare Research and Quality (AHRQ). The updated search included CENTRAL, MEDLINE, Embase, ClinicalTrials.gov and WHO ICTRP. The date of the last search was March 2018. We searched manufacturers' websites and reference lists of included trials, systematic reviews, meta‐analyses and health technology assessment reports. We asked investigators of the included trials for information about additional trials. Selection criteria We included randomised controlled trials (RCTs) randomising participants 18 years old or more with T2DM to M+S compared with metformin plus another glucose‐lowering intervention or metformin monotherapy with a treatment duration of 52 weeks or more. Data collection and analysis Two review authors read all abstracts and full‐text articles and records, assessed risk of bias and extracted outcome data independently. We used a random‐effects model to perform meta‐analysis, and calculated risk ratios (RRs) for dichotomous outcomes and mean differences (MDs) for continuous outcomes, using 95% confidence intervals (CIs) for effect estimates. We assessed the certainty of the evidence using the GRADE instrument. Main results We included 32 RCTs randomising 28,746 people. Treatment duration ranged between one to four years. We judged none of these trials as low risk of bias for all 'Risk of bias' domains. Most important events per person were all‐cause and cardiovascular mortality, serious adverse events (SAE), non‐fatal stroke (NFS), non‐fatal myocardial infarction (MI) and microvascular complications. Most important comparisons were as follows: Five trials compared M+S (N = 1194) with metformin plus a glucagon‐like peptide 1 analogue (N = 1675): all‐cause mortality was 11/1057 (1%) versus 11/1537 (0.7%), risk ratio (RR) 1.15 (95% confidence interval (CI) 0.49 to 2.67); 3 trials; 2594 participants; low‐certainty evidence; cardiovascular mortality 1/307 (0.3%) versus 1/302 (0.3%), low‐certainty evidence; serious adverse events (SAE) 128/1057 (12.1%) versus 194/1537 (12.6%), RR 0.90 (95% CI 0.73 to 1.11); 3 trials; 2594 participants; very low‐certainty evidence; non‐fatal myocardial infarction (MI) 2/549 (0.4%) versus 6/1026 (0.6%), RR 0.57 (95% CI 0.12 to 2.82); 2 trials; 1575 participants; very low‐certainty evidence. Nine trials compared M+S (N = 5414) with metformin plus a dipeptidyl‐peptidase 4 inhibitor (N = 6346): all‐cause mortality was 33/5387 (0.6%) versus 26/6307 (0.4%), RR 1.32 (95% CI 0.76 to 2.28); 9 trials; 11,694 participants; low‐certainty evidence; cardiovascular mortality 11/2989 (0.4%) versus 9/3885 (0.2%), RR 1.54 (95% CI 0.63 to 3.79); 6 trials; 6874 participants; low‐certainty evidence; SAE 735/5387 (13.6%) versus 779/6307 (12.4%), RR 1.07 (95% CI 0.97 to 1.18); 9 trials; 11,694 participants; very low‐certainty evidence; NFS 14/2098 (0.7%) versus 8/2995 (0.3%), RR 2.21 (95% CI 0.74 to 6.58); 4 trials; 5093 participants; very low‐certainty evidence; non‐fatal MI 15/2989 (0.5%) versus 13/3885 (0.3%), RR 1.45 (95% CI 0.69 to 3.07); 6 trials; 6874 participants; very low‐certainty evidence; one trial in 64 participants reported no microvascular complications were observed (very low‐certainty evidence). Eleven trials compared M+S (N = 3626) with metformin plus a thiazolidinedione (N = 3685): all‐cause mortality was 123/3300 (3.7%) versus 114/3354 (3.4%), RR 1.09 (95% CI 0.85 to 1.40); 6 trials; 6654 participants; low‐certainty evidence; cardiovascular mortality 37/2946 (1.3%) versus 41/2994 (1.4%), RR 0.78 (95% CI 0.36 to 1.67); 4 trials; 5940 participants; low‐certainty evidence; SAE 666/3300 (20.2%) versus 671/3354 (20%), RR 1.01 (95% CI 0.93 to 1.11); 6 trials; 6654 participants; very low‐certainty evidence; NFS 20/1540 (1.3%) versus 16/1583 (1%), RR 1.29 (95% CI 0.67 to 2.47); P = 0.45; 2 trials; 3123 participants; very low‐certainty evidence; non‐fatal MI 25/1841 (1.4%) versus 21/1877 (1.1%), RR 1.21 (95% CI 0.68 to 2.14); P = 0.51; 3 trials; 3718 participants; very low‐certainty evidence; three trials (3123 participants) reported no microvascular complications (very low‐certainty evidence). Three trials compared M+S (N = 462) with metformin plus a glinide (N = 476): one person died in each intervention group (3 trials; 874 participants; low‐certainty evidence); no cardiovascular mortality (2 trials; 446 participants; low‐certainty evidence); SAE 34/424 (8%) versus 27/450 (6%), RR 1.68 (95% CI 0.54 to 5.21); P = 0.37; 3 trials; 874 participants; low‐certainty evidence; no NFS (1 trial; 233 participants; very low‐certainty evidence); non‐fatal MI 2/215 (0.9%) participants in the M+S group; 2 trials; 446 participants; low‐certainty evidence; no microvascular complications (1 trial; 233 participants; low‐certainty evidence). Four trials compared M+S (N = 2109) with metformin plus a sodium‐glucose co‐transporter 2 inhibitor (N = 3032): all‐cause mortality was 13/2107 (0.6%) versus 19/3027 (0.6%), RR 0.96 (95% CI 0.44 to 2.09); 4 trials; 5134 participants; very low‐certainty evidence; cardiovascular mortality 4/1327 (0.3%) versus 6/2262 (0.3%), RR 1.22 (95% CI 0.33 to 4.41); 3 trials; 3589 participants; very low‐certainty evidence; SAE 315/2107 (15.5%) versus 375/3027 (12.4%), RR 1.02 (95% CI 0.76 to 1.37); 4 trials; 5134 participants; very low‐certainty evidence; NFS 3/919 (0.3%) versus 7/1856 (0.4%), RR 0.87 (95% CI 0.22 to 3.34); 2 trials; 2775 participants; very low‐certainty evidence; non‐fatal MI 7/890 (0.8%) versus 8/1374 (0.6%), RR 1.43 (95% CI 0.49 to 4.18; 2 trials); 2264 participants; very low‐certainty evidence; amputation of lower extremity 1/437 (0.2%) versus 1/888 (0.1%); very low‐certainty evidence. Trials reported more hypoglycaemic episodes with M+S combination compared to all other metformin‐antidiabetic agent combinations. Results for M+S versus metformin monotherapy were inconclusive. There were no RCTs comparing M+S with metformin plus insulin. We identified nine ongoing trials and two trials are awaiting assessment. Together these trials will include approximately 16,631 participants. Authors' conclusions There is inconclusive evidence whether M+S combination therapy compared with metformin plus another glucose‐lowering intervention results in benefit or harm for most patient‐important outcomes (mortality, SAEs, macrovascular and microvascular complications) with the exception of hypoglycaemia (more harm for M+S combination). No RCT reported on health‐related quality of life. Plain language summary Metformin and sulphonylurea combination therapy for adults with type 2 diabetes mellitus Review question We wanted to investigate the effects of the combination of the antidiabetic medications metformin plus sulphonylurea compared with other antidiabetic interventions in people with type 2 diabetes. Background Many people with type 2 diabetes are treated with several types of glucose‐lowering drugs such as 'sulphonylureas' (for example glibenclamide or glyburide, glipizide and gliclazide). These medications lower blood glucose by stimulating the secretion of insulin in the body, thereby increasing insulin levels in the blood. Another antidiabetic agent, metformin lowers blood glucose by improving the body's ability to make insulin work better (insulin sensitivity). The combination of metformin plus sulphonylurea is widely used. We wanted to investigate the effects of metformin plus sulphonylurea on patient‐important outcomes such as complications of diabetes (for example kidney and eye disease, heart attacks, strokes), death from any cause, health‐related quality of life and side effects of the medications. Study characteristics We found 32 randomised controlled studies (clinical trials where people are randomly put into one of two or more treatment groups), which allocated 28,746 people with type 2 diabetes to either metformin plus sulphonylurea or a comparator group. The comparator groups consisted of the following types of antidiabetic medications in addition to metformin: five studies with glucagon‐like peptide 1 analogues, nine studies with dipeptidyl‐peptidase 4 inhibitors, 11 studies with thiazolidinediones, three studies with glinides and four studies with sodium‐glucose co‐transporter 2 inhibitors. Participants of the studies were treated for between one and four years. There were big differences between people taking part in the studies, especially with regard to age, how long people had diabetes and whether diabetes complications were present at the start of the study. This evidence is up to date as of March 2018. Key results Data on patient‐important outcomes were few, and data were sparse for all comparisons of metformin plus sulphonylurea with other antidiabetic medications. The available data did not show firm differences between metformin plus sulphonylurea and other combinations of metformin with antidiabetic drugs or metformin only for most patient‐important outcomes. There were more events with low blood sugar (hypoglycaemic episodes) with metformin plus sulphonylurea combination treatment compared to all other combinations of metformin with another antidiabetic compound. We did not identify studies reporting on health‐related quality of life. We identified nine ongoing studies and two yet unpublished studies are awaiting assessment. Together these studies will include around 16,631 participants. Once results are published these studies could significantly influence the findings of our review. Certainty of the evidence All included studies had deficiencies in the way they were conducted or how study authors reported the results. For individual comparisons of the antidiabetic medications the number of participants was often small, resulting in a high risk of random error (play of chance).

UR - http://dx.doi.org/10.1002/14651858.CD012368.pub2

ER -

Record #30 of 32

Provider: John Wiley & Sons, Ltd

Content: text/plain; charset="UTF-8"

TY - JOUR

AN - CD003641

AU - Colquitt, JL

AU - Pickett, K

AU - Loveman, E

AU - Frampton, GK

TI - Surgery for weight loss in adults

JF - Cochrane Database of Systematic Reviews

PY - 2014

IS - 8

PB - John Wiley & Sons, Ltd

SN - 1465-1858

KW - Adult

KW - Female

KW - Gastric Bypass [*methods]

KW - Gastroplasty [*methods]

KW - Humans

KW - Ligation [methods]

KW - Male

KW - Obesity, Morbid [*surgery]

KW - Randomized Controlled Trials as Topic

KW - Weight Loss

N1 - [Metabolic and Endocrine Disorders]

DO - 10.1002/14651858.CD003641.pub4

AB - Abstract - Background Bariatric (weight loss) surgery for obesity is considered when other treatments have failed. The effects of the available bariatric procedures compared with medical management and with each other are uncertain. This is an update of a Cochrane review first published in 2003 and most recently updated in 2009. Objectives To assess the effects of bariatric surgery for overweight and obesity, including the control of comorbidities. Search methods Studies were obtained from searches of numerous databases, supplemented with searches of reference lists and consultation with experts in obesity research. Date of last search was November 2013. Selection criteria Randomised controlled trials (RCTs) comparing surgical interventions with non‐surgical management of obesity or overweight or comparing different surgical procedures. Data collection and analysis Data were extracted by one review author and checked by a second review author. Two review authors independently assessed risk of bias and evaluated overall study quality utilising the GRADE instrument. Main results Twenty‐two trials with 1798 participants were included; sample sizes ranged from 15 to 250. Most studies followed participants for 12, 24 or 36 months; the longest follow‐up was 10 years. The risk of bias across all domains of most trials was uncertain; just one was judged to have adequate allocation concealment. All seven RCTs comparing surgery with non‐surgical interventions found benefits of surgery on measures of weight change at one to two years follow‐up. Improvements for some aspects of health‐related quality of life (QoL) (two RCTs) and diabetes (five RCTs) were also found. The overall quality of the evidence was moderate. Five studies reported data on mortality, no deaths occurred. Serious adverse events (SAEs) were reported in four studies and ranged from 0% to 37% in the surgery groups and 0% to 25% in the no surgery groups. Between 2% and 13% of participants required reoperations in the five studies that reported these data. Three RCTs found that laparoscopic Roux‐en‐Y gastric bypass (L)(RYGB) achieved significantly greater weight loss and body mass index (BMI) reduction up to five years after surgery compared with laparoscopic adjustable gastric banding (LAGB). Mean end‐of‐study BMI was lower following LRYGB compared with LAGB: mean difference (MD) ‐5.2 kg/m² (95% confidence interval (CI) ‐6.4 to ‐4.0; P < 0.00001; 265 participants; 3 trials; moderate quality evidence). Evidence for QoL and comorbidities was very low quality. The LRGYB procedure resulted in greater duration of hospitalisation in two RCTs (4/3.1 versus 2/1.5 days) and a greater number of late major complications (26.1% versus 11.6%) in one RCT. In one RCT the LAGB required high rates of reoperation for band removal (9 patients, 40.9%). Open RYGB, LRYGB and laparoscopic sleeve gastrectomy (LSG) led to losses of weight and/or BMI but there was no consistent picture as to which procedure was better or worse in the seven included trials. MD was ‐0.2 kg/m² (95% CI ‐1.8 to 1.3); 353 participants; 6 trials; low quality evidence) in favour of LRYGB. No statistically significant differences in QoL were found (one RCT). Six RCTs reported mortality; one death occurred following LRYGB. SAEs were reported by one RCT and were higher in the LRYGB group (4.5%) than the LSG group (0.9%). Reoperations ranged from 6.7% to 24% in the LRYGB group and 3.3% to 34% in the LSG group. Effects on comorbidities, complications and additional surgical procedures were neutral, except gastro‐oesophageal reflux disease improved following LRYGB (one RCT). One RCT of people with a BMI 25 to 35 and type 2 diabetes found laparoscopic mini‐gastric bypass resulted in greater weight loss and improvement of diabetes compared with LSG, and had similar levels of complications. Two RCTs found that biliopancreatic diversion with duodenal switch (BDDS) resulted in greater weight loss than RYGB in morbidly obese patients. End‐of‐study mean BMI loss was greater following BDDS: MD ‐7.3 kg/m² (95% CI ‐9.3 to ‐5.4); P < 0.00001; 107 participants; 2 trials; moderate quality evidence). QoL was similar on most domains. In one study between 82% to 100% of participants with diabetes had a HbA1c of less than 5% three years after surgery. Reoperations were higher in the BDDS group (16.1% to 27.6%) than the LRYGB group (4.3% to 8.3%). One death occurred in the BDDS group. One RCT comparing laparoscopic duodenojejunal bypass with sleeve gastrectomy versus LRYGB found BMI, excess weight loss, and rates of remission of diabetes and hypertension were similar at 12 months follow‐up (very low quality evidence). QoL, SAEs and reoperation rates were not reported. No deaths occurred in either group. One RCT comparing laparoscopic isolated sleeve gastrectomy (LISG) versus LAGB found greater improvement in weight‐loss outcomes following LISG at three years follow‐up (very low quality evidence). QoL, mortality and SAEs were not reported. Reoperations occurred in 20% of the LAGB group and in 10% of the LISG group. One RCT (unpublished) comparing laparoscopic gastric imbrication with LSG found no statistically significant difference in weight loss between groups (very low quality evidence). QoL and comorbidities were not reported. No deaths occurred. Two participants in the gastric imbrication group required reoperation. Authors' conclusions Surgery results in greater improvement in weight loss outcomes and weight associated comorbidities compared with non‐surgical interventions, regardless of the type of procedures used. When compared with each other, certain procedures resulted in greater weight loss and improvements in comorbidities than others. Outcomes were similar between RYGB and sleeve gastrectomy, and both of these procedures had better outcomes than adjustable gastric banding. For people with very high BMI, biliopancreatic diversion with duodenal switch resulted in greater weight loss than RYGB. Duodenojejunal bypass with sleeve gastrectomy and laparoscopic RYGB had similar outcomes, however this is based on one small trial. Isolated sleeve gastrectomy led to better weight‐loss outcomes than adjustable gastric banding after three years follow‐up. This was based on one trial only. Weight‐related outcomes were similar between laparoscopic gastric imbrication and laparoscopic sleeve gastrectomy in one trial. Across all studies adverse event rates and reoperation rates were generally poorly reported. Most trials followed participants for only one or two years, therefore the long‐term effects of surgery remain unclear. Plain language summary Surgery for obesity Review question What are the effects of weight loss (bariatric) surgery for overweight or obese adults? Background Obesity is associated with many health problems and a higher risk of death. Bariatric surgery for obesity is usually only considered when other treatments have failed. We aimed to compare surgical interventions with non‐surgical interventions for obesity (such as drugs, diet and exercise) and to compare different surgical procedures. Bariatric surgery can be considered for people with a body mass index (BMI = kg/m²) greater than 40, or for those with a BMI less than 40 and obesity‐related diseases such as diabetes. Study characteristics We included 22 studies comparing surgery with non‐surgical interventions, or comparing different types of surgery. Altogether 1496 participants were allocated to surgery and 302 participants to non‐surgical interventions. Most studies followed participants for 12 to 36 months, the longest follow‐up was 10 years. The majority of participants were women and, on average, in their early 30s to early 50s. Key results Seven studies compared surgery with non‐surgical interventions. Due to differences in the way that the studies were designed we decided not to generate an average of their results. The direction of the effect indicated that people who had surgery achieved greater weight loss one to two years afterwards compared with people who did not have surgery. Improvements in quality of life and diabetes were also found. No deaths occurred, reoperations in the surgical intervention groups ranged between 2% and 13%, as reported in five studies. Three studies found that gastric bypass (GB) achieved greater weight loss up to five years after surgery compared with adjustable gastric band (AGB): the BMI at the end of the studies was on average five units less. The GB procedure resulted in greater duration of hospitalisation and a greater number of late major complications. AGB required high rates of reoperation for removal of the gastric band. Seven studies compared GB with sleeve gastrectomy (SG). Overall there were no important differences for weight loss, quality of life, comorbidities and complications, although gastro‐oesophageal reflux disease improved in more patients following GB in one study. One death occurred in the GB group. Serious adverse events occurred in 5% of the GB group and 1% of SG group, as reported in one study. Two studies reported 7% to 24% of people with GB and 3% to 34% of those with SG requiring reoperations. Two studies found that biliopancreatic diversion with duodenal switch resulted in greater weight loss than GB after two or four years in people with a relatively high BMI. BMI at the end of the studies was on average seven units lower. One death occurred in the biliopancreatic diversion group. Reoperations were higher in the biliopancreatic diversion group (16% to 28%) than the GB group (4% to 8%). One study comparing duodenojejunal bypass with SG versus GB found weight loss outcomes and rates of remission of diabetes and hypertension were similar at 12 months follow‐up. No deaths occurred in either group, reoperation rates were not reported. One study found that BMI was reduced by 10 units more following SG at three years follow‐up compared with AGB. Reoperations occurred in 20% of the AGB group and in 10% of the SG group. One study found no relevant difference in weight‐loss outcomes following gastric imbrication compared with SG. No deaths occurred; 17% of participants in the gastric imbrication group required reoperation. Quality of the evidence From the information that was available to us about the studies, we were unable to assess how well designed they were. Adverse events and reoperation rates were not consistently reported in the publications of the studies. Most studies followed participants for only one or two years, therefore the long‐term effects of surgery remain unclear. Few studies assessed the effects of bariatric surgery in treating comorbidities in participants with a lower BMI. There is therefore a lack of evidence for the use of bariatric surgery in treating comorbidities in people who are overweight or who do not meet standard criteria for bariatric surgery. Currentness of data This evidence is up to date as of November 2013.

UR - http://dx.doi.org/10.1002/14651858.CD003641.pub4

ER -

Record #31 of 32

Provider: John Wiley & Sons, Ltd

Content: text/plain; charset="UTF-8"

TY - JOUR

AN - CD006739

AU - Richter, B

AU - Bandeira‐Echtler, E

AU - Bergerhoff, K

AU - Lerch, C

TI - Dipeptidyl peptidase‐4 (DPP‐4) inhibitors for type 2 diabetes mellitus

JF - Cochrane Database of Systematic Reviews

PY - 2008

IS - 2

PB - John Wiley & Sons, Ltd

SN - 1465-1858

KW - Adamantane [*analogs & derivatives, therapeutic use]

KW - Diabetes Mellitus, Type 2 [*drug therapy]

KW - Dipeptidyl‐Peptidase IV Inhibitors [*therapeutic use]

KW - Humans

KW - Nitriles [*therapeutic use]

KW - Pyrazines [*therapeutic use]

KW - Pyrrolidines [*therapeutic use]

KW - Randomized Controlled Trials as Topic

KW - Sitagliptin Phosphate

KW - Triazoles [*therapeutic use]

KW - Vildagliptin

N1 - [Metabolic and Endocrine Disorders]

DO - 10.1002/14651858.CD006739.pub2

AB - Abstract - Background In type 2 diabetes mellitus there is a progressive loss of beta‐cell function. One new approach yielding promising results is the use of the orally active dipeptidyl peptidase‐4 (DPP‐4) inhibitors like sitagliptin and vildagliptin. Objectives To assess the effects of dipeptidyl peptidase‐4 (DPP‐4) inhibitors for type 2 diabetes mellitus. Search methods Studies were obtained from computerised searches of MEDLINE, EMBASE and The Cochrane Library . Selection criteria Studies were included if they were randomised controlled trials in adult people with type 2 diabetes mellitus and had a trial duration of at least 12 weeks. Data collection and analysis Two authors independently assessed risk of bias and extracted data. Pooling of studies was performed by means of fixed‐effect meta‐analysis. Main results Twenty‐five studies of good quality were identified, 11 trials evaluated sitagliptin and 14 trials vildagliptin treatment. Altogether, 6743 patients were randomised in sitagliptin and 6121 patients in vildagliptin studies, respectively. Sitagliptin and vildagliptin studies ranged from 12 to 52 weeks duration. No data were published on mortality, diabetic complications, costs of treatment and health‐related quality of life.   Sitagliptin and vildagliptin therapy in comparison with placebo resulted in an HbA1c reduction of approximately 0.7% and 0.6%, respectively. Data on comparisons with active comparators were limited but indicated no improved metabolic control following DPP‐4 intervention in contrast to other hypoglycaemic agents. Sitagliptin and vildagliptin therapy did not result in weight gain but weight loss was more pronounced following placebo interventions. No definite conclusions could be drawn from published data on sitagliptin and vildagliptin effects on measurements of beta‐cell function. Overall, sitagliptin and vildagliptin were well tolerated, no severe hypoglycaemia was reported in patients taking sitagliptin or vildagliptin. All‐cause infections increased significantly after sitagliptin treatment but did not reach statistical significance following vildagliptin therapy. All published randomised controlled trials of at least 12 weeks treatment with sitagliptin and vildagliptin only reported routine laboratory safety measurements Authors' conclusions DPP‐4 inhibitors have some theoretical advantages over existing therapies with oral antidiabetic compounds but should currently be restricted to individual patients. Long‐term data especially on cardiovascular outcomes and safety are urgently needed before widespread use of these new agents. More information on the benefit‐risk ratio of DPP‐4 inhibitor treatment is necessary especially analysing adverse effects on parameters of immune function. Also, long‐term data are needed investigating patient‐oriented parameters like health‐related quality of life, diabetic complications and all‐cause mortality. Plain language summary Dipeptidyl peptidase‐4 (DPP‐4) inhibitors for type 2 diabetes mellitus Dipeptidyl peptidase‐4 (DPP‐4) inhibitors like sitagliptin and vildagliptin are promising new medicines for the treatment of type 2 diabetes mellitus. They are supposed to improve metabolic control (as measured by lowering blood glucose) without causing severe hypoglycaemia (low blood sugar levels leading to unconsciousness and other symptoms).   Altogether 12.864 people took part in 25 studies investigating the new compounds sitagliptin and vildagliptin. Most studies lasted 24 weeks, the longest trials evaluated 52 weeks of treatment. So far, no study reported on patient‐oriented parameters like mortality, diabetic complications, costs of treatment and health‐related quality of life. When compared to placebo treatment sitagliptin and vildagliptin improved metabolic control. Comparison with other already established blood‐glucose lowering drugs did not reveal advantages of DPP‐4 treatment. Weight gain was not observed after sitagliptin and vildagliptin therapy. Overall, sitagliptin and vildagliptin were well tolerated, no severe hypoglycaemia was reported in patients taking sitagliptin or vildagliptin. However, all‐cause infections increased significantly after sitagliptin treatment but did not reach statistical significance following vildagliptin therapy. Unfortunately, all published randomised controlled trials of at least 12 weeks treatment with sitagliptin and vildagliptin only reported routine laboratory safety measurements. Since the new DPP‐4 inhibitors may influence immune function additional long‐term data on the safety of these drugs are necessary. Also, cardiovascular outcomes like heart attacks and strokes should not be increased with any antidiabetic therapy but data so far are lacking. Until new information arrives, DPP‐4 inhibitors should only be used under controlled conditions and in individual patients.

UR - http://dx.doi.org/10.1002/14651858.CD006739.pub2

ER -

Record #32 of 32

Provider: John Wiley & Sons, Ltd

Content: text/plain; charset="UTF-8"

TY - JOUR

AN - CD006060

AU - Richter, B

AU - Bandeira‐Echtler, E

AU - Bergerhoff, K

AU - Clar, C

AU - Ebrahim, SH

TI - Pioglitazone for type 2 diabetes mellitus

JF - Cochrane Database of Systematic Reviews

PY - 2006

IS - 4

PB - John Wiley & Sons, Ltd

SN - 1465-1858

KW - Diabetes Mellitus, Type 2 [*drug therapy]

KW - Humans

KW - Hypoglycemic Agents [*therapeutic use]

KW - Pioglitazone

KW - Randomized Controlled Trials as Topic

KW - Thiazolidinediones [*therapeutic use]

N1 - [Metabolic and Endocrine Disorders]

DO - 10.1002/14651858.CD006060.pub2

AB - Abstract - Background Diabetes has long been recognised as a strong, independent risk factor for cardiovascular disease, a problem which accounts for approximately 70% of all mortality in people with diabetes. Prospective studies show that compared to their non‐diabetic counterparts, the relative risk of cardiovascular mortality for men with diabetes is two to three and for women with diabetes is three to four. The two biggest trials in type 2 diabetes, the United Kingdom Prospective Diabetes Study (UKPDS) and the University Group Diabetes Program (UGDP) study did not reveal a reduction of cardiovascular endpoints through improved metabolic control. Theoretical benefits of the newer peroxisome proliferator activated receptor gamma (PPAR‐gamma) activators like pioglitazone on endothelial function and cardiovascular risk factors might result in fewer macrovascular disease events in people with type 2 diabetes mellitus. Objectives To assess the effects of pioglitazone in the treatment of type 2 diabetes. Search methods Studies were obtained from computerised searches of MEDLINE, EMBASE and The Cochrane Library . Selection criteria Studies were included if they were randomised controlled trials in adult people with type 2 diabetes mellitus and had a trial duration of at least 24 weeks. Data collection and analysis Two authors independently assessed trial quality and extracted data. Pooling of studies by means of random‐effects meta‐analysis could be performed for adverse events only. Main results Twenty‐two trials which randomised approximately 6200 people to pioglitazone treatment were identified. Longest duration of therapy was 34.5 months. Published studies of at least 24 weeks pioglitazone treatment in people with type 2 diabetes mellitus did not provide convincing evidence that patient‐oriented outcomes like mortality, morbidity, adverse effects, costs and health‐related quality of life are positively influenced by this compound. Metabolic control measured by glycosylated haemoglobin A1c (HbA1c) as a surrogate endpoint did not demonstrate clinically relevant differences to other oral antidiabetic drugs. Occurrence of oedema was significantly raised. The results of the single trial with relevant clinical endpoints (Prospective Pioglitazone Clinical Trial In Macrovascular Events ‐ PROactive study) have to be regarded as hypothesis‐generating and need confirmation. Authors' conclusions Until new evidence becomes available, the benefit‐risk ratio of pioglitazone remains unclear. Different therapeutic indications for pioglitazone of the two big U.S. and European drug agencies should be clarified to reduce uncertainties amongst patients and physicians. Plain language summary Pioglitazone for type 2 diabetes mellitus Diseases of the heart and blood vessels account for approximately 70% of all mortality in people with diabetes. Compared to their non‐diabetic counterparts the relative risk of mortality caused by disorders of the heart and blood vessels is two to three for men and three to four for women with diabetes. Type 2 diabetes is mainly characterised by a reduced ability of the hormone insulin to stimulate glucose uptake in body fat and muscles (insulin resistance) and affects most people suffering from diabetes. Several medications are on the market to treat diabetes, amongst them pioglitazone as a member of the 'glitazones' reduced risk factors for diseases of the heart and blood vessels. Since the two biggest trials in people with type 2 diabetes showed that improved blood glucose alone is not enough to reduce the risk of the above mentioned diseases we looked for longer‐term studies investigating 24 weeks as a minimum of pioglitazone treatment on patient‐oriented outcomes. As patient‐oriented outcomes we defined mortality, complications of diabetes, side effects of the medication, health‐related quality of life, costs and metabolic control (lowering of blood glucose to near normal levels).   Twenty‐two trials randomised approximately 6200 people to pioglitazone treatment. The longest duration of pioglitazone therapy was 34.5 months. Unfortunately, the published studies of at least 24 weeks pioglitazone treatment in people with type 2 diabetes mellitus did not provide convincing evidence that patient‐oriented outcomes are positively influenced by this compound. The occurrence of oedema was significantly raised. The results of the single trial with relevant endpoints (Prospective Pioglitazone Clinical Trial In Macrovascular Events ‐ PROactive study) have to be confirmed by other independent investigations. Until new evidence becomes available (several large trials are ongoing) the place of pioglitazone in the treatment of type 2 diabetes mellitus remains unclear.   Furthermore, confusion arises due to different labelling of pioglitazone, for example in Europe and the USA. Consumers and physicians need clear guidance and transparent information about which studies exactly are used for the decisions of the relevant drug authorities.

UR - http://dx.doi.org/10.1002/14651858.CD006060.pub2

ER -

**Supplimentary 2. Search results of Embase database**

.TY - JOUR

M3 - Article

Y1 - 2022

VL - 6

IS - 1

SN - 2397-768X

JF - npj Precision Oncology

JO - npj Precis. Oncol.

UR - https://www.embase.com/search/results?subaction=viewrecord&id=L2014865664&from=export

U2 - L2014865664

DB - Embase

U3 - 2022-02-10

L2 - http://dx.doi.org/10.1038/s41698-021-00248-2

DO - 10.1038/s41698-021-00248-2

A1 - Leitner, B.P.

A1 - Givechian, K.B.

A1 - Ospanova, S.

A1 - Beisenbayeva, A.

A1 - Politi, K.

A1 - Perry, R.J.

M1 - (Leitner B.P., brooks.leitner@yale.edu; Perry R.J., rachel.perry@yale.edu) Department of Cellular & Molecular Physiology, Yale School of Medicine, New Haven, CT, United States

M1 - (Leitner B.P., brooks.leitner@yale.edu; Perry R.J., rachel.perry@yale.edu) Department of Internal Medicine (Endocrinology), Yale School of Medicine, New Haven, CT, United States

M1 - (Givechian K.B.) Department of Genetics, Yale School of Medicine, New Haven, CT, United States

M1 - (Ospanova S.) Nazarbayev Intellectual School of Physics and Mathematics, Almaty, Kazakhstan

M1 - (Beisenbayeva A.) Kazakhstan International School, Almaty, Kazakhstan

M1 - (Politi K.) Department of Pathology, Yale School of Medicine, New Haven, CT, United States

M1 - (Politi K.) Department of Internal Medicine (Oncology), Yale School of Medicine, New Haven, CT, United States

M1 - (Politi K.) Yale Cancer Center, Yale School of Medicine, New Haven, CT, United States

AD - B.P. Leitner, Department of Cellular & Molecular Physiology, Yale School of Medicine, New Haven, CT, United States

AD - R.J. Perry, Department of Cellular & Molecular Physiology, Yale School of Medicine, New Haven, CT, United States

T1 - Multimodal analysis suggests differential immuno-metabolic crosstalk in lung squamous cell carcinoma and adenocarcinoma

LA - English

KW - adjuvant chemotherapy

KW - adult

KW - amino acid metabolism

KW - article

KW - body mass

KW - cancer prognosis

KW - controlled study

KW - fatty acid metabolism

KW - gene expression

KW - genetic transcription

KW - glucose metabolism

KW - glucose transport

KW - histology

KW - histopathology

KW - human

KW - human tissue

KW - intra-abdominal fat

KW - intraperitoneal fat

KW - lung adenocarcinoma

KW - metabolic capacity

KW - personalized medicine

KW - positron emission tomography-computed tomography

KW - protein expression

KW - single cell RNA seq

KW - squamous cell lung carcinoma

KW - T lymphocyte

KW - cystathionine gamma lyase

KW - endogenous compound

KW - fatty acid

KW - fluorodeoxyglucose f 18

KW - glucose

KW - glutaminase

KW - glutamine

KW - ribosome protein

N2 - Immunometabolism within the tumor microenvironment is an appealing target for precision therapy approaches in lung cancer. Interestingly, obesity confers an improved response to immune checkpoint inhibition in non-small cell lung cancer (NSCLC), suggesting intriguing relationships between systemic metabolism and the immunometabolic environment in lung tumors. We hypothesized that visceral fat and 18F-Fluorodeoxyglucose uptake influenced the tumor immunometabolic environment and that these bidirectional relationships differ in NSCLC subtypes, lung adenocarcinoma (LUAD) and lung squamous cell carcinoma (LUSC). By integrating 18F-FDG PET/CT imaging, bulk and single-cell RNA-sequencing, and histology, we observed that LUSC had a greater dependence on glucose than LUAD. In LUAD tumors with high glucose uptake, glutaminase was downregulated, suggesting a tradeoff between glucose and glutamine metabolism, while in LUSC tumors with high glucose uptake, genes related to fatty acid and amino acid metabolism were also increased. We found that tumor-infiltrating T cells had the highest expression of glutaminase, ribosomal protein 37, and cystathionine gamma-lyase in NSCLC, highlighting the metabolic flexibility of this cell type. Further, we demonstrate that visceral adiposity, but not body mass index (BMI), was positively associated with tumor glucose uptake in LUAD and that patients with high BMI had favorable prognostic transcriptional profiles, while tumors of patients with high visceral fat had poor prognostic gene expression. We posit that metabolic adjunct therapy may be more successful in LUSC rather than LUAD due to LUAD’s metabolic flexibility and that visceral adiposity, not BMI alone, should be considered when developing precision medicine approaches for the treatment of NSCLC.

ER -

TY - JOUR

M3 - Article

Y1 - 2022

VL - 60

IS - 2

SP - 234

EP - 240

SN - 2212-5353

SN - 2212-5345

JF - Respiratory Investigation

JO - Respir. Invest.

UR - https://www.embase.com/search/results?subaction=viewrecord&id=L2016153055&from=export

U2 - L2016153055

C5 - 34972681

DB - Embase

DB - Medline

U3 - 2022-01-04

L2 - http://dx.doi.org/10.1016/j.resinv.2021.11.003

DO - 10.1016/j.resinv.2021.11.003

A1 - Tateishi, A.

A1 - Horinouchi, H.

A1 - Yoshida, T.

A1 - Masuda, K.

A1 - Jo, H.

A1 - Shinno, Y.

A1 - Okuma, Y.

A1 - Goto, Y.

A1 - Yamamoto, N.

A1 - Ohe, Y.

M1 - (Tateishi A.; Horinouchi H., hhorinou@ncc.go.jp; Yoshida T.; Masuda K.; Jo H.; Shinno Y.; Okuma Y.; Goto Y.; Yamamoto N.; Ohe Y.) Department of Thoracic Oncology, National Cancer Center Hospital, Tokyo, Japan

M1 - (Tateishi A.) Cancer Medicine, Cooperative Graduate School, Jikei University Graduate School of Medicine,

M1 - (Yoshida T.; Yamamoto N.) Department of Experimental Therapeutics, National Cancer Center Hospital, Tokyo, Japan

AD - H. Horinouchi, National Cancer Center Hospital, 5-1-1 Tsukiji, Chuo-ku, Tokyo, Japan

T1 - Correlation between body mass index and efficacy of anti-PD-1 inhibitor in patients with non-small cell lung cancer

LA - English

KW - adult

KW - advanced cancer

KW - article

KW - body mass

KW - cancer center

KW - cancer inhibition

KW - cancer patient

KW - cancer survival

KW - cohort analysis

KW - controlled study

KW - drug combination

KW - drug efficacy

KW - drug therapy

KW - female

KW - gene expression

KW - human

KW - major clinical study

KW - male

KW - non small cell lung cancer

KW - obesity

KW - overall response rate

KW - overall survival

KW - progression free survival

KW - protein expression

KW - retrospective study

KW - endogenous compound

KW - immune checkpoint inhibitor

KW - nivolumab

KW - pembrolizumab

KW - programmed death 1 ligand 1

KW - programmed death 1 receptor

N2 - Background: High body mass index (BMI) has been reported to be associated with the efficacy of immune checkpoint inhibitors in patients with advanced non-small cell lung cancer (NSCLC), but the association between BMI and efficacy of anti-PD-1 inhibitors remains controversial. The present study investigated this association in patients with advanced NSCLC. Methods: We retrospectively reviewed patients with advanced NSCLC who received PD-1 inhibitors at the National Cancer Center Hospital between January 2016 and December 2018. The efficacy of PD-1 inhibitors (progression-free survival [PFS], overall survival [OS], and response rate) was compared between overweight (BMI ≥25 kg/m2) and non-overweight (BMI <25 kg/m2) groups. Cohort 1 included patients with high PD-L1 expression who were treated with pembrolizumab as first-line therapy; Cohort 2 included patients treated with nivolumab/pembrolizumab as second- or later-line treatment. Results: A total of 324 patients were included in this study and the median BMI (IQR) was 21.4 (19.5–23.6) kg/m2. Of the 324 patients, 279 (86.1%) and 45 (13.9%) were in the non-overweight and overweight groups, respectively. No significant differences in objective response rate (ORR), PFS, or OS were found between overweight and non-overweight patients overall (n = 324; overweight vs. non-overweight: ORR, 28.9% vs. 31.9%, respectively [p = 0.68]; PFS, 7.6 vs. 5.8 months, respectively [p = 0.43]; and OS, 17.6 vs. 15.3 months, respectively [p = 0.90]), or between overweight and non-overweight patients in Cohorts 1 and 2. Conclusions: No significant differences in the efficacy of PD-1 inhibitors were observed between overweight and non-overweight patients.

ER -

TY - JOUR

M3 - Article

Y1 - 2022

VL - 10

IS - 2

SN - 2051-1426

JF - Journal for immunotherapy of cancer

JO - J Immunother Cancer

UR - https://www.embase.com/search/results?subaction=viewrecord&id=L637280661&from=export

U2 - L637280661

C5 - 35173031

DB - Medline

U3 - 2022-02-23

L2 - http://dx.doi.org/10.1136/jitc-2021-004374

DO - 10.1136/jitc-2021-004374

A1 - Cortellini, A.

A1 - Ricciuti, B.

A1 - Vaz, V.R.

A1 - Soldato, D.

A1 - Alessi, J.V.

A1 - Dall'Olio, F.G.

A1 - Banna, G.L.

A1 - Muthuramalingam, S.

A1 - Chan, S.

A1 - Majem, M.

A1 - Piedra, A.

A1 - Lamberti, G.

A1 - Andrini, E.

A1 - Addeo, A.

A1 - Friedlaender, A.

A1 - Facchinetti, F.

A1 - Gorría, T.

A1 - Mezquita, L.

A1 - Hoton, D.

A1 - Valerie, L.

A1 - Nana, F.A.

A1 - Artingstall, J.

A1 - Comins, C.

A1 - Di Maio, M.

A1 - Caglio, A.

A1 - Cave, J.

A1 - McKenzie, H.

A1 - Newsom-Davis, T.

A1 - Evans, J.S.

A1 - Tiseo, M.

A1 - D'Alessio, A.

A1 - Fulgenzi, C.A.M.

A1 - Besse, B.

A1 - Awad, M.M.

A1 - Pinato, D.J.

M1 - (Cortellini A., alessiocortellini@gmail.com; Evans J.S.; D'Alessio A.; Fulgenzi C.A.M.; Pinato D.J.) Department of Surgery and Cancer, Imperial College London, London, United Kingdom

M1 - (Ricciuti B.; Vaz V.R.; Alessi J.V.; Awad M.M.) Lowe Center for Thoracic Oncology, Dana Farber Cancer Institute, Boston, MA, United States

M1 - (Soldato D.; Dall'Olio F.G.; Besse B.) Cancer Medicine Department, Gustave Roussy, Villejuif, France

M1 - (Banna G.L.) Candiolo Cancer Institute, FPO-IRCCS, Candiolo, Italy

M1 - (Muthuramalingam S.) Oncology Department, United Lincolnshire Hospital NHS Trust, Lincoln, United Kingdom

M1 - (Chan S.) Oncology Department, Queen Alexandra University Hospital, Portsmouth Hospitals NHS Trust, Portsmouth, United Kingdom

M1 - (Majem M.; Piedra A.) Medical Oncology Department, Hospital de la Santa Creu i Sant Pau, Barcelona, Spain

M1 - (Lamberti G.; Andrini E.) Department of Experimental, Diagnostic and Specialty Medicine, S. Orsola-Malpighi University Hospital, Alma Mater Studiorum University of Bologna, Bologna, Italy

M1 - (Addeo A.; Friedlaender A.) Medical Oncology, University Hospital of Geneva, Geneva, Switzerland

M1 - (Facchinetti F.) Biomarqueurs Prédictifs et Nouvelles Stratégies Thérapeutiques en Oncologie, Institut Gustave Roussy, Inserm, Université Paris-Saclay, Villejuif, France

M1 - (Gorría T.) Department of Medical Oncology, Hospital Clinic, Barcelona, Spain

M1 - (Mezquita L.) Medical Oncology Department, Hospital Clinic de Barcelona, Barcelona, Spain

M1 - (Hoton D.) Department of Pathology, Bruxelles, Cliniques universitaires Saint-Luc, Belgium

M1 - (Valerie L.) Department of Cardiovascular and Thoracic Surgery, IREC, Bruxelles, Cliniques Universitaires Saint-Luc, Belgium

M1 - (Nana F.A.) Institut de Recherche Expérimentale et Clinique (IREC), Pôle de Pneumologie, ORL et Dermatologie (PNEU), Université catholique de Louvain (UCLouvain), Brussels, Belgium

M1 - (Artingstall J.; Comins C.) Bristol Haematology and Oncology Centre, Bristol, United Kingdom

M1 - (Di Maio M.; Caglio A.) Department of Oncology, University of Turin and Mauriziano Hospital, Turin, Italy

M1 - (Cave J.; McKenzie H.) Department of Medical Oncology, University Hospital Southampton NHS Foundation Trust, Southampton, United Kingdom

M1 - (Newsom-Davis T.) Department of Oncology and National Centre for HIV Malignancies, Chelsea and Westminster Hospital, London, United Kingdom

M1 - (Tiseo M.) Department of Medicine and Surgery, University of Parma, Parma, Italy

M1 - (Tiseo M.) Medical Oncology Unit, University Hospital of Parma, Parma, Italy

M1 - (D'Alessio A.) Department of Biomedical Sciences, Humanitas University, Pieve Emanuele (Milan), Italy

M1 - (Fulgenzi C.A.M.) Department of Medical Oncology, University Campus Bio-Medico, Rome, Italy

M1 - (Besse B.) School of Medicine, University Paris-Saclay, Villejuif, France

M1 - (Pinato D.J.) Division of Oncology, Department of Translational Medicine, University of Piemonte Orientale, Novara, Italy

T1 - Prognostic effect of body mass index in patients with advanced NSCLC treated with chemoimmunotherapy combinations

LA - English

KW - adult

KW - advanced cancer

KW - article

KW - body mass

KW - body weight

KW - cancer chemotherapy

KW - cancer combination chemotherapy

KW - cancer patient

KW - cancer prognosis

KW - cancer staging

KW - cancer survival

KW - central nervous system

KW - clinical outcome

KW - cohort analysis

KW - controlled study

KW - ECOG Performance Status

KW - female

KW - human

KW - immunity

KW - liver metastasis

KW - log rank test

KW - major clinical study

KW - male

KW - metabolism

KW - multicenter study

KW - never smoker

KW - non small cell lung cancer

KW - obese patient

KW - obesity

KW - outcome assessment

KW - overall survival

KW - prevalence

KW - progression free survival

KW - underweight

KW - immune checkpoint inhibitor

KW - programmed death 1 receptor

N2 - INTRODUCTION: It has been recognized that increasing body mass index (BMI) is associated with improved outcome from immune checkpoint inhibitors (ICIs) in patients with various malignancies including non-small cell lung cancer (NSCLC). However, it is unclear whether baseline BMI may influence outcomes from first-line chemoimmunotherapy combinations. METHODS: In this international multicenter study, we evaluated the association between baseline BMI, progression-free survival (PFS) and overall survival (OS) in a cohort of patients with stage IV NSCLC consecutively treated with first-line chemoimmunotherapy combinations. BMI was categorized according to WHO criteria. RESULTS: Among the 853 included patients, 5.3% were underweight; 46.4% were of normal weight; 33.8% were overweight; and 14.5% were obese. Overweight and obese patients were more likely aged ≥70 years (p=0.00085), never smokers (p<0.0001), with better baseline Eastern Cooperative Oncology Group-Performance Status (p=0.0127), and had lower prevalence of central nervous system (p=0.0002) and liver metastases (p=0.0395). Univariable analyses showed a significant difference in the median OS across underweight (15.5 months), normal weight (14.6 months), overweight (20.9 months), and obese (16.8 months) patients (log-rank: p=0.045, log rank test for trend: p=0.131), while no difference was found with respect to the median PFS (log-rank for trend: p=0.510). Neither OS nor PFS was significantly associated with baseline BMI on multivariable analysis. CONCLUSIONS: In contrast to what was observed in the context of chemotherapy-free ICI-based regimens, baseline BMI does not affect clinical outcomes from chemoimmunotherapy combinations in patients with advanced NSCLC.

ER -

TY - JOUR

M3 - Article

Y1 - 2021

VL - 13

IS - 23

SN - 2072-6694

JF - Cancers

JO - Cancers

UR - https://www.embase.com/search/results?subaction=viewrecord&id=L2014757788&from=export

U2 - L2014757788

DB - Embase

U3 - 2021-12-10

L2 - http://dx.doi.org/10.3390/cancers13236109

DO - 10.3390/cancers13236109

A1 - Zhang, D.

A1 - Shah, N.J.

A1 - Cook, M.

A1 - Blackburn, M.

A1 - Serzan, M.T.

A1 - Advani, S.

A1 - Potosky, A.L.

A1 - Atkins, M.B.

A1 - Braithwaite, D.

M1 - (Zhang D., dzhang2@ufl.edu; Braithwaite D., dbraithwaite@ufl.edu) Department of Epidemiology, University of Florida College of Public Health and Health Professions, Gainesville, FL, United States

M1 - (Zhang D., dzhang2@ufl.edu; Braithwaite D., dbraithwaite@ufl.edu) University of Florida Health Cancer Center, Gainesville, FL, United States

M1 - (Shah N.J., shahn6@mskcc.org) Department of Medicine, Solid Tumor Genitourinary Oncology Service, Memorial Sloan Kettering Cancer Center, New York, NY, United States

M1 - (Cook M., Michael.R.Cook@gunet.georgetown.edu; Blackburn M., Matthew.J.Blackburn@gunet.georgetown.edu; Serzan M.T., Michael.t.serzan@gunet.georgetown.edu) Department of Medicine, Division of Hematology/Oncology MedStar, Georgetown University Hospital, Washington, DC, United States

M1 - (Advani S., sa1542@georgetown.edu; Atkins M.B., mba41@georgetown.edu) Department of Oncology, Georgetown University School of Medicine, Washington, DC, United States

M1 - (Advani S., sa1542@georgetown.edu) Transplant Education Research Center, Terasaki Institute of Biomedical Innovation, Los Angeles, CA, United States

M1 - (Potosky A.L., arnold.potosky@georgetown.edu; Atkins M.B., mba41@georgetown.edu) Georgetown Lombardi Comprehensive Cancer Center, Washington, DC, United States

M1 - (Braithwaite D., dbraithwaite@ufl.edu) Department of Aging and Geriatric Research, University of Florida, Gainesville, FL, United States

AD - M.B. Atkins, Department of Oncology, Georgetown University School of Medicine, Washington, DC, United States

AD - D. Braithwaite, Department of Epidemiology, University of Florida College of Public Health and Health Professions, Gainesville, FL, United States

T1 - Association between body mass index and immune‐related adverse events (IrAEs) among advanced‐stage cancer patients receiving immune checkpoint inhibitors: A pan‐cancer analysis

LA - English

KW - adult

KW - advanced cancer

KW - adverse drug reaction

KW - article

KW - body mass

KW - body weight

KW - cancer patient

KW - cancer staging

KW - controlled study

KW - drug therapy

KW - female

KW - human

KW - immune-related gene

KW - lung

KW - major clinical study

KW - male

KW - melanoma

KW - middle aged

KW - side effect

KW - immune checkpoint inhibitor

N2 - Evidence regarding the association between body mass index (BMI) and immune‐related adverse events (irAEs) among cancer patients receiving immune checkpoint inhibitors (ICIs) is limited. Here, we use cross‐sectional hospital‐based data to explore their relationship. Pre‐treatment BMI was treated as an ordinal variable (<25, 25 to ≤ 30, ≥30 kg/m2). The outcome of interest was irAEs after ICI initiation. A multivariable logistic regression model estimated the adjusted odds ratio (aOR) and 95% confidence interval (CI) of BMI. A total of 684 patients with stage III or IV cancer were included in the study (lung: 269, melanoma: 204, other: 211). The mean age at the first dose of ICI was 64.1 years (SD = 13.5), 394 patients (57.6%) were male, and over one‐third (N = 260, 38.0%) were non‐White. Overall, 52.9% of patients had BMI ≥ 25 kg/m2 (25 to ≤ 30: 217, ≥30: 145) and 288 (42.1%) had irAEs after ICI treatment. Patients with higher BMI tended to have a higher rate of irAEs (<25: 35.7%, 25 to ≤ 30: 47.0%, ≥30: 49.0%). The multivariable logistic regression yielded consistent results (BMI ≥ 30 vs. BMI < 25: aOR = 1.47, 95% CI = 0.96–2.23; 25 ≤ BMI < 30 vs. BMI < 25: aOR = 1.46, 95% CI = 1.02–2.11, p‐trend = 0.04). In conclusion, among patients with advanced cancer receiving ICIs, the rate of irAEs appears to be higher among those with higher BMI.

ER -

TY - JOUR

M3 - Article

Y1 - 2021

VL - 35

IS - 11

SP - 1253

EP - 1263

SN - 1864-6433

SN - 0914-7187

JF - Annals of Nuclear Medicine

JO - Ann. Nucl. Med.

UR - https://www.embase.com/search/results?subaction=viewrecord&id=L2013398837&from=export

U2 - L2013398837

C5 - 34370219

DB - Embase

DB - Medline

DB -

U3 - 2021-08-16

U4 - 2021-08-16

L2 - http://dx.doi.org/10.1007/s12149-021-01667-8

DO - 10.1007/s12149-021-01667-8

A1 - Ito, K.

A1 - Kitajima, K.

A1 - Toriihara, A.

A1 - Ishibashi, M.

A1 - Nakahara, T.

A1 - Daisaki, H.

A1 - Ohe, Y.

A1 - Honda, R.

A1 - Kijima, T.

A1 - Hasegawa, S.

A1 - Nakajo, M.

M1 - (Ito K., kimito@ncc.go.jp) Department of Diagnostic Radiology, National Cancer Center Hospital, 5-1-1 Tsukiji, Chuo-ku, Tokyo, Japan

M1 - (Kitajima K.) Division of Nuclear Medicine and PET Center, Department of Radiology, Hyogo College of Medicine, 1-1 Mukogawa-cho, Nishinomiya, Hyogo, Japan

M1 - (Toriihara A.) PET Imaging Center, Asahi General Hospital, 1326 I, Asahi, Chiba, Japan

M1 - (Ishibashi M.) Division of Radiology, Department of Pathophysiological and Therapeutic Sciences, Tottori University, 86 Nishi-cho, Yonago, Tottori, Japan

M1 - (Nakahara T.) Department of Diagnostic Radiology, Keio University School of Medicine, 35 Shinanomachi, Shinjuku-ku, Tokyo, Japan

M1 - (Daisaki H.) Graduate School of Radiological Technology, Gunma Prefectural College of Health Science, 323-1 Kamioki machi, Maebashi, Gunma, Japan

M1 - (Ohe Y.) Department of Thoracic Oncology, National Cancer Center Hospital, 5-1-1 Tsukiji, Chuo-ku, Tokyo, Japan

M1 - (Honda R.) Department of Respiratory Medicine, Asahi General Hospital, 1326 I, Asahi, Chiba, Japan

M1 - (Kijima T.) Department of Respiratory Medicine and Hematology, Department of Internal Medicine, Hyogo College of Medicine, 1-1 Mukogawa-cho, Nishinomiya, Hyogo, Japan

M1 - (Hasegawa S.) Department of Thoracic Surgery, Hyogo College of Medicine, 1-1 Mukogawa-cho, Nishinomiya, Hyogo, Japan

M1 - (Nakajo M.) Department of Radiology, Graduate School of Medical and Dental Sciences, Kagoshima University, 8-35-1, Sakuragaoka, Kagoshima, Japan

AD - K. Ito, Department of Diagnostic Radiology, National Cancer Center Hospital, 5-1-1 Tsukiji, Chuo-ku, Tokyo, Japan

T1 - 18F-FDG PET/CT for monitoring anti-PD-1 therapy in patients with non-small cell lung cancer using SUV harmonization of results obtained with various types of PET/CT scanners used at different centers

LA - English

KW - Aquiduo

KW - GXL16

KW - mCT64

KW - MI

KW - PET-CT scanner

KW - TF64

KW - antineoplastic metal complex

KW - atezolizumab

KW - durvalumab

KW - fluorodeoxyglucose f 18

KW - immune checkpoint inhibitor

KW - nivolumab

KW - pembrolizumab

KW - adult

KW - aged

KW - article

KW - cancer chemotherapy

KW - cancer immunotherapy

KW - cancer prognosis

KW - colitis

KW - controlled study

KW - disease exacerbation

KW - female

KW - human

KW - hypophysitis

KW - interstitial pneumonia

KW - lean body weight

KW - major clinical study

KW - male

KW - maximum standardized uptake value

KW - metabolic disorder

KW - multiple cycle treatment

KW - myasthenia gravis

KW - non small cell lung cancer

KW - overall survival

KW - pericardial effusion

KW - positron emission tomography-computed tomography

KW - retrospective study

KW - thyroiditis

KW - treatment outcome

KW - treatment response

KW - uveitis

KW - very elderly

KW - Aquiduo

KW - Biograph Duo

KW - Discovery 600

KW - GXL16

KW - Ingenuity TF

KW - mCT64

KW - MI

KW - TF64

C3 - Aquiduo(Toshiba)

C3 - Biograph Duo(Siemens)

C3 - Discovery 600(General Electric)

C3 - GXL16(Philips)

C3 - Ingenuity TF(Philips)

C3 - mCT64(Siemens)

C3 - MI(General Electric)

C3 - TF64(Philips)

C4 - General Electric

C4 - Philips

C4 - Siemens

C4 - Toshiba

N2 - Objective: The prognostic value of treatment response in patients with non-small cell lung cancer (NSCLC) treated with immune-checkpoint inhibitors (ICIs) shown by 18F-fludeoxyglucose (FDG) positron emission tomography/computed tomography (PET/CT) results obtained with multiple types of PET scanners using standardized uptake value (SUV) harmonization was evaluated. Methods: Fifty-eight patients treated with ICIs who underwent 18F-FDG PET/CT examinations with nine types of PET scanners at six hospitals were enrolled. SUV harmonization of multiple PET scanner results was performed using the dedicated software packages “RAVAT” and “RC Tool for Harmonization”. Tumor response was assessed by change in sum of harmonized SUVmax, according to the European Organization for Research and Treatment of Cancer (EORTC5) or the SUV of up to five lesions normalized to lean body mass, according to the PET Response Criteria in Solid Tumors (PERCIST5) and immunotherapy-modified PERCIST (imPERCIST5) criteria. The correlation between tumor response according to those three definitions and overall survival (OS) was evaluated and compared to known prognostic factors. Results: One-year OS in responders and non-responders for harmonized EROTC5 was 86 and 32%, for harmonized PERCIST5 was 86 and 32%, and for harmonized imPERCIST5 was 80 and 30%, respectively (each p = 0.001). Univariate analysis showed that all response criteria remained as prognostic factors. However, there was an overlap for the categories stable metabolic disease (SMD) and progression metabolic disease (PMD) in survival curves using the PET treatment response criteria. Conclusion: In patients with NSCLC treated with ICIs, tumor response based on the harmonized response criteria was associated with OS. PET response criteria using harmonized metabolic parameters may be difficult to routinely employ in daily practice due to overlapping SMD and PMD, although may have a supporting role for determining prognosis.

ER -

TY - JOUR

M3 - Conference Abstract

Y1 - 2021

VL - 111

IS - 3

SP - e604

EP - e605

SN - 1879-355X

SN - 0360-3016

JF - International Journal of Radiation Oncology Biology Physics

JO - Int. J. Radiat. Oncol. Biol. Phys.

UR - https://www.embase.com/search/results?subaction=viewrecord&id=L2014605170&from=export

U2 - L2014605170

DB - Embase

U4 - 2021-09-21

L2 - http://dx.doi.org/10.1016/j.ijrobp.2021.07.1613

DO - 10.1016/j.ijrobp.2021.07.1613

A1 - Chen, X.

A1 - LeCompte, M.C.

A1 - Kleinberg, L.R.

A1 - Hales, R.K.

A1 - Voong, K.R.

A1 - Forde, P.

A1 - Brahmer, J.R.

A1 - Markowski, M.

A1 - Ryan, D.M.

A1 - Lo, L.

A1 - Sciubba, D.M.

A1 - Redmond, K.J.

M1 - (Chen X.; LeCompte M.C.; Kleinberg L.R.; Hales R.K.; Voong K.R.; Redmond K.J.) Department of Radiation Oncology and Molecular Radiation Sciences, Johns Hopkins University School of Medicine, Baltimore, MD, United States

M1 - (Forde P.; Brahmer J.R.; Markowski M.) Department of Oncology, Sidney Kimmel Comprehensive Cancer Center, Johns Hopkins University School of Medicine, Baltimore, MD, United States

M1 - (Ryan D.M.) Department of Radiology and Radiological Science, Johns Hopkins University School of Medicine, Baltimore, MD, United States

M1 - (Lo L.; Sciubba D.M.) Department of Neurosurgery, Johns Hopkins University School of Medicine, Baltimore, MD, United States

AD - X. Chen, Department of Radiation Oncology and Molecular Radiation Sciences, Johns Hopkins University School of Medicine, Baltimore, MD, United States

T1 - Immune Checkpoint Inhibitors Improve Survival and Local Control in Patients With Spine Metastasis After Stereotactic Body Radiotherapy

LA - English

KW - cytotoxic T lymphocyte antigen 4

KW - endogenous compound

KW - immune checkpoint inhibitor

KW - programmed death 1 receptor

KW - adult

KW - body mass

KW - body weight

KW - cancer control

KW - cancer inhibition

KW - cancer patient

KW - cancer radiotherapy

KW - cancer survival

KW - colorectal cancer

KW - conference abstract

KW - consultation

KW - controlled study

KW - drug therapy

KW - female

KW - human

KW - liver cancer

KW - major clinical study

KW - male

KW - median survival time

KW - melanoma

KW - non small cell lung cancer

KW - overall survival

KW - radiation oncology

KW - renal cell carcinoma

KW - retrospective study

KW - spine metastasis

KW - spine surgery

KW - stereotactic body radiation therapy

KW - surgery

KW - systemic disease

KW - travel

N2 - Purpose/Objective(s): Radiotherapy, especially high-dose radiation in the form of stereotactic body radiotherapy (SBRT), may stimulate the therapeutic effect of immune checkpoint inhibitors (ICIs). Both SBRT and ICIs play an increasingly important role in the management of spine metastasis. We hypothesize that SBRT and ICI may act synergistically to improve the outcomes of patients with spine metastasis. Materials/Methods: We retrospectively reviewed patients with spine metastasis treated with SBRT at an academic center between 2009 and 2019. Patients who received ICI at any point during their disease course were compared to those with the same primary tumor types who did not receive ICI. Cox proportional hazard analyses were performed for overall survival (OS) and local control (LC), adjusting for primary tumor type, age, performance status, and prior spine surgery and/or radiation. Results: 153 patients with 194 unique spine lesions were treated with SBRT. The most common primary tumor types were renal cell carcinoma (RCC), non-small cell lung cancer (N = 48, 31% each), colorectal cancer (N = 21, 14%), melanoma and liver cancer (N = 10, 6% each). 82 patients received at least one course of ICI. The most common type of ICI was single-agent anti-PD-1 (N = 71, 88%), followed by dual anti-CTLA-4/PD-1 inhibitors (N = 14, 17%). Of patients receiving ICI, 44 (53.6%) initiated ICI before SBRT, and 38 (46.4%) received ICI after SBRT. The median survival (MS) after SBRT was 14.8 months (95% confidence interval [CI] 11.4-19.6). On univariable analysis, MS was significantly longer for patients starting ICI after SBRT (27.3 months, 95% CI 16.0-33.9) than for those who did not receive any ICI (MS 11.3 months, 95% CI 8.9-15.8, P = 0.04). Patients with RCC (HR 0.67, 95% CI 0.45-1.00, P = 0.05) and higher body mass index (BMI, HR 0.97 as continuous variable, 95% CI 0.93-1.00, P = 0.05) trended toward longer OS. On multivariable analysis after adjusting for primary tumor type, performance status and BMI, only ICI treatment after SBRT was significantly associated with increased survival (HR 0.62, 95% CI 0.39-0.98, P = 0.04). LC after SBRT was numerically higher in patients receiving ICI (1-year LC 91.3%, 95% CI 82.4-95.8%; 2-year 79.7%, 95% CI 65.7-88.5%), than those who did not (1-year 81.5%, 95% CI 65.9-90.5%; 2-year LC 55.5%, 95% CI 35.6-71.4%), although this was not statistically significant (P = 0.18). ICI Initiation before SBRT trended toward superior LC compared to no ICI (multivariable HR 0.49, 95% CI 0.22-1.08, P = 0.08). Both epidural disease and paraspinal extension were associated with worse LC on univariable and multivariable analyses. Conclusion: ICIs may enhance survival and tumor control after SBRT for patients with spine metastasis. Post-SBRT ICI may improve systemic disease control and survival, while pre-SBRT ICI may augment LC. Further research is needed to maximize the synergy between these two treatment modalities. Author Disclosure: X. Chen: None. M.C. LeCompte: None. L.R. Kleinberg: Research Grant; Novartis, Novocure, Accuray, Arbor. Honoraria; Accuray. Advisory Board; Novocure. Travel Expenses; Accuray. R.K. Hales: Research Grant; Genentech. K. Voong: Research Grant; Radiation Oncology Institute, Lung Cancer Research Foundation, Canon, Inc. P. Forde: Research Grant; Bristol Myers-Squibb, AstraZeneca, Kyowa-Kirin, Novartis. Honoraria; Bristol Myers-Squibb, AstraZeneca, Novartis, Merck, AbbVie, EMD, Inivata. J.R. Brahmer: Research Grant; AstraZeneca, Bristol Myers Squibb, Genentech/Roche, Merck, RAPT Therapeutics, Revolution Medicines. Consultant; Amgen, Bristol Myers Squibb, Genentech, Eli Lilly, GlaxoSmithKline, Merck, Sanofi. M. Markowski: Honoraria; Clovis Oncology, Exelixis. D.M. Ryan II: None. L. Lo: None. D.M. Sciubba: Consultant; Medtronic, DePuy-Synthes, Baxter. K.J. Redmond: Research Grant; Elekta AB, Accuray. Honoraria; AstraZeneca, Accuray. Travel Expenses; Elekta AB, Accuray.

ER -

TY - JOUR

M3 - Article

Y1 - 2021

VL - 111

IS - 3

SP - e604

EP - e605

SN - 1879-355X

JF - International journal of radiation oncology, biology, physics

JO - Int J Radiat Oncol Biol Phys

UR - https://www.embase.com/search/results?subaction=viewrecord&id=L636624155&from=export

U2 - L636624155

C5 - 34701854

DB - Medline

U3 - 2021-12-16

L2 - http://dx.doi.org/10.1016/j.ijrobp.2021.07.1613

DO - 10.1016/j.ijrobp.2021.07.1613

A1 - Chen, X.

A1 - LeCompte, M.C.

A1 - Kleinberg, L.R.

A1 - Hales, R.K.

A1 - Voong, K.R.

A1 - Forde, P.

A1 - Brahmer, J.R.

A1 - Markowski, M.

A1 - Ryan, D.M.

A1 - Lo, L.

A1 - Sciubba, D.M.

A1 - Redmond, K.J.

M1 - (Chen X.; LeCompte M.C.; Kleinberg L.R.; Hales R.K.; Voong K.R.; Redmond K.J.) Department of Radiation Oncology and Molecular Radiation Sciences, Johns Hopkins University School of Medicine, MD, Baltimore, United States

M1 - (Forde P.; Brahmer J.R.; Markowski M.) Department of Oncology, Sidney Kimmel Comprehensive Cancer Center, Johns Hopkins University School of Medicine, MD, Baltimore, United States

M1 - (Ryan D.M.) Department of Radiology and Radiological Science, Johns Hopkins University School of Medicine, MD, Baltimore, United States

M1 - (Lo L.; Sciubba D.M.) Department of Neurosurgery, Johns Hopkins University School of Medicine, MD, Baltimore, United States

T1 - Immune Checkpoint Inhibitors Improve Survival and Local Control in Patients With Spine Metastasis After Stereotactic Body Radiotherapy

LA - English

KW - adult

KW - article

KW - body mass

KW - body weight

KW - cancer control

KW - cancer inhibition

KW - cancer patient

KW - cancer radiotherapy

KW - cancer survival

KW - colorectal cancer

KW - consultation

KW - controlled study

KW - drug therapy

KW - female

KW - human

KW - liver cancer

KW - major clinical study

KW - male

KW - median survival time

KW - melanoma

KW - non small cell lung cancer

KW - overall survival

KW - radiation oncology

KW - renal cell carcinoma

KW - retrospective study

KW - spine metastasis

KW - spine surgery

KW - stereotactic body radiation therapy

KW - surgery

KW - systemic disease

KW - travel

KW - cytotoxic T lymphocyte antigen 4

KW - endogenous compound

KW - immune checkpoint inhibitor

KW - programmed death 1 receptor

N2 - PURPOSE/OBJECTIVE(S): Radiotherapy, especially high-dose radiation in the form of stereotactic body radiotherapy (SBRT), may stimulate the therapeutic effect of immune checkpoint inhibitors (ICIs). Both SBRT and ICIs play an increasingly important role in the management of spine metastasis. We hypothesize that SBRT and ICI may act synergistically to improve the outcomes of patients with spine metastasis. MATERIALS/METHODS: We retrospectively reviewed patients with spine metastasis treated with SBRT at an academic center between 2009 and 2019. Patients who received ICI at any point during their disease course were compared to those with the same primary tumor types who did not receive ICI. Cox proportional hazard analyses were performed for overall survival (OS) and local control (LC), adjusting for primary tumor type, age, performance status, and prior spine surgery and/or radiation. RESULTS: 153 patients with 194 unique spine lesions were treated with SBRT. The most common primary tumor types were renal cell carcinoma (RCC), non-small cell lung cancer (N = 48, 31% each), colorectal cancer (N = 21, 14%), melanoma and liver cancer (N = 10, 6% each). 82 patients received at least one course of ICI. The most common type of ICI was single-agent anti-PD-1 (N = 71, 88%), followed by dual anti-CTLA-4/PD-1 inhibitors (N = 14, 17%). Of patients receiving ICI, 44 (53.6%) initiated ICI before SBRT, and 38 (46.4%) received ICI after SBRT. The median survival (MS) after SBRT was 14.8 months (95% confidence interval [CI] 11.4-19.6). On univariable analysis, MS was significantly longer for patients starting ICI after SBRT (27.3 months, 95% CI 16.0-33.9) than for those who did not receive any ICI (MS 11.3 months, 95% CI 8.9-15.8, P = 0.04). Patients with RCC (HR 0.67, 95% CI 0.45-1.00, P = 0.05) and higher body mass index (BMI, HR 0.97 as continuous variable, 95% CI 0.93-1.00, P = 0.05) trended toward longer OS. On multivariable analysis after adjusting for primary tumor type, performance status and BMI, only ICI treatment after SBRT was significantly associated with increased survival (HR 0.62, 95% CI 0.39-0.98, P = 0.04). LC after SBRT was numerically higher in patients receiving ICI (1-year LC 91.3%, 95% CI 82.4-95.8%; 2-year 79.7%, 95% CI 65.7-88.5%), than those who did not (1-year 81.5%, 95% CI 65.9-90.5%; 2-year LC 55.5%, 95% CI 35.6-71.4%), although this was not statistically significant (P = 0.18). ICI Initiation before SBRT trended toward superior LC compared to no ICI (multivariable HR 0.49, 95% CI 0.22-1.08, P = 0.08). Both epidural disease and paraspinal extension were associated with worse LC on univariable and multivariable analyses. CONCLUSION: ICIs may enhance survival and tumor control after SBRT for patients with spine metastasis. Post-SBRT ICI may improve systemic disease control and survival, while pre-SBRT ICI may augment LC. Further research is needed to maximize the synergy between these two treatment modalities. AUTHOR DISCLOSURE: X. Chen: None. M.C. LeCompte: None. L.R. Kleinberg: Research Grant; Novartis, Novocure, Accuray, Arbor. Honoraria; Accuray. Advisory Board; Novocure. Travel Expenses; Accuray. R.K. Hales: Research Grant; Genentech. K. Voong: Research Grant; Radiation Oncology Institute, Lung Cancer Research Foundation, Canon, Inc. P. Forde: Research Grant; Bristol Myers-Squibb, AstraZeneca, Kyowa-Kirin, Novartis. Honoraria; Bristol Myers-Squibb, AstraZeneca, Novartis, Merck, AbbVie, EMD, Inivata. J.R. Brahmer: Research Grant; AstraZeneca, Bristol Myers Squibb, Genentech/Roche, Merck, RAPT Therapeutics, Revolution Medicines. Consultant; Amgen, Bristol Myers Squibb, Genentech, Eli Lilly, GlaxoSmithKline, Merck, Sanofi. M. Markowski: Honoraria; Clovis Oncology, Exelixis. D.M. Ryan II: None. L. Lo: None. D.M. Sciubba: Consultant; Medtronic, DePuy-Synthes, Baxter. K.J. Redmond: Research Grant; Elekta AB, Accuray. Honoraria; AstraZeneca, Accuray. Travel Expenses; Elekta AB, Accuray.

ER -

TY - JOUR

M3 - Article

Y1 - 2021

VL - 70

IS - 10

SP - 2771

EP - 2780

SN - 1432-0851

SN - 0340-7004

JF - Cancer Immunology, Immunotherapy

JO - Cancer Immunol. Immunother.

UR - https://www.embase.com/search/results?subaction=viewrecord&id=L2010569582&from=export

U2 - L2010569582

C5 - 33625531

DB - Embase

DB - Medline

U3 - 2021-03-04

U4 - 2021-03-04

U4 - 2021-12-23

L2 - http://dx.doi.org/10.1007/s00262-021-02888-6

DO - 10.1007/s00262-021-02888-6

A1 - Guzman-Prado, Y.

A1 - Ben Shimol, J.

A1 - Samson, O.

M1 - (Guzman-Prado Y., juliguzmanprado@outlook.com) International Centre for Medical Research, Dorset, United Kingdom

M1 - (Ben Shimol J., jennifer.benshimol@gmail.com) Department of Medicine, E. Wolfson Medical Center, Holon, Israel

M1 - (Ben Shimol J., jennifer.benshimol@gmail.com) Sackler Faculty of Medicine, Tel Aviv University, Ramat Gan, Israel

M1 - (Samson O., ondsam@gmail.com) Alltrista, Christchurch, Dorset, United Kingdom

AD - Y. Guzman-Prado, International Centre for Medical Research, Dorset, United Kingdom

T1 - Sarcopenia and the risk of adverse events in patients treated with immune checkpoint inhibitors: a systematic review

LA - English

KW - cytotoxic T lymphocyte antigen 4 antibody

KW - gilvetmab

KW - immune checkpoint inhibitor

KW - ipilimumab

KW - nivolumab

KW - pembrolizumab

KW - acute kidney failure

KW - adrenal insufficiency

KW - adult

KW - adverse outcome

KW - aged

KW - arthralgia

KW - arthritis

KW - article

KW - body mass

KW - cancer immunotherapy

KW - cancer patient

KW - colitis

KW - cytopenia

KW - demography

KW - diabetic ketoacidosis

KW - diarrhea

KW - female

KW - follow up

KW - hepatitis

KW - human

KW - hypophysitis

KW - hypopituitarism

KW - hypothyroidism

KW - interstitial nephritis

KW - kidney cancer

KW - liver function

KW - liver metastasis

KW - lung cancer

KW - lung metastasis

KW - male

KW - melanoma

KW - metastasis

KW - metastatic melanoma

KW - monotherapy

KW - myositis

KW - non small cell lung cancer

KW - pneumonia

KW - polyarthritis

KW - polymyositis

KW - prevalence

KW - rash

KW - renal cell carcinoma

KW - risk assessment

KW - risk factor

KW - sarcopenia

KW - side effect

KW - skin pruritus

KW - solid malignant neoplasm

KW - systematic review

KW - unspecified side effect

KW - uveitis

N2 - Background: Sarcopenia has been associated with negative clinical outcomes in cancer patients, particularly response to treatment and survival. The exponential growth in the use of immune checkpoint inhibitors (ICIs) has led to an increase in the reporting of both adverse events in general (AEs) and immune-related adverse events (irAEs), which are unintended immune-related phenomenon that take place as a result of checkpoint blockade. However, there are no systematic reviews evaluating the relationship between sarcopenia and the risk of developing AEs and irAEs in cancer patients on ICI therapies. Methods: PubMed, MEDLINE, Embase, Cochrane and grey literature, repositories, websites Open Grey, Google Scholar, and abstracts of major international congresses were searched up to April 2020 for observational studies on sarcopenia and both AEs and irAEs in patients treated with ICIs. Study quality was assessed with The Newcastle–Ottawa quality assessment scale. PROSPERO registration number: CRD42020197178. Results: One hundred and thirteen discrete articles were identified. Seven studies were included after evaluation of the eligibility criteria. Important sources of heterogeneity including the specific cut-points defining sarcopenia, sample size, inclusion and exclusion criteria, treatment regimen, and baseline demographics were evaluated and accounted for accordingly. Conclusion: Most of the included studies showed an increased risk of AEs with use of ICIs in cancer patients with sarcopenia, and in the majority of these, the increase was statistically significant. Due to the small number of available studies and the expanding use of ICIs, additional research is warranted.

ER -

TY - JOUR

M3 - Article

Y1 - 2021

VL - 28

IS - 11

SP - 6294

EP - 6306

SN - 1534-4681

SN - 1068-9265

JF - Annals of Surgical Oncology

JO - Ann. Surg. Oncol.

UR - https://www.embase.com/search/results?subaction=viewrecord&id=L2011130825&from=export

U2 - L2011130825

C5 - 33839975

DB - Embase

DB - Medline

U3 - 2021-04-26

U4 - 2021-04-26

U4 - 2021-10-26

L2 - http://dx.doi.org/10.1245/s10434-021-09862-7

DO - 10.1245/s10434-021-09862-7

A1 - Dubois, C.

A1 - Nuytens, F.

A1 - Behal, H.

A1 - Gronnier, C.

A1 - Manceau, G.

A1 - Warlaumont, M.

A1 - Duhamel, A.

A1 - Denost, Q.

A1 - Honoré, C.

A1 - Facy, O.

A1 - Tuech, J.-J.

A1 - Tiberio, G.

A1 - Brigand, C.

A1 - Bail, J.-P.

A1 - Salame, E.

A1 - Meunier, B.

A1 - Lefevre, J.H.

A1 - Mathonnet, M.

A1 - Idrissi, M.S.

A1 - Renaud, F.

A1 - Piessen, G.

A1 - Browet, F.

A1 - Sabbagh, C.

A1 - Regimbeau, J.-M.

A1 - Lermite, E.

A1 - Hamy, A.

A1 - Kraft, K.

A1 - Douard, R.

A1 - Wind, P.

A1 - Gersen-Cherdieu, H.

A1 - Collet, D.

A1 - Cabau, M.

A1 - Laurent, C.

A1 - Rullier, E.

A1 - Coniglio, A.

A1 - Gancel, C.-H.

A1 - Badic, B.

A1 - Ouedraogo, G.

A1 - Beuran, M.

A1 - Brams, A.

A1 - Kanor, M.

A1 - Louis, C.

A1 - Russier, Y.

A1 - Panis, Y.

A1 - Maggiori, L.

A1 - Caille, C.

A1 - Tuyeras, G.

A1 - Msika, S.

A1 - De Magistris, L.

A1 - Perrin, T.

A1 - Rat, P.

A1 - Deballon, P.O.

A1 - Meignie, P.

A1 - Bronner, J.F.

A1 - Moline, J.

A1 - Mondersert, C.

A1 - Caamano, A.

A1 - Arvieux, C.

A1 - Faucheron, J.-L.

A1 - Letoublon, C.

A1 - Guillaud, A.

A1 - Lardenois, S.

A1 - Nuss, J.-M.

A1 - Chevillotte, P.

A1 - Vinatier, E.

A1 - Tessier, W.

A1 - Caiazzo, R.

A1 - Pattou, F.

A1 - Lamande, N.

A1 - Jafari, M.

A1 - Decanter, G.

A1 - Paraf, F.

A1 - Alyami, M.

A1 - Vaudoyer, D.

A1 - Passot, G.

A1 - Glehen, O.

A1 - Demian, H.

A1 - Ducerf, C.

A1 - Mabrut, J.-Y.

A1 - Rivoire, M.

A1 - Garbit, V.

A1 - Leclercq, T.

A1 - Loire, J.

A1 - Raspado, O.

A1 - Le Huu Nho, R.

A1 - Ouaissi, M.

A1 - Sieleznef, I.

A1 - Sastre, B.

A1 - Pol, B.

A1 - Campanile, M.

A1 - Hamed, S.

A1 - Hardwigsen, J.

A1 - Le Treut, Y.-P.

A1 - Carbonnel, G.

A1 - de Saint Roman, C.

A1 - Tréot, M.

A1 - Sockeel, P.

A1 - Tourreau, G.

A1 - Baltzopoulos, V.

A1 - Mourregot, A.

A1 - Rouanet, P.

A1 - Bresler, L.

A1 - Senellart, P.

A1 - Meurette, G.

A1 - Lehur, P.A.

A1 - Regenet, N.

A1 - Casanova, V.

A1 - Schneck, A.S.

A1 - Ianelli, A.

A1 - Gugenheim, J.

A1 - Karoui, M.

A1 - Vaillant, J.C.

A1 - Bachet, J.B.

A1 - Hannoun, L.

A1 - Lupinacci, R.

A1 - Menegaux, F.

A1 - Tresallet, C.

A1 - Brouquet, A.

A1 - Benoist, S.

A1 - Penna, C.

A1 - Balladur, P.

A1 - Parc, Y.

A1 - Paye, F.

A1 - Chafai, N.

A1 - Tiret, E.

A1 - Cattan, P.

A1 - Sarfati, E.

A1 - Benhaim, L.

A1 - Sista, F.

A1 - Houry, S.

A1 - Mariani, P.

A1 - Severino, B.U.

A1 - Gayet, B.

A1 - Perniceni, T.

A1 - Richer, J.-P.

A1 - Faure, J.P.

A1 - Kraimps, J.L.

A1 - Deguelte-Lardiere, S.

A1 - Bouche, O.

A1 - Kianmanesch, R.

A1 - Le Clec’h, C.

A1 - Sulpice, L.

A1 - Meunier, B.

A1 - Boudjema, K.

A1 - Huet, E.

A1 - Scotte, M.

A1 - Michot, F.

A1 - Sole, T.

A1 - Costanza, C.

A1 - Vermesch, C.

A1 - Bruno, S.

A1 - Porcheron, J.

A1 - Raharimanantsoa, M.

A1 - Pessaux, P.

A1 - Perretta, S.

A1 - Mutter, D.

A1 - Mercoli, H.

A1 - Klipfel, A.

A1 - Triki, E.H.

A1 - Romain, B.

A1 - Dragomir, S.

A1 - Chilintseva, N.

A1 - Olliern, J.C.

A1 - Rohr, S.

A1 - Rault, A.

A1 - Julio, C.H.

A1 - du Rieu, M.C.

A1 - Carrere, N.

A1 - Pradère, B.

A1 - Senellart, P.

A1 - Thébault, B.

A1 - De Manzini, N.

A1 - Henry, C.

A1 - Bonvalot, S.

M1 - (Dubois C.; Nuytens F., frederiek.nuytens@gmail.com; Warlaumont M.; Piessen G.) Department of Digestive and Oncological Surgery, University Lille, Claude Huriez University Hospital, CHU de Lille, Lille, France

M1 - (Behal H.; Duhamel A.) University Lille, CHU Lille, ULR 2694 – METRICS : Évaluation des technologies de santé et des pratiques médicales, Lille, France

M1 - (Gronnier C.; Denost Q.) Department of Digestive Surgery, Haut Lévêque University Hospital, Bordeaux, France

M1 - (Manceau G.) Department of Digestive Surgery, Pitié-Salpêtrière University Hospital, Paris, France

M1 - (Honoré C.) Department of Surgery, Institut Gustave Roussy, Villejuif, France

M1 - (Facy O.) Department of Digestive Surgery, Dijon University Hospital, Dijon, France

M1 - (Tuech J.-J.) Department of Digestive Surgery, Charles Nicolle University Hospital, Rouen, France

M1 - (Tiberio G.) General Surgery, Department of Clinical and Experimental Sciences, University of Brescia, Brescia, Italy

M1 - (Brigand C.) Department of Digestive Surgery, Hautepierre University Hospital, Strasbourg, France

M1 - (Bail J.-P.) Department of Digestive Surgery, Brest University Hospital, Brest, France

M1 - (Salame E.) Department of Digestive Surgery, Tours University Hospital, Tours, France

M1 - (Meunier B.) Department of Digestive Surgery, Pontchailloux University Hospital, Rennes, France

M1 - (Lefevre J.H.) Department of Digestive Surgery, Sorbonne Université, AP-HP, Hôpital Saint Antoine, Paris, France

M1 - (Mathonnet M.) Department of Digestive Surgery, Limoges University Hospital, Limoges, France

M1 - (Idrissi M.S.) Department of Digestive Surgery, Clinique Claude Bernard RAMSAY, Ermont, France

M1 - (Renaud F.) Department of Pathology, Lille University Hospital, Lille, France

M1 - (Renaud F.; Piessen G.) University Lille, CNRS, Inserm, CHU Lille, UMR9020-U1277 – CANTHER – Cancer Heterogeneity, Plasticity and Resistance to Therapies, Lille, France

M1 - (Browet F.; Sabbagh C.; Regimbeau J.-M.) Department of Digestive Surgery, Amiens Picardie University Hospital, Amiens, France

M1 - (Lermite E.; Hamy A.) Department of Digestive Surgery, Angers, France

M1 - (Kraft K.) Department of Digestive Surgery, Blois, France

M1 - (Douard R.; Wind P.) Department of Digestive Surgery, Hôpital Avicennes, Bobigny, France

M1 - (Gersen-Cherdieu H.; Collet D.; Cabau M.) Department of Digestive Surgery, Haut-Levêque University Hospital, Bordeaux, France

M1 - (Laurent C.; Rullier E.) Department of Digestive Surgery, Hôpital Saint André, Bordeaux, France

M1 - (Coniglio A.) General Surgery, Department of Experimental and Clinical Sciences, University of Brescia, Brescia, Italy

M1 - (Gancel C.-H.; Badic B.) Department of Digestive Surgery, Cavale Blanche University Hospital, Brest, France

M1 - (Ouedraogo G.) Department of Digestive Surgery, Briey Hospital, Briey, France

M1 - (Beuran M.) Department of Digestive Surgery, Coltea Hospital, Bucharest, Romania

M1 - (Brams A.; Kanor M.; Louis C.; Russier Y.) Department of Digestive Surgery, Polyclinique Synergia, Carpentras, France

M1 - (Panis Y.; Maggiori L.) Department of Digestive Surgery, Beaujon University Hospital, Clichy, France

M1 - (Caille C.; Tuyeras G.; Msika S.) Department of Digestive Surgery, Louis Mourier University Hospital, Colombes, France

M1 - (De Magistris L.; Perrin T.; Rat P.; Deballon P.O.) Department of Digestive Surgery, Dijon, France

M1 - (Meignie P.) Department of Digestive Surgery, Douai Hospital, France

M1 - (Bronner J.F.; Moline J.) Department of Digestive Surgery, Clinique de la Ligne Bleue, Epinal, France

M1 - (Mondersert C.) Department of Digestive Surgery, Forez Hospital, Feurs, France

M1 - (Caamano A.) Department of Digestive Surgery, Furiani Polyclinic, Furiani, France

M1 - (Arvieux C.; Faucheron J.-L.; Letoublon C.; Guillaud A.) Department of Digestive Surgery, Grenoble, France

M1 - (Lardenois S.; Nuss J.-M.) Department of Digestive Surgery, Saint Odile Clinic, Haguenau, France

M1 - (Chevillotte P.) Department of Digestive Surgery, Fondation Hôtel-Dieu Hospital, Le Creusot, France

M1 - (Vinatier E.; Tessier W.) Department of Oncological and Digestive Surgery, Lille University Hospital, Lille, France

M1 - (Caiazzo R.; Pattou F.) Department of Digestive and Endocrine Surgery, Lille University Hospital, Lille, France

M1 - (Lamande N.; Jafari M.; Decanter G.) Department of Digestive Surgery, Lille, Oscar Lambret Center, Lille, France

M1 - (Paraf F.) Department of Digestive Surgery, Limoges, France

M1 - (Alyami M.; Vaudoyer D.; Passot G.; Glehen O.) Department of Digestive Surgery, Lyon-Sud Hospital, Lyon, France

M1 - (Demian H.; Ducerf C.; Mabrut J.-Y.) Department of Digestive Surgery, Croix-Rousse Hospital, Lyon, France

M1 - (Rivoire M.) Department of Digestive Surgery, Centre Leon Berard, Lyon, France

M1 - (Garbit V.; Leclercq T.; Loire J.; Raspado O.) Department of Digestive Surgery, Infirmerie Protestante, Lyon, France

M1 - (Le Huu Nho R.; Ouaissi M.; Sieleznef I.; Sastre B.) Department of Digestive Surgery, la Timone Hospital, Marseilles, France

M1 - (Pol B.; Campanile M.) Department of Digestive Surgery, Saint Joseph Hospital, Marseilles, France

M1 - (Hamed S.; Hardwigsen J.; Le Treut Y.-P.) Department of Digestive Surgery, la Conception Hospital, Marseilles, France

M1 - (Carbonnel G.) Department of Digestive Surgery, Mende, France

M1 - (de Saint Roman C.; Tréot M.; Sockeel P.) Department of Digestive Surgery, Legouest Hospital, Metz, France

M1 - (Tourreau G.; Baltzopoulos V.) Department of Digestive Surgery, Saint François Clinic, Montluçon, France

M1 - (Mourregot A.; Rouanet P.) Department of Digestive Surgery, Val D’Aurelle Hospital, Montpellier, France

M1 - (Bresler L.) Department of Digestive Surgery, Nancy University Hospital, Nancy, France

M1 - (Senellart P.; Meurette G.; Lehur P.A.; Regenet N.) Department of Digestive Surgery, Nantes University Hospital, Nantes, France

M1 - (Casanova V.; Schneck A.S.; Ianelli A.; Gugenheim J.) Department of Digestive Surgery, Nice, France

M1 - (Karoui M.; Vaillant J.C.; Bachet J.B.; Hannoun L.) Department of Digestive Surgery, Pitié-Salpétrière University Hospital, Paris, France

M1 - (Lupinacci R.; Menegaux F.; Tresallet C.) Department of Digestive and Visceral Surgery, Pitié-Salpétrière University Hospital, Paris, France

M1 - (Brouquet A.; Benoist S.; Penna C.) Department of Digestive Surgery, Kremlin-Bicètre Hospital, Le Kremlin-Bicêtre, France

M1 - (Balladur P.; Parc Y.; Paye F.; Chafai N.; Tiret E.) Department of Digestive Surgery, Saint-Antoine Hospital, Paris, France

M1 - (Cattan P.; Sarfati E.; Benhaim L.) Department of Digestive Surgery, Saint Louis Hospital, Paris, France

M1 - (Sista F.; Houry S.) Department of Digestive Surgery, Tenon Hospital, Paris, France

M1 - (Mariani P.) Department of Digestive Surgery, Institut Curie, Paris, France

M1 - (Severino B.U.; Gayet B.; Perniceni T.) Department of Digestive Surgery, Institut Mutualiste Montsouris, Paris, France

M1 - (Richer J.-P.; Faure J.P.; Kraimps J.L.) Department of Digestive Surgery, Poitiers, France

M1 - (Deguelte-Lardiere S.; Bouche O.; Kianmanesch R.) Department of Digestive Surgery, Reims, France

M1 - (Le Clec’h C.; Sulpice L.; Meunier B.; Boudjema K.) Department of Digestive Surgery, Rennes, France

M1 - (Huet E.; Scotte M.; Michot F.) Department of Digestive Surgery, Rouen, France

M1 - (Sole T.; Costanza C.; Vermesch C.; Bruno S.; Porcheron J.) Department of Digestive Surgery, Saint-Etienne, France

M1 - (Raharimanantsoa M.; Pessaux P.; Perretta S.; Mutter D.) Department of Digestive Surgery, New Civil Hospital, Strasbourg, France

M1 - (Mercoli H.; Klipfel A.; Triki E.H.; Romain B.; Dragomir S.; Chilintseva N.; Olliern J.C.; Rohr S.) Department of Digestive Surgery, Strasbourg, CHU, Hôpital de Hautepierre, Strasbourg, France

M1 - (Rault A.) Department of Digestive Surgery, Foch Hospital, Suresnes, France

M1 - (Julio C.H.; du Rieu M.C.; Carrere N.; Pradère B.) Department of Digestive Surgery, Purpan Hospital, Toulouse, France

M1 - (Senellart P.; Thébault B.) Department of Digestive Surgery, Tours, France

M1 - (De Manzini N.) Department of Digestive Surgery, Trieste, Italy

M1 - (Henry C.) Department of Digestive Surgery, Valence, France

M1 - (Bonvalot S.) Department of Digestive Surgery, Gustave-Roussy Institute, Villejuif, France

M1 - ()

AD - F. Nuytens, Department of Digestive and Oncological Surgery, University Lille, Claude Huriez University Hospital, CHU de Lille, Lille, France

T1 - Limited Resection Versus Pancreaticoduodenectomy for Duodenal Gastrointestinal Stromal Tumors? Enucleation Interferes in the Debate: A European Multicenter Retrospective Cohort Study

LA - English

KW - platelet derived growth factor A

KW - protein tyrosine kinase inhibitor

KW - abdominal abscess

KW - abdominal mass

KW - abdominal pain

KW - adult

KW - American Society of Anaesthesiologists score

KW - anemia

KW - anorexia

KW - article

KW - asthenia

KW - body weight loss

KW - cancer diagnosis

KW - cancer mortality

KW - cancer recurrence

KW - cancer staging

KW - cancer surgery

KW - clinical feature

KW - cohort analysis

KW - computer assisted tomography

KW - demography

KW - disease free survival

KW - duodenum tumor

KW - dying

KW - enucleation

KW - exon

KW - female

KW - gastrointestinal biopsy

KW - gastrointestinal hemorrhage

KW - gastrointestinal stromal tumor

KW - heart failure

KW - histopathology

KW - human

KW - human tissue

KW - intermethod comparison

KW - jaundice

KW - length of stay

KW - long term survival

KW - lung disease

KW - major clinical study

KW - male

KW - micturition disorder

KW - middle aged

KW - mitosis index

KW - morbidity

KW - multicenter study

KW - neoadjuvant chemotherapy

KW - neurologic disease

KW - observational study

KW - oncogene c kit

KW - operation duration

KW - overall survival

KW - pancreaticoduodenectomy

KW - pancreaticojejunostomy

KW - peroperative complication

KW - pneumonia

KW - postoperative complication

KW - reoperation

KW - retrospective study

KW - segmental duodenectomy

KW - segmentectomy

KW - short term survival

KW - surgical approach

KW - surgical mortality

KW - surgical technique

KW - thromboembolism

KW - tumor biopsy

KW - tumor enucleation

KW - tumor localization

KW - tumor perforation

KW - tumor volume

KW - wedge resection

N2 - Background: The optimal surgical procedure for duodenal gastrointestinal stromal tumors (D-GISTs) remains poorly defined. Pancreaticoduodenectomy (PD) allows for a wide resection but is associated with a high morbidity rate. Objectives: The aim of this study was to compare the short- and long-term outcomes of PD versus limited resection (LR) for D-GISTs and to evaluate the role of tumor enucleation (EN). Methods: In this retrospective European multicenter cohort study, 100 patients who underwent resection for D-GIST between 2001 and 2013 were compared between PD (n = 19) and LR (n = 81). LR included segmental duodenectomy (n = 47), wedge resection (n = 21), or EN (n = 13). The primary objective was to evaluate disease-free survival (DFS) between the groups, while the secondary objectives were to analyze the overall morbidity and mortality, radicality of resection, and 5-year overall survival (OS) and recurrence rates between groups. Furthermore, the short- and long-term outcomes of EN were evaluated. Results: Baseline characteristics were comparable between the PD and LR groups, except for a more frequent D2 tumor location in the PD group (68.3% vs. 29.6%; p = 0.016). Postoperative morbidity was higher after PD (68.4% vs. 23.5%; p < 0.001). OS (p = 0.70) and DFS (p = 0.64) were comparable after adjustment for D2 location and adjuvant therapy rate. EN was performed more in American Society of Anesthesiologists (ASA) stage III/IV patients with tumors < 5 cm and was associated with a 5-year OS rate of 84.6%, without any disease recurrences. Conclusions: For D-GISTs, LR should be the procedure of choice due to lower morbidity and similar oncological outcomes compared with PD. In selected patients, EN appears to be associated with equivalent short- and long-term outcomes. Based on these results, a surgical treatment algorithm is proposed.

ER -

TY - JOUR

M3 - Article

Y1 - 2021

VL - 6

IS - 5

SN - 2059-7029

JF - ESMO Open

JO - ESMO Open

UR - https://www.embase.com/search/results?subaction=viewrecord&id=L2014354546&from=export

U2 - L2014354546

C5 - 34481329

DB - Embase

DB - Medline

U3 - 2021-12-07

L2 - http://dx.doi.org/10.1016/j.esmoop.2021.100254

DO - 10.1016/j.esmoop.2021.100254

A1 - Mountzios, G.

A1 - Samantas, E.

A1 - Senghas, K.

A1 - Zervas, E.

A1 - Krisam, J.

A1 - Samitas, K.

A1 - Bozorgmehr, F.

A1 - Kuon, J.

A1 - Agelaki, S.

A1 - Baka, S.

A1 - Athanasiadis, I.

A1 - Gaissmaier, L.

A1 - Elshiaty, M.

A1 - Daniello, L.

A1 - Christopoulou, A.

A1 - Pentheroudakis, G.

A1 - Lianos, E.

A1 - Linardou, H.

A1 - Kriegsmann, K.

A1 - Kosmidis, P.

A1 - El Shafie, R.

A1 - Kriegsmann, M.

A1 - Psyrri, A.

A1 - Andreadis, C.

A1 - Fountzilas, E.

A1 - Heussel, C.-P.

A1 - Herth, F.J.

A1 - Winter, H.

A1 - Emmanouilides, C.

A1 - Oikonomopoulos, G.

A1 - Meister, M.

A1 - Muley, T.

A1 - Bischoff, H.

A1 - Saridaki, Z.

A1 - Razis, E.

A1 - Perdikouri, E.-I.

A1 - Stenzinger, A.

A1 - Boukovinas, I.

A1 - Reck, M.

A1 - Syrigos, K.

A1 - Thomas, M.

A1 - Christopoulos, P.

M1 - (Mountzios G., gmountzios@gmail.com) Fourth Oncology Department and Clinical Trials Unit, Henry Dunant Hospital Center, Athens, Greece

M1 - (Samantas E.; Oikonomopoulos G.) Second Oncology Department, Metropolitan Hospital, Pireaus, Athens, Greece

M1 - (Senghas K.; Bozorgmehr F.; Kuon J.; Gaissmaier L.; Elshiaty M.; Daniello L.; Heussel C.-P.; Herth F.J.; Winter H.; Meister M.; Muley T.; Bischoff H.; Thomas M.; Christopoulos P., petros.christopoulos@med.uni-heidelberg.de) Thoraxklinik and National Center for Tumor Diseases at Heidelberg University Hospital, Heidelberg, Germany

M1 - (Zervas E.) 7th Pneumonology Department ‘Sotiria’ Hospital, Athens, Greece

M1 - (Krisam J.) Institute of Medical Biometry and Statistics, Heidelberg University Hospital, Heidelberg, Germany

M1 - (Samitas K.; Agelaki S.) Department of Medical Oncology, University of Irakleion School of Medicine, Iraklion, Greece

M1 - (Baka S.; Emmanouilides C.) Department of Medical Oncology, Interbalkan Medical Center, Thessaloniki, Greece

M1 - (Athanasiadis I.) Department of Medical Oncology, ‘Mitera’ Hospital, Athens, Greece

M1 - (Gaissmaier L.; Elshiaty M.; Kriegsmann M.; Heussel C.-P.; Herth F.J.; Winter H.; Meister M.; Muley T.; Stenzinger A.; Thomas M.; Christopoulos P., petros.christopoulos@med.uni-heidelberg.de) Translational Lung Research Center Heidelberg, German Center for Lung Research (DZL), Heidelberg, Germany

M1 - (Christopoulou A.) Department of Medical Oncology, General Hospital of Patras ‘Agios Andreas’, Patras, Greece

M1 - (Pentheroudakis G.) Department of Medical Oncology, University of Ioannina School of Medicine, Ioannina, Greece

M1 - (Lianos E.) Department of Medical Oncology, ‘Metaxa’ Cancer Hospital, Pireaus, Greece

M1 - (Linardou H.) Fourth Oncology Department, Metropolitan Hospital, Pireaus, Athens, Greece

M1 - (Kriegsmann K.; Kriegsmann M.; Stenzinger A.) Department of Hematology, Oncology and Rheumatology, University Hospital Heidelberg, Heidelberg, Germany

M1 - (Kosmidis P.) Second Oncology Department, ‘Hygeia’ Hospital, Athens, Greece

M1 - (El Shafie R.) Department of Radiation Oncology, Heidelberg University Hospital, Heidelberg, Germany

M1 - (Psyrri A.) Department of Medical Oncology, ‘Attikon’ University Hospital, Athens, Greece

M1 - (Andreadis C.) Third Department of Medical Oncology, ‘Theageneion’ Cancer Hospital, Thessaloniki, Greece

M1 - (Fountzilas E.) Department of Medical Oncology, ‘Euromedica’ Clinic, Thessaloniki, Greece

M1 - (Saridaki Z.) Department of Medical Oncology, ‘Asclepius’ Clinic, Iraklion, Greece

M1 - (Razis E.) Third Department of Medical Oncology, Hygeia Hospital, Athens, Greece

M1 - (Perdikouri E.-I.) Department of Medical Oncology, ‘Achilopouleio’ General Hospital of Volos, Volos, Greece

M1 - (Boukovinas I.) Department of Medical Oncology, ‘Bioclinica’ Hospital, Thessaloniki, Greece

M1 - (Reck M.) LungenClinic Großhansdorf GmbH, Großhansdorf, Germany

M1 - (Reck M.) Airway Research Center North, German Center for Lung Research, Großhansdorf, Germany

M1 - (Syrigos K.) Department of Medical Oncology, Sotiria General Hospital of Athens, Athens, Greece

AD - G. Mountzios, Fourth Oncology Department and Clinical Trials Unit, Henry Dunant Hospital Center, Mesogeion 107, PC 115 27, Athens, Greece. Tel: +30-6983-519-989; Fax: +30-2107-715-690,

AD - P. Christopoulos, Thoraxklinik and National Center for Tumor Diseases at Heidelberg University Hospital, Röntgenstr. 1, 69126 Heidelberg, Germany. Tel: +49-6221-396-1371; Fax: +49-6221-396-1362,

T1 - Association of the advanced lung cancer inflammation index (ALI) with immune checkpoint inhibitor efficacy in patients with advanced non-small-cell lung cancer

LA - English

KW - adult

KW - advanced cancer

KW - article

KW - calculation

KW - cancer chemotherapy

KW - cancer combination chemotherapy

KW - cancer inhibition

KW - cancer patient

KW - cancer prognosis

KW - cancer staging

KW - cancer survival

KW - clinical outcome

KW - cohort analysis

KW - controlled study

KW - cross validation

KW - drug therapy

KW - female

KW - Germany

KW - Greece

KW - human

KW - immunotherapy

KW - inflammation

KW - major clinical study

KW - male

KW - monotherapy

KW - multicenter study

KW - neutrophil lymphocyte ratio

KW - non small cell lung cancer

KW - overall survival

KW - protein function

KW - retrospective study

KW - biological marker

KW - endogenous compound

KW - immune checkpoint inhibitor

KW - platinum

KW - programmed death 1 ligand 1

N2 - Background: The advanced lung cancer inflammation index [ALI: body mass index × serum albumin/neutrophil-to-lymphocyte ratio (NLR)] reflects systemic host inflammation, and is easily reproducible. We hypothesized that ALI could assist guidance of non-small-cell lung cancer (NSCLC) treatment with immune checkpoint inhibitors (ICIs). Patients and methods: This retrospective study included 672 stage IV NSCLC patients treated with programmed death-ligand 1 (PD-L1) inhibitors alone or in combination with chemotherapy in 25 centers in Greece and Germany, and a control cohort of 444 stage IV NSCLC patients treated with platinum-based chemotherapy without subsequent targeted or immunotherapy drugs. The association of clinical outcomes with biomarkers was analyzed with Cox regression models, including cross-validation by calculation of the Harrell's C-index. Results: High ALI values (>18) were significantly associated with longer overall survival (OS) for patients receiving ICI monotherapy [hazard ratio (HR) = 0.402, P < 0.0001, n = 460], but not chemo-immunotherapy (HR = 0.624, P = 0.111, n = 212). Similar positive correlations for ALI were observed for objective response rate (36% versus 24%, P = 0.008) and time-on-treatment (HR = 0.52, P < 0.001), in case of ICI monotherapy only. In the control cohort of chemotherapy, the association between ALI and OS was weaker (HR = 0.694, P = 0.0002), and showed a significant interaction with the type of treatment (ICI monotherapy versus chemotherapy, P < 0.0001) upon combined analysis of the two cohorts. In multivariate analysis, ALI had a stronger predictive effect than NLR, PD-L1 tumor proportion score, lung immune prognostic index, and EPSILoN scores. Among patients with PD-L1 tumor proportion score ≥50% receiving first-line ICI monotherapy, a high ALI score >18 identified a subset with longer OS and time-on-treatment (median 35 and 16 months, respectively), similar to these under chemo-immunotherapy. Conclusions: The ALI score is a powerful prognostic and predictive biomarker for patients with advanced NSCLC treated with PD-L1 inhibitors alone, but not in combination with chemotherapy. Its association with outcomes appears to be stronger than that of other widely used parameters. For PD-L1-high patients, an ALI score >18 could assist the selection of cases that do not need addition of chemotherapy.

ER -

TY - JOUR

M3 - Conference Abstract

Y1 - 2021

VL - 16

IS - 10

SP - S1081

SN - 1556-1380

SN - 1556-0864

JF - Journal of Thoracic Oncology

JO - J. Thorac. Oncol.

UR - https://www.embase.com/search/results?subaction=viewrecord&id=L2015170176&from=export

U2 - L2015170176

DB - Embase

U4 - 2021-10-22

L2 - http://dx.doi.org/10.1016/j.jtho.2021.08.462

DO - 10.1016/j.jtho.2021.08.462

A1 - Barba Joaquín, A.

A1 - Mosquera, J.

A1 - Riudavets Melià, M.

A1 - Gomez-Randulfe, M.I.

A1 - García Campelo, M.R.

A1 - Sullivan, I.

A1 - Serra Lopez, J.

A1 - Aguado, M.

A1 - Piedra, A.

A1 - Majem, M.

M1 - (Barba Joaquín A.; Riudavets Melià M.; Sullivan I.; Serra Lopez J.; Aguado M.; Piedra A.) Medical Oncology, Hospital de La Santa Creu I Sant Pau, Barcelona, Spain

M1 - (Mosquera J.; Gomez-Randulfe M.I.; García Campelo M.R.) Medical Oncology, Hospital Universitario A Coruña, A Coruña, Spain

M1 - (Majem M.) Hospital de La Santa Creu I Sant Pau, Barcelona, Spain

T1 - P42.03 Predictive Factors of Response to PD-(L)1 Inhibitors in Patients With Advanced Non-Small Cell Lung and High PD-L1 Expression

LA - English

KW - endogenous compound

KW - programmed death 1 ligand 1

KW - advanced cancer

KW - aged

KW - bivariate analysis

KW - body mass

KW - bone metastasis

KW - cancer chemotherapy

KW - cancer inhibition

KW - cancer patient

KW - cancer survival

KW - clinical feature

KW - conference abstract

KW - controlled study

KW - current smoker

KW - ex-smoker

KW - female

KW - follow up

KW - gene expression

KW - histology

KW - histopathology

KW - human

KW - human tissue

KW - immunotherapy

KW - Kaplan Meier method

KW - log rank test

KW - major clinical study

KW - male

KW - monotherapy

KW - non small cell lung cancer

KW - obesity

KW - overall response rate

KW - protein expression

KW - retrospective study
[truncated: 903,481 more chars]
